# Supplementary material for: Boron-mediated directed aromatic C–H hydroxylation
Source: Nat Commun. 2020 Mar 12;11:1316. doi: 10.1038/s41467-020-15207-x (PMC7067857; doi:10.1038/s41467-020-15207-x)
Supplement: Supplementary file 1 — Supplementary Information [file 41467_2020_15207_MOESM1_ESM.pdf]

Supplementary Information

Boron-Mediated Directed Aromatic C–H Hydroxylation

Jiahang Lv et al.

Supplementary Methods

General experimental methods

All reactions were carried out in flame-dried 25 mL Schlenk tubes with Teflon screw caps under argon. BBr_3 (1 M in DCM) was purchased from TCI. BBr_3 (99.9%) was purchased from Adamas. Unless otherwise stated, the concentration of BBr_3 used in all experiments was 1M in DCM. Other reagents and solvents were directly used from the supplier without further purification unless noted. All new compounds were fully characterized. ^{1}H, ^{13}C, and ^{19}F NMR spectra were recorded on a Bruker AVANCE III 400 MHz or 500 MHz spectrometer. Chemical shifts (δ values) were reported in ppm with $CDCl_3$ (7.26 and 77.00 ppm for ^{1}H and ^{13}C respectively) or DMSO-d_6 (2.50 and 39.50 ppm for ^{1}H and ^{13}C respectively). Data are reported as s = singlet, d = doublet, t = triplet, q = quartet, m = multiplet, brs = broad signal, coupling constant(s) in Hertz, integration. Mass spectra were conducted at Agilent 6540 Ultra-High-Definition (UHD) Accurate-Mass Quadrupole Time-of-Flight (Q-TOF) liquid chromatography/mass spectrometry (LC/MS) system and Thermo Scientific TRACE 1300 ISQ LT gas chromatography/mass spectrometry (GC/MS) system. IR spectra were recorded on a Bruker FT-IR spectrometer. The substrates **1-28a**, **41-45a, 46-69a**[1], **29-40a**[2] were synthesized according to the literatures.

Experimental procedures and characterization of products

Genera procedure A

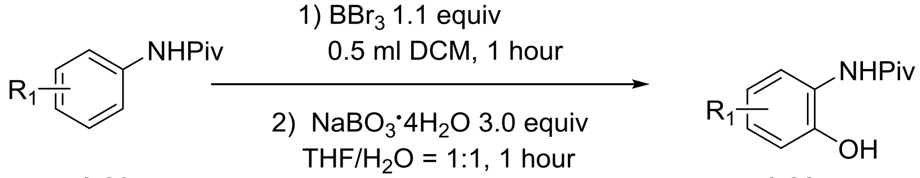

1-28a **1-28c**

Flame-dried 25 mL Schlenk tube was flushed with argon and charged with *N*-pivaloyl amide substrates **1-28a** (0.2 mmol, 1.0 equiv) and dry DCM (0.5 mL, 0.4 M). A solution of BBr_3 (1.0 M in DCM, 0.22 mL, 1.1 equiv) was added slowly under argon atmosphere. The mixture was stirred at room temperature for 1 hour. After stirring, the

solvent was removed under vacuum directly. $NaBO_3 \cdot 4H_2O$ (92.3 mg, 0.6 mmol, 3.0 equiv), 0.5 mL THF, and 0.5 mL H_2O were sequentially added to the reaction mixture and stirred at room temperature for another 1 hour (monitored by TLC). After that, the excess water was removed by filtration with $MgSO_4$ and then washed with EtOAc (10.0 mL × 3). The filtrate was collected and the solvent removed in vacuum and purified by flash column chromatography.

***N*-(4-Chloro-2-hydroxyphenyl)pivalamide (1c)** [3]

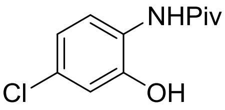

1c

According to the GP-A, **1c** was obtained as a white solid (41.5 mg, 92%), purified by flash column chromatography (PE : EtOAc = 10 : 1). **^{1}H NMR (400 MHz, Chloroform-*d*)** δ 9.06 (s, 1H), 7.57 (brs, 1H), 7.01 (d, *J* = 2.3 Hz, 1H), 6.95 (d, *J* = 8.5 Hz, 1H), 6.84 (dd, *J* = 8.5, 2.3 Hz, 1H), 1.35 (s, 9H); **^{13}C NMR (101 MHz, Chloroform-*d*)** δ 179.2, 149.7, 132.1, 124.4, 122.9, 120.4, 120.0, 39.5, 27.6; **ATR-FTIR (cm^{-1})** 3054, 1645, 1413, 937, 840 cm^{-1}; **HRMS m/z (ESI)** called for $C_{11}H_{15}ClNO_2^+$ $(M + H)^+$ 228.0786, found 228.0778.

***N*-(2-Hydroxyphenyl)pivalamide (2c)** [4]

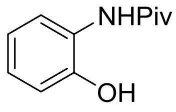

2c

According to the GP-A, **2c** was obtained as a white solid (32.8 mg, 85%), purified by flash column chromatography (PE : EtOAc = 10 : 1). **^{1}H NMR (500 MHz, Chloroform-*d*)** δ 8.81 (s, 1H), 7.61 (brs, 1H), 7.14 – 7.11 (m, 1H), 7.04 – 7.00 (m, 2H), 6.90 – 6.82 (m, 1H), 1.36 (s, 9H); **^{13}C NMR (101 MHz, Chloroform-*d*)** δ 178.9, 148.5, 126.8, 125.6, 122.0, 120.4, 119.3, 39.5, 27.6; **ATR-FTIR (cm^{-1})** 3430, 2789, 1688, 1154, 989 cm^{-1}; **HRMS m/z (ESI)** called for $C_{11}H_{16}NO_2^+$ $(M + H)^+$ 194.1176, found 194.1171.

***N*-(2-Hydroxy-4-methylphenyl)pivalamide (3c)** [4]

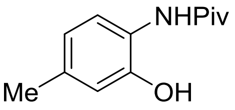

3c

According to the GP-A, **3c** was obtained as a white solid (35.1 mg, 85%), purified by flash column chromatography (PE : EtOAc = 10 : 1). **^{1}H NMR (400 MHz, DMSO-*d*$_6$)** δ 9.62 (brs, 1H), 8.53 (brs, 1H), 7.58 (d, *J* = 8.1 Hz, 1H), 6.69 (d, *J* = 1.4 Hz, 1H), 6.61 – 6.57 (m, 1H), 2.20 (s, 3H), 1.23 (s, 9H); **^{13}C NMR (101 MHz, DMSO-*d*$_6$)** δ 176.8, 148.3, 134.4, 124.3, 122.5, 120.0, 116.7, 39.5, 27.8, 21.1; **ATR-FTIR (cm^{-1})** 3427, 1666, 1052, 822 cm^{-1}; **HRMS m/z (ESI)** called for $C_{12}H_{18}NO_2^+$ (M + H)$^+$ 208.1332, found 208.1329.

***N*-(2-Hydroxy-5-methylphenyl)pivalamide (4c)**

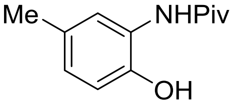

4c

According to the GP-A, **4c** was obtained as a white solid (40.1 mg, 98%), purified by flash column chromatography (PE : EtOAc = 10 : 1). **^{1}H NMR (400 MHz, DMSO-*d*$_6$)** δ 9.49 (s, 1H), 8.53 (brs, 1H), 7.59 (s, 1H), 6.75 (d, *J* = 1.2 Hz, 2H), 2.19 (s, 3H), 1.23 (s, 9H); **^{13}C NMR (101 MHz, DMSO-*d*$_6$)** δ 176.9, 145.9, 128.1, 126.6, 125.3, 122.8, 115.8, 39.6, 27.8, 20.9; **ATR-FTIR (cm^{-1})** 3384, 1686, 1571, 1263, 1062, 857, 732 cm^{-1}; **HRMS m/z (ESI)** called for $C_{12}H_{18}NO_2^+$ (M + H)$^+$ 208.1332, found 208.1327.

***N*-(2-Hydroxy-6-methylphenyl)pivalamide (5c)**

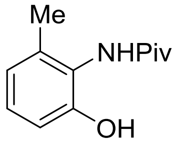

5c

According to the GP-A, **5c** was obtained as a white solid (36.8 mg, 89%), purified by flash column chromatography (PE : EtOAc = 10 : 1). **^{1}H NMR (400 MHz, Chloroform-*d*)** δ 8.57 (s, 1H), 7.40 (brs, 1H), 7.07 (t, *J* = 7.8 Hz, 1H), 6.92 (dd, *J* = 8.2, 1.5 Hz, 1H), 6.76 (ddd, *J* = 7.4, 1.6, 0.8 Hz, 1H), 2.28 (s, 3H), 1.39 (s, 9H); **^{13}C NMR (101 MHz, Chloroform-*d*)** δ 179.0, 150.2, 130.1, 127.2, 124.3, 122.2, 118.4, 39.8, 27.8, 18.1; **ATR-FTIR (cm^{-1})** 3295, 1625, 1473, 1208, 960, 776 cm^{-1}; **HRMS m/z (ESI)** called for $C_{12}H_{18}NO_2^+$ (M + H)$^+$ 208.1332, found 208.1328.

***N*-(4-(*Tert*-butyl)-2-hydroxyphenyl)pivalamide (6c)**

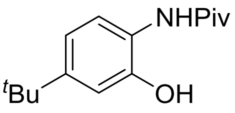

6c

According to the GP-A, **6c** was obtained as a white solid (42.1 mg, 85%), purified by flash column chromatography (PE : EtOAc = 10 : 1). **^{1}H NMR (400 MHz, DMSO-*d*$_6$)** δ 9.56 (s, 1H), 8.54 (brs, 1H), 7.59 (d, *J* = 8.4 Hz, 1H), 6.88 (d, *J* = 2.0 Hz, 1H), 6.80 (dd, *J* = 8.4, 2.0 Hz, 1H), 1.23 (s, 9H), 1.22 (s, 9H); **^{13}C NMR (101 MHz, DMSO-*d*$_6$)** δ 176.5, 147.5, 123.8, 121.8, 115.8, 112.7, 34.0, 31.2, 27.3; **ATR-FTIR (cm^{-1})** 3267, 1654, 1528, 1264, 941, 733 cm^{-1}; **HRMS m/z (ESI)** called for $C_{15}H_{24}NO_2^+$ (M + H)$^+$ 250.1802, found 250.1797

***N*-(4-Hydroxy-[1,1'-biphenyl]-3-yl)pivalamide (7c)**

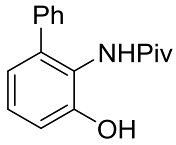

7c

According to the GP-A, **7c** was obtained as a white solid (45.2 mg, 84%), purified by flash column chromatography (PE : EtOAc = 10 : 1). **^{1}H NMR (400 MHz, DMSO-*d*$_6$)** δ 9.24 (s, 1H), 8.47 (s, 1H), 7.36 – 7.26 (m, 5H), 7.15 (t, *J* = 7.9 Hz, 1H), 6.88 (dd, *J* = 8.1, 1.4 Hz, 1H), 6.76 (dd, *J* = 7.6, 1.4 Hz, 1H), 1.02 (s, 9H); **^{13}C NMR (101 MHz, DMSO-*d*$_6$)** δ 177.2, 154.5, 142.1, 140.0, 129.2, 128.1, 127.8, 127.2, 123.5, 120.7, 115.5, 38.8, 27.7; **ATR-FTIR (cm^{-1})** 3355, 1636, 1520, 898, 703 cm^{-1}; **HRMS m/z (ESI)** called for $C_{17}H_{20}NO_2^+$ (M + H)$^+$ 270.1489, found 270.1487.

***N*-(4-Fluoro-2-hydroxyphenyl)pivalamide (8c)**

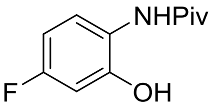

8c

According to the GP-A, **8c** was obtained as a white solid (35.6 mg, 84%), purified by flash column chromatography (PE : EtOAc = 10 : 1). **^{1}H NMR (400 MHz, DMSO-*d*$_6$)** δ 10.23 (s, 1H), 8.51 (brs, 1H), 7.65 (dd, *J* = 8.8, 6.5 Hz, 1H), 6.67 (dd, *J* = 10.3, 2.9 Hz, 1H), 6.64 – 6.58 (m, 1H), 1.22 (s, 9H); **^{13}C NMR (101 MHz, DMSO-*d*$_6$)** δ 176.5, 159.0 (d, *J* = 240.1 Hz), 149.8 (d, *J* = 11.4 Hz), 123.8 (d, *J* = 10.1 Hz), 122.9 (d, *J* = 3.03 Hz), 105.0 (d, *J* = 22.0 Hz), 102.7 (d, *J* = 25.0 Hz), 39.0 , 27.3; **^{19}F NMR (376 MHz, DMSO-*d*$_6$)** δ -117.00; **ATR-FTIR (cm^{-1})** 2974, 1670, 1330, 1120, 908, 795 cm^{-1};

HRMS m/z (ESI) called for $C_{11}H_{15}FNO_2^+$ $(M + H)^+$ 212.1081, found 212.1081.

***N*-(4-Bromo-2-hydroxyphenyl)pivalamide (9c)**

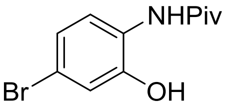

9c

According to the GP-A, **9c** was obtained as a white solid (50.1 mg, 93%), purified by flash column chromatography (PE : EtOAc = 10 : 1). **^{1}H NMR (400 MHz, DMSO-*d*$_6$)** δ 10.37 (s, 1H), 8.47 (brs, 1H), 7.73 (d, *J* = 8.6 Hz, 1H), 7.02 (d, *J* = 2.3 Hz, 1H), 6.96 (dd, *J* = 8.6, 2.3 Hz, 1H), 1.22 (s, 9H); **^{13}C NMR (101 MHz, DMSO-*d*$_6$)** δ 176.3, 149.1, 126.0, 123.5, 121.7, 117.8, 115.7, 39.2, 27.2; **ATR-FTIR (cm^{-1})** 3054, 1648, 1264, 935, 731, 703 cm^{-1}; **HRMS m/z (ESI)** called for $C_{11}H_{15}BrNO_2^+$ $(M + H)^+$ 272.0281, found 272.0276.

***N*-(2-Bromo-4-chloro-6-hydroxyphenyl)pivalamide (10c)**

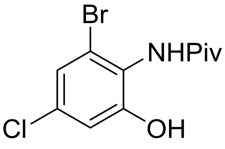

10c

According to the GP-A, **10c** was obtained as a white solid (46.2 mg, 76%), purified by flash column chromatography (PE : EtOAc = 10 : 1). **^{1}H NMR (400 MHz, DMSO-*d*$_6$)** δ 10.21 (s, 1H), 8.79 (s, 1H), 7.17 (d, *J* = 2.3 Hz, 1H), 6.90 (d, *J* = 2.3 Hz, 1H), 1.21 (s, 9H); **^{13}C NMR (101 MHz, DMSO-*d*$_6$)** δ 176.4, 155.8, 131.7, 125.0, 124.6, 121.7, 115.1, 38.6, 27.4; **ATR-FTIR (cm^{-1})** 3126, 1649, 1506, 1405, 1089, 830, 809 cm^{-1}; **HRMS m/z (ESI)** called for $C_{11}H_{14}BrClNO_2^+$ $(M + H)^+$ 305.9891, found 305.9886.

***N*-(2-Bromo-6-hydroxyphenyl)pivalamide (11c)**

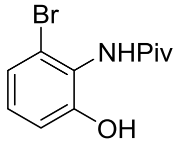

11c

According to the GP-A, **11c** was obtained as a white solid (46.1 mg, 85%), purified by flash column chromatography (PE : EtOAc = 10 : 1). **^{1}H NMR (400 MHz, DMSO-*d*$_6$)** δ 9.61 (s, 1H), 8.75 (s, 1H), 7.09 – 6.97 (m, 2H), 6.86 (dd, *J* = 7.9, 1.7 Hz, 1H), 1.23 (s, 9H); **^{13}C NMR (101 MHz, DMSO-*d*$_6$)** δ 176.4, 155.2, 128.5, 125.0, 124.3, 122.4, 115.3, 38.6, 27.4; **ATR-FTIR (cm^{-1})** 3136, 1659, 1089, 830, 809 cm^{-1}; **HRMS m/z (ESI)** called for $C_{11}H_{15}BrNO_2^+$ $(M + H)^+$ 272.0281, found 272.0276.

N-(2-Hydroxy-4-iodophenyl)pivalamide (12c)

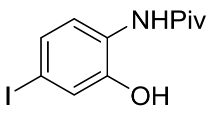

12c

According to the GP-A, **12c** was obtained as a white solid (58.1 mg, 91%), purified by flash column chromatography (PE : EtOAc = 10 : 1). **^{1}H NMR (400 MHz, DMSO-*d*$_6$)** δ 10.25 (s, 1H), 8.45 (brs, 1H), 7.60 (d, *J* = 8.4 Hz, 1H), 7.19 (d, *J* = 2.0 Hz, 1H), 7.12 (dd, *J* = 8.4, 2.0 Hz, 1H), 1.22 (s, 9H); **^{13}C NMR (101 MHz, DMSO-*d*$_6$)** δ 176.3, 148.9, 127.7, 126.5, 123.7, 123.5, 87.5, 39.2, 27.2; **ATR-FTIR (cm^{-1})** 3419, 1640, 1535, 1373, 926, 853 734 cm^{-1}; **HRMS m/z (ESI)** called for $C_{11}H_{15}INO_2^+$ $(M + H)^+$ 320.0142, found 320.0136.

N-(2-Hydroxy-5-iodophenyl)pivalamide (13c)

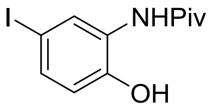

13c

According to the GP-A, **13c** was obtained as a white solid (61.1 mg, 97%), purified by flash column chromatography (PE : EtOAc = 10 : 1). **^{1}H NMR (400 MHz, DMSO-*d*$_6$)** δ 10.20 (brs, 1H), 8.46 (brs, 1H), 8.17 (d, *J* = 2.1 Hz, 1H), 7.25 (dd, *J* = 8.4, 2.1 Hz, 1H), 6.71 (d, *J* = 8.4 Hz, 1H), 1.22 (s, 9H); **^{13}C NMR (101 MHz, DMSO-*d*$_6$)** δ 176.9, 148.1, 133.2, 130.0, 128.8, 118.0, 81.1, 39.7, 27.6; **ATR-FTIR (cm^{-1})** 3432, 1668, 951, 820, 700 cm^{-1}; **HRMS m/z (ESI)** called for $C_{11}H_{15}INO_2^+$ $(M + H)^+$ 320.0142, found 320.0146.

N-(2-Hydroxy-4-(trifluoromethyl)phenyl)pivalamide (14c)

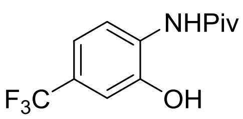

14c

The compound **14c** was performed according to the slightly modified GP-A with *N*-(4-(trifluoromethyl)phenyl)pivalamide (49.2 mg, 0.2 mmol), BBr_3 (99.9%, 500.0 mg, 2.0 mmol, 10.0 equiv) in dry DCM (0.1 mL) at room temperature under Ar for 1 hour. The solvent was removed under vaccum directly, diluted with 0.5 mL THF, $NaBO_3 \cdot 4H_2O$ (92.3 mg, 0.6 mmol) and 0.5 mL H_2O were sequentially added to the reaction mixture and stirred at room temperature for another 30 minutes. After that, the excess water was removed by filtration with $MgSO_4$ and then washed with EtOAc

(10.0 mL × 3). The filtrate was collected and the solvent removed in vacuum directly. The crude product was purified by flash column chromatography on silica gel (PE : EtOAc = 5 : 1) to afford 40.2 mg (77 %) of **14c** as a white solid. **^{1}H NMR (400 MHz, DMSO-*d*$_6$)** δ 10.88 (s, 1H), 8.55 (brs, 1H), 8.23 (d, *J* = 2.3 Hz, 1H), 7.30 (ddd, *J* = 8.5, 2.4, 0.9 Hz, 1H), 7.04 (dd, *J* = 8.4, 0.9 Hz, 1H), 1.24 (s, 9H); **^{13}C NMR (101 MHz, DMSO-*d*$_6$)** δ 176.6, 150.8, 126.8, 124.6 (q, *J* = 270.9 Hz), 121.5 (q, *J* = 3.8 Hz), 119.4 (q, *J* = 31.9 Hz), 118.2 (q, *J* = 3.9 Hz), 115.2, 39.3, 27.1; **^{19}F NMR (376 MHz, DMSO-*d*$_6$)** δ -59.85;. **ATR-FTIR (cm^{-1})** 3054, 1655, 1352, 1263, 1101,862, 731 cm^{-1}; **HRMS m/z (ESI)** called for $C_{12}H_{15}F_3NO_2^+$ $(M + H)^+$ 262.1049, found 262.1044.

N-(2-Hydroxy-5-(trifluoromethyl)phenyl)pivalamide (15c)

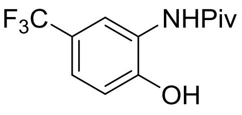

15c

The compound **15c** was performed according to the slightly modified GP-A with *N*-(3-(trifluoromethyl)phenyl)pivalamide (49.2 mg, 0.2 mmol), BBr_3 (99.9%, 500.0 mg, 2.0 mmol, 10.0 equiv) in dry DCM (0.1 mL) at room temperature under Ar for 1 hour. The solvent was removed under vaccum directly, diluted with 0.5 mL THF, $NaBO_3{\cdot}4H_2O$ (92.3 mg, 0.6 mmol) and 0.5 mL H_2O were sequentially added to the reaction mixture and stirred at room temperature for another 30 minutes. After that, the excess water was removed by filtration with $MgSO_4$ and then washed with EtOAc (10.0 mL × 3). The filtrate was collected and the solvent removed in vacuum directly. The crude product was purified by flash column chromatography on silica gel (PE : EtOAc = 5 : 1) to afford 38.1 mg (73 %) of **15c** as a white solid. **^{1}H NMR (400 MHz, DMSO-*d*$_6$)** δ 10.68 (s, 1H), 8.57 (brs, 1H), 8.09 (d, *J* = 7.9 Hz, 1H), 7.14 (d, *J* = 7.6 Hz, 2H), 1.24 (s, 9H); **^{13}C NMR (101 MHz, DMSO-*d*$_6$)** δ 176.5, 147.5, 130.2, 124.2 (q, *J* = 271.4 Hz), 124.2 (q, *J* = 31.9 Hz), 121.2, 116.0 (q, *J* = 4.1 Hz), 111.1 (q, *J* = 4.0 Hz), 39.4, 27.1; **^{19}F NMR (376 MHz, DMSO-*d*$_6$)** δ -60.59; **ATR-FTIR (cm^{-1})** 3429, 1675, 1333, 1116, 823, 760 cm^{-1}; **HRMS m/z (ESI)** called for $C_{12}H_{15}F_3NO_2^+$ $(M + H)^+$ 262.1049, found 262.1047.

Ethyl 2-(3-hydroxy-4-pivalamidophenyl)acetate (16c)

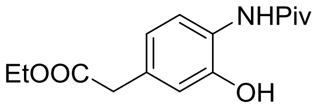

16c

According to the GP-A, **16c** was obtained as a white solid (44.6 mg, 80%), purified by flash column chromatography (PE : EtOAc = 10 : 1). **^{1}H NMR (400 MHz, DMSO-d_6)** δ 9.80 (s, 1H), 8.51 (brs, 1H), 7.66 (d, *J* = 8.1 Hz, 1H), 6.79 (d, *J* = 1.9 Hz, 1H), 6.66 (dd, *J* = 8.2, 1.9 Hz, 1H), 4.06 (q, *J* = 7.1 Hz, 2H), 3.53 (s, 2H), 1.23 (s, 9H), 1.17 (t, *J* = 7.1 Hz, 3H); **^{13}C NMR (101 MHz, DMSO-d_6)** δ 176.3, 171.13, 147.7, 130.7, 125.2, 121.8, 119.9, 116.2, 60.2, 40.0 , 27.3, 14.1; **ATR-FTIR (cm^{-1})** 3314, 1731, 1650, 1524, 1264, 1029, 875, 797 cm^{-1}; **HRMS m/z (ESI)** called for $C_{15}H_{22}NO_4^+$ $(M + H)^+$ 280.1543, found 280.1545.

***N*-(4-Cyano-2-hydroxyphenyl)pivalamide (17c)**

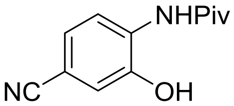

17c

The compound **17c** was performed according to the slightly modified GP-A with *N*-(4-cyanophenyl)pivalamide (40.4 mg, 0.2 mmol), BBr_3 (99.9%, 500.0 mg, 2.0 mmol, 10.0 equiv) in dry DCM (0.1 mL) at room temperature under Ar for 1 hour. The solvent was removed under vaccum directly, diluted with 0.5 mL THF, $NaBO_3{\cdot}4H_2O$ (92.3 mg, 0.6 mmol) and 0.5 mL H_2O were sequentially added to the reaction mixture and stirred at room temperature for another 30 minutes. After that, the excess water was removed by filtration with $MgSO_4$ and then washed with EtOAc (10.0 mL × 3). The filtrate was collected and the solvent removed in vacuum directly. The crude product was purified by flash column chromatography on silica gel (PE : EtOAc = 5 : 1) to afford 29.1 mg (66 %) of **17c** as a white solid. **^{1}H NMR (400 MHz, DMSO-d_6)** δ 10.88 (s, 1H), 8.58 (brs, 1H), 8.13 (d, *J* = 8.4 Hz, 1H), 7.27 (dd, *J* = 8.3, 1.9 Hz, 1H), 7.19 (d, *J* = 1.9 Hz, 1H), 1.24 (s, 9H); **^{13}C NMR (101 MHz, DMSO-d_6)** δ 177.0, 147.6, 131.9, 124.5, 121.4, 119.5, 117.7, 40.0, 27.6; **ATR-FTIR (cm^{-1})** 3369, 1696, 1137, 964, 710 cm^{-1}; **HRMS m/z (ESI)** called for $C_{12}H_{14}N_2NaO_2^+$ $(M + Na)^+$ 241.0947, found 241.0949.

***N*-(4-((*Tert*-butyldimethylsilyl)oxy)-2-hydroxyphenyl)pivalamide (18c)**

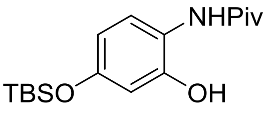

18c

According to the GP-A, **18c** was obtained as a white solid (39.9 mg, 62%), purified by flash column chromatography (PE : EtOAc = 10 : 1). **^{1}H NMR (400 MHz, DMSO-d_6)** δ 9.69 (s, 1H), 8.46 (brs, 1H), 7.47 (d, *J* = 8.7 Hz, 1H), 6.39 (d, *J* = 2.7 Hz, 1H), 6.28 (dd, *J* = 8.6, 2.7 Hz, 1H), 1.21 (s, 9H), 0.93 (s, 9H), 0.16 (s, 6H); **^{13}C NMR (101 MHz, DMSO-d_6)** δ 176.3, 152.2, 149.4, 123.6, 120.4, 110.2, 107.5, 27.3, 25.6, 17.9, -4.6; **ATR-FTIR (cm^{-1})** 2966, 1701, 1589, 1421, 895, 778 cm^{-1}; **HRMS m/z (ESI)** called for $C_{17}H_{30}NO_3Si^+$ $(M + H)^+$ 324.1989, found 324.1988.

***N*-(2-Hydroxy-4-(methylthio)phenyl)pivalamide (19c)**

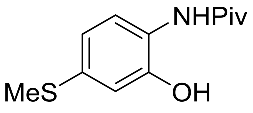

19c

The compound **19c** was performed according to the slightly modified GP-A with *N*-(4-(methylthio)phenyl)pivalamide (44.6 mg, 0.2 mmol), BBr_3 (99.9%, 500.0 mg, 2.0 mmol, 10.0 equiv) in dry DCM (0.1 mL) at room temperature under Ar for 1 hour. The solvent was removed under vaccum directly, diluted with 0.5 mL THF, $NaBO_3{\cdot}4H_2O$ (92.3 mg, 0.6 mmol) and 0.5 mL H_2O were sequentially added to the reaction mixture and stirred at room temperature for another 30 minutes. After that, the excess water was removed by filtration with $MgSO_4$ and then washed with EtOAc (10.0 mL × 3). The filtrate was collected and the solvent removed in vacuum directly. The crude product was purified by flash column chromatography on silica gel (PE : EtOAc = 5 : 1) to afford 29.1 mg (63 %) of **19c** as a white solid. **^{1}H NMR (400 MHz, Chloroform-*d*)** δ 9.05 (s, 1H), 7.59 (brs, 1H), 6.93 (d, *J* = 8.3 Hz, 1H), 6.89 (d, *J* = 2.1 Hz, 1H), 6.75 (dd, *J* = 8.3, 2.2 Hz, 1H), 2.44 (s, 3H), 1.34 (s, 9H); **^{13}C NMR (101 MHz, Chloroform-*d*)** δ 179.0, 149.2, 137.3, 122.9, 122.4, 118.7, 117.3, 39.4, 27.7, 15.9; **ATR-FTIR (cm^{-1})** 3422, 1632, 1407,1119, 794 cm^{-1}; **HRMS m/z (ESI)** called for $C_{12}H_{18}NO_2S^+$ $(M + H)^+$ 240.1053, found 240.1049.

***N*-(2,4-dihydroxyphenyl)pivalamide (20c)**

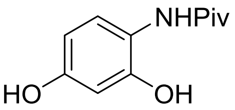

20c

The compound **20c** was prepared according to the GP-A with starting materials *N*-(4-methoxyphenyl)pivalamide (41.4 mg, 0.2 mmol) and BBr_3 (0.5 ml, 0.5mmol). **20c** was obtained as a yellow oil (33.3 mg, 81%), purified by flash column chromatography (PE : EtOAc = 10 : 1). **^{1}H NMR (400 MHz, DMSO-*d*$_6$)** δ 9.47 (s, 1H), 9.09 (s, 1H), 8.45 (brs, 1H), 7.32 (d, *J* = 8.6 Hz, 1H), 6.32 (d, *J* = 2.6 Hz, 1H), 6.18 (dd, *J* = 8.6, 2.7 Hz, 1H), 1.21 (s, 9H); **^{13}C NMR (101 MHz, DMSO-*d*$_6$)** δ 176.3, 154.9, 149.7, 124.0, 118.1, 105.8, 103.1, 38.8, 27.4; **ATR-FTIR (cm^{-1})** 3434, 1660, 1051, 1023, 1003, 821, 758 cm^{-1}; **HRMS m/z (ESI)** called for $C_{11}H_{16}NO_3^+$ $(M + H)^+$ 210.1125, found 210.1122.

(*E*)-*N*-(2-Hydroxy-4-(phenyldiazenyl)phenyl)pivalamide (21c)

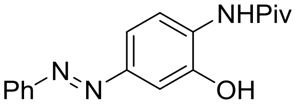

21c

The compound **21c** was performed according to the slightly modified GP-A with *(E)-N*-(4-(phenyldiazenyl)phenyl) pivalamide (56.2 mg, 0.2 mmol), BBr_3 (99.9%, 500.0 mg, 2.0 mmol, 10.0 equiv) in dry DCM (0.1 mL) at room temperature under Ar for 1 hour. The solvent was removed under vaccum directly, diluted with 0.5 mL THF, $NaBO_3\cdot 4H_2O$ (92.3 mg, 0.6 mmol) and 0.5 mL H_2O were sequentially added to the reaction mixture and stirred at room temperature for another 30 minutes. After that, the excess water was removed by filtration with $MgSO_4$ and then washed with EtOAc (10.0 mL × 3). The filtrate was collected and the solvent removed in vacuum directly. The crude product was purified by flash column chromatography on silica gel (PE : EtOAc = 5 : 1) to afford 19.1 mg (33 %) of **21c** as a yellow solid. **^{1}H NMR (400 MHz, Chloroform-*d*)** δ 8.65 (brs, 1H), 7.90 – 7.87 (m, 2H), 7.86 (brs, 1H), 7.57 (d, *J* = 2.1 Hz, 1H), 7.55 – 7.44 (m, 5H), 1.38 (s, 9H); **^{13}C NMR (101 MHz, Chloroform-*d*)** δ 178.8, 152.5, 150.9, 148.5, 130.9, 129.1, 128.4, 122.8, 121.8, 116.9, 111.9, 39.8, 27.7; **ATR-FTIR (cm^{-1})** 3422, 1658,1417, 1197, 895 cm^{-1}; **HRMS m/z (ESI)** called for $C_{17}H_{20}N_3O_2^+$ $(M + H)^+$ 298.1550, found 298.1550.

N-(2-Hydroxyphenyl)-N-methylpivalamide (22c)

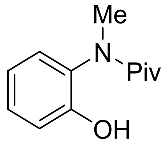

22c

According to the GP-A, **22c** was obtained as a white solid (32.7 mg, 79%), purified by flash column chromatography (PE : EtOAc = 10 : 1). **^{1}H NMR (400 MHz, DMSO-d_6)** δ 9.77 (brs, 1H), 7.21 – 7.12 (m, 2H), 6.91 (dd, J = 8.1, 1.4 Hz, 1H), 6.82 (td, J = 7.5, 1.4 Hz, 1H), 2.98 (s, 3H), 0.96 (s, 9H); **^{13}C NMR (101 MHz, DMSO-d_6)** δ 176.7, 153.6, 131.8, 130.1, 129.1, 119.0, 116.4, 39.8, 38.9, 28.6; **ATR-FTIR (cm^{-1})** 3428, 1630, 1023, 1003, 821, 759 cm^{-1}; **HRMS m/z (ESI)** called for $C_{12}H_{18}NO_2^+$ $(M + H)^+$ 208.1332, found 208.1330.

1-(8-Hydroxy-3,4-dihydroquinolin-1(2*H*)-yl)-2,2-dimethylpropan-1-one (23c)

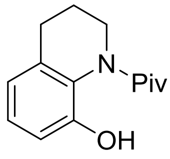

23c

According to the GP-A, **23c** was obtained as a white solid (44.2 mg, 95%), purified by flash column chromatography (PE : EtOAc = 10 : 1). **^{1}H NMR (400 MHz, Chloroform-*d*)** δ 6.86 – 6.81 (m, 1H), 6.79 – 6.74 (m, 1H), 6.58 (t, J = 7.7 Hz, 1H), 3.76 (brs, 1H), 3.34 – 3.29 (m, 2H), 2.79 (t, J = 6.4 Hz, 2H), 1.97 – 1.91 (m, 2H), 1.39 (s, 9H); **^{13}C NMR (101 MHz, Chloroform-*d*)** δ 176.6, 137.1, 136.6, 126.5, 123.3, 119.4, 115.9, 41.6, 39.3, 27.3, 26.8, 21.7; **ATR-FTIR (cm^{-1})** 2931, 1748, 1615, 1501, 1264, 727, 703 cm^{-1}; **HRMS m/z (ESI)** called for $C_{14}H_{20}NO_2^+$ $(M + H)^+$ 234.1489, found 234.1490.

1-(7-Hydroxyindolin-1-yl)-2,2-dimethylpropan-1-one (24c)

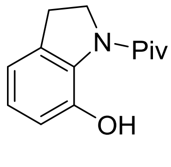

24c

According to the GP-A, **24c** was obtained as a white solid (41.2 mg, 94%), purified by flash column chromatography (PE : EtOAc = 10 : 1). **^{1}H NMR (400 MHz, Chloroform-*d*)** δ 10.21 (s, 1H), 7.06 (t, J = 8.1, 7.3 Hz, 1H), 6.86 – 6.83 (m, 1H), 6.77 – 6.74 (m, 1H), 4.21 (t, J = 7.7 Hz, 2H), 3.07 (t, J = 7.7 Hz, 2H), 1.41 (s, 9H); **^{13}C NMR (101 MHz, Chloroform-*d*)** δ 177.7, 147.2, 134.02 , 130.7, 127.3, 118.0, 115.6, 51.0, 39.9, 30.0, 28.0; **ATR-FTIR (cm^{-1})** 2970, 1621, 1474, 1261, 906, 777 cm^{-1}; **HRMS m/z (ESI)** called for $C_{13}H_{18}NO_2^+$ $(M + H)^+$ 220.1332, found 220.1330.

***N*-(3-Hydroxythiophen-2-yl)pivalamide (25c)**

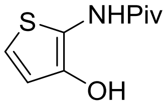

25c

According to the GP-A, **25c** was obtained as a white solid (23.2 mg, 51%), purified by flash column chromatography (PE : EtOAc = 10 : 1). **^{1}H NMR (400 MHz, DMSO-*d*$_6$)** δ 9.09 (brs, 1H), 9.07 (s, 1H), 6.88 (d, *J* = 5.8 Hz, 1H), 6.55 (d, *J* = 5.8 Hz, 1H), 1.21 (s, 9H); **^{13}C NMR (101 MHz, DMSO-*d*$_6$)** δ 174.8, 142.1, 117.9, 116.0, 114.7, 38.0, 26.7; **ATR-FTIR (cm^{-1})** 3433, 1659, 1264, 821, 780 cm^{-1}; **HRMS m/z (ESI)** called for $C_9H_{14}NO_2S^+$ $(M + H)^+$ 200.0740, found 200.0741.

***N*-(3-Hydroxy-9*H*-fluoren-2-yl)pivalamide (26c)**

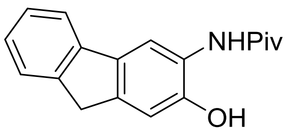

26c

According to the GP-A, **26c** was obtained as a white solid (47.7 mg, 85%), purified by flash column chromatography (PE : EtOAc = 10 : 1). **^{1}H NMR (400 MHz, DMSO-*d*$_6$)** δ 9.94 (s, 1H), 8.59 (brs, 1H), 8.08 (s, 1H), 7.72 (d, *J* = 7.5 Hz, 1H), 7.52 (d, *J* = 7.4 Hz, 1H), 7.36 – 7.31 (m, 2H), 7.25 (td, *J* = 7.4, 1.1 Hz, 1H), 3.79 (s, 2H), 1.26 (s, 9H); **^{13}C NMR (101 MHz, DMSO-*d*$_6$)** δ 176.2, 146.8, 143.6, 141.2, 137.0, 133.7, 126.6, 126.0, 125.9, 124.9, 119.3, 117.9, 106.5, 35.9, 27.3; **ATR-FTIR (cm^{-1})** 2931, 1748, 1615, 1501, 1264, 727, 703 cm^{-1}; **HRMS m/z (ESI)** called for $C_{18}H_{20}NO_2^+$ $(M + H)^+$ 282.1489, found 282.1485.

***N*-(5-(Dibenzo[*b,d*]thiophen-2-yl)-2-hydroxyphenyl)pivalamide (27c)**

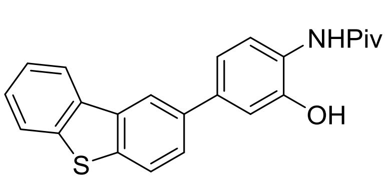

27c

According to the GP-A, **27c** was obtained as a white solid (61.5 mg, 78%), purified by flash column chromatography (PE : EtOAc = 10 : 1). **^{1}H NMR (400 MHz, DMSO-*d*$_6$)** δ 10.05 (brs, 1H), 8.69 (s, 1H), 8.54 (d, *J* = 1.7 Hz, 1H), 8.49 (ddd, *J* = 5.2, 2.9, 0.7 Hz, 1H), 8.26 (d, *J* = 2.3 Hz, 1H), 8.10 – 7.99 (m, 2H), 7.71 (dd, *J* = 8.4, 1.9 Hz, 1H), 7.56 – 7.50 (m, 2H), 7.47 (dd, *J* = 8.3, 2.3 Hz, 1H), 7.03 (d, *J* = 8.3 Hz, 1H), 1.28 (s, 9H); **^{13}C NMR (101 MHz,**

DMSO-*d*$_6$) δ 175.7, 146.7, 138.0, 136.2, 135.8, 134.7, 134.1, 130.1, 126.2, 125.9, 124.6, 123.7, 122.3, 122.1, 121.3, 119.7, 118.2, 115.0, 26.3; **ATR-FTIR (cm^{-1})** 3427, 1671, 1118, 821, 761 cm^{-1}; **HRMS m/z (ESI)** called for $C_{23}H_{22}NO_2S^+$ (M + H)$^+$ 376.1366, found 376.1361.

N-(2-Hydroxypyren-1-yl)pivalamide (28c)

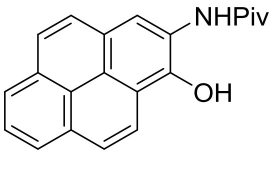

28c

According to the GP-A, **28c** was obtained as a white solid (57.7 mg, 91%), purified by flash column chromatography (PE : EtOAc = 10 : 1). **^{1}H NMR (400 MHz, DMSO-*d*$_6$)** δ 9.96 (brs, 1H), 9.28 (brs, 1H), 8.24 – 8.19 (m, 2H), 8.14 – 8.02 (m, 3H), 7.98 – 7.91 (m, 2H), 7.80 (s, 1H), 1.40 (s, 9H); **^{13}C NMR (101 MHz, DMSO-*d*$_6$)** δ 177.7, 151.9, 130.4, 129.5, 129.2, 129.1, 127.4, 127.3, 126.4, 125.3, 125.1, 124.9, 123.9, 122.7, 120.3, 118.5, 112.2, 38.9, 27.7; **ATR-FTIR (cm^{-1})** 2974, 1660, 1264, 1025, 820, 729, 701 cm^{-1}; **HRMS m/z (ESI)** called for $C_{21}H_{20}NO_2^+$ (M + H)$^+$ 318.1489, found 318.1485.

Genera proceduce B

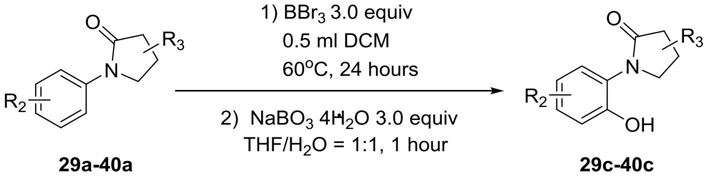

Flame-dried 25 mL Schlenk tube was flushed with argon and charged with *N*-arylpyrrolidinone substrates **29-40a** (0.2 mmol, 1.0 equiv) and dry DCM (0.5 mL, 0.4 M). A solution of BBr_3 (1.0 M in DCM, 0.6 mL, 3.0 equiv) was added slowly under argon atmosphere. The mixture was stirred at 60°C for 24 hours. After stirring, the solvent was removed under vacuum directly. 0.5 mL THF, $NaBO_3 \cdot 4H_2O$ (92.3 mg, 0.6 mmol) and 0.5 mL 1M K_2CO_3 aq were sequentially added to the reaction mixture and stirred at room temperature for another 1hour (monitored by TLC). After that, the excess water was removed by filtration with $MgSO_4$ and then washed with EtOAc

(10.0 mL × 3). The filtrate was collected and the solvent removed in vacuum and purified by flash column chromatography.

1-(2-Hydroxyphenyl)pyrrolidin-2-one (29c) [5]

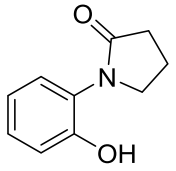

29c

According to the GP-B, **29c** was obtained as a white solid (29.1 mg, 80%), purified by flash column chromatography (PE : EtOAc = 10 : 1). **^{1}H NMR (400 MHz, DMSO-*d*$_6$)** δ 9.50 (s, 1H), 7.12 (t, *J* = 7.6 Hz, 2H), 6.93 – 6.88 (m, 1H), 6.81 (td, *J* = 7.6, 1.4 Hz, 1H), 3.69 (t, *J* = 7.0 Hz, 2H), 2.39 (dd, *J* = 8.6, 7.5 Hz, 2H), 2.08 (m, 2H); **^{13}C NMR (101 MHz, DMSO-*d*$_6$)** δ 173.8, 152.0, 127.5, 127.3, 125.8, 118.6, 116.4, 48.7, 30.3, 17.9; **ATR-FTIR (cm^{-1})** 3434, 1686, 1275, 1005, 820, 757 cm^{-1}; **HRMS m/z (ESI)** called for $C_{10}H_{12}NO_2^+$ $(M + H)^+$ 178.0863, found 178.0862.

1-(2-Hydroxy-5-methylphenyl)pyrrolidin-2-one (30c) [5]

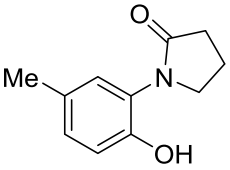

30c

According to the GP-B, **30c** was obtained as a white solid (35.5 mg, 93%), purified by flash column chromatography (PE : EtOAc = 10 : 1). **^{1}H NMR (400 MHz, DMSO-*d*$_6$)** δ 9.22 (s, 1H), 6.92 (d, *J* = 8.9 Hz, 2H), 6.78 (d, *J* = 8.0 Hz, 1H), 3.68 (t, *J* = 7.0 Hz, 2H), 2.38 (t, *J* = 8.1 Hz, 2H), 2.19 (s, 3H), 2.07 (m, 2H). **^{13}C NMR (101 MHz, DMSO-*d*$_6$)** δ 173.8, 149.5, 127.8, 127.3, 125.4, 116.3, 48.8, 30.4, 19.5, 17.9; **ATR-FTIR (cm^{-1})** 3427, 1672, 1023, 1004, 759, 729 cm^{-1}; **HRMS m/z (ESI)** called for $C_{11}H_{14}NO_2^+$ $(M + H)^+$ 192.1019, found 192.1019.

1-(4-Ethyl-2-hydroxyphenyl)pyrrolidin-2-one (31c)

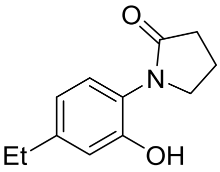

31c

According to the GP-B, **31c** was obtained as a white solid (40.2 mg, 98%), purified by flash column chromatography (PE : EtOAc = 10 : 1). **^{1}H NMR (400 MHz, DMSO-*d*$_6$)** δ 9.30 (s, 1H), 6.96 (d, *J* = 8.0 Hz, 1H), 6.68 (d, *J* = 1.9 Hz, 1H), 6.60 (dd, *J* = 8.0, 1.9 Hz, 1H), 3.61 (t, *J* = 7.0 Hz, 2H), 2.46 (q, *J* = 7.5 Hz, 2H), 2.32 (t, *J* =

8.1 Hz, 2H), 2.01 (p, *J* = 7.4 Hz, 2H), 1.09 (t, *J* = 7.6 Hz, 3H); **^{13}C NMR (101 MHz, DMSO-*d*$_6$)** δ 173.8, 151.7, 143.3, 126.9, 123.4, 118.1, 115.6, 48.8, 30.3, 27.3, 17.9, 15.0; **ATR-FTIR (cm^{-1})** 3427, 1675, 1051, 1025, 823, 761 cm^{-1}; **HRMS m/z (ESI)** called for $C_{12}H_{16}NO_2^+$ (M + H)$^+$ 206.1176, found 206.1174.

1-(5-Fluoro-2-hydroxyphenyl)pyrrolidin-2-one (32c) [6]

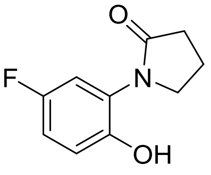

32c

According to the GP-B, **32c** was obtained as a white solid (34.7 mg, 89%), purified by flash column chromatography (PE : EtOAc = 10 : 1). **^{1}H NMR (400 MHz, DMSO-*d*$_6$)** δ 9.50 (s, 1H), 7.04 (dd, *J* = 9.7, 3.1 Hz, 1H), 6.97 (ddd, *J* = 8.9, 8.1, 3.1 Hz, 1H), 6.88 (dd, *J* = 9.0, 5.5 Hz, 1H), 3.71 (t, *J* = 7.0 Hz, 2H), 2.39 (t, *J* = 8.0 Hz, 2H), 2.13 – 2.03 (m, 2H); **^{13}C NMR (101 MHz, DMSO-*d*$_6$)** δ 173.9, 154.3 (d, *J* = 234.8 Hz), 148.3, 126.2 (d, *J* = 10.1 Hz), 116.9 (d, *J* = 8.9 Hz), 113.8, 113.5 (d, *J* = 3.0 Hz), 48.5, 30.2, 17.9; **^{19}F NMR (376 MHz, DMSO-*d*$_6$)** δ -124.97; **ATR-FTIR (cm^{-1})** 3424, 1687, 1252, 1023, 1003, 821, 759 cm^{-1}; **HRMS m/z (ESI)** called for $C_{12}H_{16}NO_2^+$ (M + H)$^+$ 196.0768, found 196.0772.

1-(5-Chloro-2-hydroxyphenyl)pyrrolidin-2-one (33c) [5]

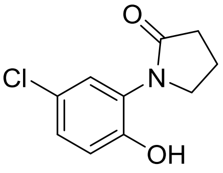

33c

According to the GP-B, **33c** was obtained as a white solid (33.3 mg, 79%), purified by flash column chromatography (PE : EtOAc = 10 : 1), . **^{1}H NMR (400 MHz, DMSO-*d*$_6$)** δ 9.86 (brs, 1H), 7.22 (d, *J* = 2.6 Hz, 1H), 7.17 (dd, *J* = 8.7, 2.7 Hz, 1H), 6.91 (d, *J* = 8.7 Hz, 1H), 3.69 (t, *J* = 7.0 Hz, 2H), 2.38 (t, *J* = 8.0 Hz, 2H), 2.07 (m, 2H); **^{13}C NMR (101 MHz, DMSO-*d*$_6$)** δ 173.9, 151.1, 127.1, 127.1, 126.8, 121.4, 117.6, 48.4, 30.1, 17.9; **ATR-FTIR (cm^{-1})** 3434, 1686, 1052, 1005, 820, 728 cm^{-1}; **HRMS m/z (ESI)** called for $C_{10}H_{11}ClNO_2^+$ (M + H)$^+$ 212.0473, found 212.0473.

1-(4-Hydroxy-[1,1'-biphenyl]-3-yl)pyrrolidin-2-one (34c)

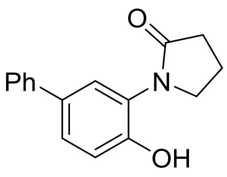

34c

According to the GP-B, **34c** was obtained as a white solid (41.5 mg, 82%), purified by flash column chromatography (PE : EtOAc = 10 : 1). **^{1}H NMR (400 MHz, Chloroform-*d*)** δ 8.61 (brs, 1H), 7.55 – 7.49 (m, 2H), 7.47 – 7.38 (m, 3H), 7.36 – 7.30 (m, 1H), 7.25 (s, 1H), 7.13 (d, *J* = 8.4 Hz, 1H), 4.04 (t, *J* = 7.0 Hz, 2H), 2.72 (t, *J* = 8.0 Hz, 2H), 2.39 – 2.25 (m, 2H); **^{13}C NMR (101 MHz, Chloroform-*d*)** δ 176.3, 149.7, 140.4, 134.0, 128.8, 127.9, 127.0, 126.8, 126.5, 121.1, 119.9, 51.0, 32.3, 19.6; **ATR-FTIR (cm^{-1})** 3055, 1658, 1264, 733, 701 cm^{-1}; **HRMS m/z (ESI)** called for $C_{16}H_{16}NO_2^+$ $(M + H)^+$ 254.1176, found 254.1175.

1-(2-Hydroxyphenyl)-5-methylpyrrolidin-2-one (35c) [7]

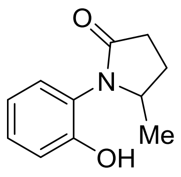

35c

According to the GP-B, **35c** was obtained as a white solid (19.9 mg, 45%), purified by flash column chromatography (PE : EtOAc = 10 : 1). **^{1}H NMR (500 MHz, DMSO-d_6)** δ 9.46 (s, 1H), 7.13 (td, *J* = 7.9, 1.7 Hz, 1H), 7.03 (dd, *J* = 7.8, 1.6 Hz, 1H), 6.89 (dd, *J* = 8.1, 1.3 Hz, 1H), 6.81 (td, *J* = 7.6, 1.3 Hz, 1H), 4.16 – 4.09 (m, 1H), 2.40 – 2.35 (m, 2H), 2.33 – 2.25 (m, 1H), 1.69 – 1.60 (m, 1H), 0.98 (d, *J* = 6.3 Hz, 3H); **^{13}C NMR (126 MHz, DMSO-d_6)** δ 173.4, 152.8, 129.2, 127.7, 124.1, 118.5, 116.0, 54.3, 29.9, 26.5, 19.5; **ATR-FTIR (cm^{-1})** 3431, 1664, 1051, 1003, 821, 759 cm^{-1}; **HRMS m/z (ESI)** called for $C_{11}H_{14}NO_2^+$ $(M + H)^+$ 192.1019, found 192.1017.

2-(2-Hydroxyphenyl)-2-azaspiro[4.5]decan-3-one (36c)

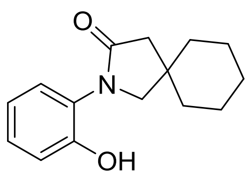

36c

According to the GP-B, **36c** was obtained as a white solid (35.7 mg, 73%), purified by flash column chromatography (PE : EtOAc = 10 : 1). **^{1}H NMR (400 MHz, DMSO-d_6)** δ 9.52 (s, 1H), 7.15 – 7.07 (m, 2H), 6.92 – 6.87 (m, 1H), 6.80 (m, 1H), 3.49 (s, 2H), 2.28 (s, 2H), 1.58 – 1.35 (m, 10H); **^{13}C NMR (101 MHz, DMSO-d_6)** δ 172.5, 151.9, 127.3, 127.1, 125.7, 118.6, 116.3, 59.5, 43.1, 36.3, 35.4, 24.9, 21.9; **ATR-FTIR (cm^{-1})** 3427, 1685, 1265, 1024, 1004, 822, 759 cm^{-1}; **HRMS**

m/z (ESI) called for $C_{15}H_{20}NO_2^+$ $(M + H)^+$ 246.1489, found 246.1489.

1-(2-Hydroxyphenyl)piperidin-2-one (37c) [8]

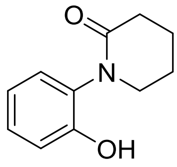

37c

According to the GP-B, **37c** was obtained as a white solid (27.5 mg, 72%), purified by flash column chromatography (PE : EtOAc = 10 : 1). **^{1}H NMR (400 MHz, DMSO-*d*$_6$)** δ 9.31 (s, 1H), 7.12 – 7.08 (m, 1H), 7.04 (dd, *J* = 7.8, 1.7 Hz, 1H), 6.81 – 6.76 (dd, *J* = 8.2, 1.4 Hz, 1H), 6.78 (m, 1H), 3.48 – 3.38 (m, 2H), 2.34 (t, *J* = 6.1 Hz, 2H), 1.86 – 1.80 (m, 4H); **^{13}C NMR (101 MHz, DMSO-*d*$_6$)** δ 168.2, 152.2, 130.1, 128.5, 127.5, 118.7, 116.1, 49.7, 31.9, 22.4, 20.5; **ATR-FTIR (cm^{-1})** 3423, 1642, 1023, 1003, 822, 759 cm^{-1}; **HRMS m/z (ESI)** called for $C_{11}H_{14}NO_2^+$ $(M + H)^+$ 192.1019, found 192.1019.

1-(2-Hydroxyphenyl)azepan-2-one (38c) [8]

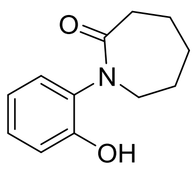

38c

According to the GP-B, **38c** was obtained as a white solid (26.7 mg, 65%), purified by flash column chromatography (PE : EtOAc = 10 : 1). **^{1}H NMR (500 MHz, DMSO-*d*$_6$)** δ 9.35 (s, 1H), 7.07 (td, *J* = 7.7, 1.7 Hz, 1H), 7.00 (dd, *J* = 7.8, 1.7 Hz, 1H), 6.87 (dd, *J* = 8.1, 1.5 Hz, 1H), 6.76 (td, *J* = 7.6, 1.5 Hz, 1H), 3.58 – 3.48 (m, 2H), 2.55 (d, *J* = 8.0 Hz, 2H), 1.73 – 1.68 (m, 6H); **^{13}C NMR (126 MHz, DMSO-*d*$_6$)** δ 174.2, 151.9, 131.5, 128.4, 127.2, 118.5, 116.0, 51.4, 36.5, 28.9, 27.5, 22.6; **ATR-FTIR (cm^{-1})** 3435, 1650, 1024, 1005, 820, 758 cm^{-1}; **HRMS m/z (ESI)** called for $C_{12}H_{16}NO_2^+$ (M + H)$^+$ 206.1176, found 206.1175.

3-(2-Hydroxyphenyl)oxazolidin-2-one (39c) [9]

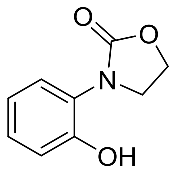

39c

According to the GP-B, **39c** was obtained as a white solid (30.7 mg, 86%), purified by flash column chromatography (PE : EtOAc = 10 : 1). **^{1}H NMR (400 MHz, DMSO-*d*$_6$)** δ 9.79 (s, 1H), 7.22 (dd, *J* = 7.8, 1.6 Hz, 1H), 7.15 (ddd, *J* = 8.0, 7.4, 1.7 Hz, 1H), 6.92 (dd, *J* = 8.2, 1.3 Hz,

1H), 6.82 (td, J = 7.6, 1.4 Hz, 1H), 4.46 – 4.38 (m, 2H), 3.93 – 3.84 (m, 2H); **^{13}C NMR (101 MHz, DMSO-d_6)** δ 156.2, 152.6, 128.0, 127.9, 124.3, 118.7, 116.1, 61.7, 46.0; **ATR-FTIR (cm^{-1})** 3428, 1747, 1024, 1005, 821, 729 cm^{-1}; **HRMS m/z (ESI)** called for $C_9H_{10}NO_3^+$ $(M + H)^+$ 180.0655, found 180.0653.

1-(3-Hydroxythiophen-2-yl)pyrrolidin-2-one (40c)

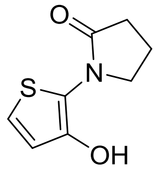

40c

According to the GP-B, **40c** was obtained as a white solid (33.7 mg, 81%), purified by flash column chromatography (PE : EtOAc = 10 : 1). **^{1}H NMR (400 MHz, DMSO-d_6)** δ 7.09 (d, J = 5.8 Hz, 1H), 6.59 (d, J = 5.8 Hz, 1H), 3.73 (dd, J = 7.5, 6.7 Hz, 2H), 2.37 (t, J = 8.1 Hz, 2H), 2.11 – 2.01 (m, 2H); **^{13}C NMR (101 MHz, DMSO-d_6)** δ 173.7, 145.8, 118.9, 118.8, 114.2, 49.1, 29.6, 17.5; **ATR-FTIR (cm^{-1})** 3412, 1682, 1024, 1003, 822, 729 cm^{-1}; **HRMS m/z (ESI)** called for $C_8H_9NNaO_2S^+$ $(M + Na)^+$ 206.0246, found 206.0242.

***N,N'*-(3,3'-Dihydroxy-[1,1'-biphenyl]-4,4'-diyl)bis(2,2-dimethylpropanamide) (41c)**

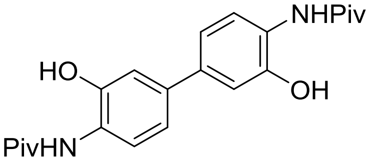

41c

The compound **41c** was performed according to the slightly modified GP-A with *N,N'*-([1,1'-biphenyl]-4,4'-diyl)bis(2,2-dimethylpropanamide) (70.4 mg, 0.2 mmol), BBr_3 (1.0 M in DCM, 0.4 mL, 2.0 equiv) in dry DCM (0.5 mL) at room temperature under Ar for 1 hour. The solvent was removed under vaccum directly, diluted with 0.5 mL THF, $NaBO_3 \cdot 4H_2O$ (229.5 mg, 1.5 mmol) and 0.5 mL H_2O were sequentially added to the reaction mixture and stirred at room temperature for another 1 hour. After that, the excess water was removed by filtration with $MgSO_4$ and then washed with EtOAc (10.0 mL × 3). The filtrate was collected and the solvent removed in vacuum directly. The crude product was purified by flash column chromatography on silica gel (PE : EtOAc = 5 : 1) to afford 52.7 mg (74 %) of **41c** as a white solid. **^{1}H NMR (500 MHz, DMSO-d_6)**

δ 9.98 (brs, 2H), 8.55 (s, 2H), 7.84 (d, *J* = 8.3 Hz, 2H), 7.08 (d, *J* = 2.2 Hz, 2H), 7.02 (dt, *J* = 8.4 Hz, 2H), 1.25 (s, 18H); **^{13}C NMR (126 MHz, DMSO-*d*$_6$)** δ 175.9, 144.9, 136.5, 124.9, 124.7, 122.7, 115.7, 38.7, 26.8, 18.2; **ATR-FTIR (cm^{-1})** 3395, 1656, 1050, 823, 702 cm^{-1}; **HRMS m/z (ESI)** called for $C_{22}H_{29}N_2O_4^+$ (M + H)$^+$ 385.2122, found 385.2123.

_N,N'_-(3,3'-Dihydroxy-5,5'-dimethyl-[1,1'-biphenyl]-4,4'-diyl)bis(2,2-dimethylpropanamide) (42c)

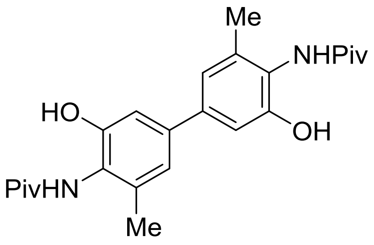

42c

The compound **42c** was performed according to the slightly modified GP-A with *N,N'*-(3-hydroxy-3'-methyl-[1,1'-biphenyl]-4,4'-diyl)bis(2,2-dimethylpropanamide) (76.4 mg, 0.2 mmol), BBr_3 (1.0 M in DCM, 0.4 mL, 2.0 equiv) in dry DCM (0.5 mL) at room temperature under Ar for 1 hour. The solvent was removed under vaccum directly, diluted with 0.5 mL THF, $NaBO_3 \cdot 4H_2O$ (229.5 mg, 1.5 mmol) and 0.5 mL H_2O were sequentially added to the reaction mixture and stirred at room temperature for another 1 hour. After that, the excess water was removed by filtration with $MgSO_4$ and then washed with EtOAc (10.0 mL × 3). The filtrate was collected and the solvent removed in vacuum directly. The crude product was purified by flash column chromatography on silica gel (PE : EtOAc = 5 : 1) to afford 50.1 mg (60 %) of **42c** as a white solid. **^{1}H NMR (500 MHz, DMSO-*d*$_6$)** δ 9.17 (brs, 2H), 8.57 (brs, 2H), 6.91 (s, 4H), 2.14 (s, 6H), 1.26 (s, 18H); **^{13}C NMR (126 MHz, DMSO-*d*$_6$)** δ 176.2, 152.8, 138.1, 136.5, 123.3, 118.1, 111.0, 38.2, 27.0, 17.5; **ATR-FTIR (cm^{-1})** 3433, 1622,1266, 1004, 821, 785 cm^{-1}; **HRMS m/z (ESI)** called for $C_{24}H_{33}N_2O_4^+$ (M + H)$^+$ 413.2435, found 413.2435.

_N,N'_-(5,5'-Dihydroxy-2,2'-dimethyl-[1,1'-biphenyl]-4,4'-diyl)bis(2,2-dimethylpropanamide) (43c)

The compound **43c** was performed according to the slightly modified GP-A with

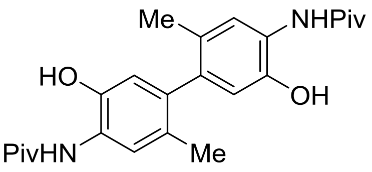

43c

N,N'-(2,2'-dimethyl-[1,1'-biphenyl]-4,4'-diyl)bis(2,2-dimethylpropanamide) (76.0 mg, 0.2 mmol), BBr_3 (1.0 M in DCM, 0.4 mL, 2.0 equiv) in dry DCM (0.5 mL) at room temperature under Ar for 1 hour. The solvent was removed under vaccum directly, diluted with 0.5 mL THF, $NaBO_3{\cdot}4H_2O$ (229.5 mg, 1.5 mmol) and 0.5 mL H_2O were sequentially added to the reaction mixture and stirred at room temperature for another 1 hour. After that, the excess water was removed by filtration with $MgSO_4$ and then washed with EtOAc (10.0 mL × 3). The filtrate was collected and the solvent removed in vacuum directly. The crude product was purified by flash column chromatography on silica gel (PE : EtOAc = 5 : 1) to afford 73.3 mg (89 %) of **43c** as a white solid. **^{1}H NMR (500 MHz, DMSO-*d*$_6$)** δ 9.61 (brs, 2H), 8.56 (s, 2H), 7.67 (s, 2H), 6.55 (s, 2H), 1.90 (s, 6H), 1.25 (s, 18H);**^{13}C NMR (126 MHz, DMSO-*d*$_6$)** δ 175.9, 144.9, 136.5, 124.9, 124.7, 122.7, 115.7, 38.7, 26.8, 18.2; **ATR-FTIR (cm^{-1})** 3427, 1663,1420, 1265, 822, 760 cm^{-1}; **HRMS m/z (ESI)** called for $C_{24}H_{33}N_2O_4^+$ $(M + H)^+$ 413.2435, found 413.2437.

***N,N'*-(Methylenebis(2-hydroxy-4,1-phenylene))bis(2,2-dimethylpropanamide) (44c)**

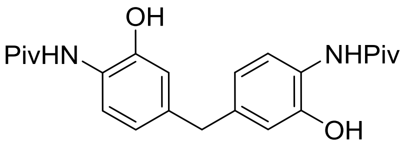

44c

The compound **44c** was performed according to the slightly modified GP-A with *N,N'*-(methylenebis(4,1-phenylene))bis(2,2-dimethylpropanamide) (73.2 mg, 0.2 mmol), BBr_3 (1.0 M in DCM, 0.4 mL, 2.0 equiv) in dry DCM (0.5 mL) at room temperature under Ar for 1 hour. The solvent was removed under vaccum directly, diluted with 0.5 mL THF, $NaBO_3{\cdot}4H_2O$ (229.5 mg, 1.5 mmol) and 0.5 mL H_2O were sequentially added to the reaction mixture and stirred at room temperature for another 1 hour. After that, the excess water was removed by filtration with $MgSO_4$ and then washed with EtOAc (10.0 mL × 3). The filtrate was collected and the solvent removed in vacuum directly. The crude product was purified by flash column chromatography on silica gel (PE : EtOAc = 5 : 1) to

afford 62.1 mg (78 %) of **44c** as a white solid. **^{1}H NMR (400 MHz, DMSO-*d*$_6$)** δ 9.67 (s, 2H), 8.49 (s, 2H), 7.63 (d, *J* = 8.0 Hz, 2H), 6.66 – 6.60 (m, 4H), 3.73 (s, 2H), 1.22 (s, 18H); **^{13}C NMR (101 MHz, DMSO-*d*$_6$)** δ 175.8, 147.3, 137.3, 123.9, 121.4, 118.9, 115.3, 26.8; **ATR-FTIR (cm^{-1})** 3428, 1660, 822, 795 cm^{-1}; **HRMS m/z (ESI)** called for $C_{23}H_{31}N_2O_4^+$ (M + H)$^+$ 399.2278, found 399.2279.

***N,N'*-(Oxybis(2-hydroxy-4,1-phenylene))bis(2,2-dimethylpropanamide) (45c)**

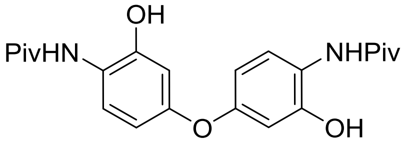

45c

The compound **45c** was performed according to the slightly modified GP-A with *N,N'*-(oxybis(4,1-phenylene))bis(2,2-dimethylpropanamide) (73.6 mg, 0.2 mmol), BBr_3 (1.0 M in DCM, 0.4 mL, 2.0 equiv) in dry DCM (0.5 mL) at room temperature under Ar for 1 hour. The solvent was removed under vaccum directly, diluted with 0.5 mL THF, $NaBO_3{\cdot}4H_2O$ (229.5 mg, 1.5 mmol) and 0.5 mL H_2O were sequentially added to the reaction mixture and stirred at room temperature for another 1 hour. After that, the excess water was removed by filtration with $MgSO_4$ and then washed with EtOAc (10.0 mL × 3). The filtrate was collected and the solvent removed in vacuum directly. The crude product was purified by flash column chromatography on silica gel (PE : EtOAc = 5 : 1) to afford 50.4 mg (63 %) of **45c** as a white solid.**^{1}H NMR (400 MHz, DMSO-*d*$_6$)** δ 9.99 (s, 2H), 8.48 (s, 2H), 7.65 (d, *J* = 8.7 Hz, 2H), 6.50 (d, *J* = 2.6 Hz, 2H), 6.44 (dd, *J* = 8.7, 2.7 Hz, 2H), 1.23 (s, 18H); **^{13}C NMR (101 MHz, DMSO-*d*$_6$)** δ 175.9, 153.3, 149.0, 123.1, 121.5, 108.5, 105.5, 38.5, 26.8;**ATR-FTIR (cm^{-1})** 3424, 1663, 1513, 1050, 822 cm^{-1}; **HRMS m/z (ESI)** called for $C_{22}H_{29}N_2O_5^+$ (M + H)$^+$ 401.2071, found 401.2074.

Genera proceduce C

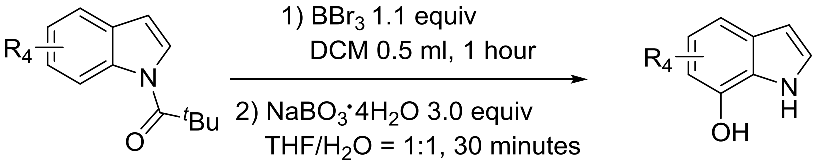

46a-57a **46c-57c**

To a flame-dried 25 mL Schlenk tube was flushed with argon and charged with *N*-pivaloyl indole **46-57a** (0.2 mmol, 1.0 equiv) and dry DCM (0.5 mL, 0.4 M). A solution of BBr_3 (1 M in DCM, 0.22 mL, 1.1 equiv) was added slowly under argon atmosphere. The reaction mixture was stirred at room temperature for 1 hour. After stirring, the solvent was removed under vacuum directly. 0.5 mL THF, $NaBO_3 \cdot 4H_2O$ (92.3 mg, 0.6 mmol) and 0.5 mL 1M K_2CO_3 aq were sequentially added to the reaction mixture and stirred at room temperature for another 30 minutes. The reaction mixture was quenched by the addition of Et_3N (0.1 mL) and stirred for another 30 minutes. Then the reaction mixture was extracted with brine(20.0 ml) EtOAc (30.0 mL) and 1 M HCl (5.0 mL). The organic layer was dried (Na_2SO_4) and evaporated. The crude residue was purified by flash chromatography on silica gel to give the products in good to moderate yields.

1*H*-indol-7-ol (46c) [10]

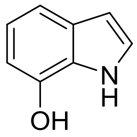

46c

According to the GP-C, **46c** was obtained as a white solid (29.1 mg, 80%), purified by flash column chromatography (PE : EtOAc = 5 : 1). **^{1}H NMR (400 MHz, Chloroform-*d*)** δ 8.39 (brs, 1H), 7.25 (dt, *J* = 8.0, 0.9 Hz, 1H), 7.17 (t, *J* = 2.8 Hz, 1H), 6.94 (t, *J* = 7.8 Hz, 1H), 6.59 – 6.50 (m, 2H), 5.26 (brs, 1H); **^{13}C NMR (101 MHz, Chloroform-*d*)** δ 141.4, 130.1, 125.7, 124.2, 120.1, 113.6, 106.4, 102.9; **ATR-FTIR (cm^{-1})** 3855, 2926, 1655, 1583, 1132, 898, 451 cm^{-1}; **HRMS m/z (ESI)** called for $C_8H_8NO^+$ $(M + H)^+$ 134.0600, found 134.0595.

5-Methyl-1*H*-indol-7-ol (47c)

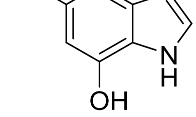

47c

According to the GP-C, **47c** was obtained as a white solid (25.1 mg, 85%), purified by flash column chromatography (PE : EtOAc = 5 : 1). **^{1}H NMR (400 MHz, Chloroform-*d*)** δ 8.32 (brs, 1H), 7.15 (t, *J* = 2.8 Hz, 1H), 7.06 (s, 1H), 6.46 (dd, *J* = 3.1, 2.1 Hz, 1H), 6.41 (s, 1H), 5.27 (brs, 1H), 2.40 (s, 3H); **^{13}C NMR (101 MHz, Chloroform-*d*)** δ 141.0,

130.2, 129.8, 124.3, 124.0, 113.1, 108.2, 102.4, 21.4; **ATR-FTIR (cm^{-1})** 1420, 1360, 1221, 1091, 733, 529 cm^{-1}; **HRMS m/z (ESI)** called for $C_9H_9NNaO^+$ $(M + Na)^+$ 170.0576, found 170.0580.

4-Methyl-1*H*-indol-7-ol (48c)

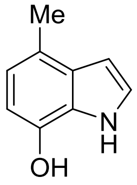

48c

According to the GP-C, **48c** was obtained as a white solid (22.1 mg, 74%), purified by flash column chromatography (PE : EtOAc = 5 : 1). **^{1}H NMR (400 MHz, Chloroform-*d*)** δ 8.42 (brs, 1H), 7.23 – 7.16 (m, 1H), 6.73 (dd, *J* = 7.6, 0.8 Hz, 1H), 6.55 (dd, *J* = 3.1, 2.2 Hz, 1H), 6.50 (d, *J* = 7.6 Hz, 1H), 5.10 (brs, 1H), 2.50 (s, 3H); **^{13}C NMR (101 MHz, Chloroform-*d*)** δ 139.6, 129.7, 125.3, 123.6, 122.8, 119.5, 106.3, 101.6, 18.2; **ATR-FTIR (cm^{-1})** 3053, 1421, 1264, 895, 730, 703 cm^{-1}; **HRMS m/z (ESI)** called for $C_9H_{10}NO^+$ $(M + H)^+$148.0757, found 148.0752.

6-Methyl-1*H*-indol-7-ol (49c)

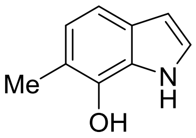

49c

According to the GP-C, **49c** was obtained as a white solid (22.6 mg, 77%), purified by flash column chromatography (PE : EtOAc = 5 : 1). **^{1}H NMR (400 MHz, Chloroform-d)** δ 8.31 (brs, 1H), 7.19 (d, J = 8.0 Hz, 1H), 7.14 (dd, J = 3.1, 2.4 Hz, 1H), 6.89 (d, J = 8.0 Hz, 1H), 6.51 (dd, J = 3.1, 2.1 Hz, 1H), 4.79 (brs, 1H), 2.37 (s, 3H); **^{13}C NMR (101 MHz, Chloroform-*d*)** δ 139.3, 128.4, 126.3, 123.7, 122.7, 113.9, 113.1, 102.7, 14.9; **ATR-FTIR (cm^{-1})** 2973, 1423, 1264, 895, 703 cm^{-1}; **HRMS m/z (ESI)** called for $C_9H_{10}NO^+$ $(M + H)^+$ 148.0757, found 148.0752.

4-Fluoro-1*H*-indol-7-ol (50c)

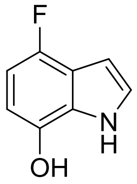

50c

According to the GP-C, **50c** was obtained as a white solid (22.3 mg, 74%), purified by flash column chromatography (PE : EtOAc = 5 : 1). **^{1}H NMR (400 MHz, Chloroform-*d*)** δ 8.48 (brs, 1H), 7.18 (t, *J* = 2.8 Hz,

1H), 6.63 – 6.56 (m, 2H), 6.45 (dd, *J* = 8.2, 3.5 Hz, 1H), 5.10 (brs, 1H). **^{13}C NMR (101 MHz, Chloroform-*d*)** δ 152.29 , 137.65 , 124.02 , 105.77 (d, *J* = 7.8 Hz), 103.68 (d, *J* = 21.3 Hz), 99.33 .**^{19}F NMR (376 MHz, Chloroform-*d*)** δ -131.42; **ATR-FTIR (cm^{-1})** 2993, 1446, 938, 846 cm^{-1}; **HRMS m/z (ESI)** called for $C_8H_6FNNaO^+$ (M + Na)$^+$ 174.0326, found 174.0339.

6-Fluoro-1*H*-indol-7-ol (51c)

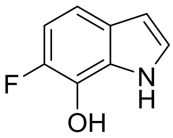

51c

According to the GP-C, **51c** was obtained as a white solid (18.3 mg, 61%), purified by flash column chromatography (PE : EtOAc = 5 : 1). **^{1}H NMR (400 MHz, Chloroform-*d*)** δ 8.38 (brs, 1H), 7.20 (dd, *J* = 3.2, 2.3 Hz, 1H), 7.12 – 7.09 (m, 1H), 6.91 (dd, *J* = 11.0, 8.7 Hz, 1H), 6.51 (dd, *J* = 3.2, 2.2 Hz, 1H), 5.35 (brs, 1H); **^{13}C NMR (101 MHz, Chloroform-*d*)** δ 146.7 (d, *J* = 226.2 Hz), 129.1 (d, *J* = 18.7 Hz), 126.0 , 124.7 (d, *J* = 3.0 Hz), 112.1 (d, *J* = 8.5 Hz), 108.7 (d, *J* = 21.3 Hz), 102.9; **^{19}F NMR (376 MHz, Chloroform-*d*)** δ -152.67; **ATR-FTIR (cm^{-1})** 2983, 1223, 1043, 846, 607 cm^{-1}; **HRMS m/z (ESI)** called for $C_8H_7FNO^+$ (M + H)$^+$ 152.0506, found 152.0536.

4-Chloro-1*H*-indol-7-ol (52c)

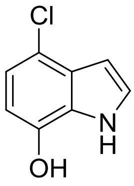

52c

According to the GP-C, **52c** was obtained as a white solid (26.5 mg, 78%), purified by flash column chromatography (PE : EtOAc = 5 : 1). **^{1}H NMR (400 MHz, Chloroform-*d*)** δ 8.49 (brs, 1H), 7.23 (t, *J* = 2.8 Hz, 1H), 6.93 (d, *J* = 8.0 Hz, 1H), 6.63 (dd, *J* = 3.2, 2.3 Hz, 1H), 6.50 (d, *J* = 8.0 Hz, 1H), 5.14 (brs, 1H); **^{13}C NMR (101 MHz, Chloroform-*d*)** δ 140.4, 128.4 126.2 124.6, 119.3, 118.0, 106.9, 101.8; **ATR-FTIR (cm^{-1})** 3323, 1322, 977, 898, 735 cm^{-1}; **HRMS m/z (ESI)** called for $C_8H_7ClNO^+$ (M + H)$^+$ 168.0211, found 168.0256.

6-Chloro-1*H*-indol-7-ol (53c)

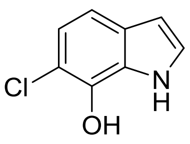

53c

According to the GP-C, **53c** was obtained as a white solid (25.5 mg, 75%), purified by flash column chromatography (PE : EtOAc = 5 : 1). **^{1}H NMR (400 MHz, Chloroform-*d*)** δ 8.40 (brs, 1H), 7.22 – 7.15 (m, 2H), 7.05 (d, *J* = 8.5 Hz, 1H), 6.53 (t, *J* = 2.7 Hz, 1H), 5.69 (s, 1H). **^{13}C NMR (101 MHz, Chloroform-*d*)** δ 137.6, 128.8 125.3 124.7, 120.2, 113.5 111.3, 103.2; **ATR-FTIR (cm^{-1})** 3352, 1268, 1090, 898, 792 cm^{-1}; **HRMS m/z (ESI)** called for $C_8H_7ClNO^+$ $(M + H)^+$ 168.0211, found 168.0261.

5,6-Dichloro-1*H*-indol-7-ol (54c)

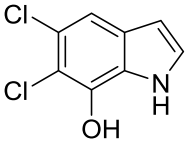

54c

Aaccording to the GP-C, **54c** was obtained as a white solid (26.5 mg, 66%), purified by flash column chromatography (PE : EtOAc = 5 : 1). **^{1}H NMR (400 MHz, Chloroform-*d*)** δ 8.39 (brs, 1H), 7.35 (d, *J* = 0.7 Hz, 1H), 7.22 (dd, *J* = 3.2, 2.4 Hz, 1H), 6.46 (dd, *J* = 3.2, 2.2 Hz, 1H), 5.79 (brs, 1H); **^{13}C NMR (101 MHz, Chloroform-*d*)** δ 138.5, 127.9, 125.6, 123.8, 123.2, 113.7, 110.3, 102.9; **ATR-FTIR (cm^{-1})** 2983, 1446, 938, 846, 607 cm^{-1}; **HRMS m/z (ESI)** called for $C_8H_5Cl_2NO$ (M) 200.9748, found 200.9715.

4-Bromo-1*H*-indol-7-ol (55c)

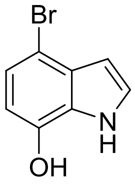

55c

According to the GP-C, **55c** was obtained as a white solid (28.9 mg, 69%), purified by flash column chromatography (PE : EtOAc = 5 : 1). **^{1}H NMR (400 MHz, Chloroform-*d*)** δ 8.55 (brs, 1H), 7.25 – 7.22 (m, 1H), 7.09 (d, *J* = 8.0 Hz, 1H), 6.57 (dd, *J* = 3.2, 2.3 Hz, 1H), 6.47 (d, *J* = 8.0 Hz, 1H), 5.34 (brs, 1H). **^{13}C NMR (101 MHz, Chloroform-*d*)** δ 141.0, 130.2, 126.0, 124.6, 122.5, 107.5, 105.6, 103.4; **ATR-FTIR (cm^{-1})** 2983, 1444, 1091, 1043, 703 cm^{-1}; **HRMS m/z (ESI)** called for $C_8H_7BrNO^+$ $(M + H)^+$ 211.9706, found 211.699.

6-bromo-1*H*-indol-7-ol (56c)

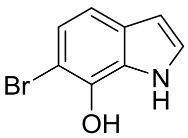

56c

According to the GP-C, **56c** was obtained as a white solid (26.1 mg, 62%), purified by flash column chromatography (PE : EtOAc = 5 : 1). **^{1}H NMR (400 MHz, Chloroform-*d*)** δ 8.43 (brs, 1H), 7.19 (dd, *J* = 3.2, 2.4 Hz, 1H), 7.17 – 7.11 (m, 2H), 6.53 (dd, *J* = 3.1, 2.2 Hz, 1H), 5.66 (brs, 1H); **^{13}C NMR (101 MHz, Chloroform-*d*)** δ 138.6, 129.4, 125.3, 124.6, 122.7, 114.1, 103.2, 101.1; **ATR-FTIR (cm^{-1})** 2985, 1373, 910, 847, 607 cm^{-1}; **HRMS m/z (ESI)** called for $C_8H_7BrNO^+$ (M + H)$^+$211.9706, found 211.9708.

5-Phenyl-1*H*-indol-7-ol (57c)

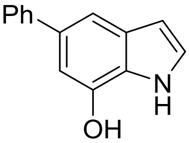

57c

The compound **57c** was prepared according to the GP-C purified by flash column chromatography (PE : EtOAc = 5 : 1), **57c** was obtained as a white solid (29.2 mg, 70%). **^{1}H NMR (400 MHz, Chloroform-*d*)** δ 8.40 (brs, 1H), 7.63 – 7.57 (m, 2H), 7.46 (s, 1H), 7.41 (t, *J* = 7.7 Hz, 2H), 7.30 (t, *J* = 7.4 Hz, 1H), 7.22 (t, *J* = 2.8 Hz, 1H), 6.83 (d, *J* = 1.4 Hz, 1H), 6.58 (dd, *J* = 3.1, 2.1 Hz, 1H), 5.24 (brs, 1H); **^{13}C NMR (101 MHz, Chloroform-*d*)** δ 142.3, 141.5, 134.2, 130.5, 128.6, 127.3, 126.4, 125.2, 124.7, 112.3, 106.4, 103.4; **ATR-FTIR (cm^{-1})** 2984, 1502, 997, 795, 764 cm^{-1}; **HRMS m/z (ESI)** called for $C_{14}H_{12}NO^+$ (M + H)$^+$ 210.0913, found 210.0890.

Genera procedure D

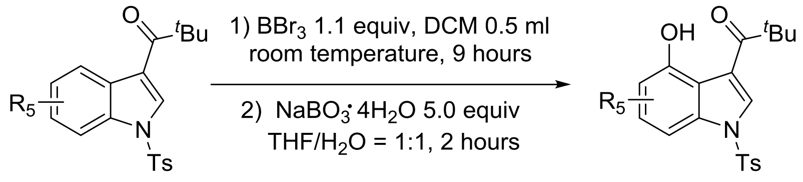

Flame-dried 25 mL Schlenk tube was flushed with argon and charged with indole substrates **61a-69a** (0.2 mmol, 1.0 equiv) and dry DCM (0.5 mL, 0.4 M). A solution of BBr_3 (1.0 M in DCM, 0.22 mL, 1.1 equiv) was added slowly under argon atmosphere. The mixture was stirred at room temperature for 9.0 hours. After stirring,

the solvent was removed under vacuum directly. 0.5 mL THF, $NaBO_3 \cdot 4H_2O$ (153.3 mg, 1.0 mmol) and 0.5 mL H_2O were sequentially added to the reaction mixture and stirred at room temperature for another 2 hours (monitored by TLC). After that, the excess water was removed by filtration with $MgSO_4$ and then washed with EtOAc (10.0 mL × 3). The filtrate was collected and the solvent removed in vacuum and purified by flash column chromatography.

1-(4-Hydroxy-1*H*-indol-3-yl)-2,2-dimethylpropan-1-one (58c)

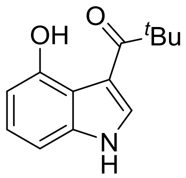

58c

The compound **58c** was performed according to the slightly modified GP-D with 1-(1*H*-indol-3-yl)-2,2-dimethylpropan-1-one (40.2 mg, 0.2 mmol), BBr_3 (1.0 M in DCM, 0.6 mL, 3.0 equiv) in dry DCM (0.5 mL) at 60℃ under Ar for 10 hours. The solvent was removed under vaccum directly, diluted with 0.5 mL THF, $NaBO_3 \cdot 4H_2O$ (229.2 mg, 1.5 mmol) and 0.5 mL H_2O were sequentially added to the reaction mixture and stirred at 60℃ for another 6 hours. After that, the excess water was removed by filtration with $MgSO_4$ and then washed with EtOAc (10.0 mL × 3). The filtrate was collected and the solvent removed in vacuum directly. The crude product was purified by flash column chromatography on silica gel (PE : EtOAc = 5 : 1) to afford 25.7 mg (59 %) of **58c** as a white solid. **^{1}H NMR (400 MHz, DMSO-*d*$_6$)** δ 12.18 (brs, 1H), 11.95 (s, 1H), 8.52 (d, *J* = 3.3 Hz, 1H), 7.05 (t, *J* = 7.9 Hz, 1H), 6.88 (dd, *J* = 8.0, 0.9 Hz, 1H), 6.46 (dd, *J* = 7.7, 0.9 Hz, 1H), 1.39 (s, 9H); **^{13}C NMR (101 MHz, DMSO-*d*$_6$)** δ 204.5, 151.2, 137.5, 134.7, 124.7, 114.9, 112.8, 106.1, 102.3, 42.8, 28.6; **ATR-FTIR (cm^{-1})** 3279, 1587, 1421, 915, 703 cm^{-1}; **HRMS m/z (ESI)** called for $C_{13}H_{16}NO_2^+$ $(M + H)^+$ 218.1176, found 218.1171.

1-(4-Hydroxy-1-methyl-1*H*-indol-3-yl)-2,2-dimethylpropan-1-one (59c)

The compound **59c** was performed according to the slightly modified GP-D with 2,2-Dimethyl-1-(1-methyl-1*H*-indol-3-yl)propan-1-one (43.0 mg, 0.2 mmol), BBr_3

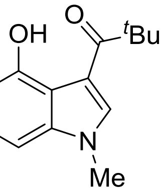

59c

(1.0 M in DCM, 0.6 mL, 3.0 equiv) in dry DCM (0.5 mL) at 60℃ under Ar for 10 hours. The solvent was removed under vaccum directly, diluted with 0.5 mL THF, $NaBO_3·4H_2O$ (229.2 mg, 1.5 mmol) and 0.5 mL H_2O were sequentially added to the reaction mixture and stirred at 60℃ for another 6 hours. After that, the excess water was removed by filtration with $MgSO_4$ and then washed with EtOAc (10.0 mL × 3). The filtrate was collected and the solvent removed in vacuum directly. The crude product was purified by flash column chromatography on silica gel (PE : EtOAc = 5 : 1) to afford 32.9 mg (71 %) of **59c** as a white solid. **^{1}H NMR (400 MHz, Chloroform-*d*)** δ 12.07 (s, 1H), 7.84 (s, 1H), 7.19 (t, *J* = 8.0 Hz, 1H), 6.73 (ddd, *J* = 15.7, 8.0, 0.8 Hz, 2H), 3.79 (s, 3H), 1.43 (s, 9H); **^{13}C NMR (101 MHz, Chloroform-*d*)** δ 204.4, 152.7, 138.7, 135.7, 125.8, 116.4, 113.8, 108.0, 100.2, 43.9, 33.9, 29.6; **ATR-FTIR (cm^{-1})** 2971, 1586, 1412, 1125, 972, 785 cm^{-1}; **HRMS m/z (ESI)** called for $C_{14}H_{18}NO_2^+$ $(M + H)^+$ 232.1332, found 232.1333.

1-(1-benzyl-4-hydroxy-1*H*-indol-3-yl)-2,2-dimethylpropan-1-one (60c)

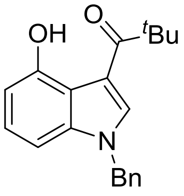

60c

The compound **60c** was performed according to the slightly modified GP-D with 1-(1-benzyl-1*H*-indol-3-yl)-2,2-dimethyl propan-1-one (58.2 mg, 0.2 mmol), BBr_3 (1.0 M in DCM, 0.6 mL, 3.0 equiv) in dry DCM (0.5 mL) at 60℃ under Ar for 10 hours. The solvent was removed under vaccum directly, diluted with 0.5 mL THF, $NaBO_3·4H_2O$ (229.2 mg, 1.5 mmol) and 0.5 mL H_2O were sequentially added to the reaction mixture and stirred at 60℃ for another 6 hours. After that, the excess water was removed by filtration with $MgSO_4$ and then washed with EtOAc (10.0 mL × 3). The filtrate was collected and the solvent removed in vacuum directly. The crude product was purified by flash column chromatography on silica gel (PE : EtOAc = 5 : 1) to afford 33.2 mg (54 %) of **60c** as a white solid. **^{1}H NMR (400 MHz, Chloroform-*d*)** δ 12.04 (s, 1H), 7.88 (s, 1H), 7.39 – 7.31 (m, 3H), 7.19 – 7.12 (m, 3H), 6.72 (ddd, *J* = 7.9, 5.9, 0.8 Hz, 2H), 5.30 (s, 2H), 1.42 (s, 9H); **^{13}C NMR (101**

MHz, Chloroform-*d*) δ 204.6, 152.8, 138.3, 135.3, 135.0, 129.1, 128.3, 126.9, 126.0, 116.6, 114.2, 108.2, 100.8, 51.0, 43.9, 29.6; **ATR-FTIR (cm^{-1})** 2970, 1585, 1385, 1179, 972, 907 cm^{-1}; **HRMS m/z (ESI)** called for $C_{20}H_{22}NO_2^+$ $(M + H)^+$ 308.1645, found 308.1647.

1-(4-Hydroxy-1-tosyl-1*H*-indol-3-yl)-2,2-dimethylpropan-1-one (61c) [1]

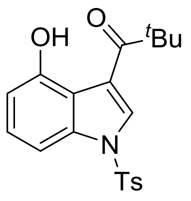

61c

According to the GP-D, **61c** was obtained as a white solid (63.1 mg, 85%), purified by flash column chromatography (PE : EtOAc = 5 : 1).**^{1}H NMR (400 MHz, Chloroform-d)** δ 11.42 (s, 1H), 8.39 (s, 1H), 7.84 – 7.78 (m, 2H), 7.36 (dd, *J* = 8.3, 0.9 Hz, 1H), 7.32 – 7.27 (m, 2H), 7.23 (t, *J* = 8.1 Hz, 1H), 6.77 (dd, *J* = 8.0, 0.9 Hz, 1H), 2.38 (s, 3H), 1.47 (s, 9H); **^{13}C NMR (101 MHz, Chloroform-*d*)** δ 206.0, 152.4, 146.2, 135.7, 134.0, 132.3, 130.3, 128.0, 127.2, 118.4, 116.7, 110.9, 103.7, 44.7, 29.2, 21.7; **ATR-FTIR (cm^{-1})** 3725, 2978, 1697, 1370, 1165, 1080, 660 cm$^{-1;}$ **HRMS m/z (ESI)** called for $C_{20}H_{22}NO_4S^+$ $(M + H)^+$ 372.1264, found 372.1269.

1-(4-Hydroxy-6-methyl-1-tosyl-1*H*-indol-3-yl)-2,2-dimethylpropan-1-one (62c)

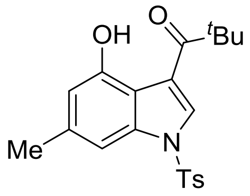

62c

According to the GP-D, **62c** was obtained as a white solid (63.1 mg, 89%), purified by flash column chromatography (PE : EtOAc = 5 : 1). **^{1}H NMR (400 MHz, Chloroform-*d*)** δ 11.32 (s, 1H), 8.32 (s, 1H), 7.80 (d, *J* = 8.5 Hz, 2H), 7.33 – 7.27 (m, 2H), 7.18 (dd, *J* = 1.4, 0.8 Hz, 1H), 6.60 (dd, *J* = 1.3, 0.6 Hz, 1H), 2.38 (s, 3H), 2.37 (s, 3H), 1.46 (s, 9H); **^{13}C NMR (101 MHz, Chloroform-*d*)** δ 205.9, 151.8, 146.0, 138.7, 136.0, 134.1, 131.8, 130.3, 127.1, 118.4, 114.5, 112.2, 104.0, 44.6, 29.2, 21.8, 21.6; **ATR-FTIR (cm^{-1})** 2976, 1615, 1576, 1370, 1173, 1097, 929, 588 cm^{-1}; **HRMS m/z (ESI)** $C_{21}H_{24}NO_4S^+$ $(M + H)^+$ 386.1421, found 386.1416.

1-(5-Fluoro-4-hydroxy-1-tosyl-1*H*-indol-3-yl)-2,2-dimethylpropan-1-one (63c)

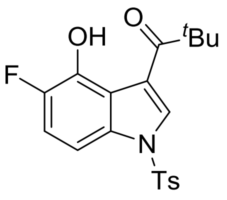

63c

According to the GP-D, **62c** was obtained as a white solid (68.5 mg, 88%), purified by flash column chromatography (PE : EtOAc = 5 : 1). **^{1}H NMR (400 MHz, Chloroform-*d*)** δ 11.61 (s, 1H), 8.39 (s, 1H), 7.82 – 7.76 (m, 2H), 7.34 – 7.26 (m, 3H), 7.11 (dd, *J* = 10.9, 8.9 Hz, 1H), 2.39 (s, 3H), 1.47 (s, 9H); **^{13}C NMR (101 MHz, Chloroform-*d*)** δ 205.9, 148.2 (d, *J* = 237.1 Hz), 146.4, 140.1 (d, *J* = 14.5 Hz), 133.9, 133.5, 131.2, 130.4, 127.2, 118.7 (d, *J* = 4.5 Hz), 115.6 (d, *J* = 22.3 Hz), 102.9 (d, *J* = 7.8 Hz), 44.7, 29.2, 21.7; **^{19}F NMR (376 MHz, Chloroform-*d*)** δ -142.70; **ATR-FTIR (cm^{-1})** 2978, 1640, 1379, 1158, 929, 733 cm^{-1}; **HRMS m/z (ESI)** called for $C_{20}H_{21}FNO_4S^+$ (M + H)$^+$ 390.1170, found 390.1165.

1-(6-Chloro-4-hydroxy-1-tosyl-1*H*-indol-3-yl)-2,2-dimethylpropan-1-one (64c)

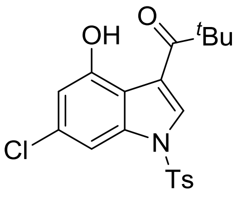

64c

According to the GP-D, **64c** was obtained as a white solid (49.4 mg, 61%), purified by flash column chromatography (PE : EtOAc = 5 : 1). **^{1}H NMR (400 MHz, Chloroform-*d*)** δ 11.63 (s, 1H), 8.35 (s, 1H), 7.80 (d, *J* = 8.1 Hz, 2H), 7.39 (d, *J* = 1.7 Hz, 1H), 7.33 (d, *J* = 8.1 Hz, 2H), 6.76 (d, *J* = 1.6 Hz, 1H), 2.40 (s, 3H), 1.46 (s, 9H); **^{13}C NMR (101 MHz, Chloroform-*d*)** δ 206.0, 152.9, 146.5, 135.7, 133.7, 133.7, 132.5, 130.5, 127.2, 118.3, 115.7, 111.8, 104.0, 44.7, 29.1, 21.7; **ATR-FTIR (cm^{-1})** 2974, 1633, 1364, 1125, 905, 806 cm^{-1}; **HRMS m/z (ESI)** called for $C_{20}H_{21}ClNO_4S^+$ (M + H)$^+$ 406.0874, found 406.0874.

1-(5-Chloro-4-hydroxy-1-tosyl-1*H*-indol-3-yl)-2,2-dimethylpropan-1-one (65c)

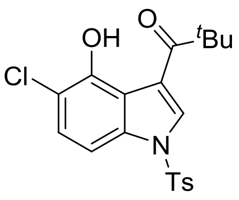

65c

According to the GP-D, **65c** was obtained as a white solid (62.4 mg, 77%), purified by flash column chromatography (PE : EtOAc = 5 : 1). **^{1}H NMR (400 MHz, Chloroform-*d*)** δ 12.19 (s, 1H), 8.39 (s, 1H), 7.81 – 7.76 (m, 2H), 7.34 – 7.28 (m, 4H), 2.38 (s, 3H), 1.46 (s, 9H); **^{13}C NMR (101 MHz, Chloroform-*d*)** δ 206.1, 148.1, 146.5, 134.0, 133.6, 133.0, 130.4, 128.5, 127.2, 118.0, 117.8, 116.0,

104.1, 44.7, 29.2, 21.7; **ATR-FTIR (cm^{-1})** 2975, 1631, 1285, 1088, 940, 819, 597 cm^{-1}; **HRMS m/z (ESI)** called for $C_{20}H_{21}ClNO_4S^+$ $(M + H)^+$ 406.0874, found 406.0870.

1-(5-Bromo-4-hydroxy-1-tosyl-1*H*-indol-3-yl)-2,2-dimethylpropan-1-one (66c)

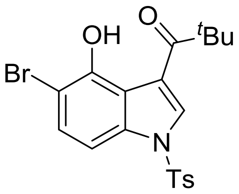

66c

According to the GP-D, **66c** was obtained as a white solid (59.3 mg, 66%), purified by flash column chromatography (PE : EtOAc = 5 : 1). **^{1}H NMR (400 MHz, Chloroform-*d*)** δ 12.28 (s, 1H), 8.37 (s, 1H), 7.81 – 7.75 (m, 2H), 7.47 (d, *J* = 8.8 Hz, 1H), 7.34 – 7.29 (m, 2H), 7.28 – 7.24 (m, 2H), 2.39 (s, 3H), 1.47 (s, 9H); **^{13}C NMR (101 MHz, Chloroform-*d*)** δ 206.1, 149.1, 146.5, 134.7, 133.7, 132.9, 131.4, 130.4, 127.2, 117.9, 117.7, 105.0, 104.7, 44.8, 29.2, 21.7; **ATR-FTIR (cm^{-1})** 2974, 1630, 1378, 1263, 733, 666, 593 cm^{-1}; **HRMS m/z (ESI)** called for $C_{20}H_{21}BrNO_4S^+$ $(M + H)^+$ 450.0369, found 450.0365.

1-(6-Bromo-4-hydroxy-1-tosyl-1*H*-indol-3-yl)-2,2-dimethylpropan-1-one (67c)

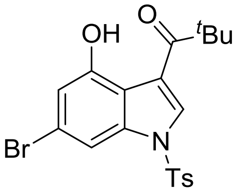

67c

According to the GP-D, **67c** was obtained as a white solid (63.1 mg, 70%), purified by flash column chromatography (PE : EtOAc = 5 : 1). **^{1}H NMR (400 MHz, Chloroform-*d*)** δ 11.62 (s, 1H), 8.33 (s, 1H), 7.80 (d, *J* = 8.4 Hz, 2H), 7.54 (d, *J* = 1.6 Hz, 1H), 7.37 – 7.32 (m, 2H), 6.92 (d, *J* = 1.6 Hz, 1H), 2.41 (s, 3H), 1.46 (s, 9H); **^{13}C NMR (101 MHz, Chloroform-*d*)** δ 205.9, 153.0, 146.5, 136.0, 133.7, 132.4, 130.5, 127.2, 121.2, 118.4, 116.1, 114.5, 106.9, 44.8, 29.1, 21.7; **ATR-FTIR (cm^{-1})** 2973, 1631, 1539, 1179, 943, 893 cm^{-1}; **HRMS m/z (ESI)** called for $C_{20}H_{21}BrNO_4S^+$ $(M + H)^+$ 450.0369, found 450.0364.

1-(4-Hydroxy-6-iodo-1-tosyl-1*H*-indol-3-yl)-2,2-dimethylpropan-1-one (68c)

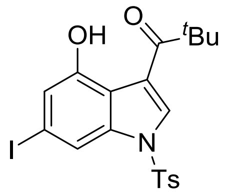

68c

According to the GP-D, **68c** was obtained as a white solid (62.5 mg, 63%), purified by flash column chromatography (PE : EtOAc = 5 : 1). **^{1}H NMR (400 MHz, Chloroform-*d*)** δ 11.55 (s, 1H), 8.29 (s, 1H), 7.81 – 7.78 (m, 2H), 7.73 (d, *J* = 1.4 Hz, 1H), 7.36 – 7.31 (m, 2H), 7.10 (d, *J* = 1.4 Hz, 1H), 2.40 (s, 3H), 1.46 (s, 9H); **^{13}C NMR (101 MHz, Chloroform-*d*)** δ 205.9, 152.8, 146.5, 136.2, 133.7, 132.2, 130.5, 127.2, 120.2, 118.4, 116.7, 112.8, 91.8, 44.7, 29.1, 21.7; **ATR-FTIR (cm^{-1})** 2984, 1630, 1368, 1264, 811, 581 cm^{-1}; **HRMS m/z (ESI)** $C_{20}H_{21}INO_4S^+$ 498.0230, found 498.0226.

1-(4-Hydroxy-6-phenyl-1-tosyl-1*H*-indol-3-yl)-2,2-dimethylpropan-1-one (69c)

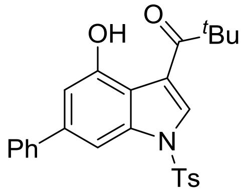

69c

According to the GP-D, **69c** was obtained as a white solid (50.1 mg, 56%) purified by flash column chromatography (PE : EtOAc = 5 : 1). **^{1}H NMR (400 MHz, Chloroform-*d*)** δ 11.81 (s, 1H), 8.42 (s, 1H), 7.84 (d, *J* = 8.4 Hz, 2H), 7.58 – 7.54 (m, 2H), 7.45 – 7.38 (m, 3H), 7.36 – 7.27 (m, 4H), 2.40 (s, 3H), 1.48 (s, 9H); **^{13}C NMR (101 MHz, Chloroform-*d*)** δ 206.1, 149.3, 146.2, 138.7, 135.0, 134.0, 132.7, 130.4, 129.8, 129.6, 128.0, 127.3, 126.6, 124.0, 118.8, 117.3, 103.8, 44.8, 29.3, 21.7; **ATR-FTIR (cm^{-1})** 2972, 1595, 1377, 1089, 939, 811 cm^{-1}; **HRMS m/z (ESI)** called for $C_{26}H_{26}NO_4S^+$ $(M + H)^+$ 448.1577, found 448.1579.

Synthetic applications.

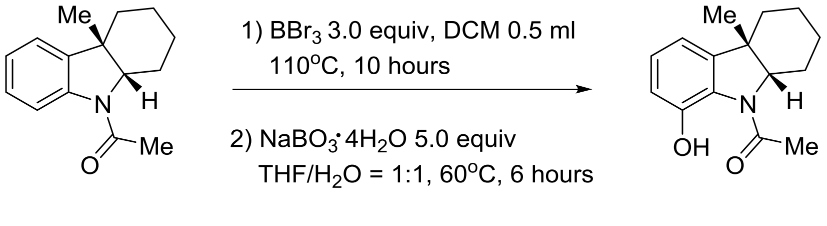

70 71

1-((4aS,9aS)-8-Hydroxy-4a-methyl-1,2,3,4,4a,9a-hexahydro-9H-carbazol-9-yl)ethan-1-one (71)

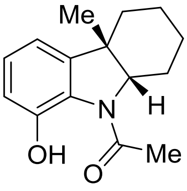

71

To a 10 mL Schlenk tube were added **70** (46.0 mg, 0.2 mmol), BBr_3 (1M in DCM, 0.6 mL, 3.0 equiv), DCM (0.5 mL). The mixture was then stirred at 110 ℃ for 10 hours. After being cooled to room temperature, the solvent was removed under vaccum directly. $NaBO_3·4H_2O$ (153.6 mg, 1.0 mmol), 0.5 mL THF and 0.5 mL H_2O added to the reaction mixture and stired at 60 ℃ for another 6 hours. Afterwards the resulting solution was filtered through a plug of $MgSO_4$ and the residue was washed with EtOAc (10.0 mL × 3). The solvent was removed under vacuum directly and the crude product was purified by silica gel column chromatography (PE : EtOAc = 5 : 1) to afford 35.8 mg (77%) of **71** as a white solid. **^{1}H NMR (400 MHz, Chloroform-*d*)** δ 10.79 (brs, 1H), 7.08 (dd, *J* = 8.1, 7.3 Hz, 1H), 6.82 (dd, *J* = 8.2, 1.2 Hz, 1H), 6.63 (dd, *J* = 7.3, 1.2 Hz, 1H), 3.81 (dd, *J* = 10.0, 6.3 Hz, 1H), 2.33 (s, 3H), 2.31 – 2.24 (m, 1H), 2.05 – 1.97 (m, 1H), 1.66 – 1.48 (m, 3H), 1.28 – 1.12 (m, 6H); **^{13}C NMR (101 MHz, Chloroform-*d*)** δ 168.9, 147.8, 141.5, 127.6, 126.8, 117.7, 112.4, 70.2, 43.9, 32.4, 30.6, 28.6, 23.1, 22.6, 21.8; **ATR-FTIR (cm^{-1})** 2928, 1628, 1574, 1472, 1259, 734 cm^{-1}; **HRMS m/z (ESI)** called for $C_{15}H_{20}NO_2^+$ $(M + H)^+$ 246.1489, found 246.1485.

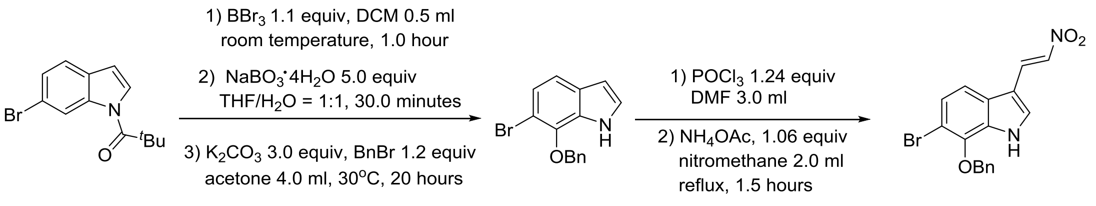

7-(Benzyloxy)-6-bromo-1*H*-indole (73) [11]

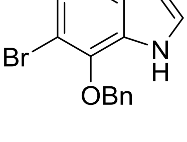

73

To a 10 mL Schlenk tube were added **72** (139.5mg, 0.5 mmol), BBr_3 (1M in DCM, 0.55 mL, 1.1 equiv), DCM (0.5 mL). The mixture was then stirred at room temperature for 1 hour. After that, the solvent was removed under vaccum directly. $NaBO_3·4H_2O$ (91.8 mg, 0.6 mmol), 0.5 mL THF and 0.5 mL H_2O added to the reaction mixture and stired at room

temperature for another 1 hour. Afterwards the resulting solution was filtered through a plug of $MgSO_4$ and the residue was washed with EtOAc (10.0 mL × 3). The solvent was removed under vacuum directly to get a red reaction mixture.

To this reaction mixture were added K_2CO_3 (207.3 mg, 1.5 mmol, 3.0 equiv), BnBr (103 mg, 0.6 mmol, 1.2 equiv),acetone (4.0 ml). The mixture was then stirred at 30℃ for 20 hours. After being cooled to room temperature, the reaction was diluted with DCM and filtered through a pad of Celite, which was washed with DCM (10.0 mL × 3). The solvent was removed under vacuum directly and the crude product was purified by silica gel column chromatography (PE : EtOAc = 5 : 1) to afford 94.0 mg (63%) of **73** as a white solid. **^{1}H NMR (400 MHz, Chloroform-*d*)** δ 7.94 (s, 1H), 7.54 – 7.48 (m, 2H), 7.47 – 7.37 (m, 3H), 7.29 (d, *J* = 0.8 Hz, 2H), 7.04 (dd, *J* = 3.1, 2.4 Hz, 1H), 6.50 (dd, *J* = 3.2, 2.1 Hz, 1H), 5.19 (s, 2H); **^{13}C NMR (101 MHz, Chloroform-*d*)** δ 141.7, 137.4, 131.0, 129.4, 128.8, 128.5, 128.4, 124.7, 124.4, 117.8, 108.5, 103.3, 75.7; **ATR-FTIR (cm^{-1})** 3434, 1606, 1327, 1216, 733, 697 cm^{-1}; **HRMS m/z (ESI)** called for $C_{15}H_{12}BrNNaO^+$ $(M + Na)^+$ 323.9994, found 324.0003.

(*E*)-7-(Benzyloxy)-6-bromo-3-(2-nitrovinyl)-1*H*-indole (74) [11]

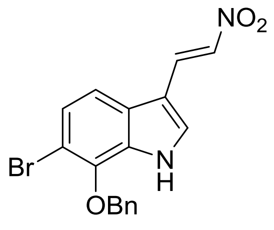

74

Freshly-distilled $POCl_3$ (38.4mg,0.25mmol, 1.24equiv) was added dropwise to dry DMF (1 mL) at 0 °C, stirred for 15 min then transferred via a cannula to a solution of indole **73** (60.1 mg, 0.2 mmol) in DMF (1.0 mL). The reaction mixture was stirred at 0 °C for 30 minutes, then at 40 °C for 1 hour. The resulting yellow mixture was cooled to 0 °C then basified with a 1 N solution of NaOH before being heated to reflux. After 20 minutes, the reaction mixture was cooled to ambient temperature, then diluted with EtOAc (10.0 mL), washed with 10% aqueous NaCl (10.0 mL), brine (10.0 ml) and concentrated the organic phase to get a yellow reaction mixture.

To this reaction mixture were added nitromethane (2.0 ml), NH_4OAc (24.8 mg, 0.32 mmol, 1.06 equiv) at room temperature. The yellow solution was then heated to reflux

for 1.5 hours before being cooled to room temperature. Concentration and purification by silica gel chromatography (DCM : MeOH : H_2O : NH_4OH = 90 : 10 : 0.6 : 0.6) to afford **74** (67.7mg, 91%) as an orange solid. **^{1}H NMR (500 MHz, DMSO-*d*$_6$)** δ 12.57 (s, 1H), 8.39 (d, *J* = 13.4 Hz, 1H), 8.29 (s, 1H), 8.04 (d, *J* = 13.5 Hz, 1H), 7.73 (d, *J* = 8.5 Hz, 1H), 7.65 – 7.59 (m, 2H), 7.45 – 7.35 (m, 4H), 5.12 (s, 2H); **^{13}C NMR (126 MHz, DMSO-*d*$_6$)** δ 141.8, 136.5, 136.3, 133.8, 132.2, 131.9, 128.6, 128.3, 128.2, 126.7, 125.9, 117.8, 109.8, 109.0, 74.9;**ATR-FTIR (cm^{-1})** 3234, 1618, 1301, 1228, 1110, 975, 749 cm^{-1}; **HRMS m/z (ESI)** called for $C_{17}H_{14}BrN_2O_3^+$ $(M + H)^+$ 373.0182, found 373.0182.

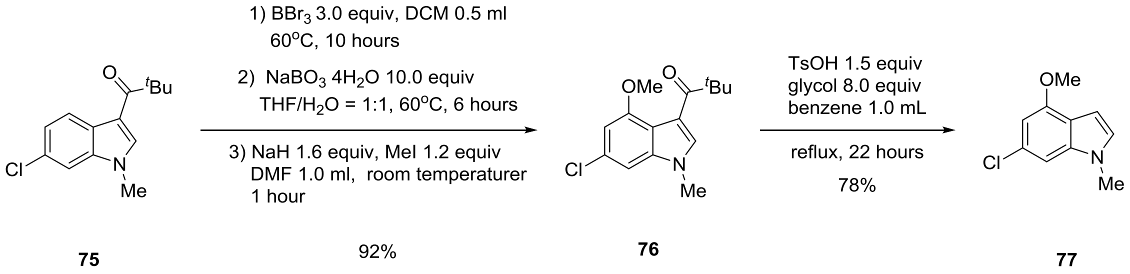

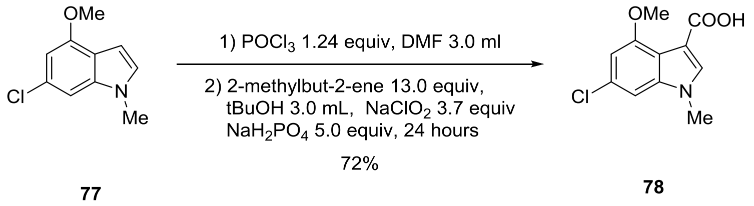

1-(6-Chloro-4-methoxy-1-methyl-1*H*-indol-3-yl)-2,2-dimethylpropan-1-one (76)

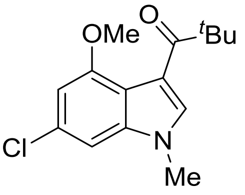

76

To a 10 mL Schlenk tube were added **75** (48.8mg, 0.2 mmol), BBr_3 (1M in DCM, 0.6 mL, 3.0 equiv), DCM (0.5 mL). The mixture was then stirred at 60°C for 10 hours. After that, the solvent was removed under vaccum directly. $NaBO_3$·$4H_2O$ (304.3 mg, 2.0 mmol),1.0 mL THF and 1.0 mL H_2O added to the reaction mixture and stired at 60°C for another 6 hours. After being cooled to room temperature, the excess water was removed by filtration with $MgSO_4$ and then washed

with EtOAc (10.0 mL × 3). The filtrate was collected and the solvent removed in vacuum directly. The crude product was purified by flash column chromatography on silica gel (PE : EtOAc = 5 : 1) to afford 50.8 mg (96 %) of 1-(6-chloro-4-hydroxy-1-methyl-1*H*-indol-3-yl)-2,2-dimethylpropan-1-one as a white solid.

The indole derivative (53.0 mg, 0.2 mmol, 1.0 equiv.) was dissolved in dry DMF (1.0 ml) and at 0 ℃. NaH (12.8mg, 0.32 mg, 1.6 eq., 60% dispersion in mineral oil) was added portion wise. The ice bath was removed and the reaction mixture was stirred at ambient temperature. After 30 minutes, methyl iodide (34.1mg, 0.24 mmol, 1.2 equiv.) was added at 0 ℃, afterwards the reaction mixture was allowed to warm to room temperature and stirred for another 1 hour. Water was carefully added and the aqueous phase was extracted using DCM (10.0 ml). The combined organic layers were washed with water (10.0 mL × 2) and sat NaCl aq (10.0 mL × 2). Then the combined organic layers were dried using Na_2SO_4 and after filtration the solvent was removed in vacuum. The crude product was purified by flash column chromatography on silica gel (PE : EtOAc = 5 : 1) to afford 55.1 mg (99 %) of **76** as a white solid. **^{1}H NMR (400 MHz, Chloroform-*d*)** δ 7.02 (s, 1H), 6.93 (d, *J* = 1.6 Hz, 1H), 6.54 (d, *J* = 1.6 Hz, 1H), 3.87 (s, 3H), 3.71 (s, 3H), 1.27 (s, 9H); **^{13}C NMR (101 MHz, Chloroform-*d*)** δ 207.9, 153.7, 137.6, 129.3, 126.6, 115.7, 115.2, 102.7, 102.3, 55.6, 45.0, 33.2, 27.1; **ATR-FTIR (cm^{-1})** 2964, 1680, 1478, 1122, 898, 735 cm^{-1}; **HRMS m/z (ESI)** called for $C_{15}H_{19}ClNO_2^+$ $(M + H)^+$ 280.1099, found 280.1103.

6-Chloro-4-methoxy-1-methyl-1*H*-indole (77)

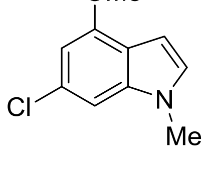

77

A solution of **76** (55.8 mg, 0.2 mmol), TsOH (51.7 mg, 0.3 mmol, 1.5 equiv) and glycol (99.3 mg, 1.6 mmol, 8.0 equiv) in benzene (1.0 mL) as heated under reflux conditions for 22 hours. The solvent was then removed under reduced pressure. The crude product was purified by column chromatography (PE : EtOAc = 10 : 1) to afford 30.4 mg (78%) of **77** as a white solid. **^{1}H NMR (400 MHz, Chloroform-*d*)** δ 6.97 (t,

J = 1.2 Hz, 1H), 6.93 (d, J = 3.1 Hz, 1H), 6.55 (dd, J = 3.2, 0.9 Hz, 1H), 6.53 (d, J = 1.5 Hz, 1H), 3.94 (s, 3H), 3.72 (s, 3H); **^{13}C NMR (101 MHz, Chloroform-*d*)** δ 153.4, 137.8, 128.0, 127.7, 117.6, 102.8, 100.9, 98.5, 55.5, 33.0; **ATR-FTIR (cm^{-1})** 2937, 1606, 1575, 1068, 884, 763 cm^{-1}; **HRMS m/z (ESI)** called for $C_{10}H_{11}ClNO^+$ (M + H)$^+$ 196.0524, found 196.0528.

6-Chloro-4-methoxy-1-methyl-1*H*-indole-3-carboxylic acid (78) [12]

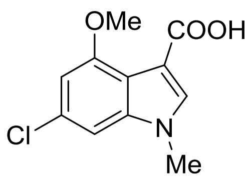

78

Freshly-distilled $POCl_3$ (38.4mg,0.25mmol, 1.24equiv) was added dropwise to dry DMF (1 mL) at 0 °C, stirred for 15 min then transferred via a cannula to a solution of **77** (39.0 mg, 0.2 mmol) in DMF (1.0 mL). The reaction mixture was stirred at 0 °C for 30 minutes, then at 40 °C for 1 hour. The resulting yellow mixture was cooled to 0 °C then basified with a 1 N solution of NaOH before being heated to reflux. After 20 minutes, the reaction mixture was cooled to ambient temperature, then diluted with EA (20.0 mL), washed with 10% aqueous NaCl (10.0 mL), brine (10.0 ml) and concentrated to get a yellow reaction mixture.

To a stirred solution of yellow product (0.18 mmol, 1.0 equiv) and 2-methylbut-2-ene (182 mg, 2.6 mmol, 13.0 equiv) in tBuOH (3.0 mL) were added a saturated solution of $NaClO_2$ (66.8 mg, 0.74 mmol, 3.7 equiv) and NaH_2PO_4 (156.1 mg, 1.0 mmol, 5.0 equiv). The mixture was stirred 24 hours at room temperature. The mixture was quenched with saturated NH_4Cl and extracted with EtOAc(10.0 mL × 3). The combined organic layers were washed with brine, dried over Na_2SO_4, filtered, concentrated, and purified by silica gel column chromatography (PE : EtOAc = 10 : 1) to give the desired **77** (34.4 mg, 72%) as a white solid. **^{1}H NMR (500 MHz, DMSO-*d*$_6$)** δ 11.62 (s, 1H), 7.96 (s, 1H), 7.27 (d, J = 1.7 Hz, 1H), 6.74 (d, J = 1.7 Hz, 1H), 3.88 (s, 3H), 3.78 (s, 3H); **^{13}C NMR (126 MHz, DMSO-*d*$_6$)** δ 163.8, 153.4, 138.8, 136.8, 127.9, 113.8, 107.2, 104.0, 103.4, 56.1, 33.3; **ATR-FTIR (cm^{-1})** 3434, 1660, 1051, 1023, 1004, 821, 758 cm^{-1}; **HRMS m/z (ESI)** called for $C_{11}H_{11}ClNO_3^+$ (M + H)$^+$ 240.0422, found 240.0420.

Supplementary References

1 Lv, J., Chen, H., Houk, K. N., Shi, Z. Metal-free directed sp^2-C–H borylation. *Nature*. **575**, 336–340 (2019).

2 Lritch, J. A., Wilson, P. B., Frost, C. G. Ruthenium(II)-Catalyzed C−H Functionalization Using the Oxazolidinone Heterocycle as a Weakly Coordinating Directing Group: Experimental and Computational Insights. *ACS Catal.* **6**, 5520−5529 (2016).

3 Yang, X., Shan, G., Rao, Y. Synthesis of 2‑ Aminophenols and Heterocycles by Ru-Catalyzed C-H Mono- and Dihydroxylation. *Org. Letter.* **15**, 2334-2337 (2013)

4 Hwlmick, J. S., Novak, M. Mechanism of the reaction of carbon and nitrogen nucleophiles with the model carcinogens *O*-pivaloyl-*N*-arylhydroxylamines: competing SN2 substitution and SN1 solvolysis. *J. Am. Chem. Soc.* **113**, 3459-3466 (1991).

5 Yang, Y. H., Shi, M. Selective syntheses of benzoxazoles and N-(2-hydroxyaryl)pyrrolidin-2-ones from the corresponding cyclopropyl amides with PPh_3/CX_4. *Tetrahedron*, **62**, 2420-2427 (2006).

6 Kalyani, D., Sanford, M. S. Regioselectivity in Palladium-Catalyzed C-H Activation / Oxygenation Reactions. *Organic Letters*, **7**, 4149-4152 (2005).

7 Esguerra, K. V. Unified Synthesis of 1,2-Oxy-aminoarenes via a Bio-inspired Phenol-Amine Coupling. *Chem*, **2**, 533-549 (2017).

8 Bassoli, A. Synthesis of a new family of *N*-aryl lactams active on chemesthesis and taste. *European Journal of Organic Chemistry*, **7**, 1656-1663 (2006).

9 Musolino, M., Arico. F. Benzo-Fused 1,4-Heterocycles via Dialkyl Carbonate Chemistry. *Synthesis*, **51**, 1770-1778 (2019).

10. Mishrs, A., Vats, T. K., Deb, I.. Rhodium-Catalyzed sp^2 C–H Acetoxylation of N-Aryl Azaindoles/N-Heteroaryl Indolines. *J. Org. Chem*, **82**, 12406-12415 (2017).

11 Sofiyev, V., Trauner, D. Total Synthesis of Exiguamines A and B Inspired by Catecholamine Chemistry. *Chem. Eur. J.* **18**, 4999–5005 (2012).

12 Zhang, X., Renata, H. Total Synthesis of Tambromycin by Combining Chemocatalytic and Biocatalytic C-H Functionalization. *Angew .Chem. Int.Ed.* **57**, 5037–5041 (2018).

Supplementary Figures

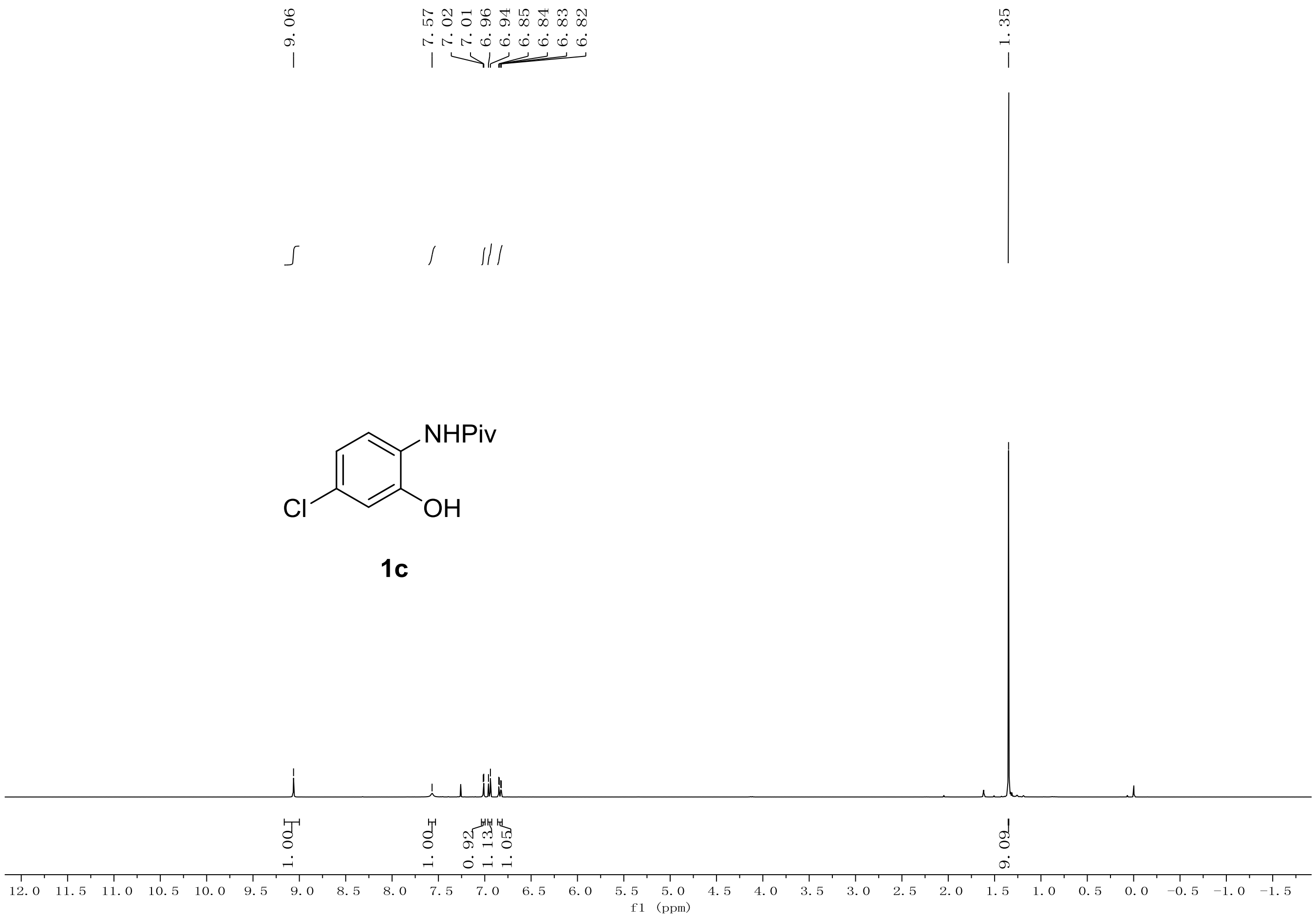

Supplementary Figure 1. ^{1}H NMR spectrum for **1c**

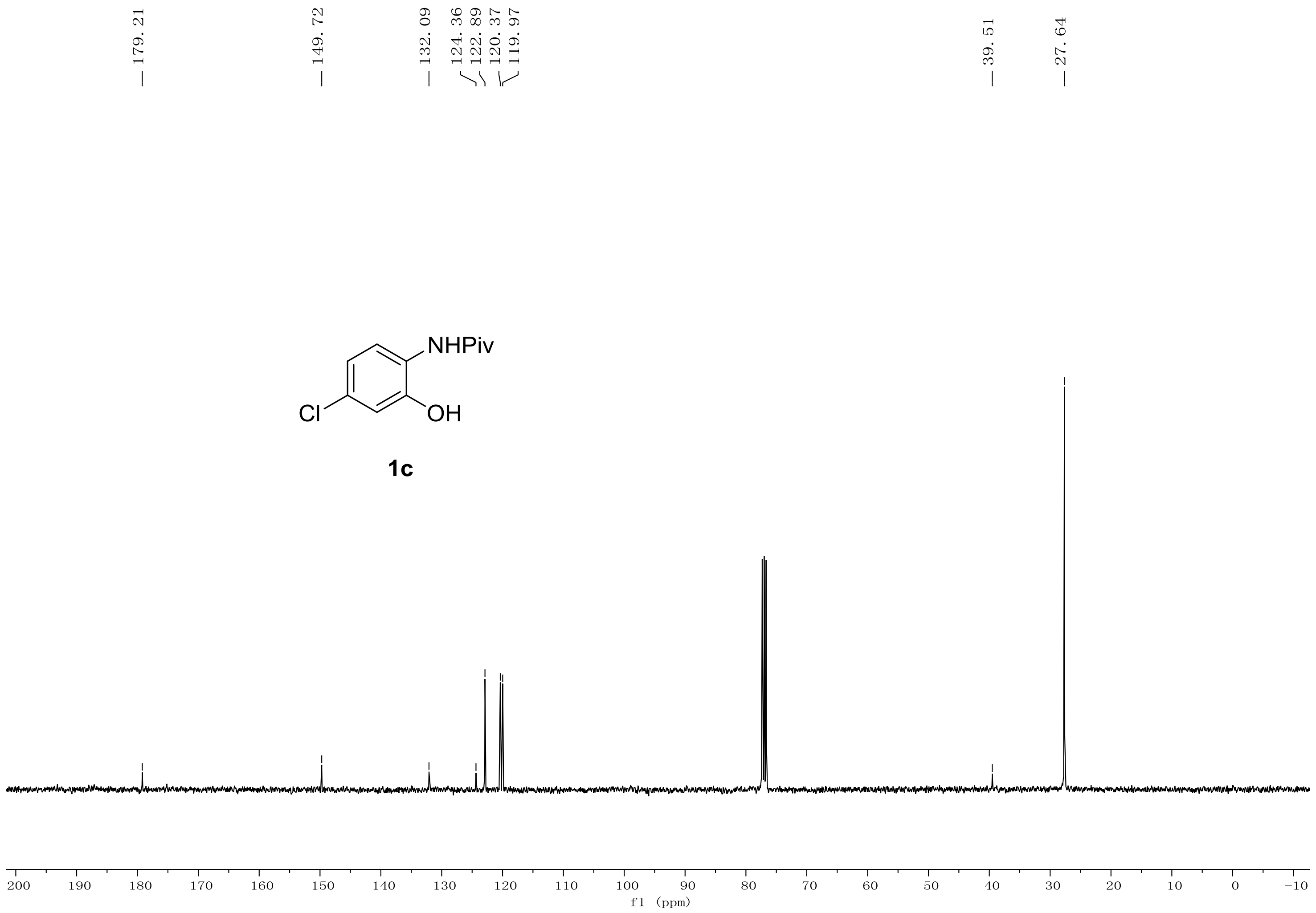

Supplementary Figure 2. ^{13}C NMR spectrum for **1c**

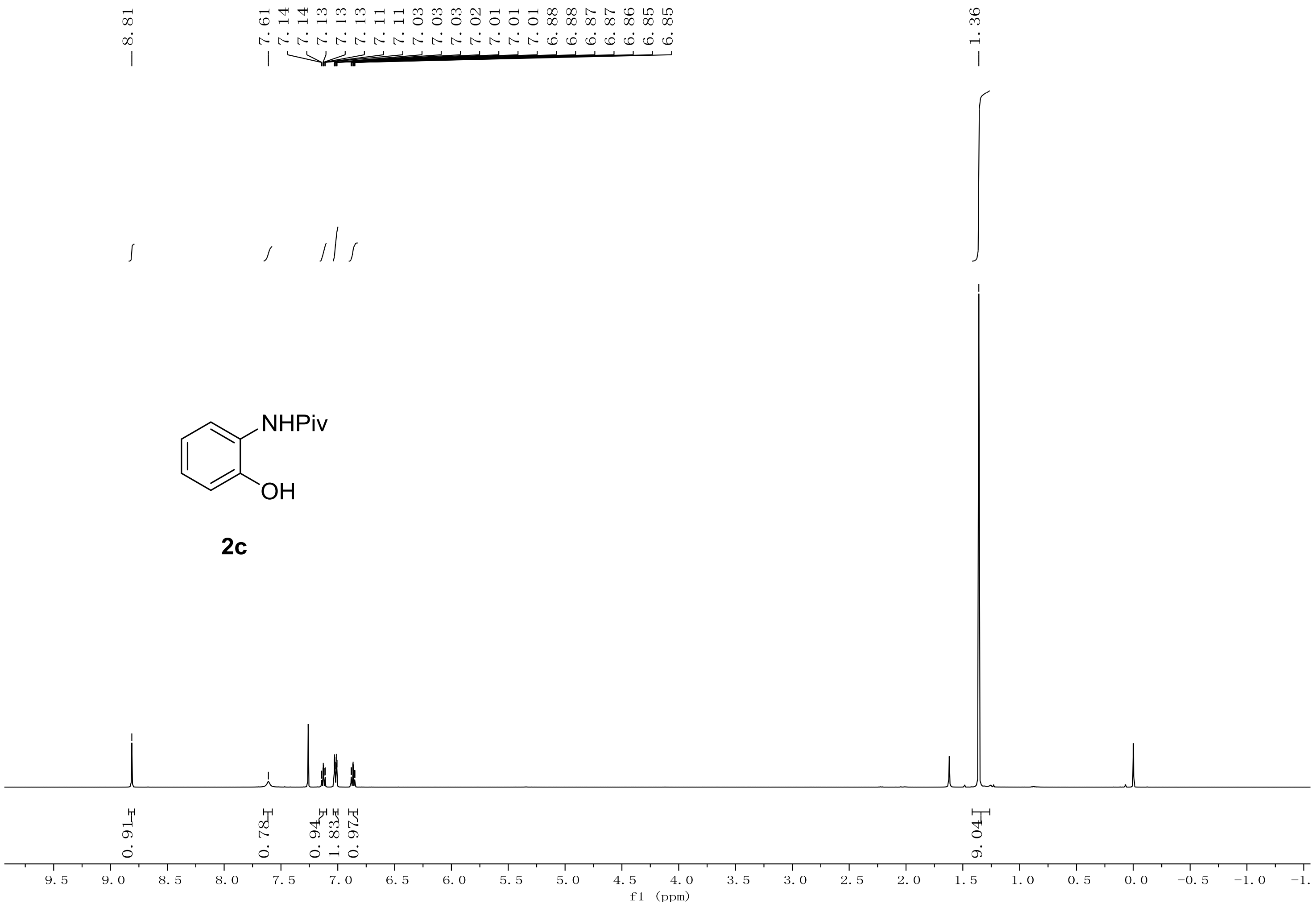

Supplementary Figure 3. ^{1}H NMR spectrum for **2c**

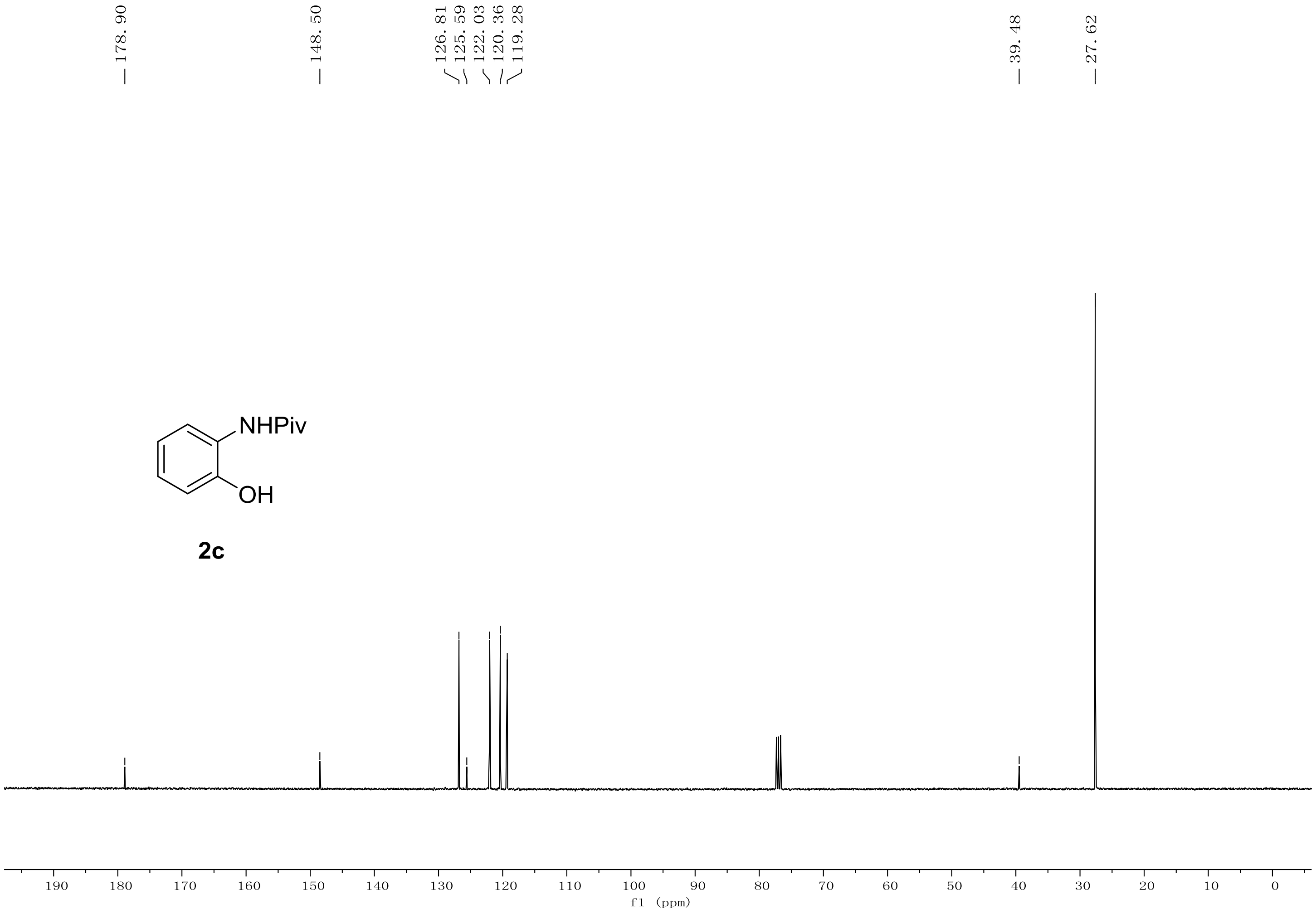

Supplementary Figure 4. ^{13}C NMR spectrum for **2c**

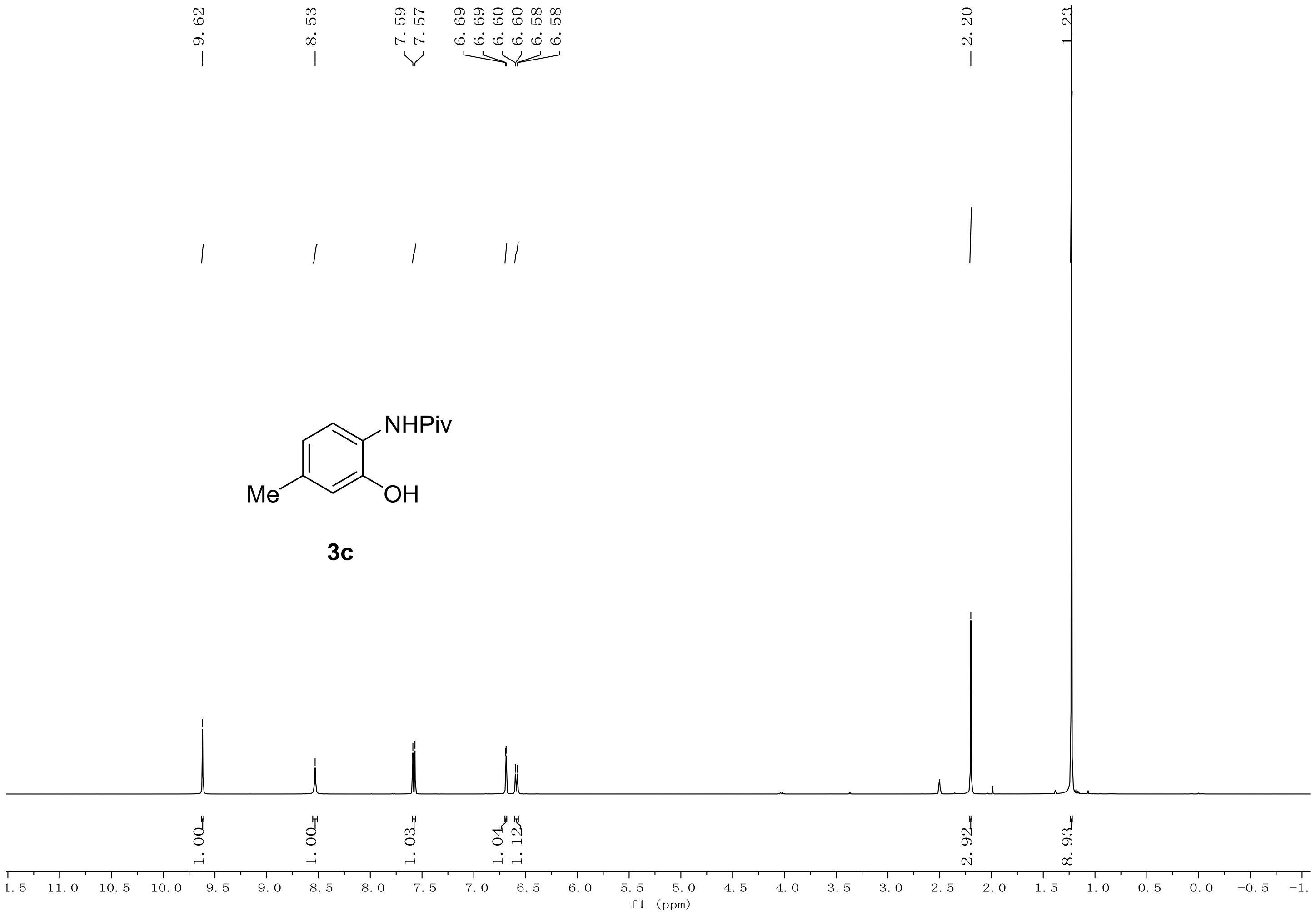

Supplementary Figure 5. ^{1}H NMR spectrum for **3c**

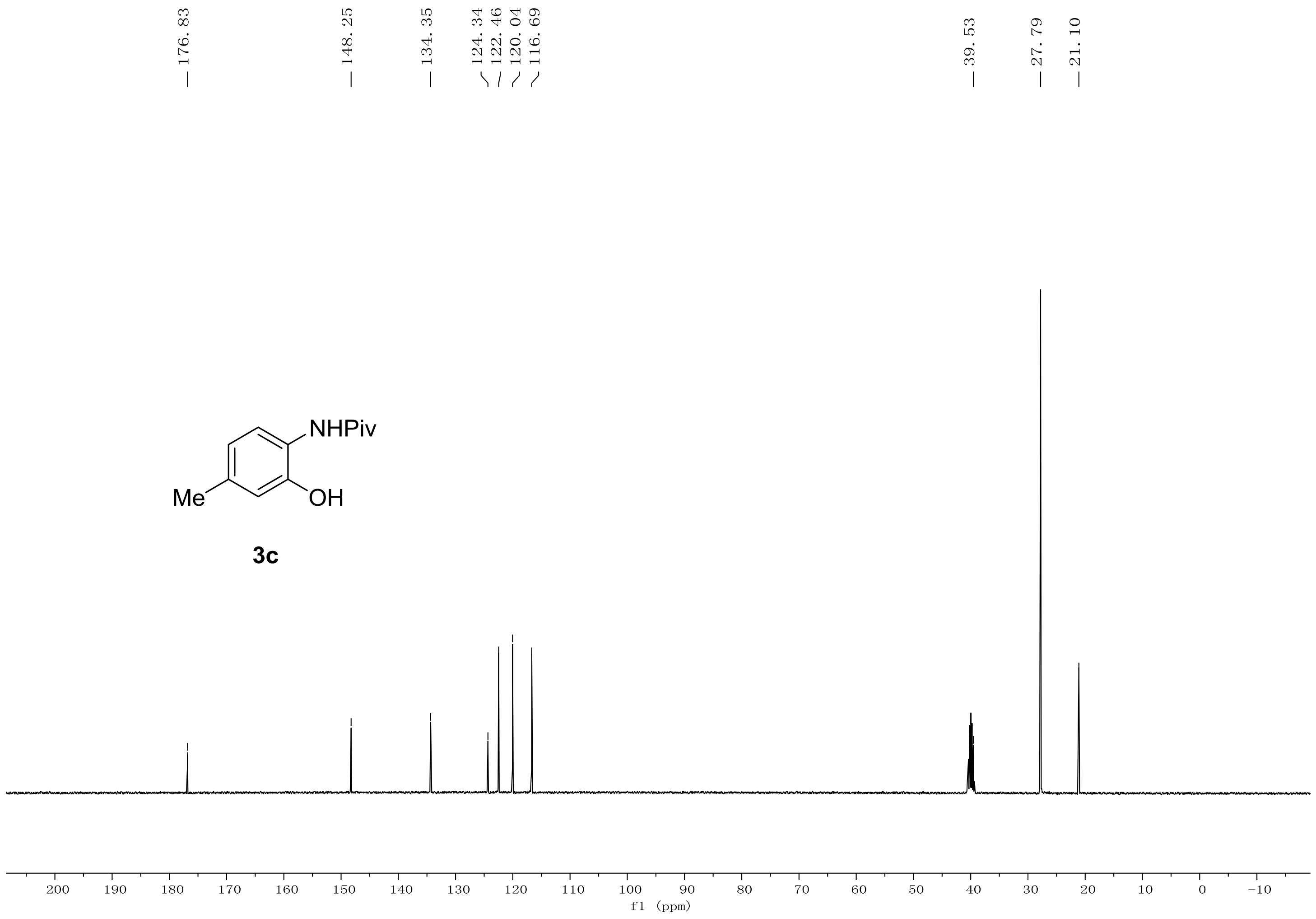

Supplementary Figure 6. ^{13}C NMR spectrum for **3c**

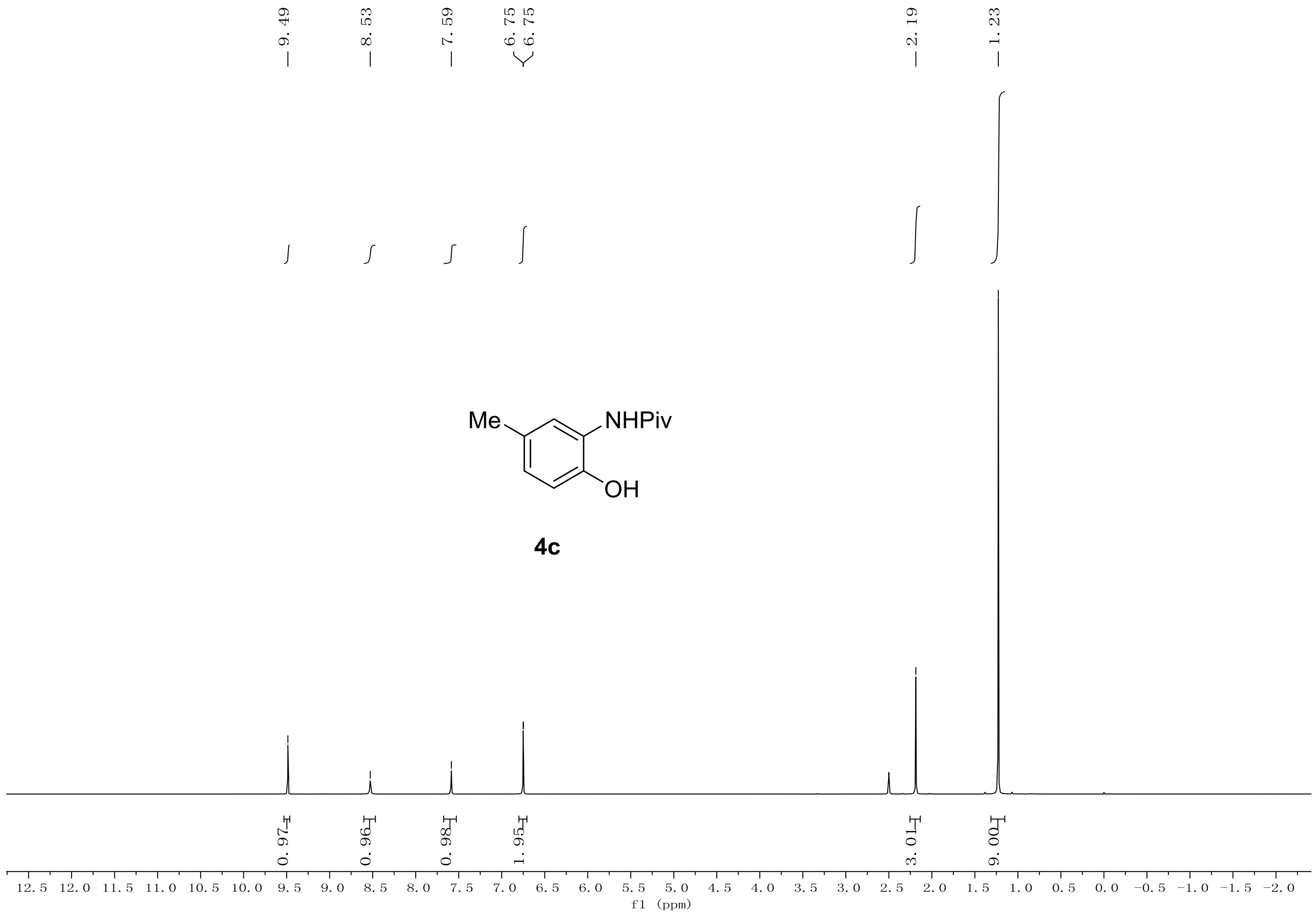

Supplementary Figure 7. ^{1}H NMR spectrum for **4c**

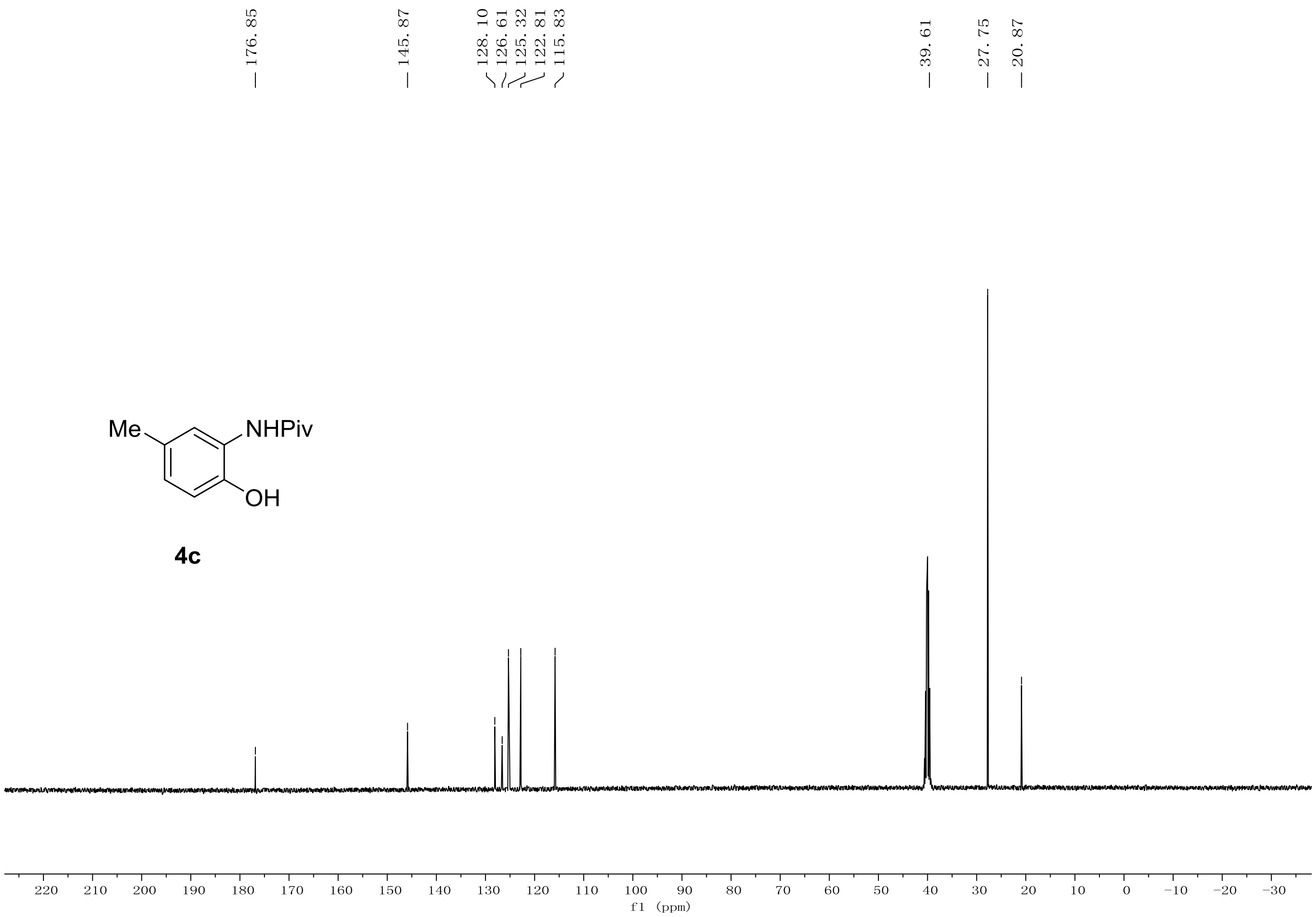

Supplementary Figure 8. ^{13}C NMR spectrum for **4c**

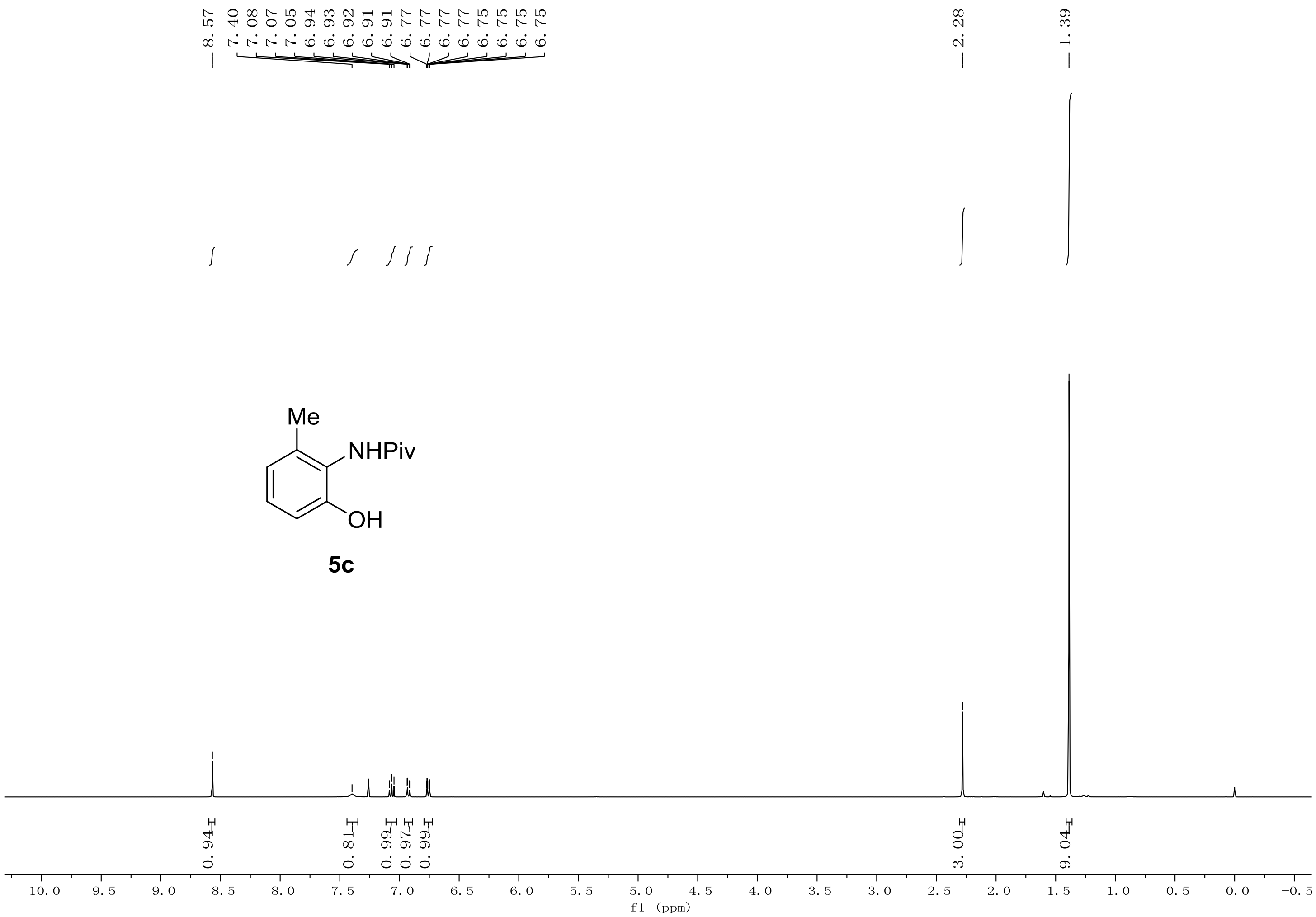

Supplementary Figure 9. ^{1}H NMR spectrum for **5c**

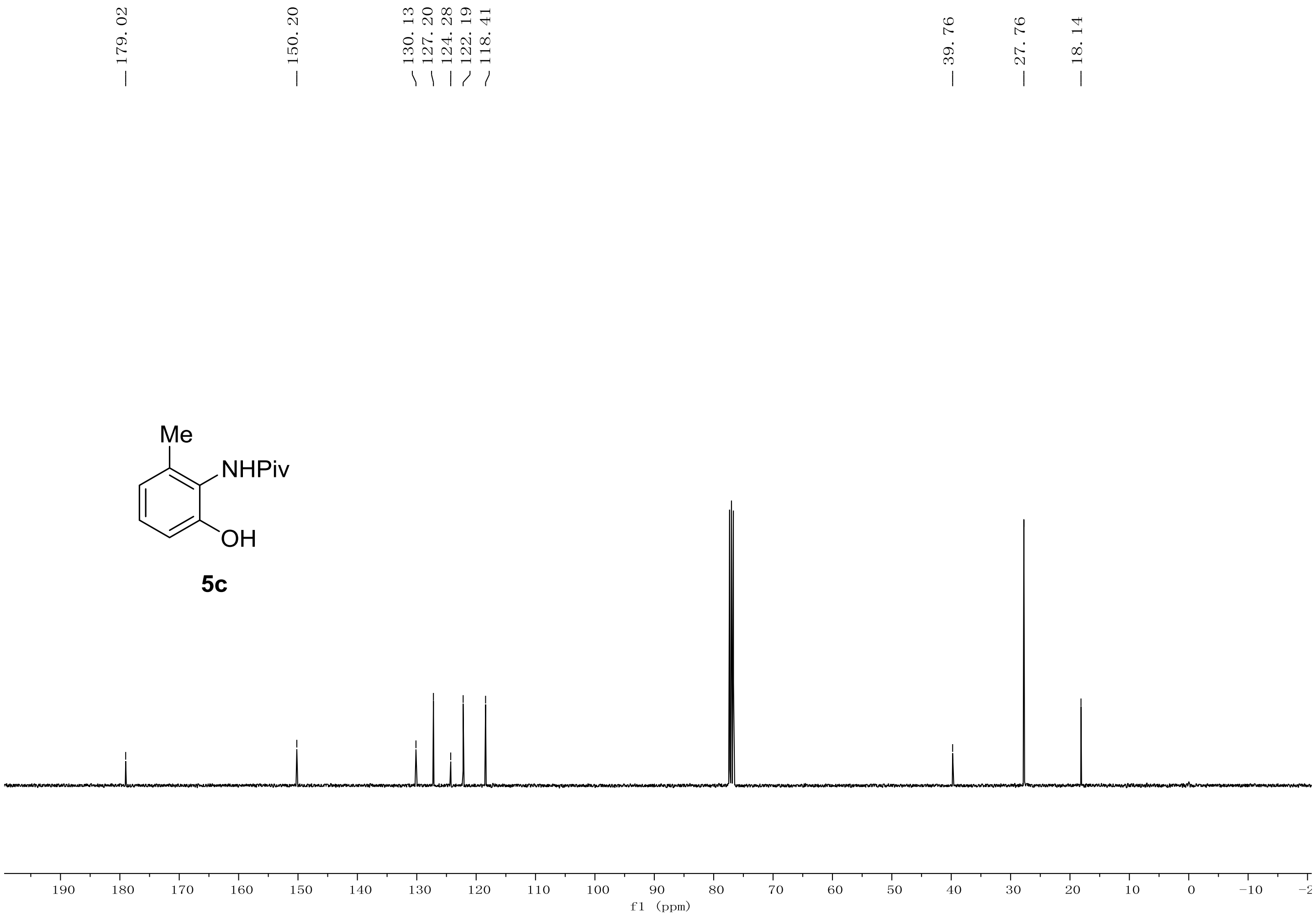

Supplementary Figure 10. ^{13}C NMR spectrum for **5c**

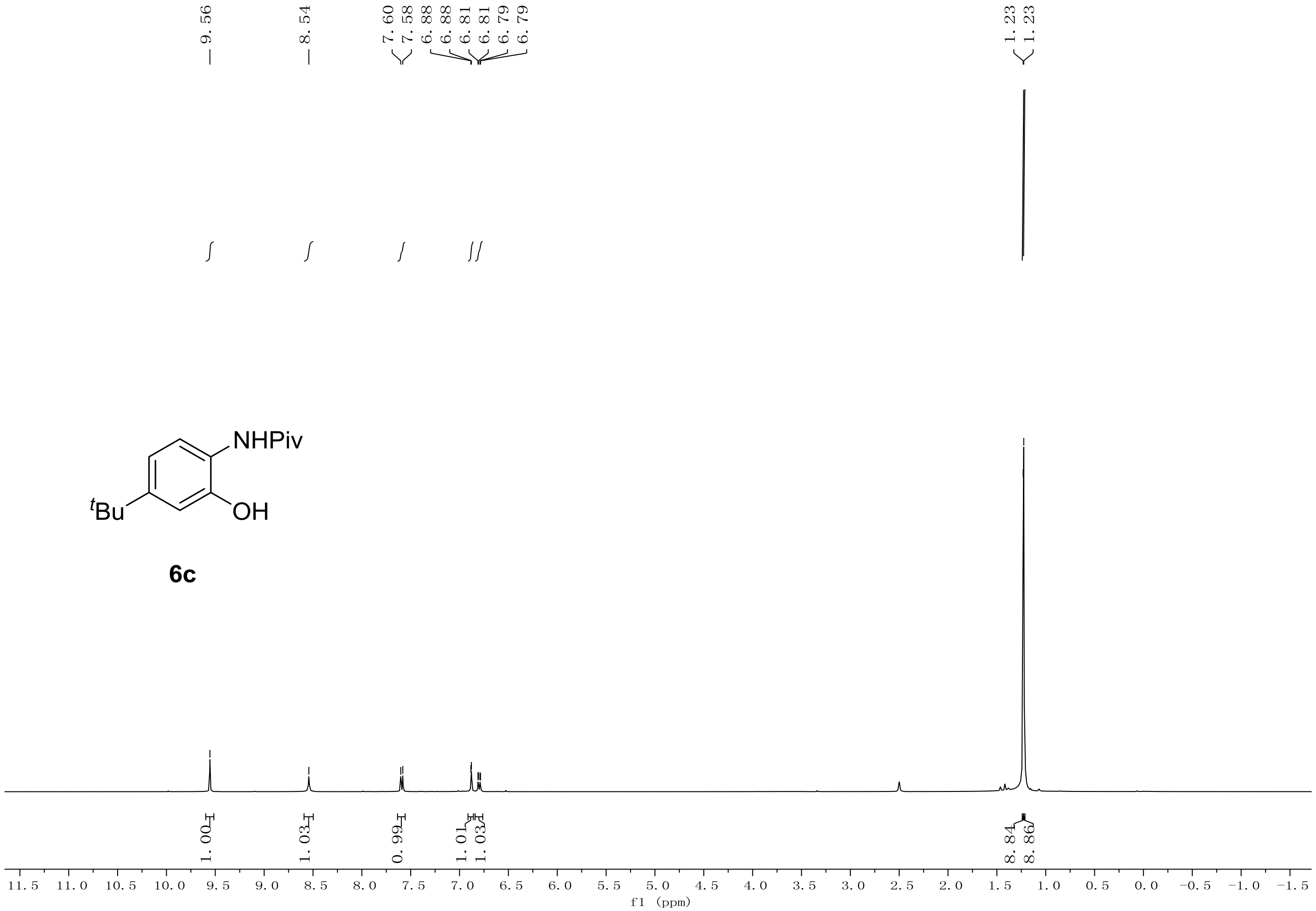

Supplementary Figure 11. ^{1}H NMR spectrum for **6c**

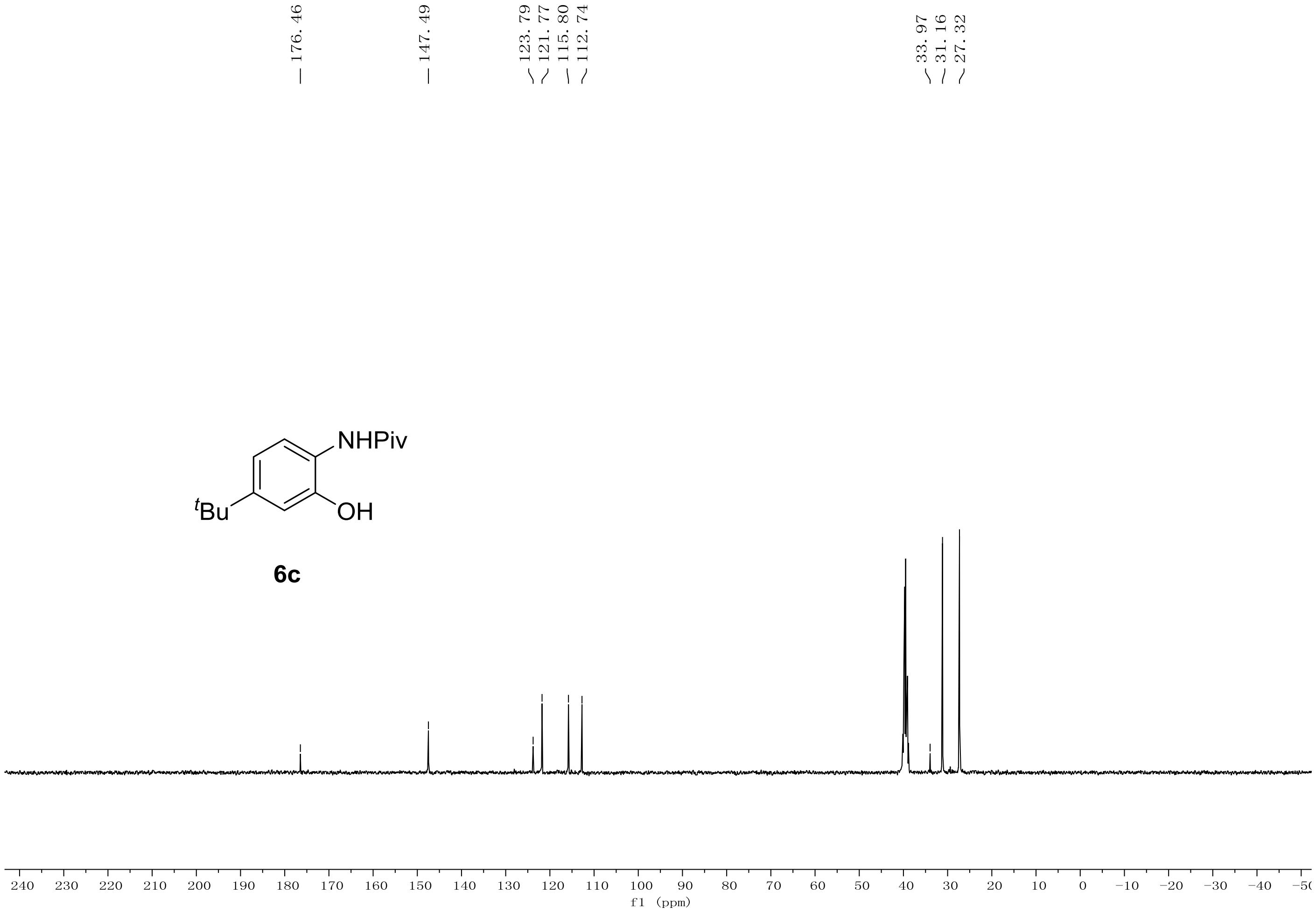

Supplementary Figure 12. ^{13}C NMR spectrum for **6c**

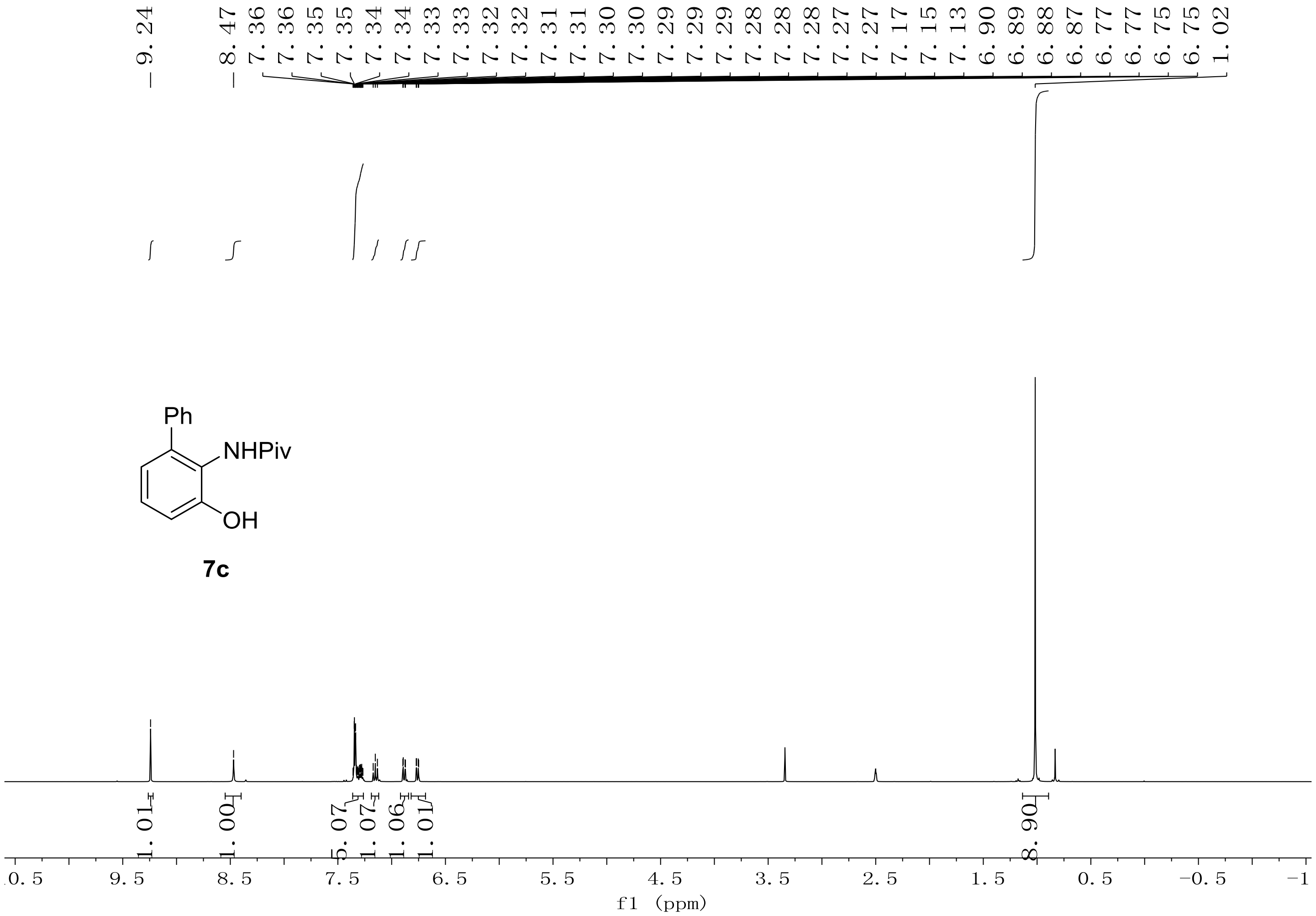

Supplementary Figure 13. ^{1}H NMR spectrum for **7c**

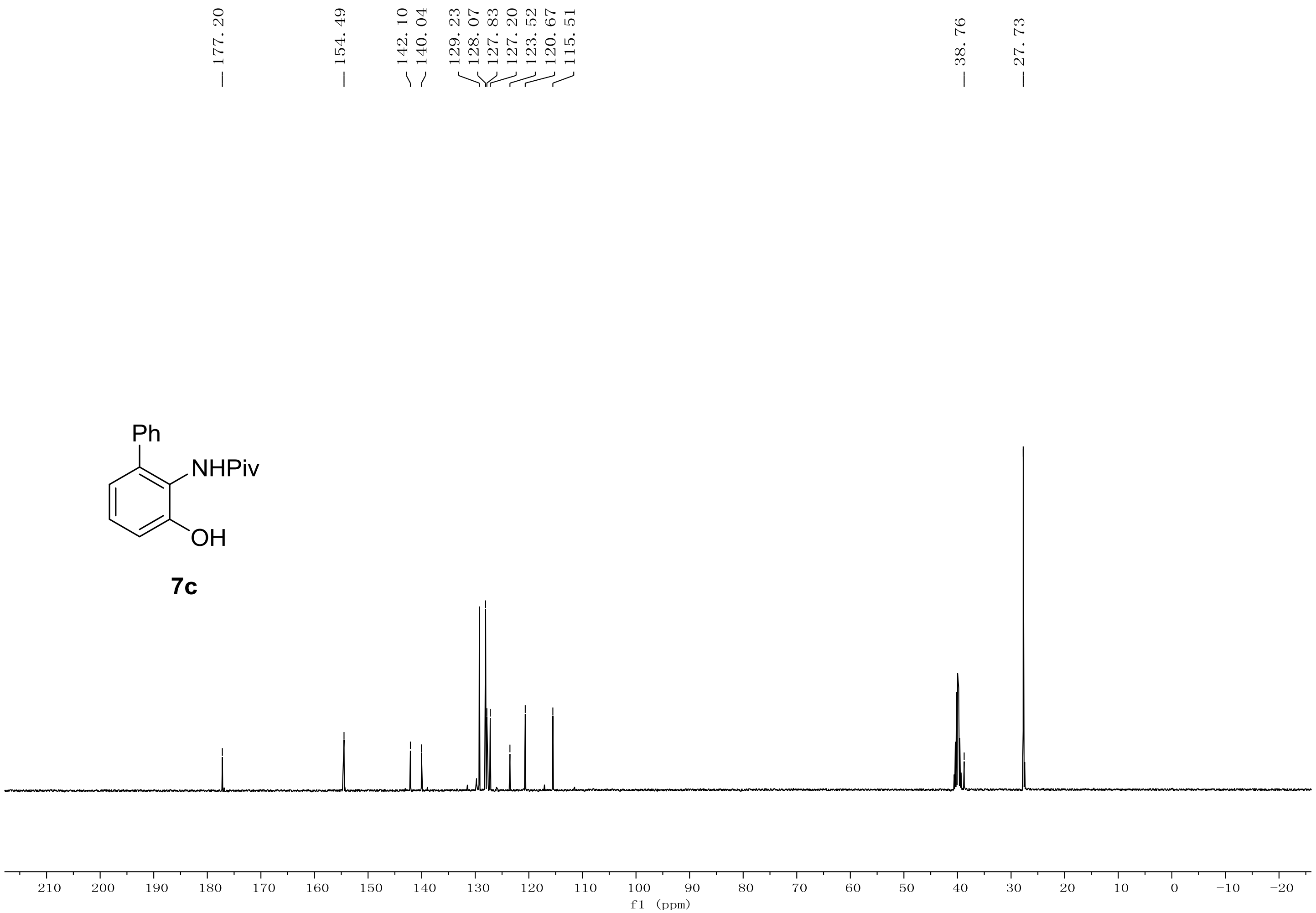

Supplementary Figure 4. ^{13}C NMR spectrum for **7c**

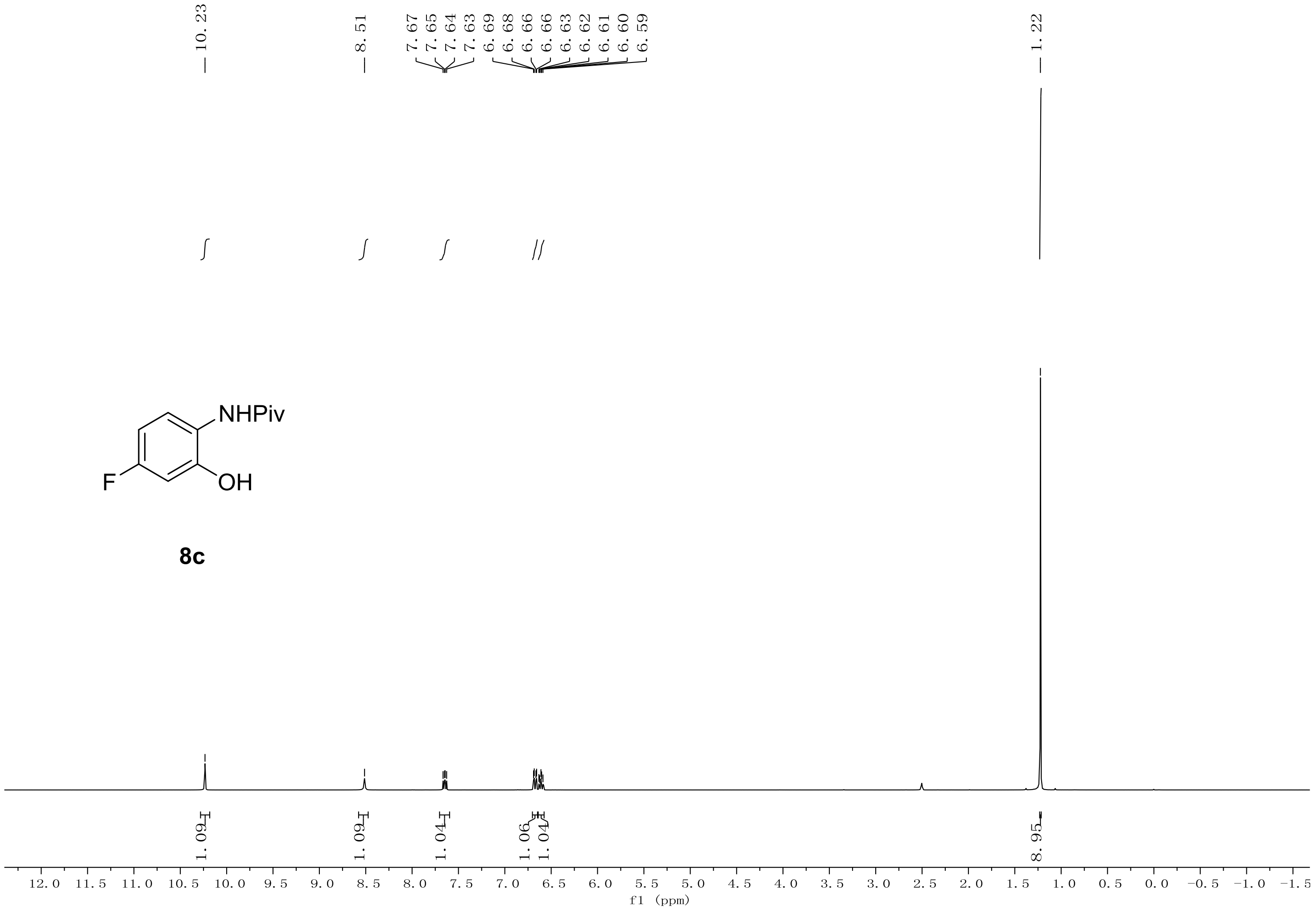

Supplementary Figure 15. ^{1}H NMR spectrum for **8c**

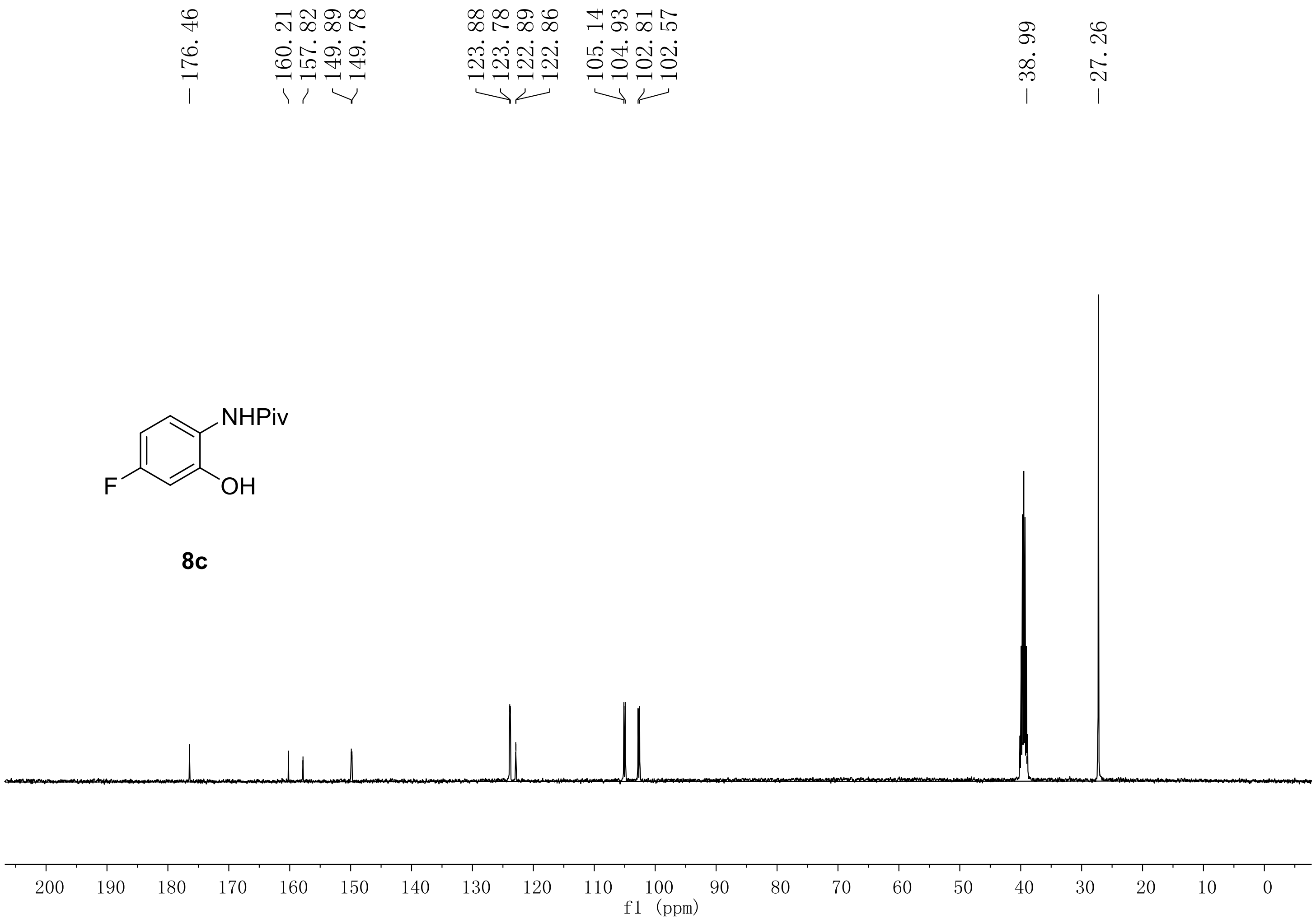

Supplementary Figure 16. ^{13}C NMR spectrum for **8c**

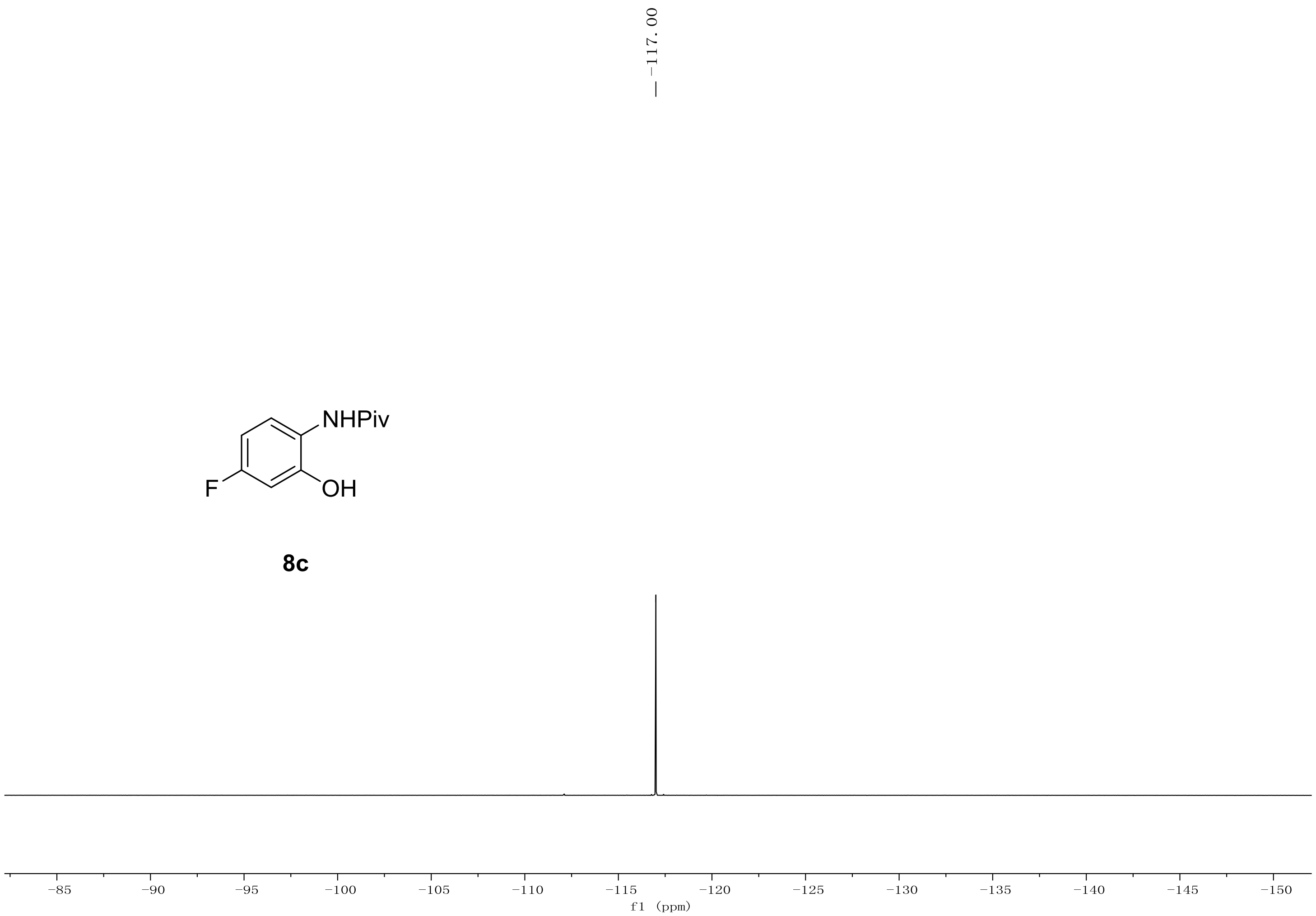

Supplementary Figure 17. ^{19}F NMR spectrum for **8c**

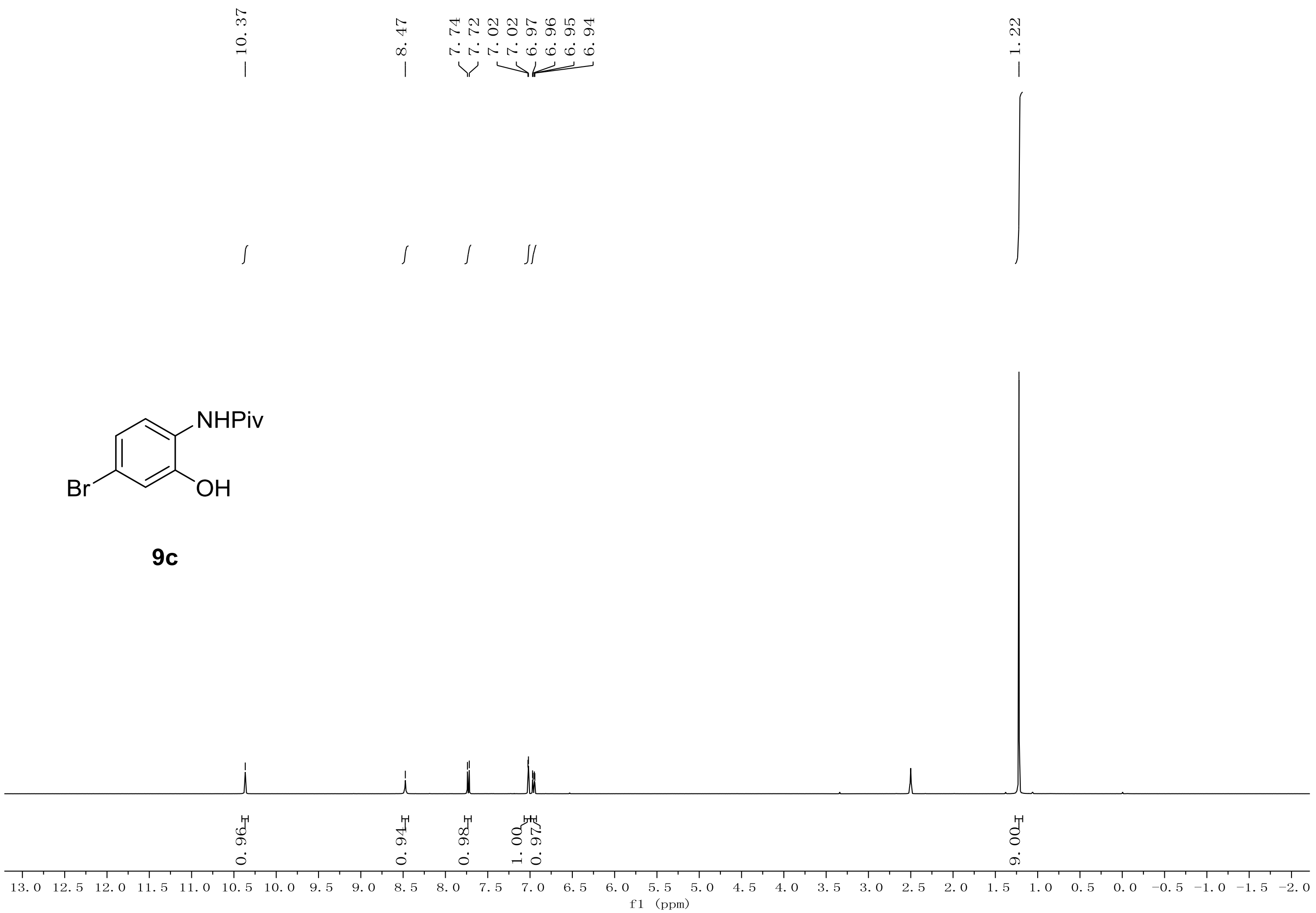

Supplementary Figure 18. ^{1}H NMR spectrum for **9c**

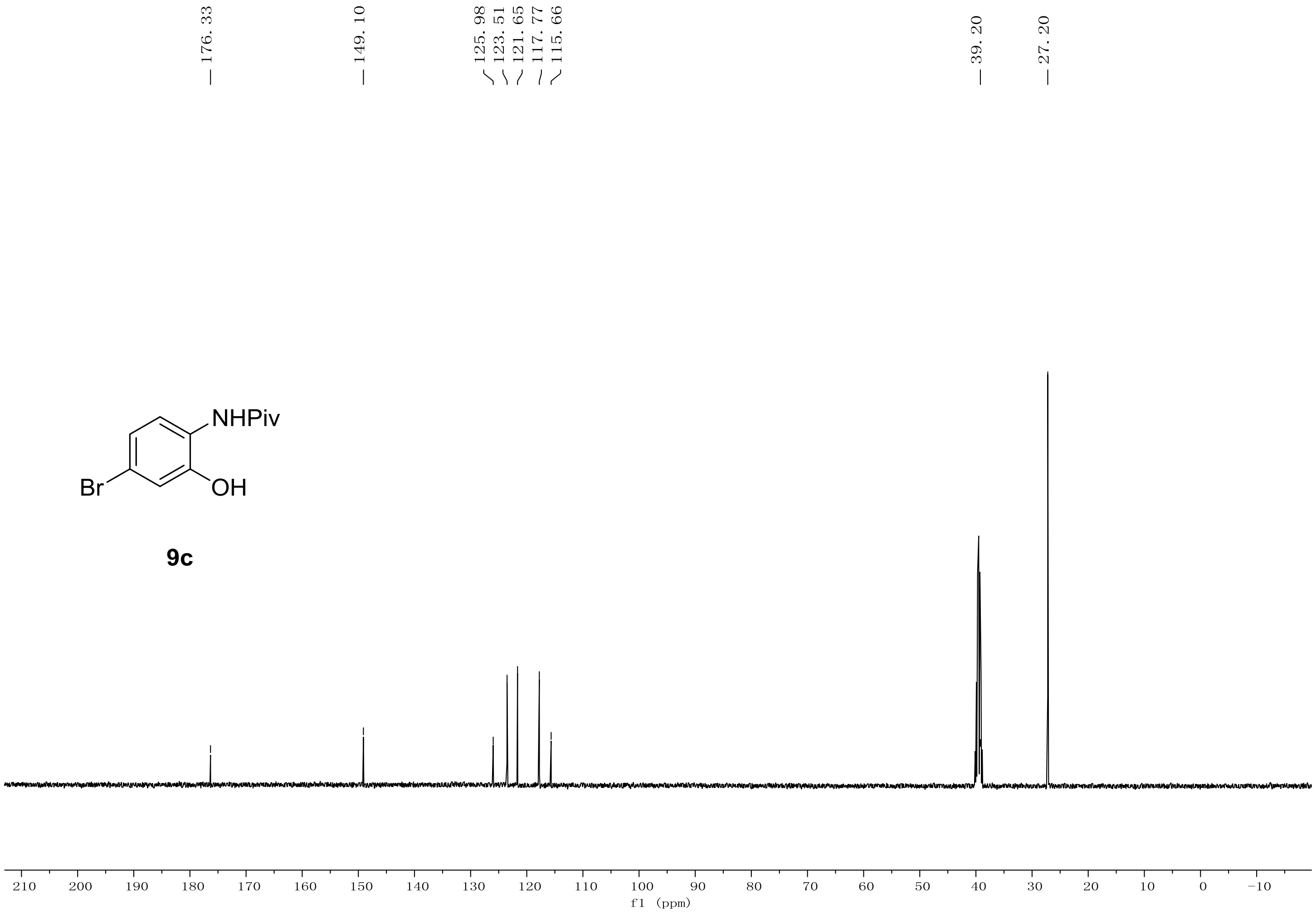

Supplementary Figure 19. ^{13}C NMR spectrum for **9c**

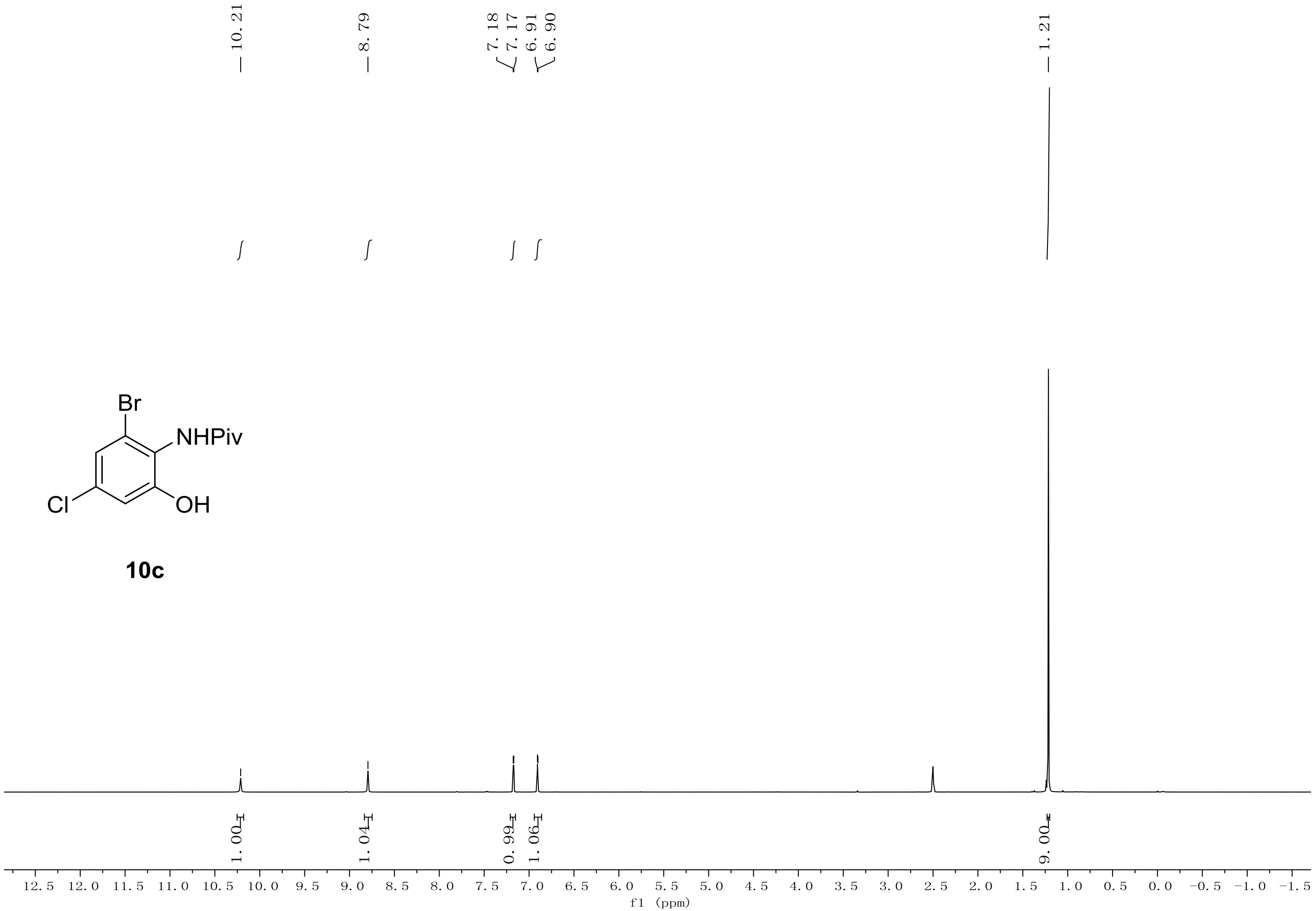

10c

Supplementary Figure 20. ^{1}H NMR spectrum for **10c**

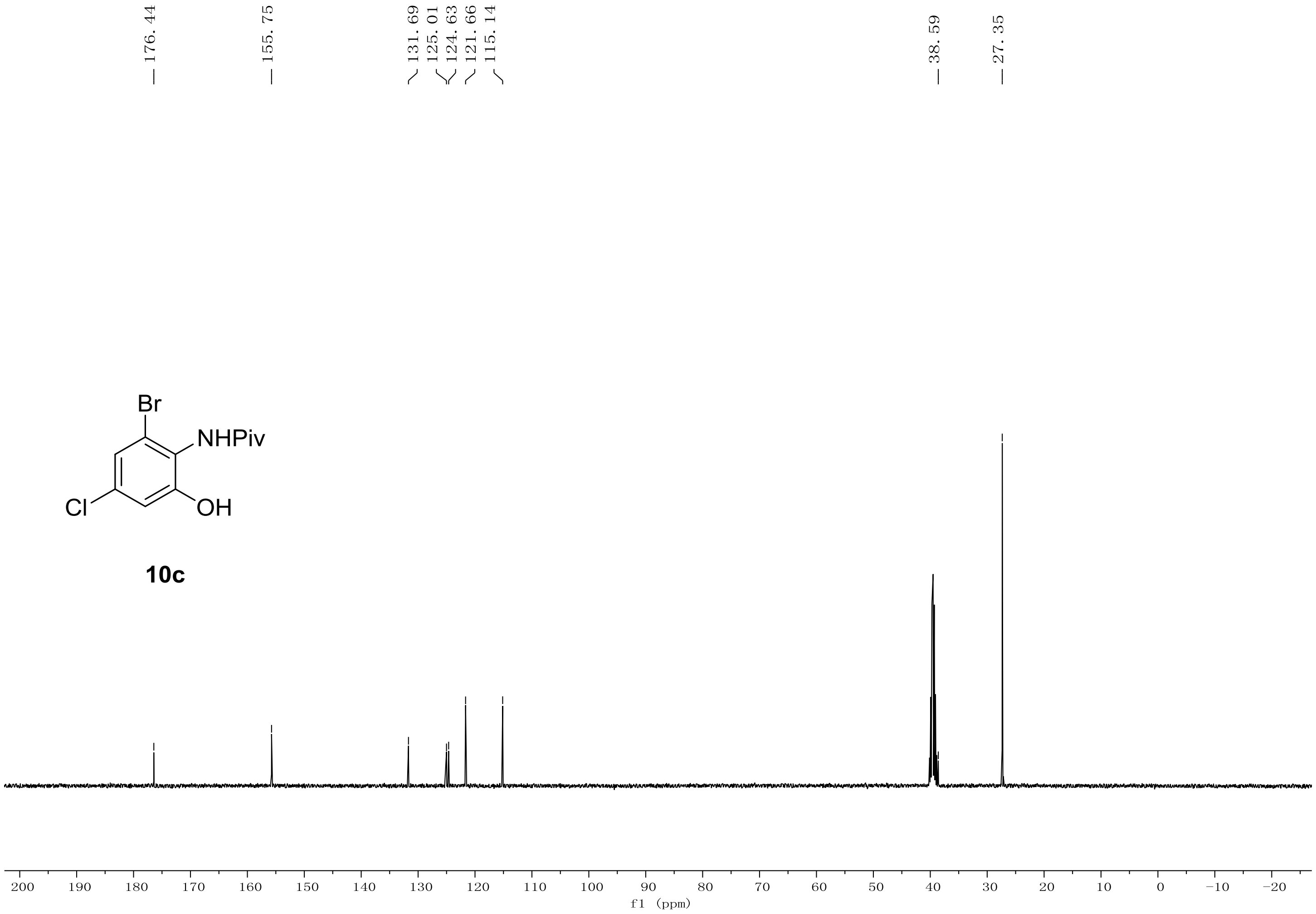

Supplementary Figure 21. ^{13}C NMR spectrum for **10c**

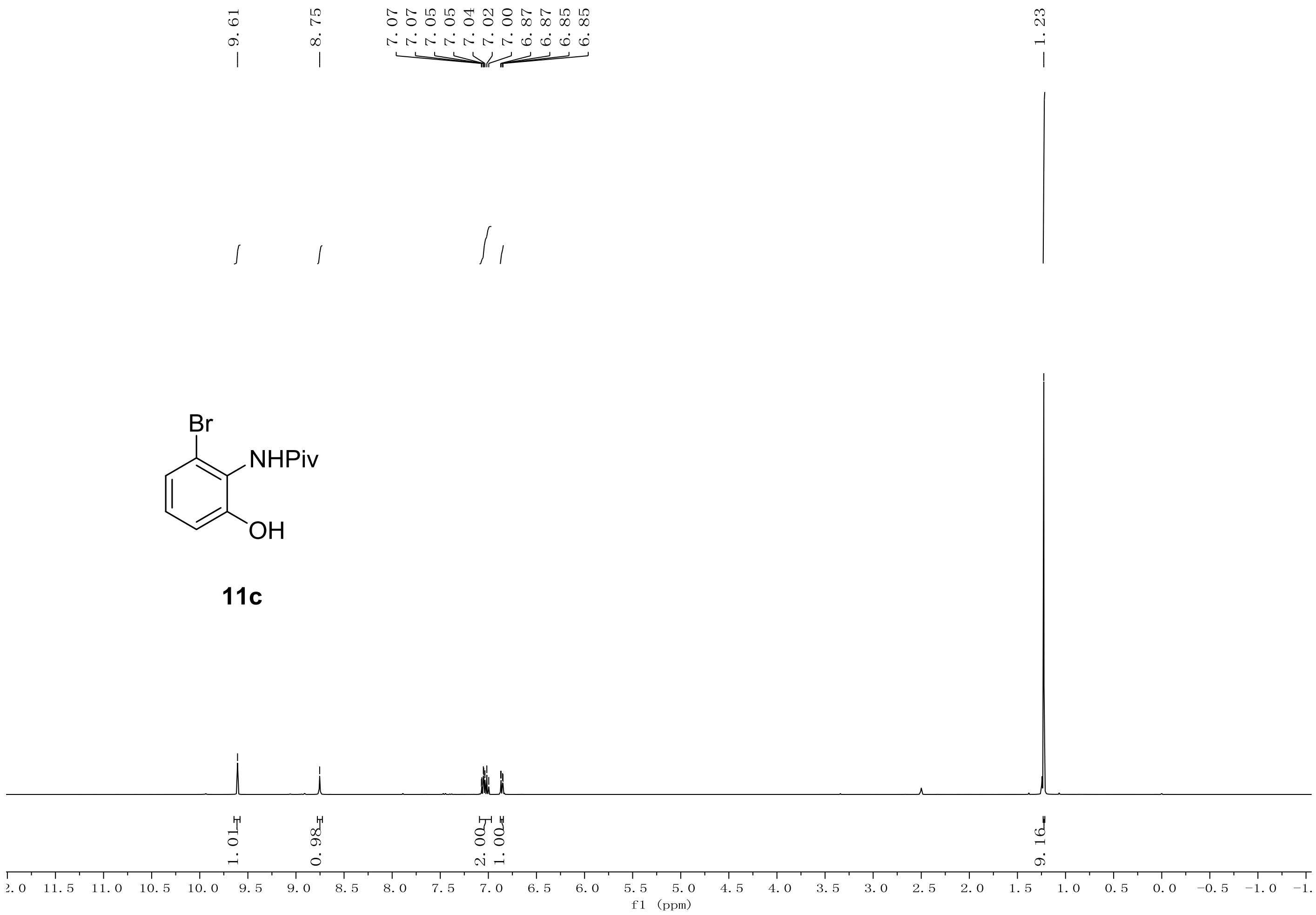

Supplementary Figure 22. ^{1}H NMR spectrum for **11c**

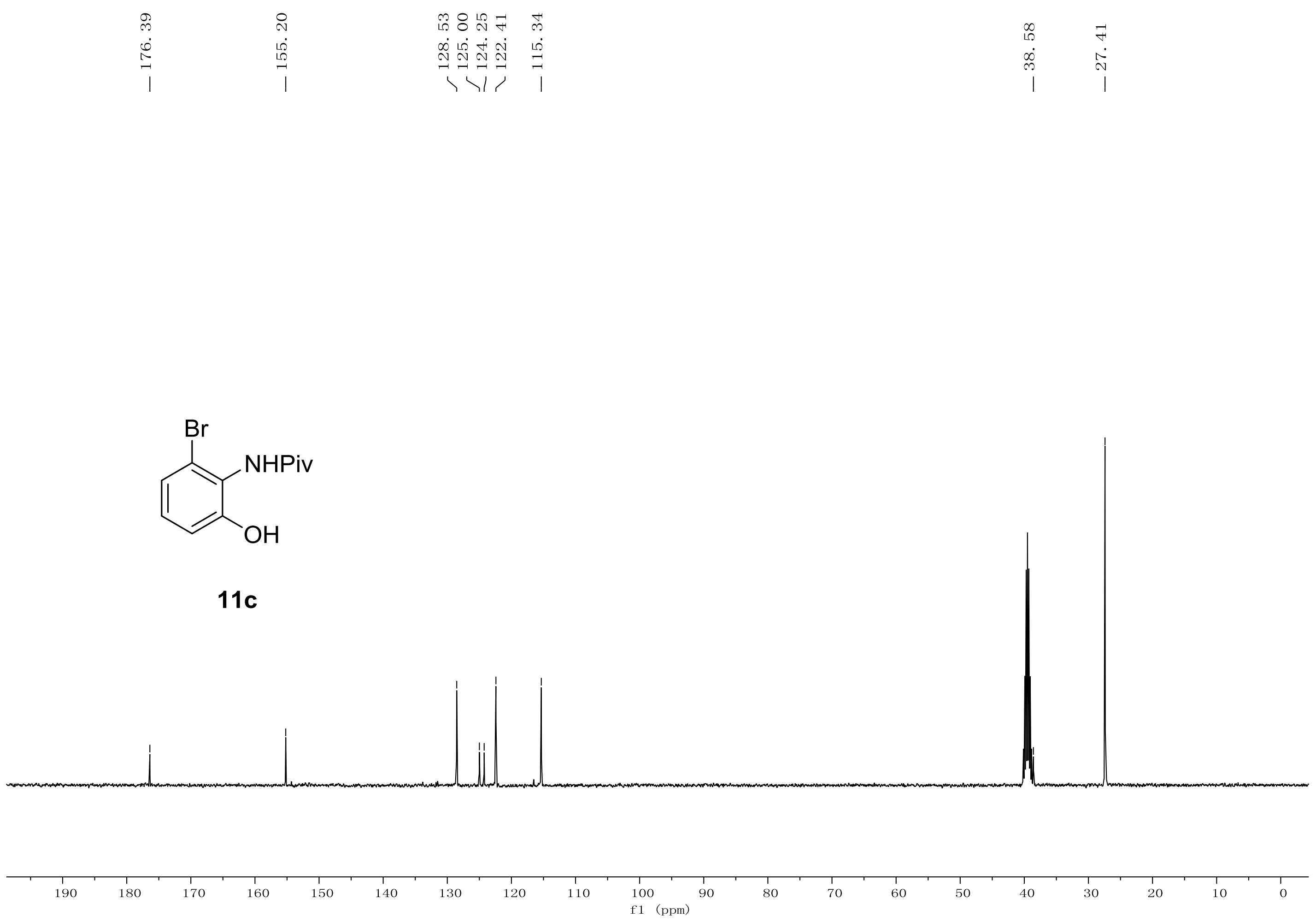

Supplementary Figure 23. ^{13}C NMR spectrum for **11c**

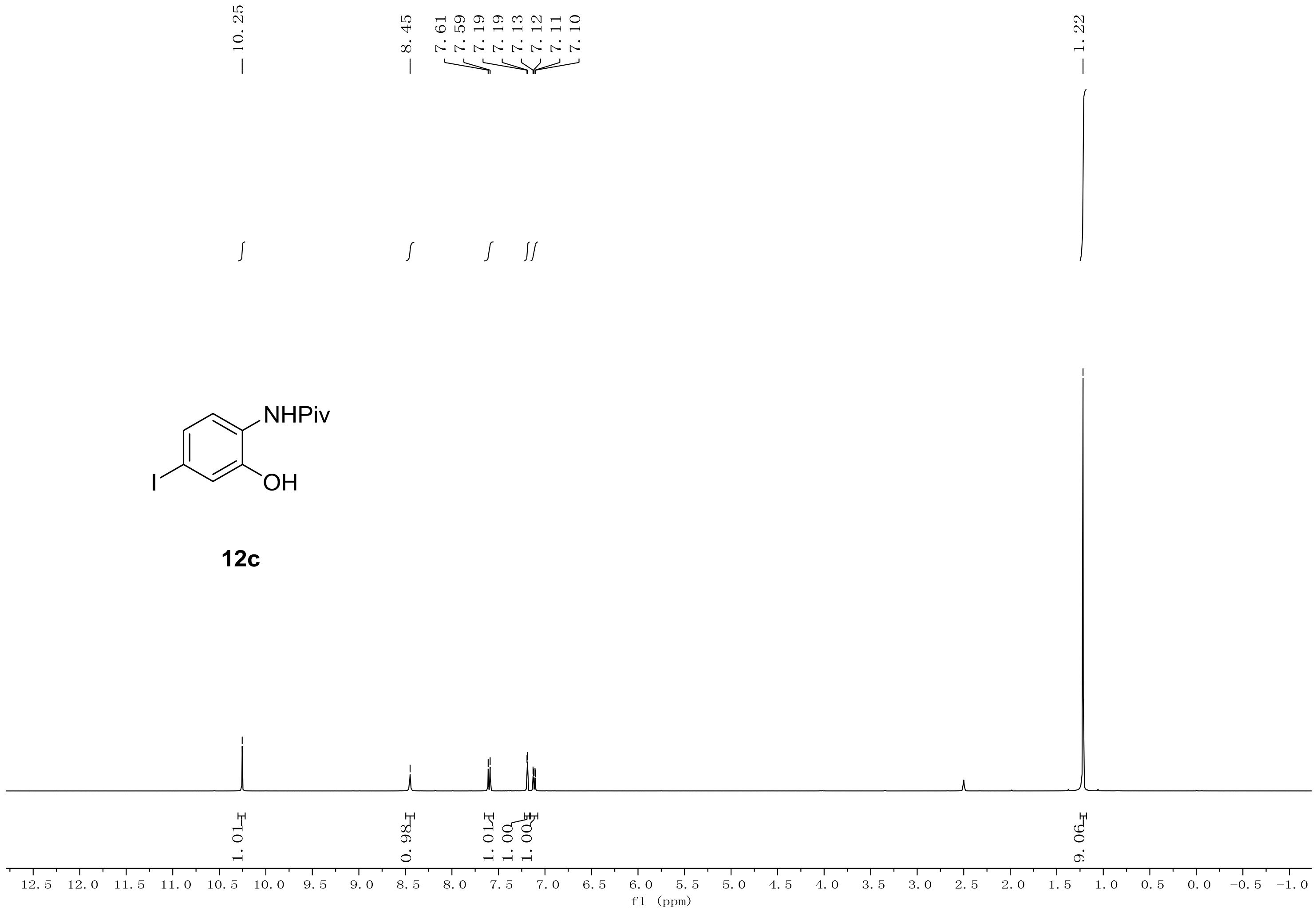

Supplementary Figure 24. ^{1}H NMR spectrum for **12c**

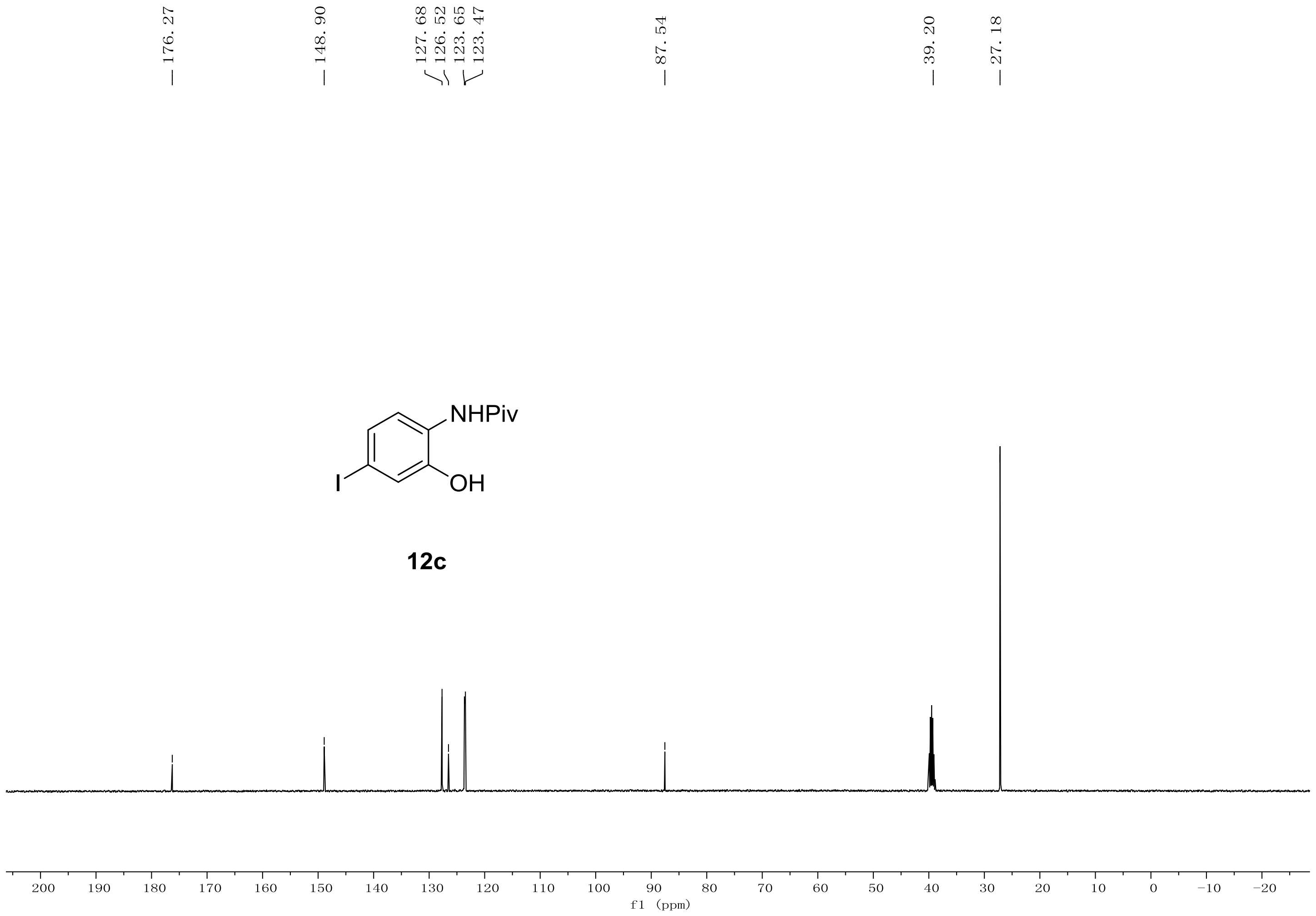

Supplementary Figure 25. ^{13}C NMR spectrum for **12c**

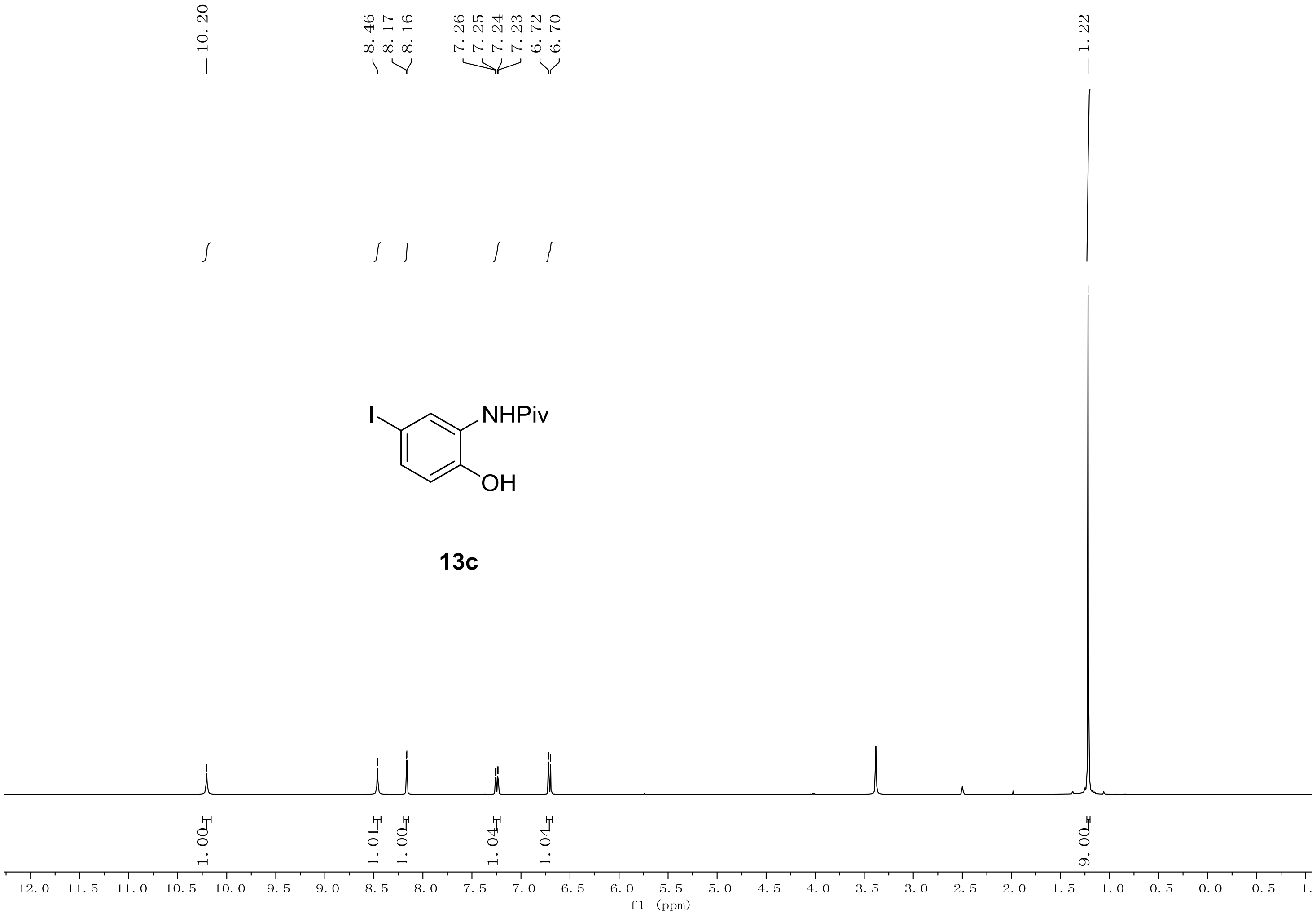

Supplementary Figure 26. ^{1}H NMR spectrum for **13c**

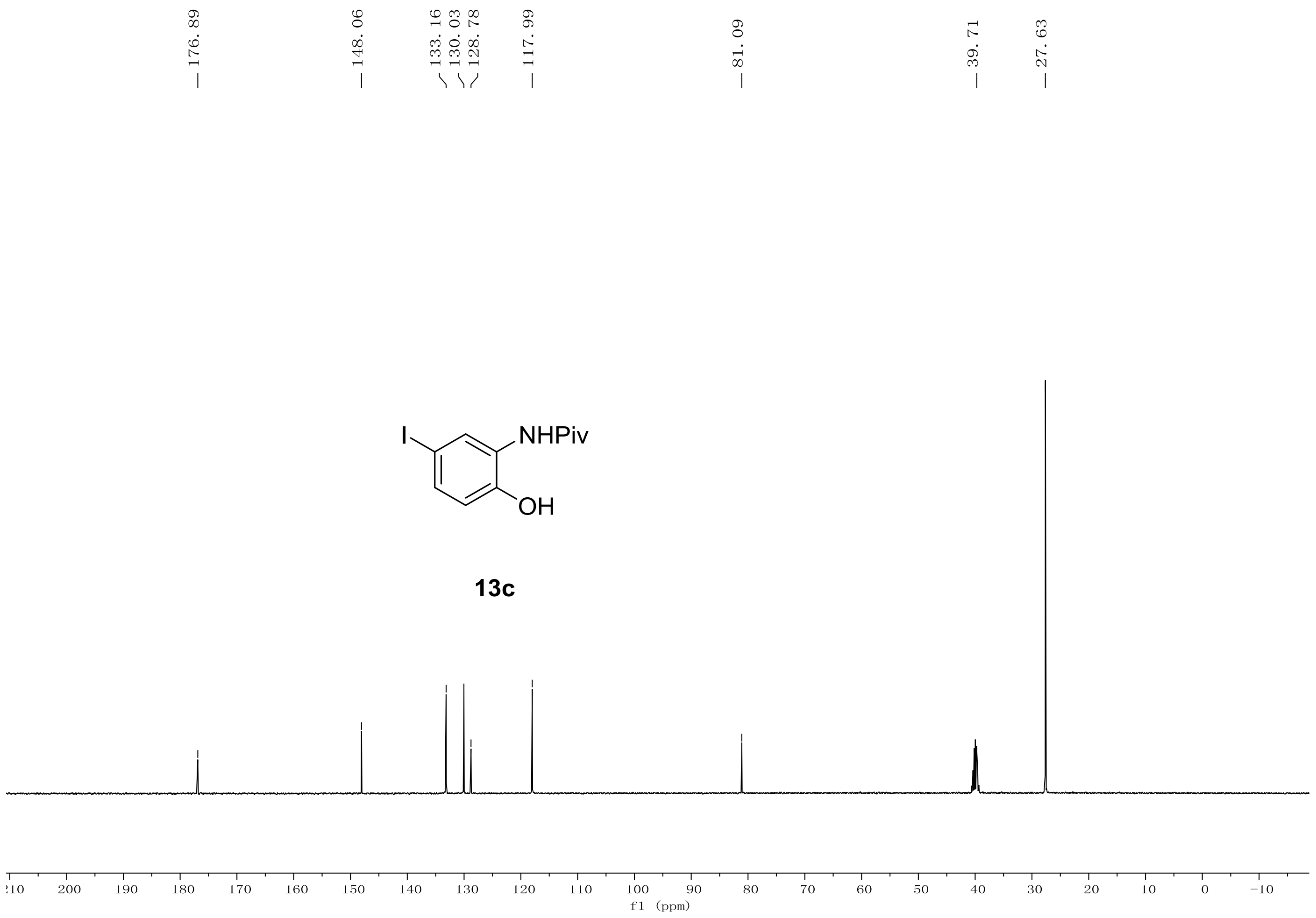

Supplementary Figure 27. ^{13}C NMR spectrum for **13c**

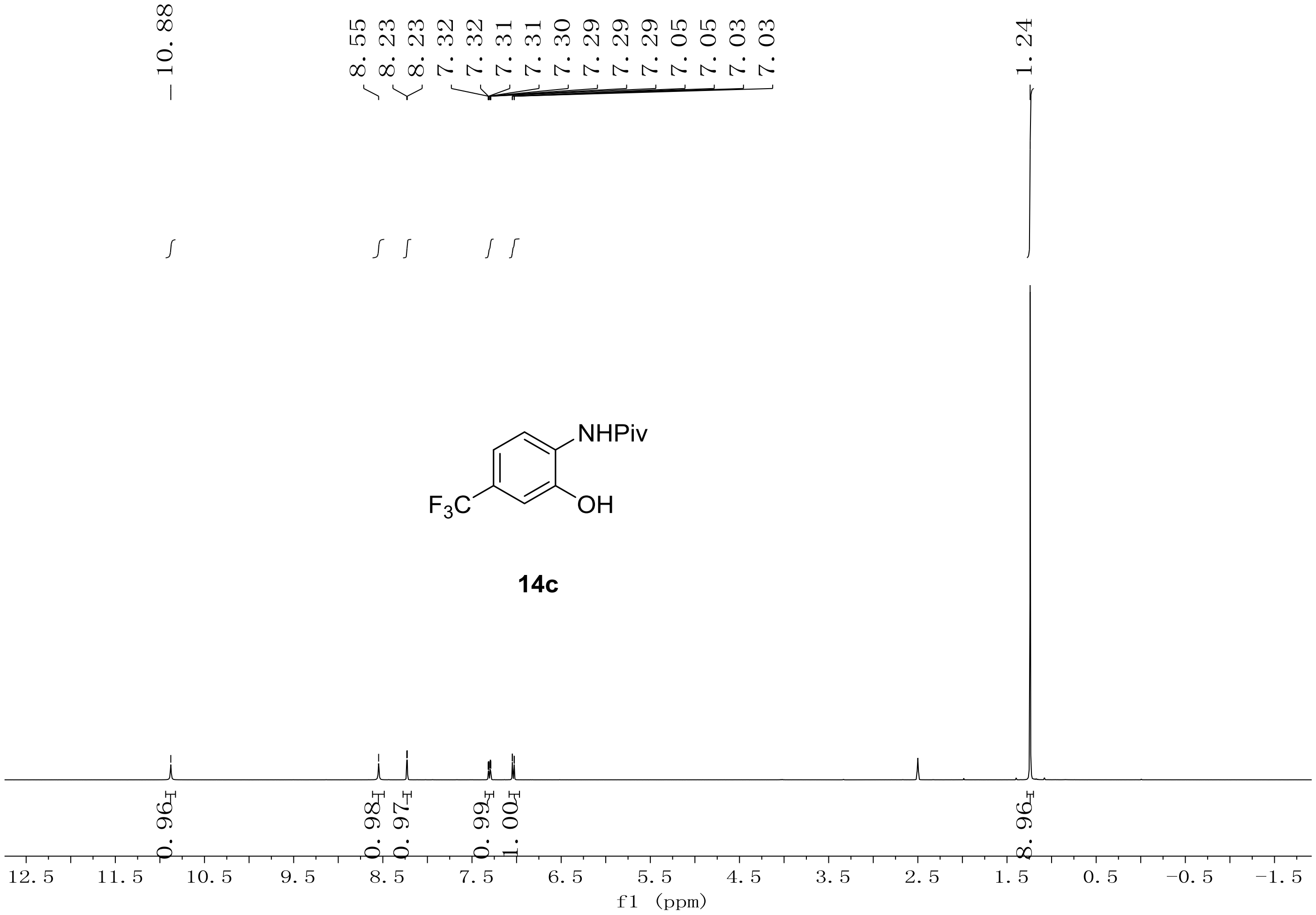

14c

Supplementary Figure 28. ^{1}H NMR spectrum for **14c**

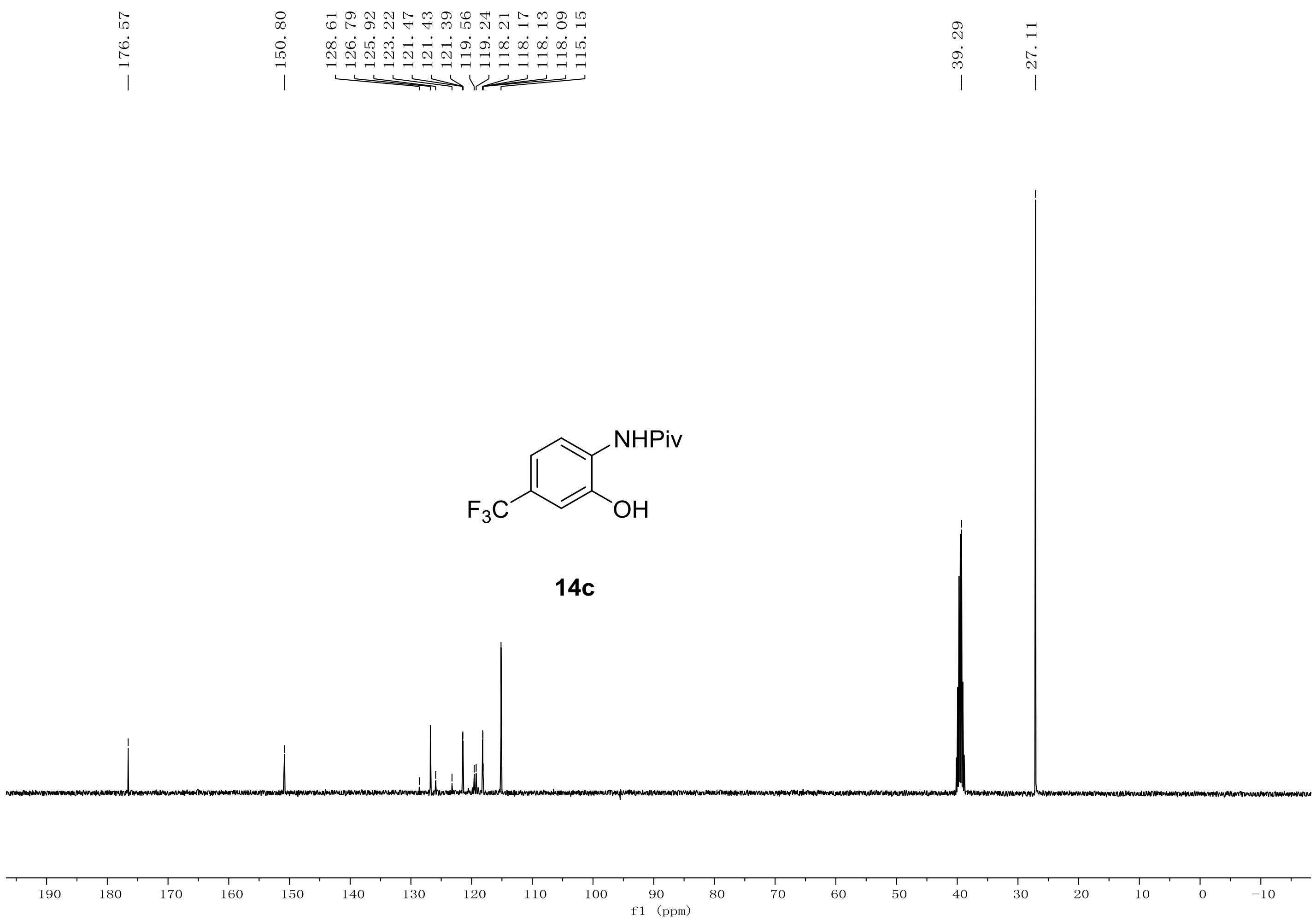

Supplementary Figure 29. ^{13}C NMR spectrum for **14c**

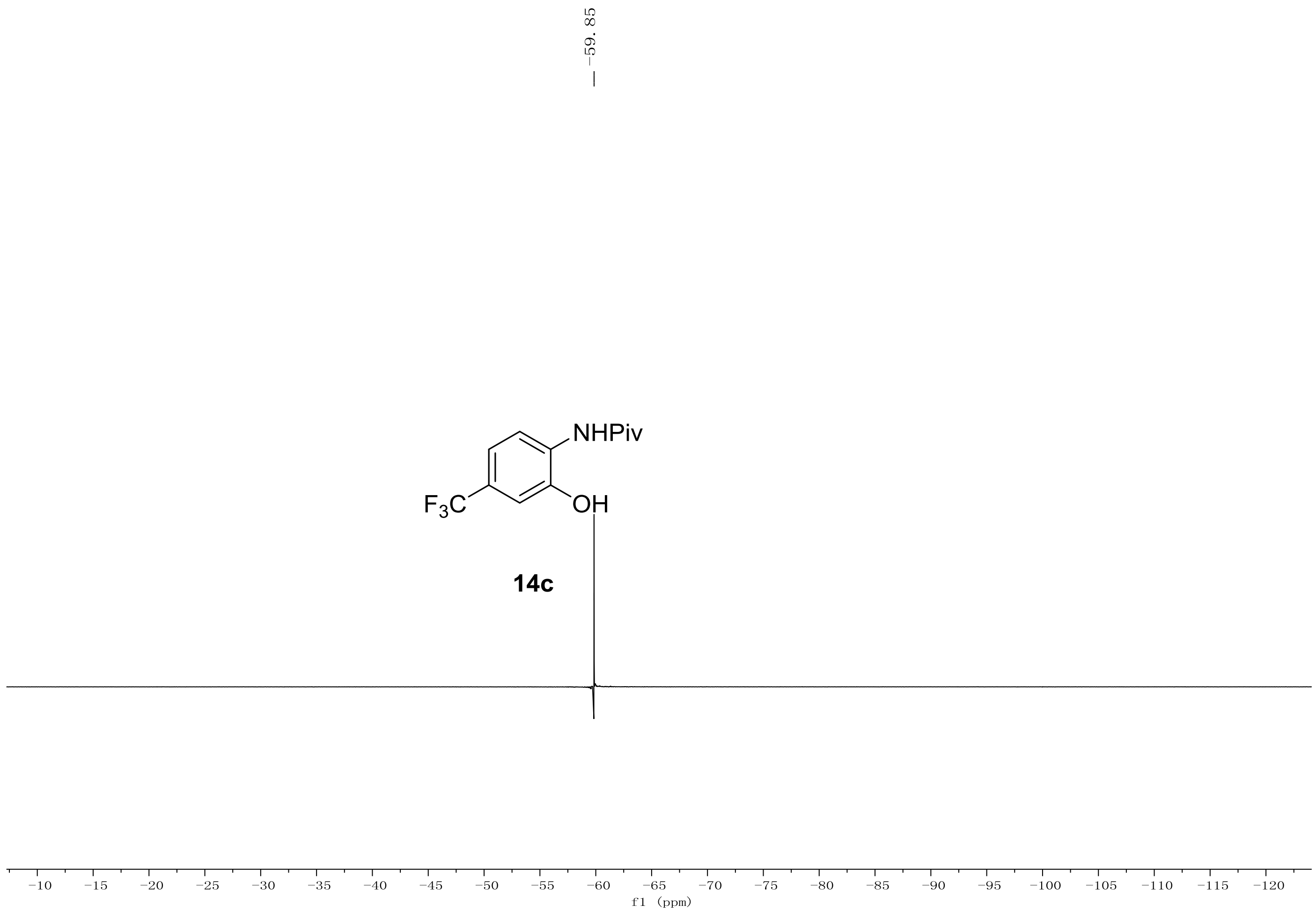

Supplementary Figure 30. ^{1}H NMR spectrum for **14c**

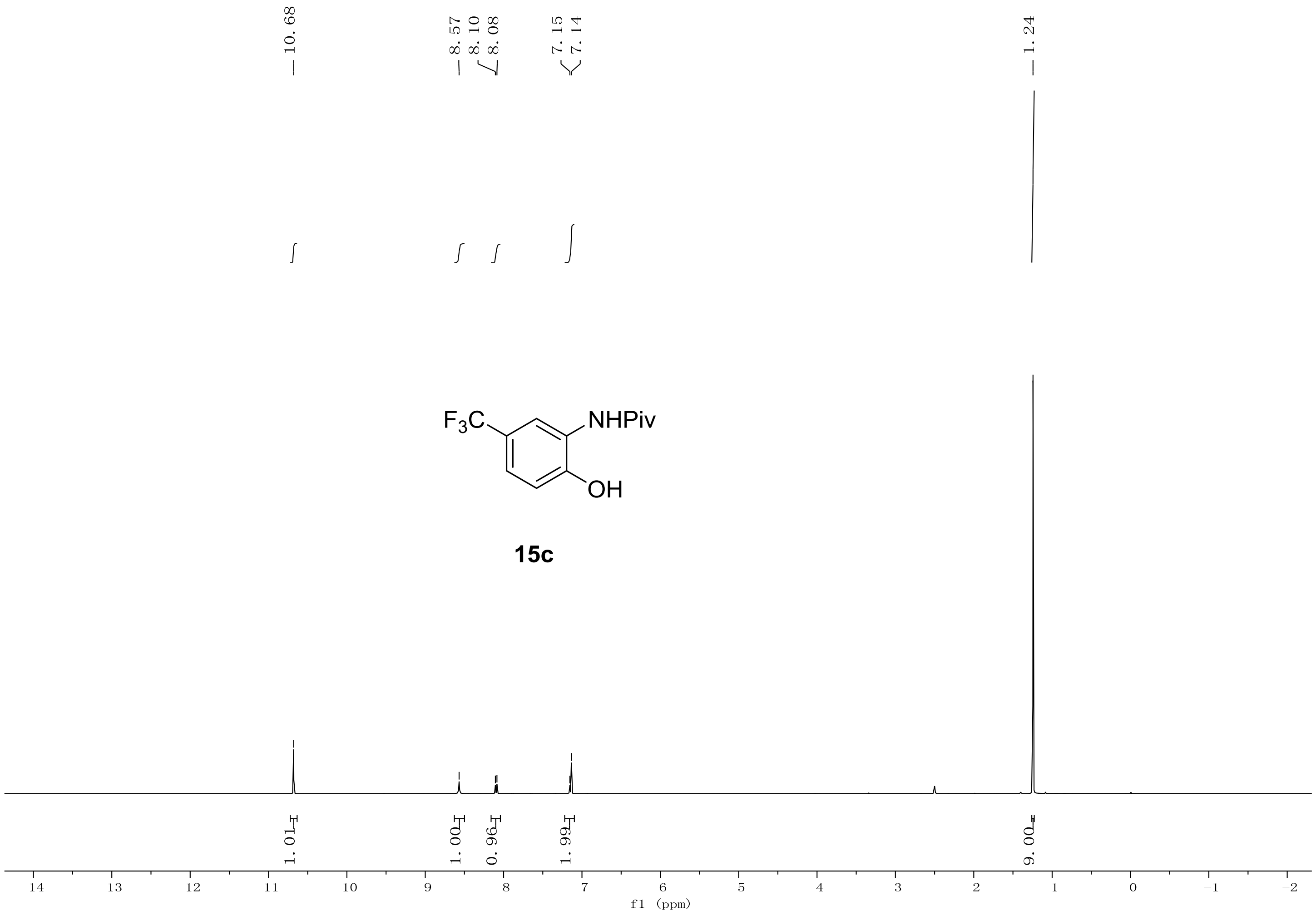

Supplementary Figure 31. ^{13}C NMR spectrum for **15c**

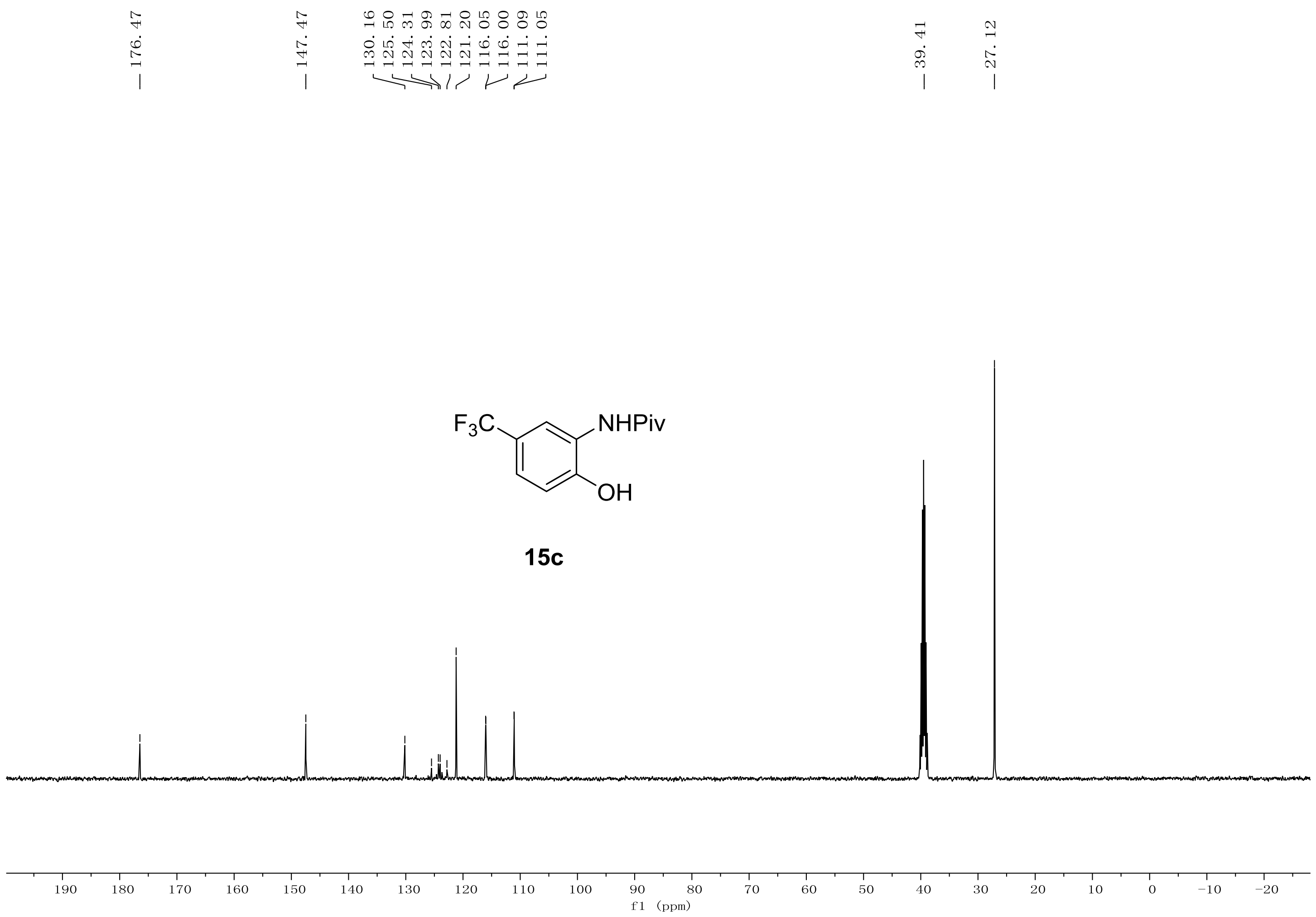

Supplementary Figure 32. ^{13}C NMR spectrum for **32c**

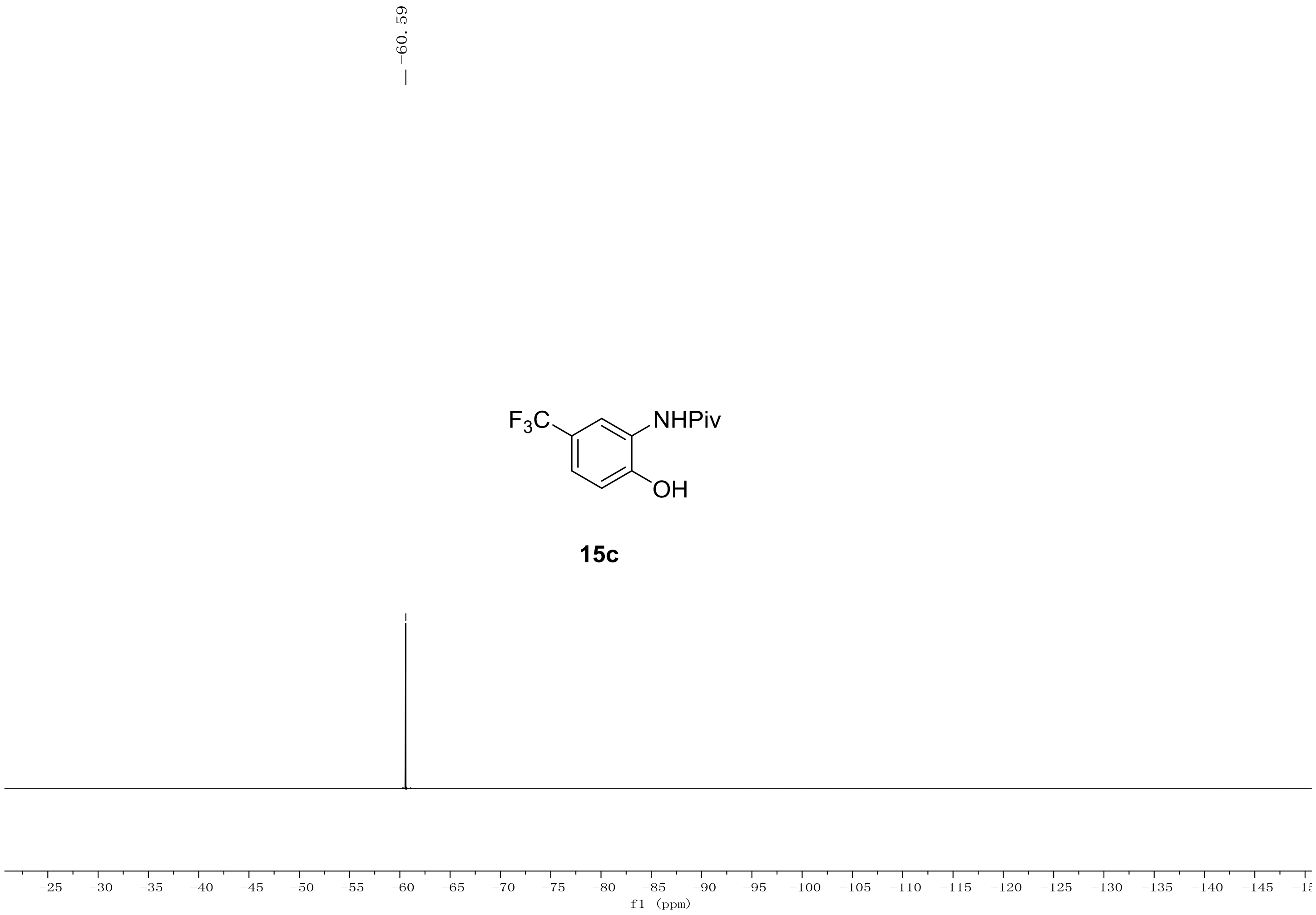

Supplementary Figure 33. ^{19}F NMR spectrum for **15c**

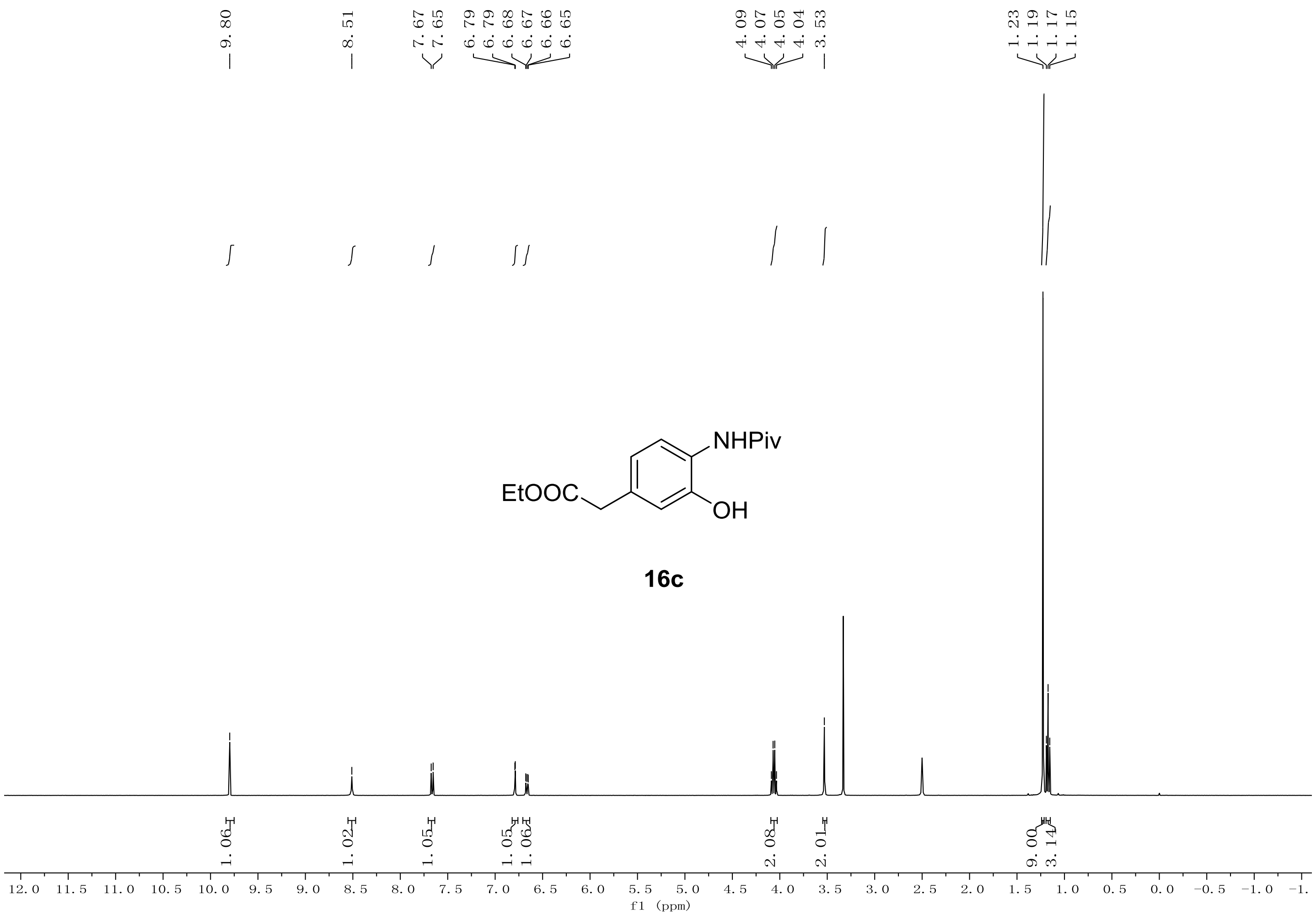

Supplementary Figure 34. ^{1}H NMR spectrum for **16c**

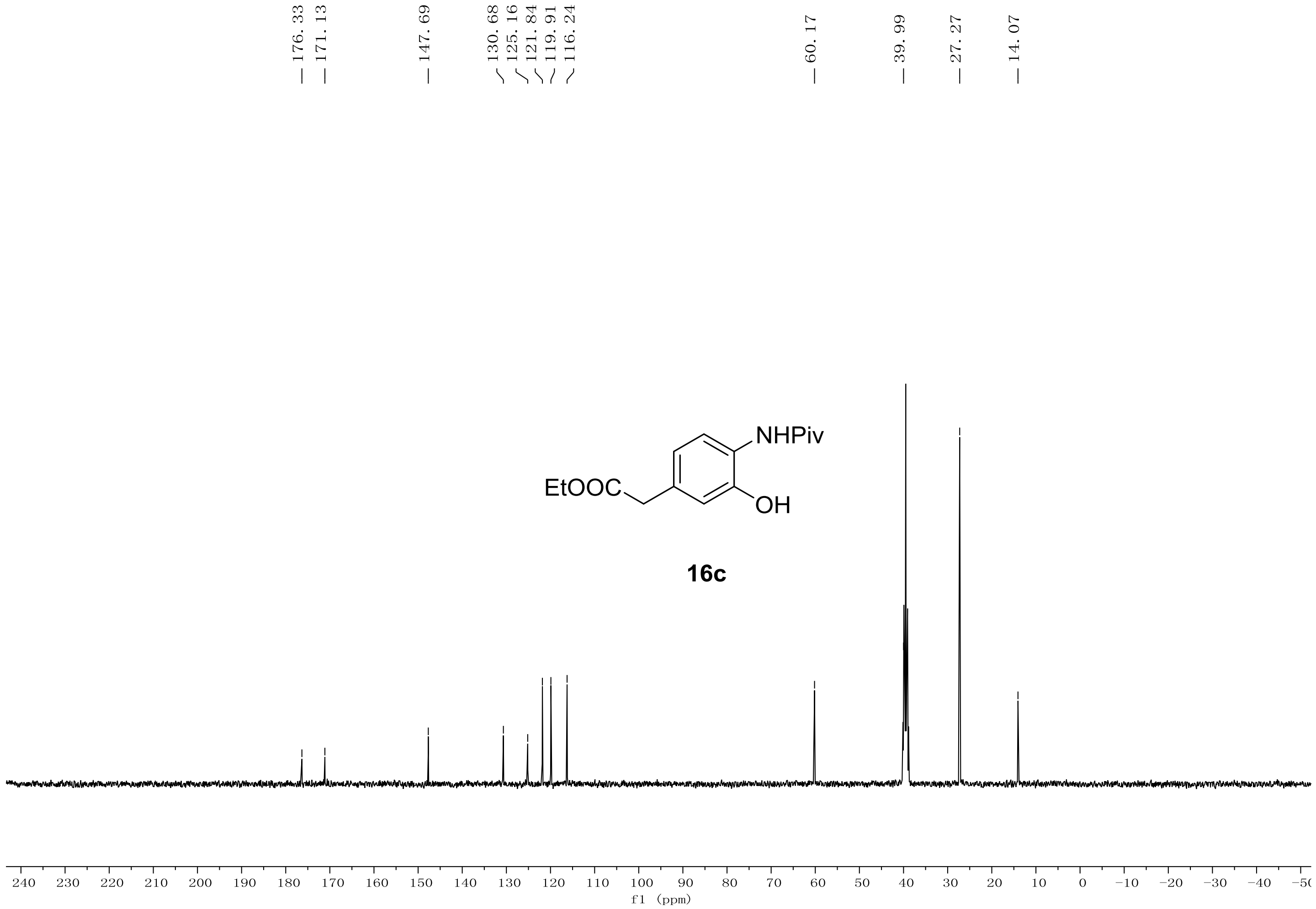

Supplementary Figure 35. ^{13}C NMR spectrum for **16c**

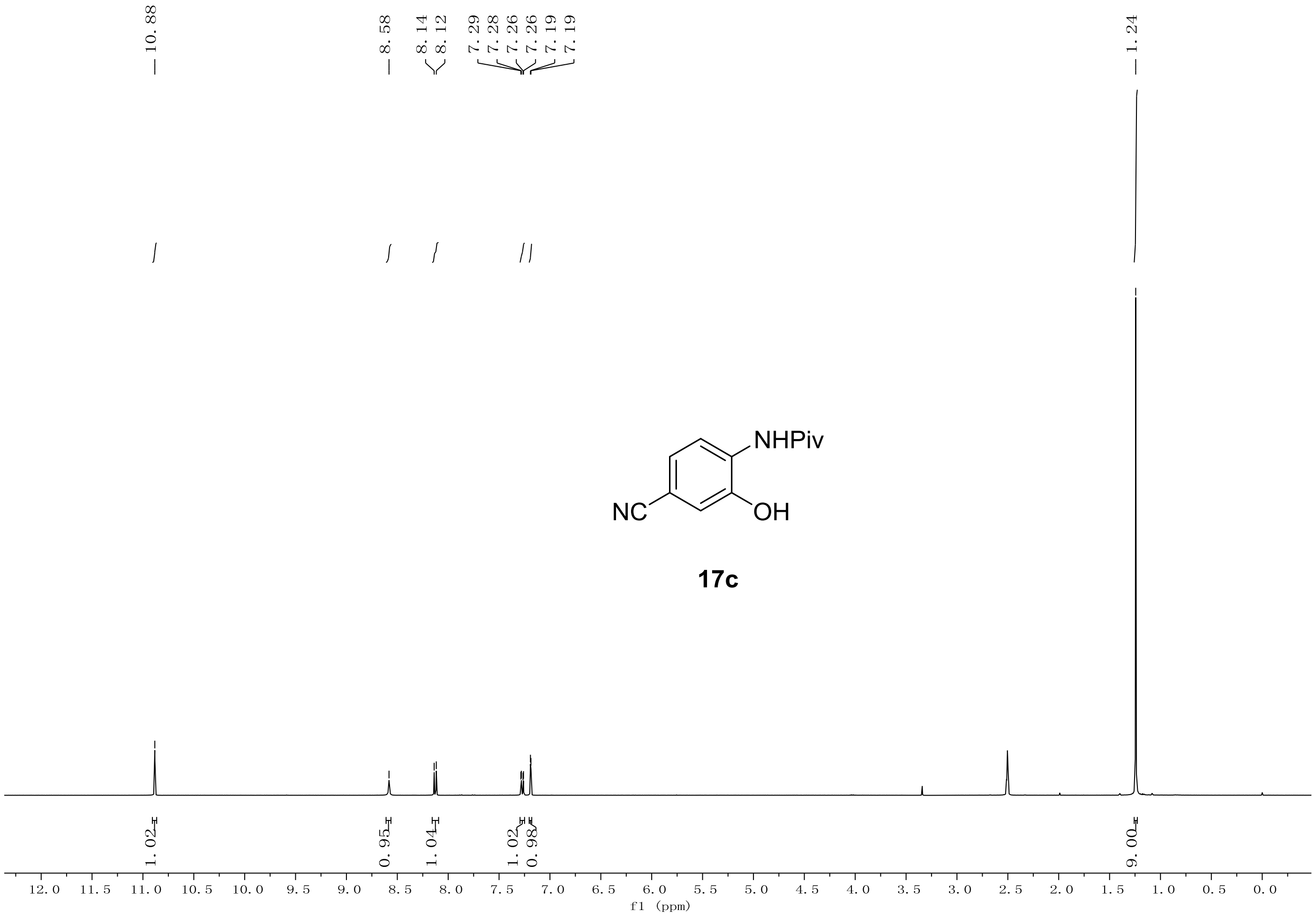

Supplementary Figure 36. ^{1}H NMR spectrum for **17c**

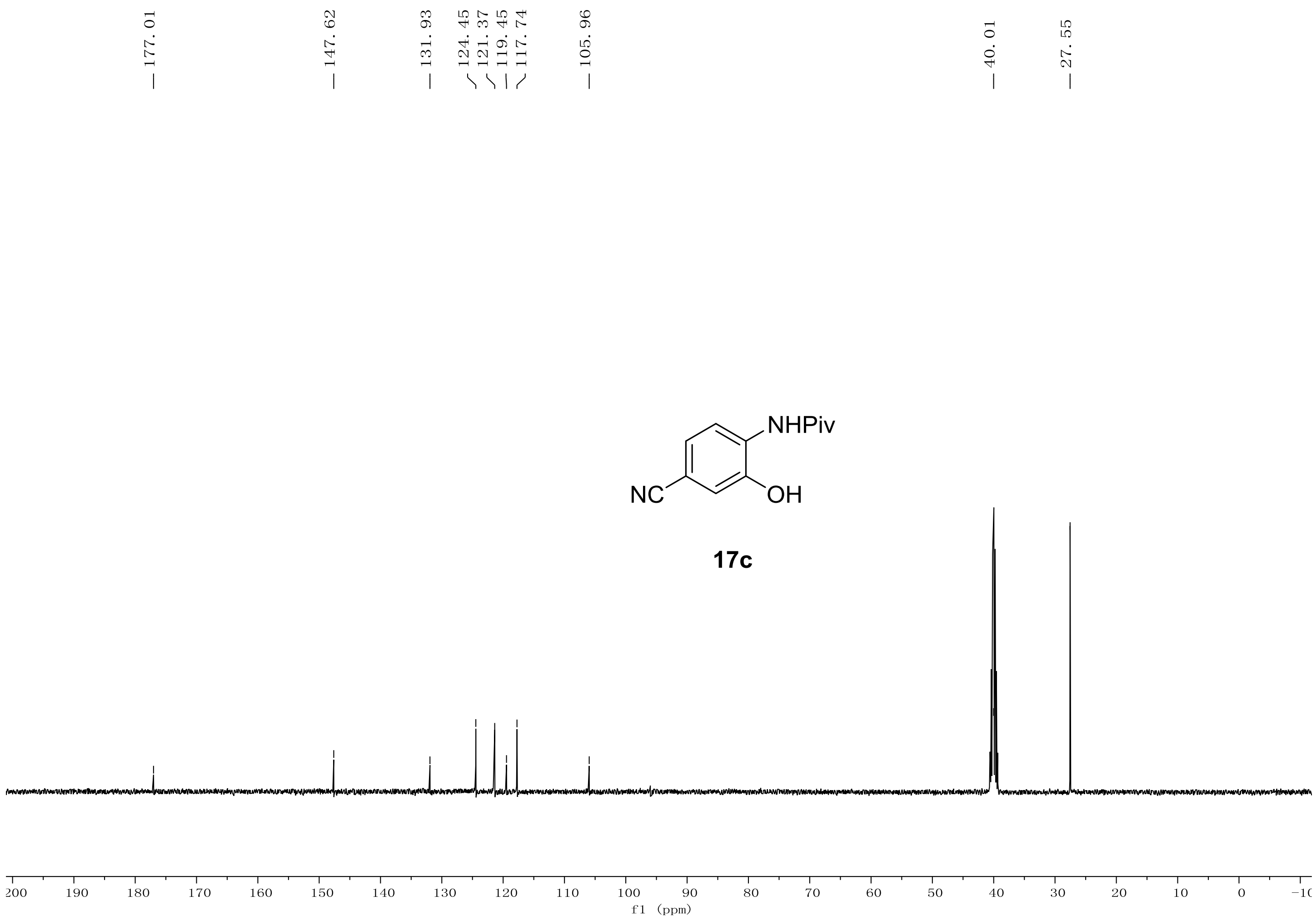

Supplementary Figure 37. ^{13}C NMR spectrum for **17c**

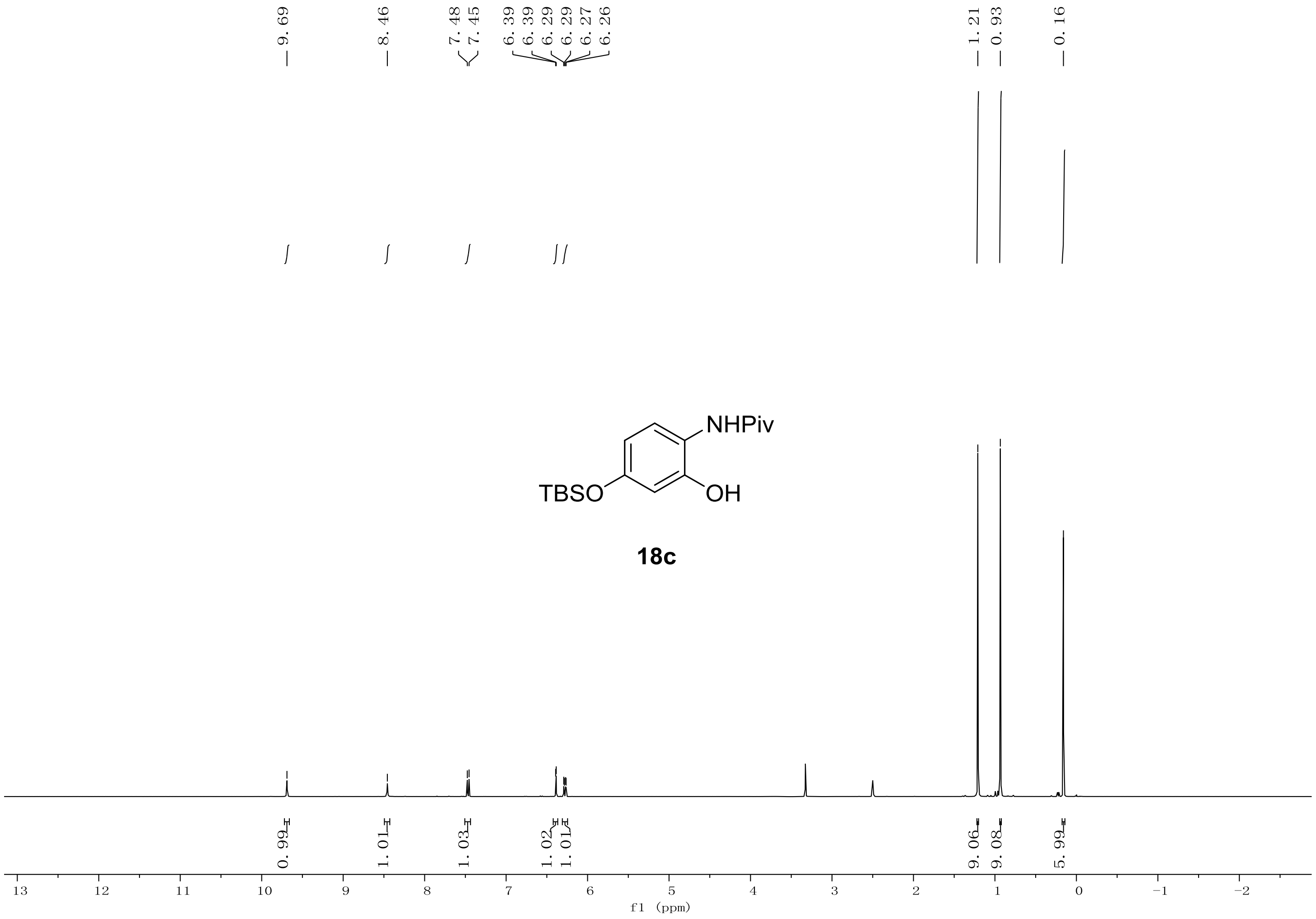

Supplementary Figure 38. ^{1}H NMR spectrum for **18c**

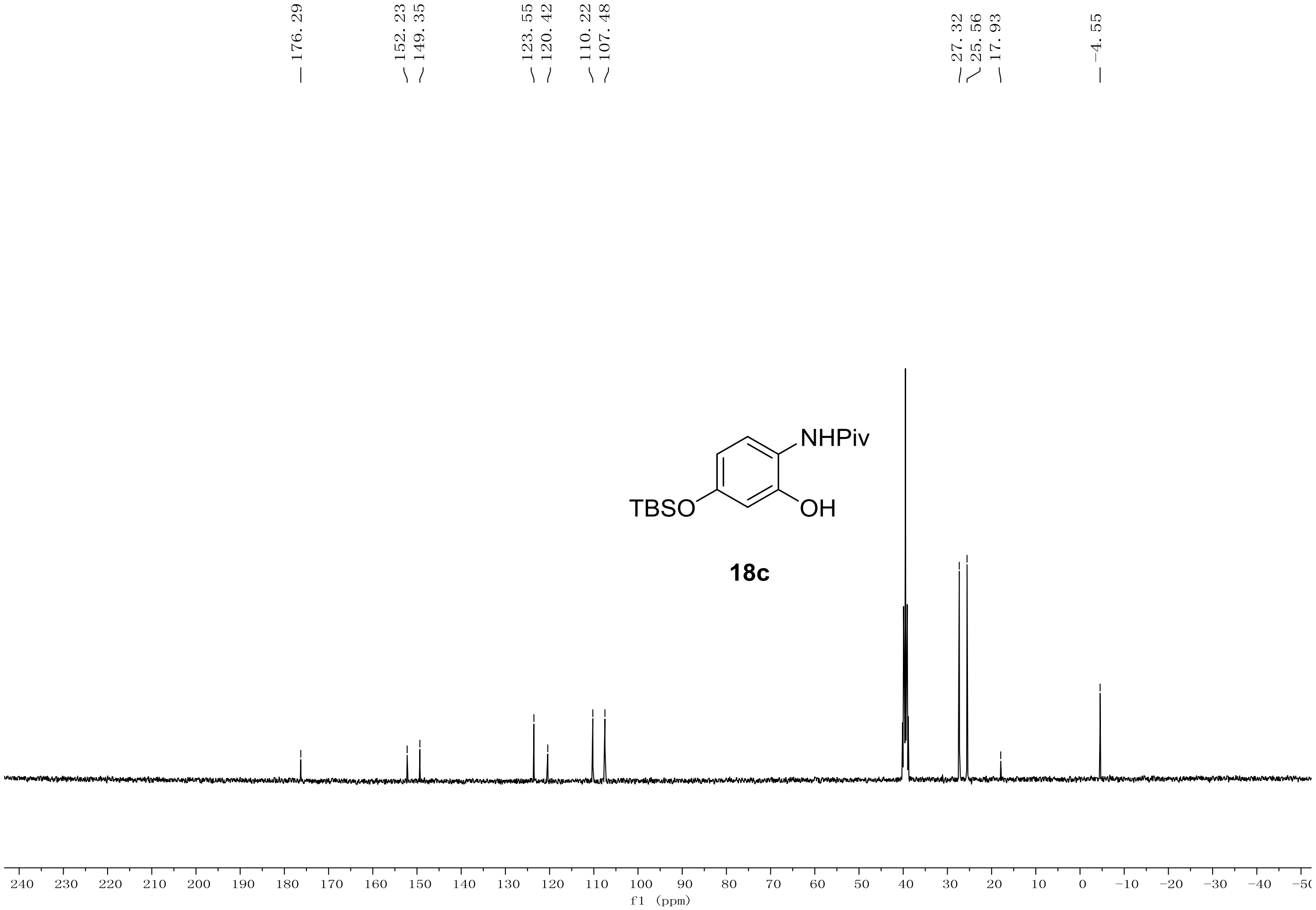

Supplementary Figure 39. ^{13}C NMR spectrum for **18c**

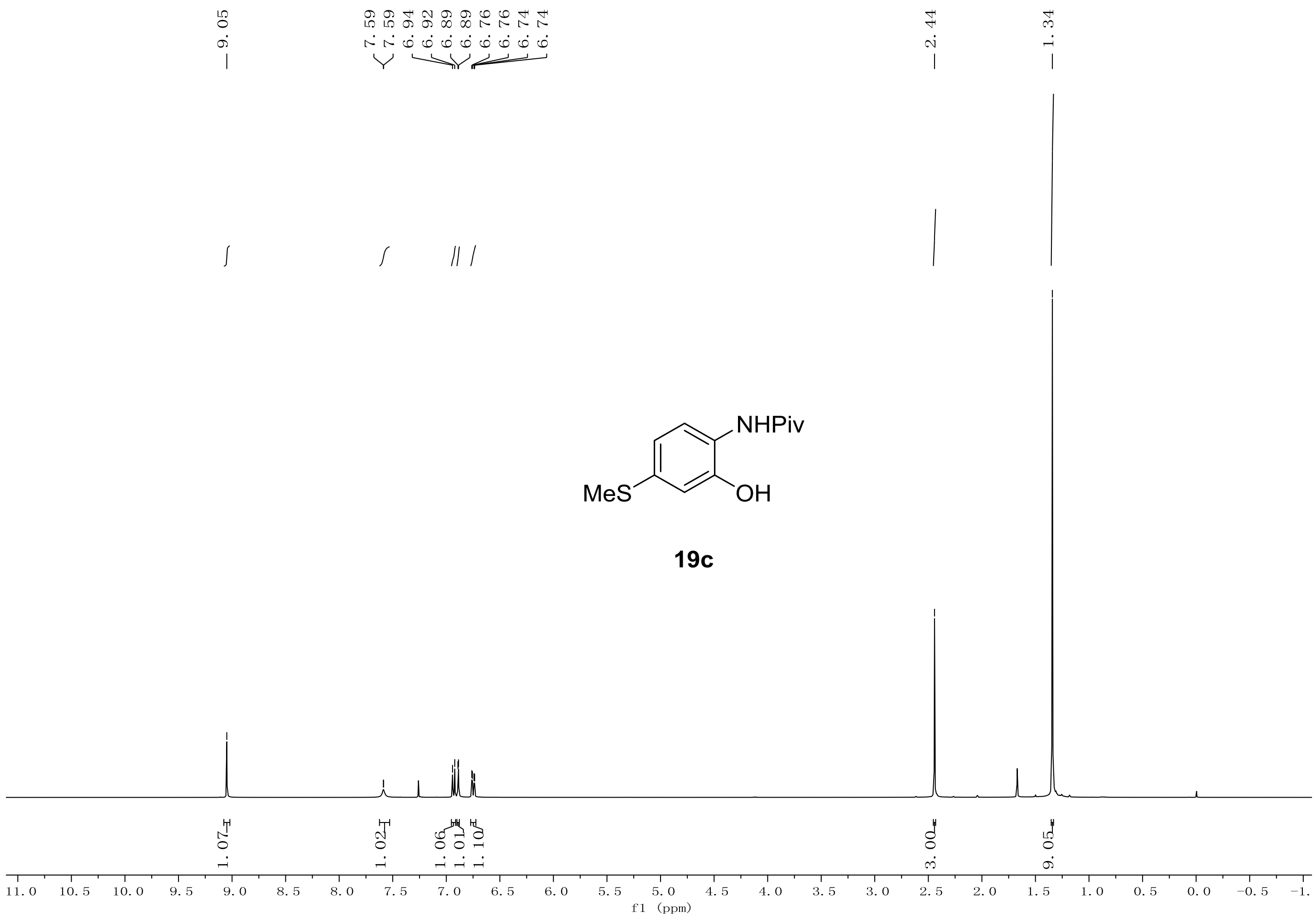

Supplementary Figure 40. ^{1}H NMR spectrum for **19c**

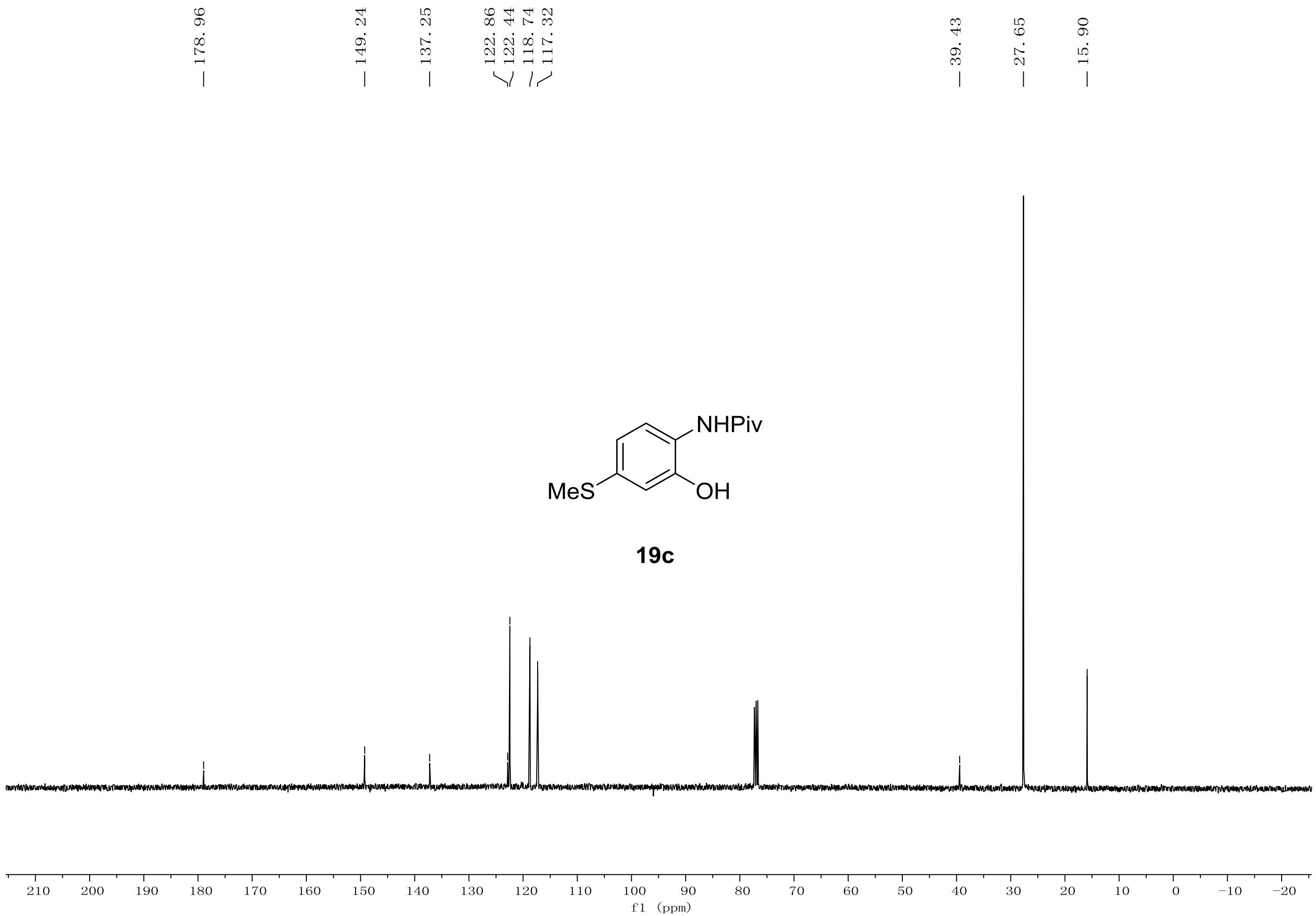

Supplementary Figure 41. ^{13}C NMR spectrum for **19c**

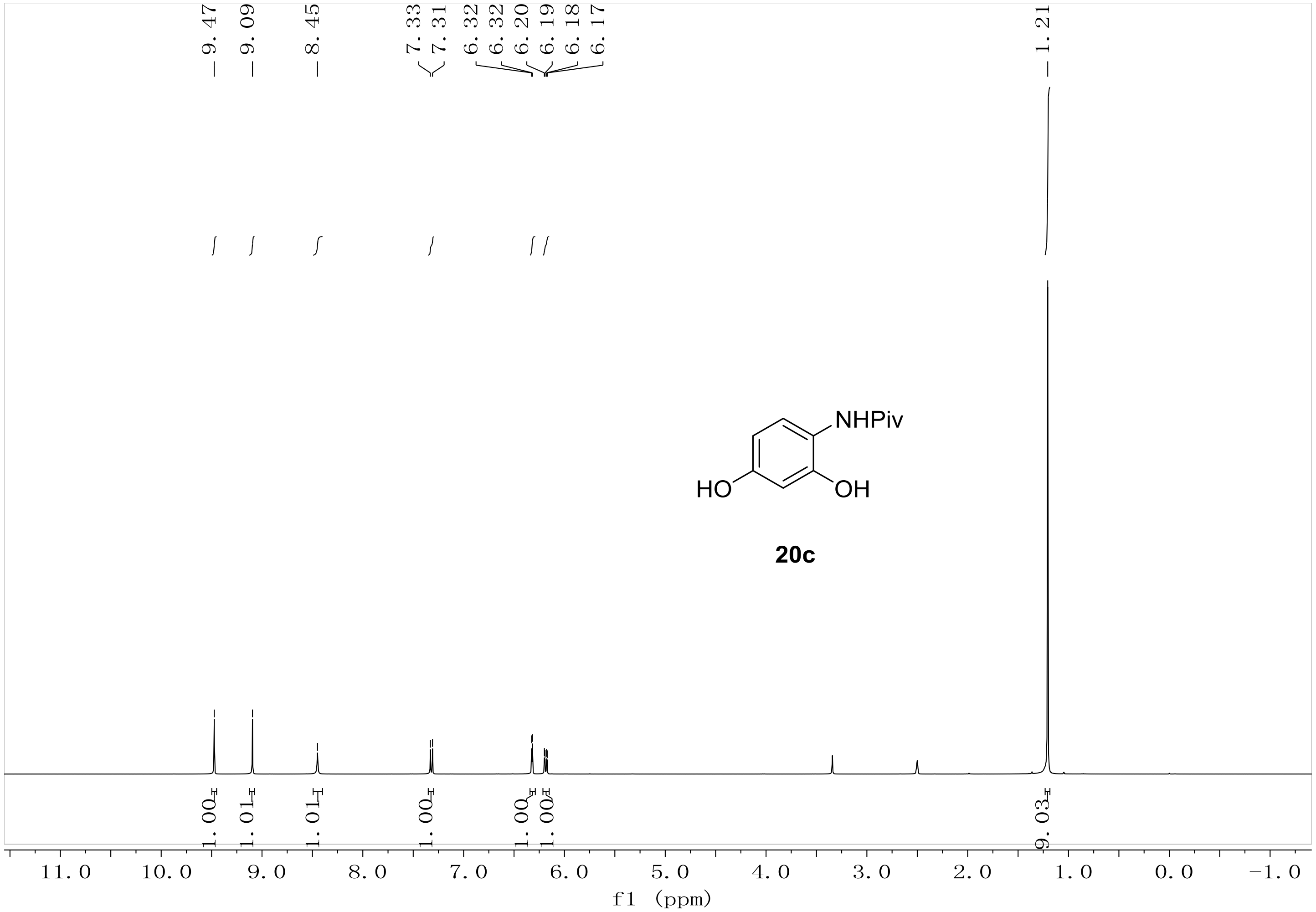

Supplementary Figure 42. ^{1}H NMR spectrum for **20c**

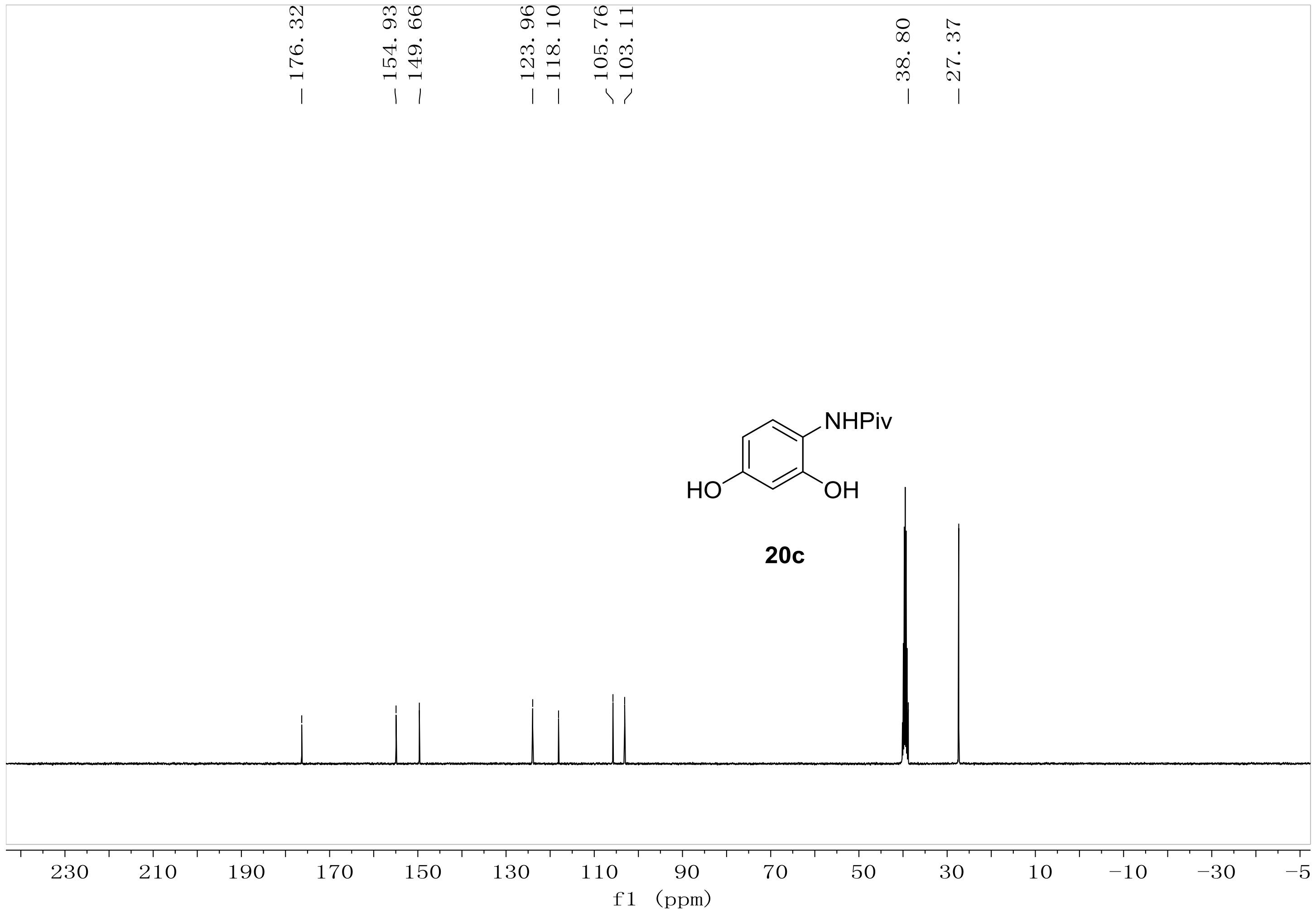

Supplementary Figure 43. ^{13}C NMR spectrum for **20c**

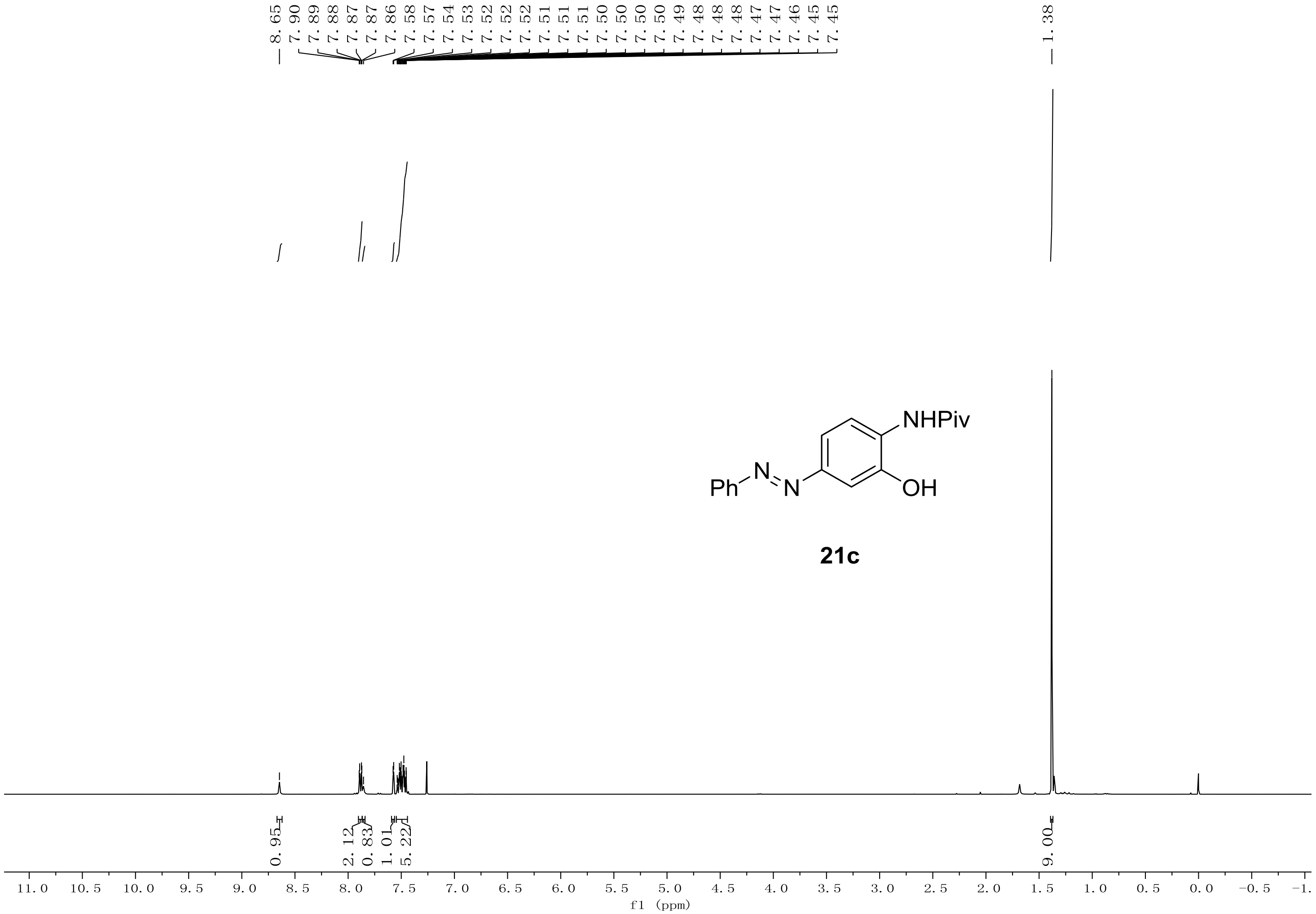

Supplementary Figure 44. ^{1}H NMR spectrum for **21c**

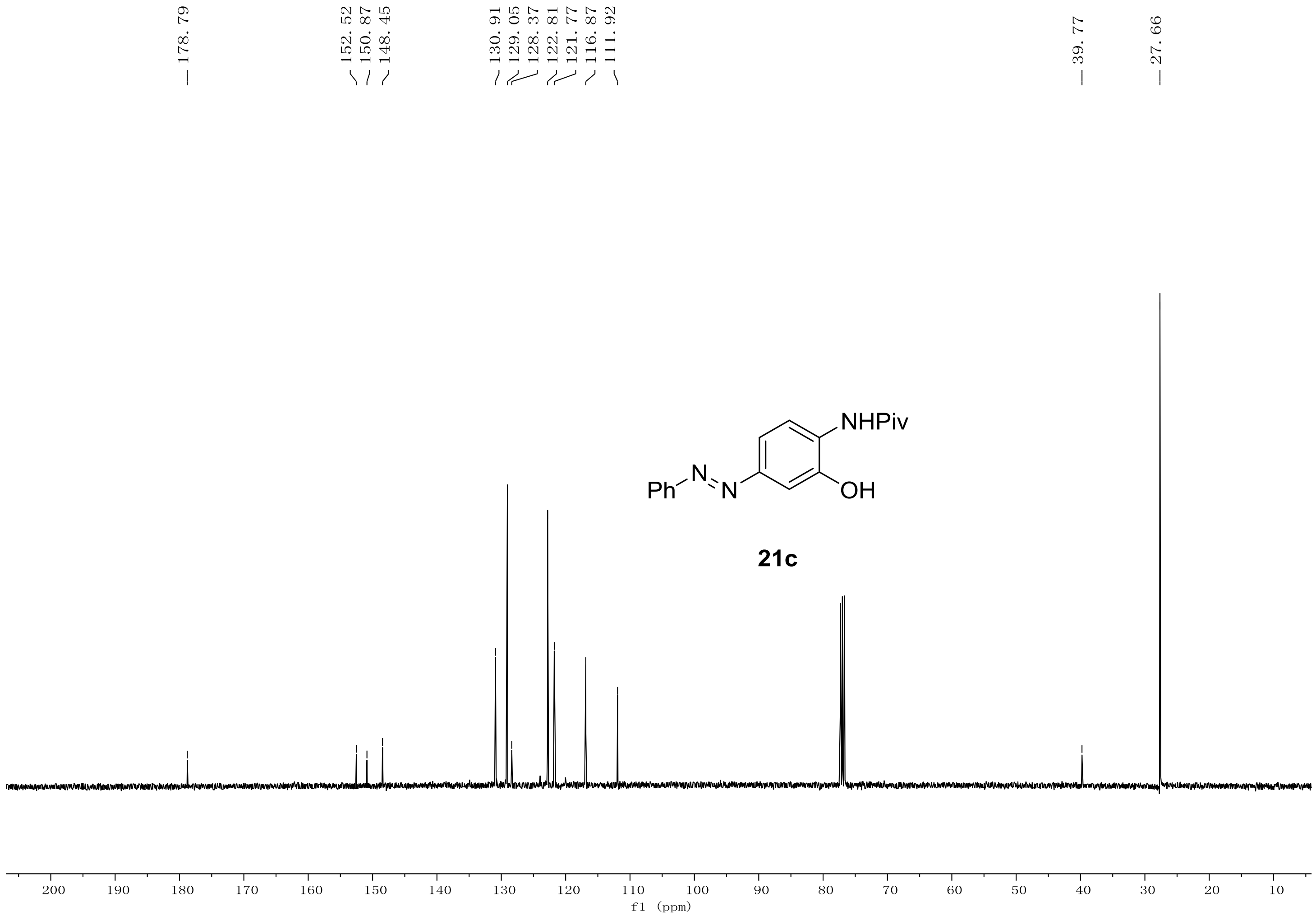

Supplementary Figure 45. ^{13}C NMR spectrum for **21c**

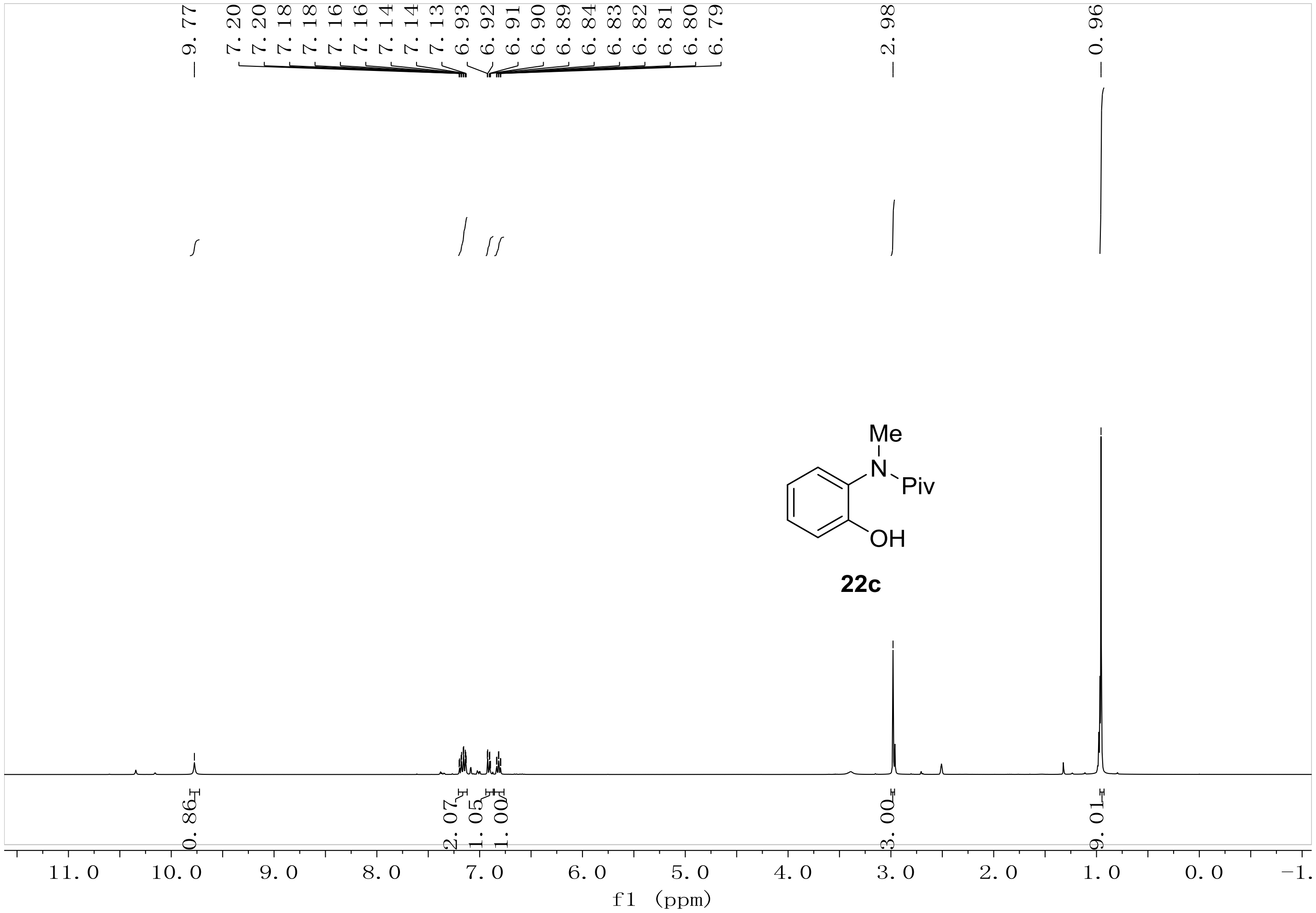

Supplementary Figure 46. ^{1}H NMR spectrum for **22c**

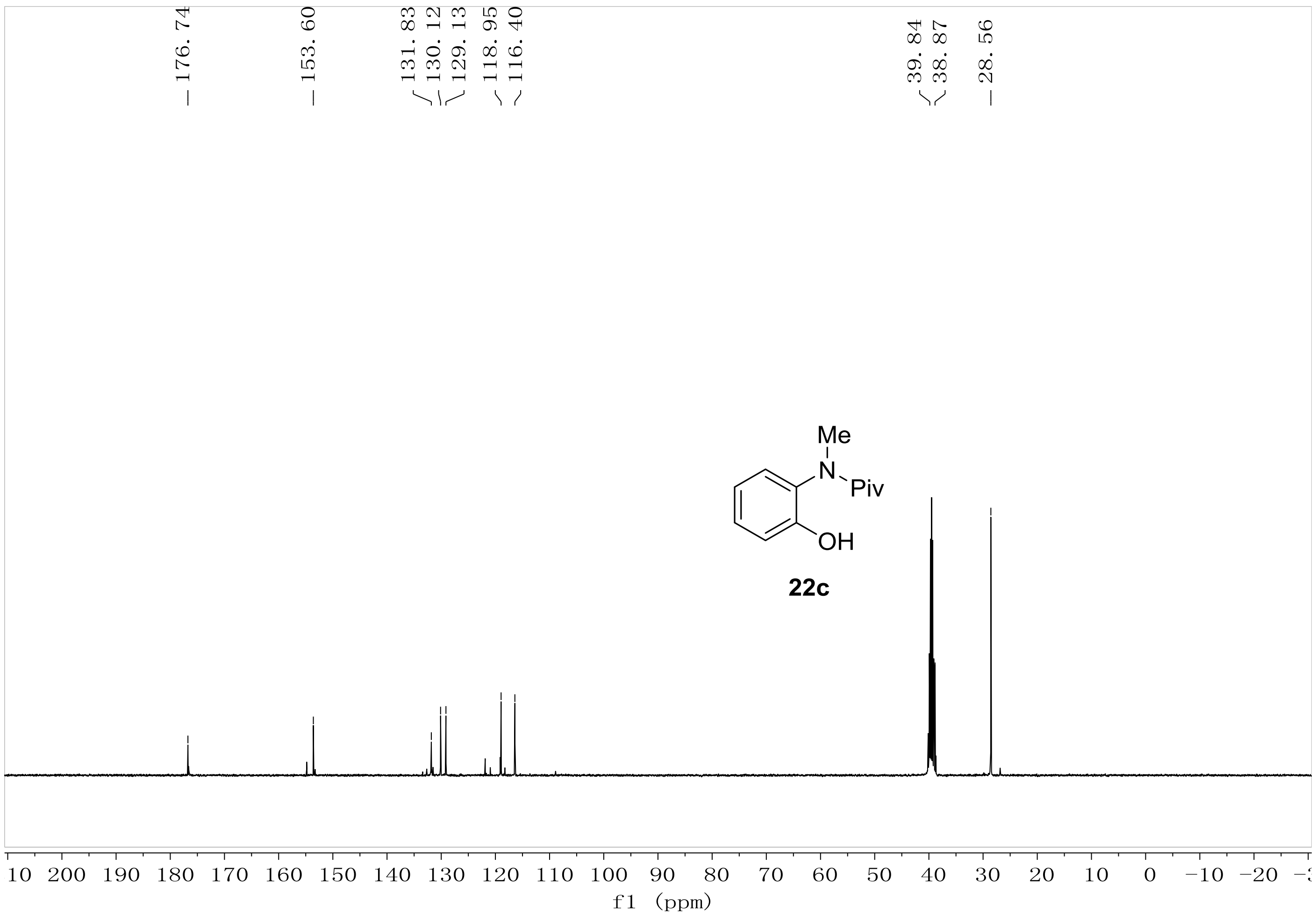

Supplementary Figure 47. ^{13}C NMR spectrum for **22c**

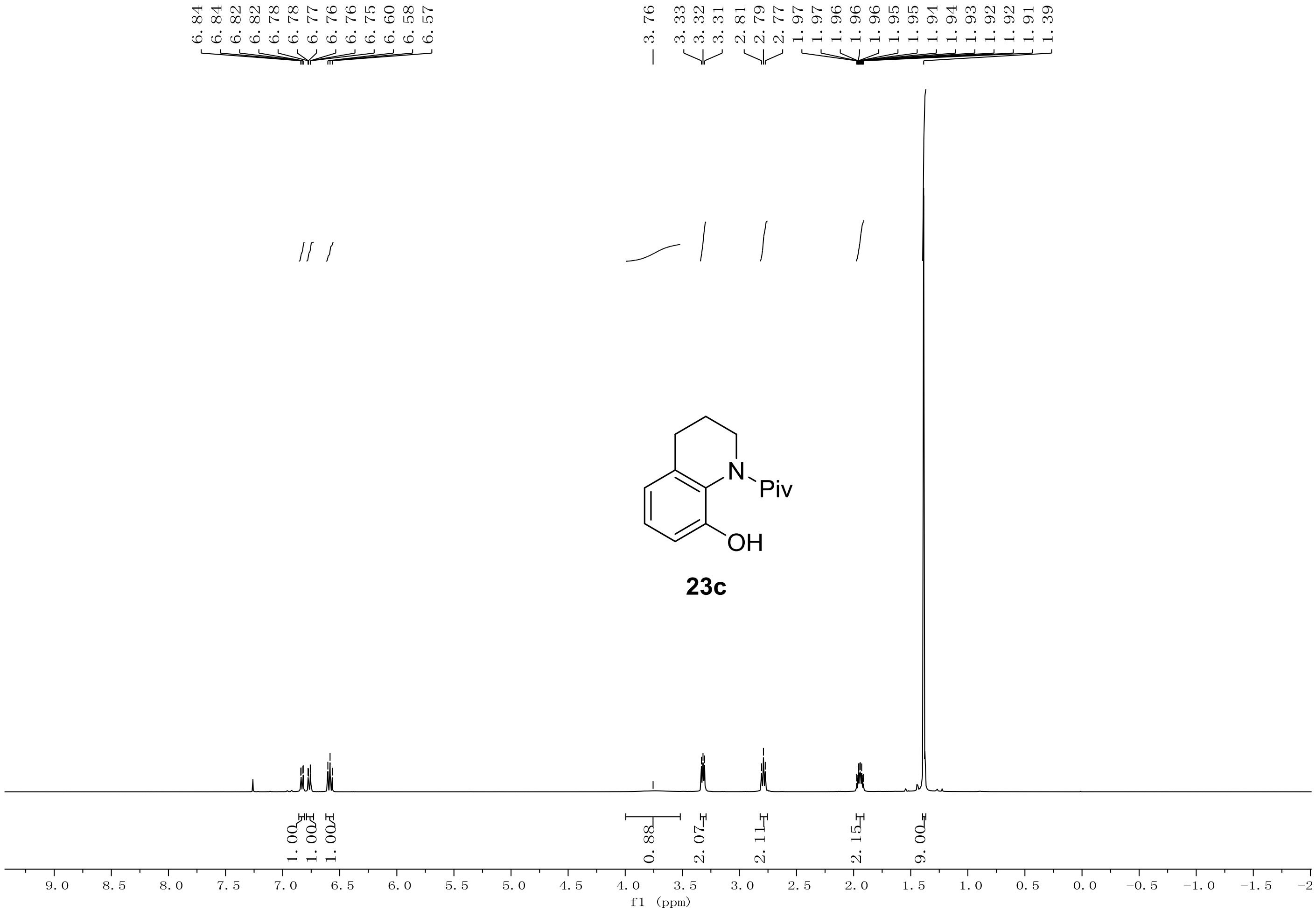

23c

Supplementary Figure 48. ^{1}H NMR spectrum for **23c**

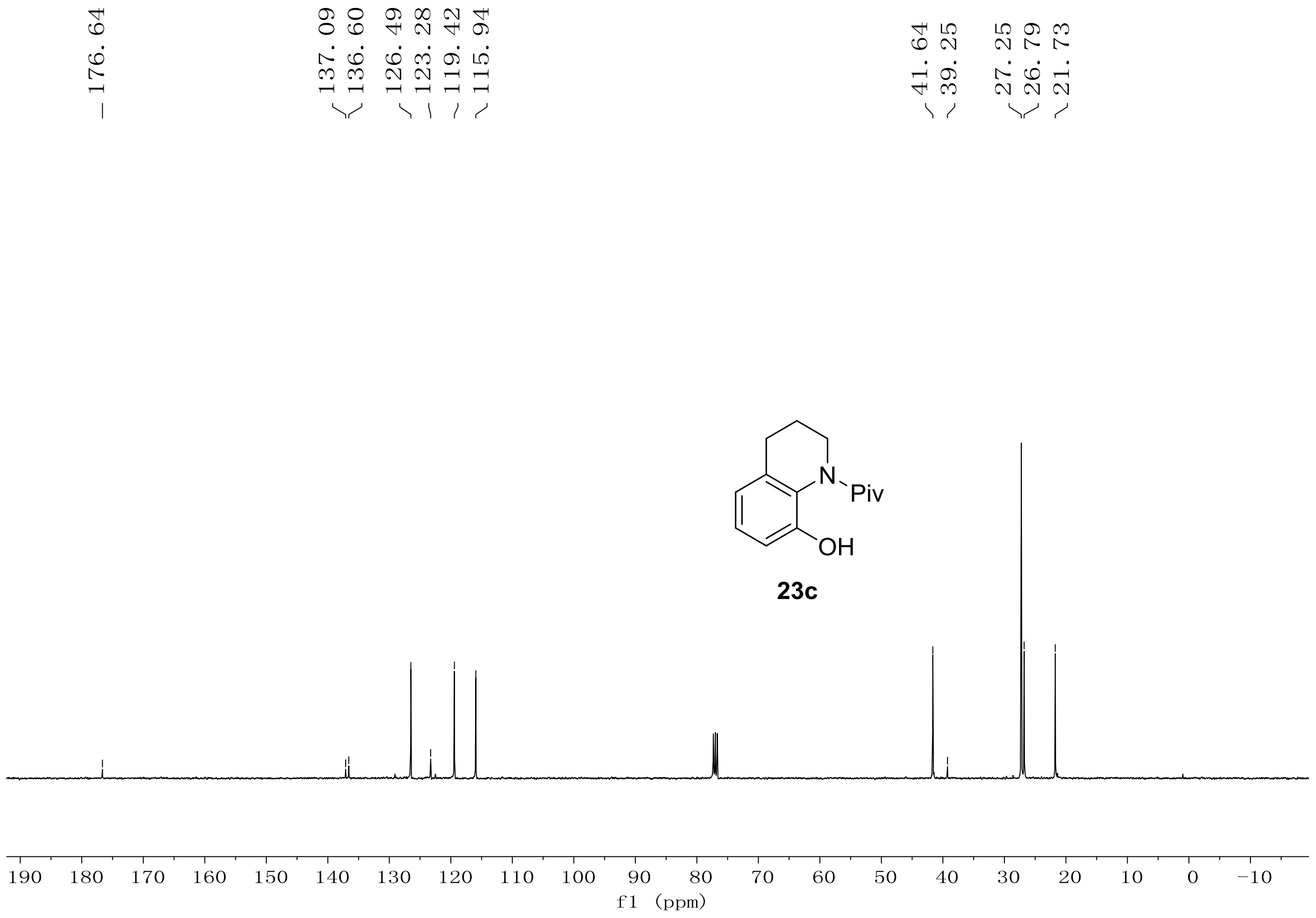

Supplementary Figure 49. ^{13}C NMR spectrum for **23c**

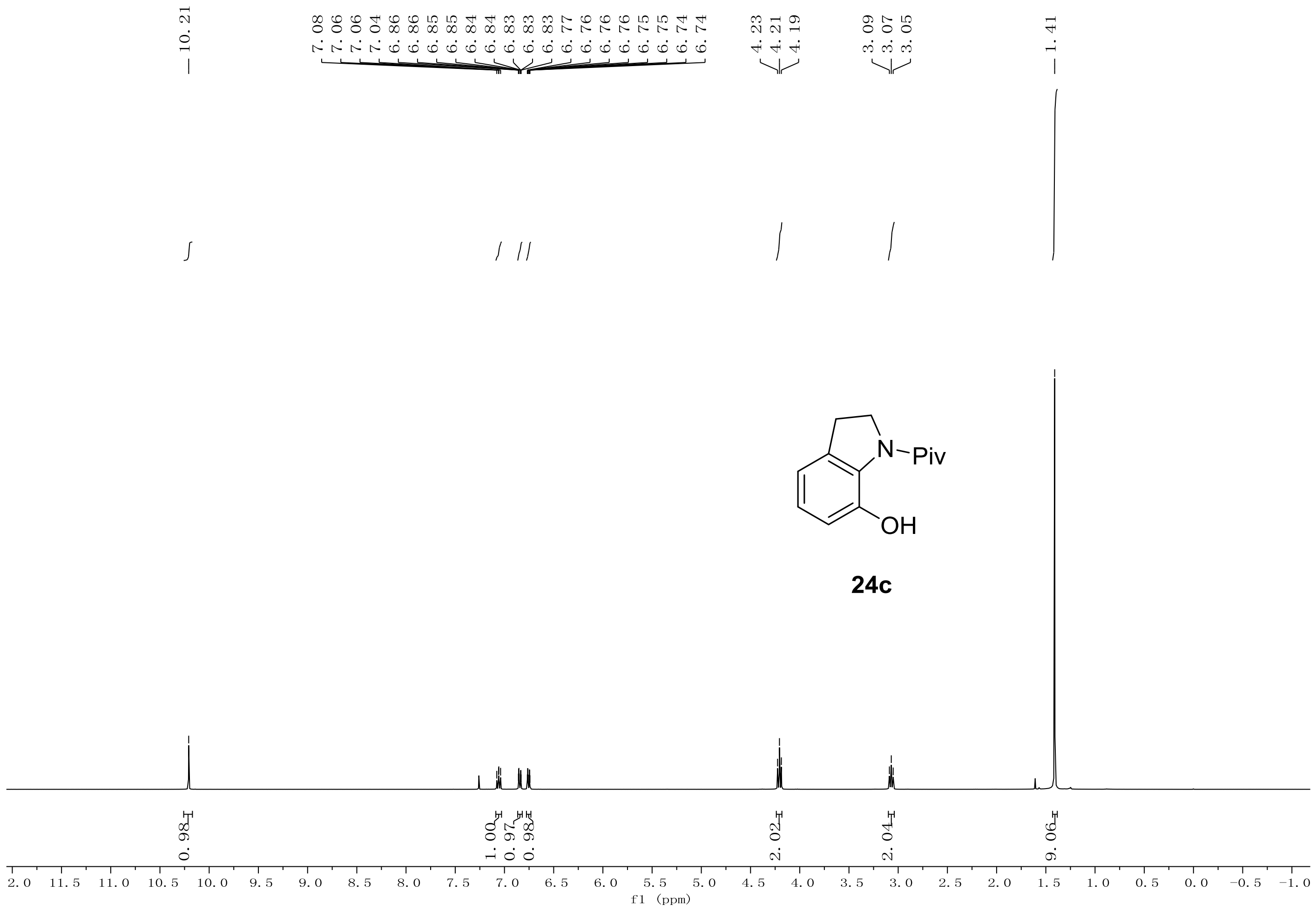

Supplementary Figure 50. ^{1}H NMR spectrum for **24c**

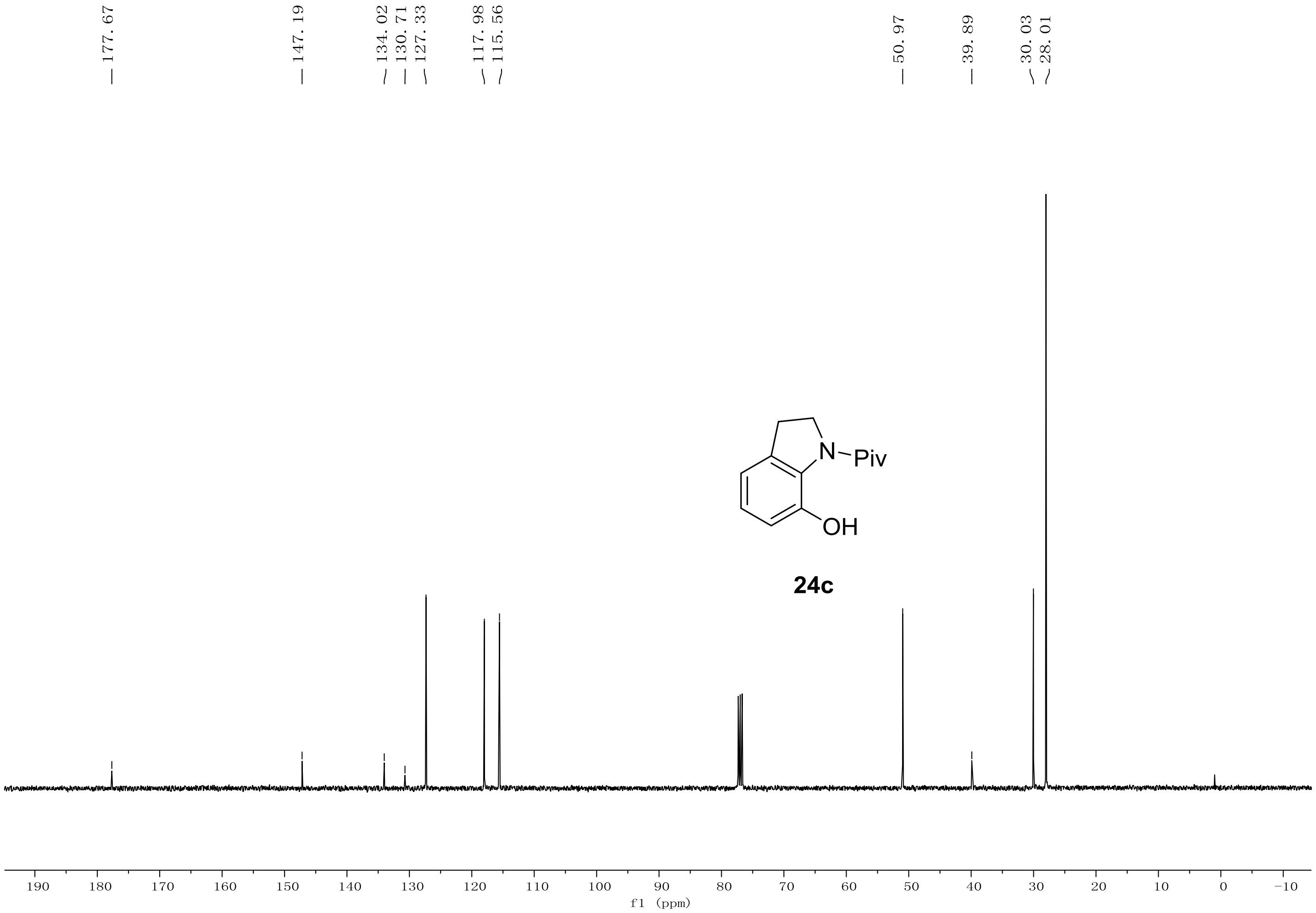

Supplementary Figure 51. ^{13}C NMR spectrum for **24c**

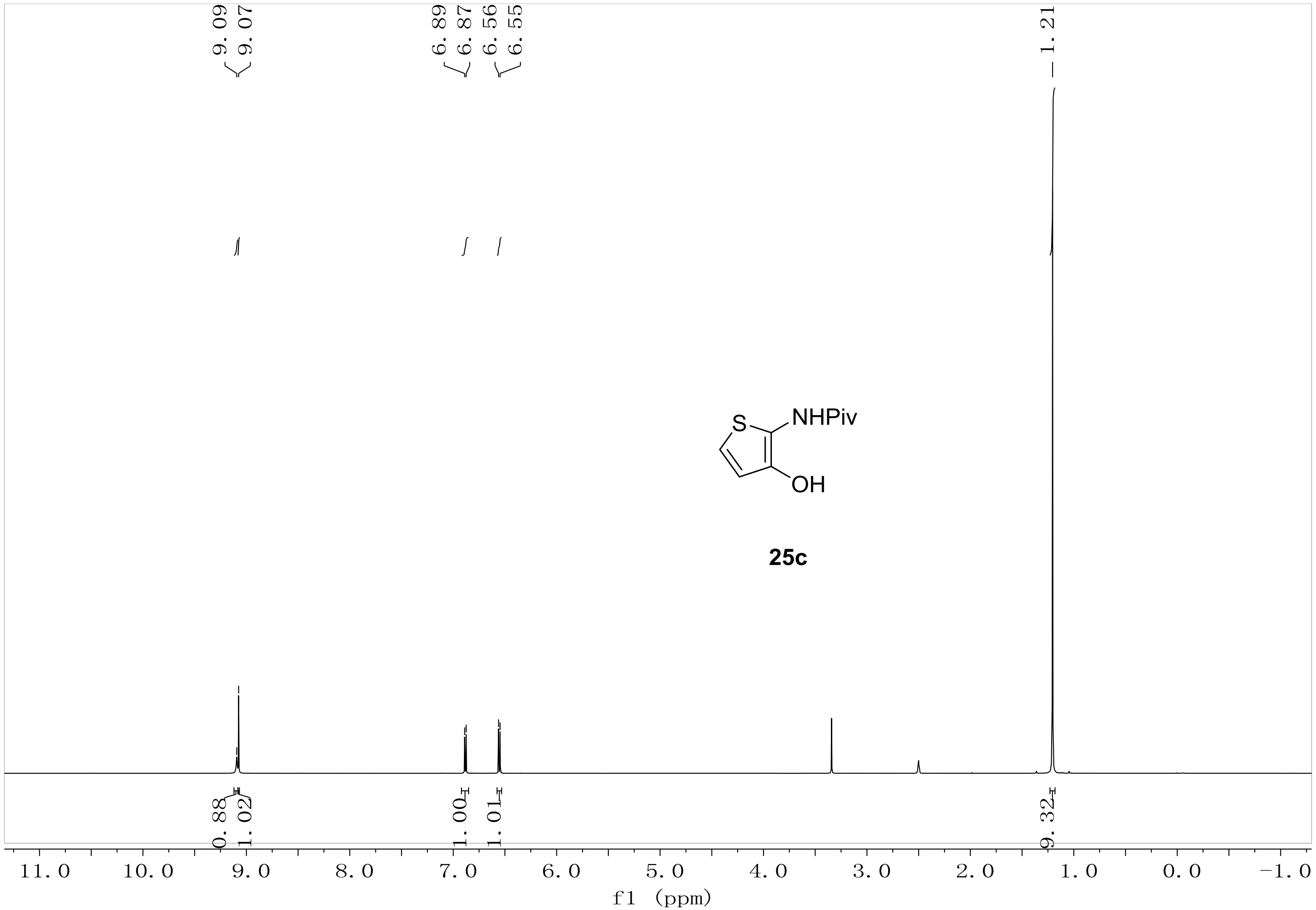

Supplementary Figure 52. ^{1}H NMR spectrum for **25c**

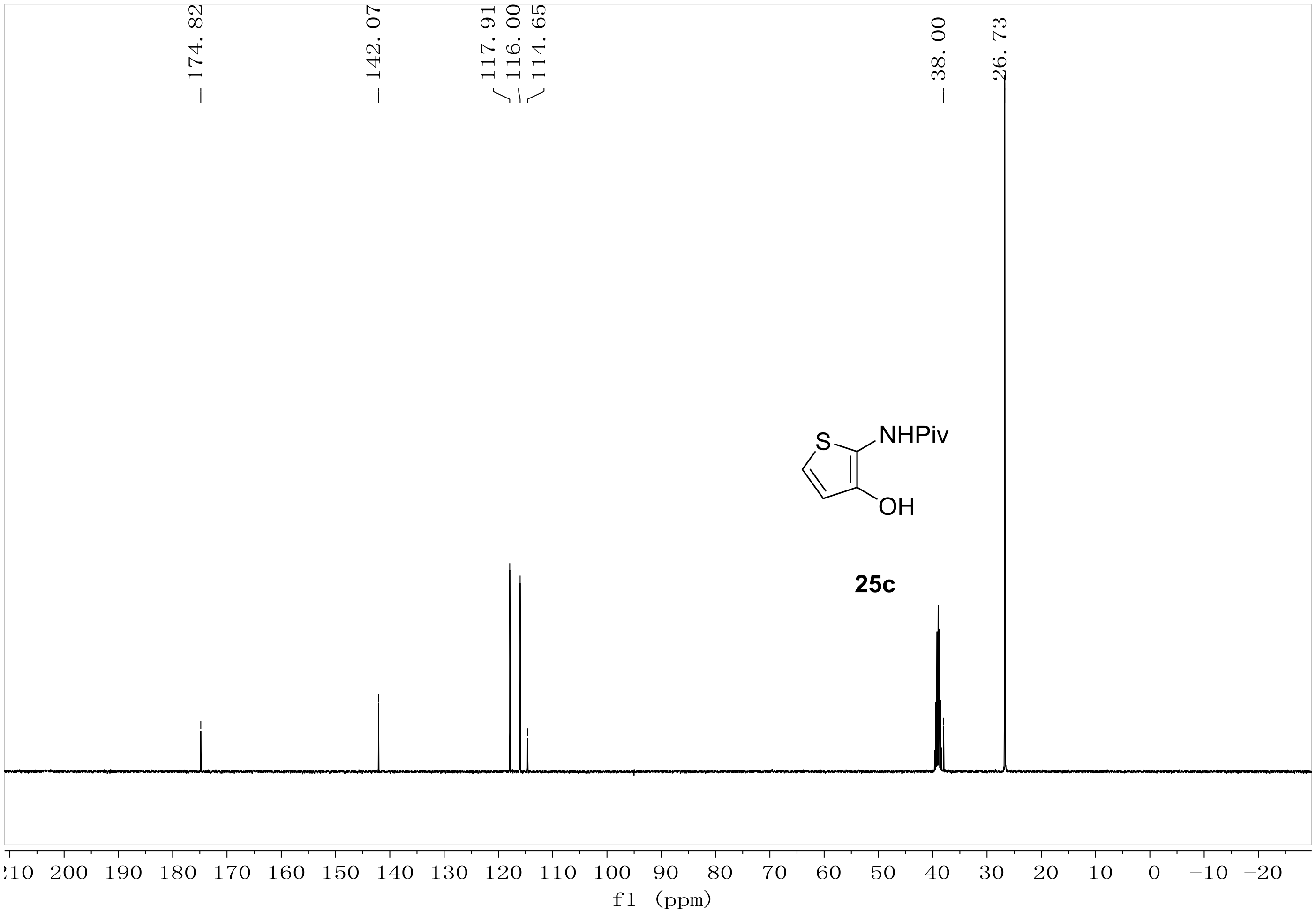

Supplementary Figure 53. ^{13}C NMR spectrum for **25c**

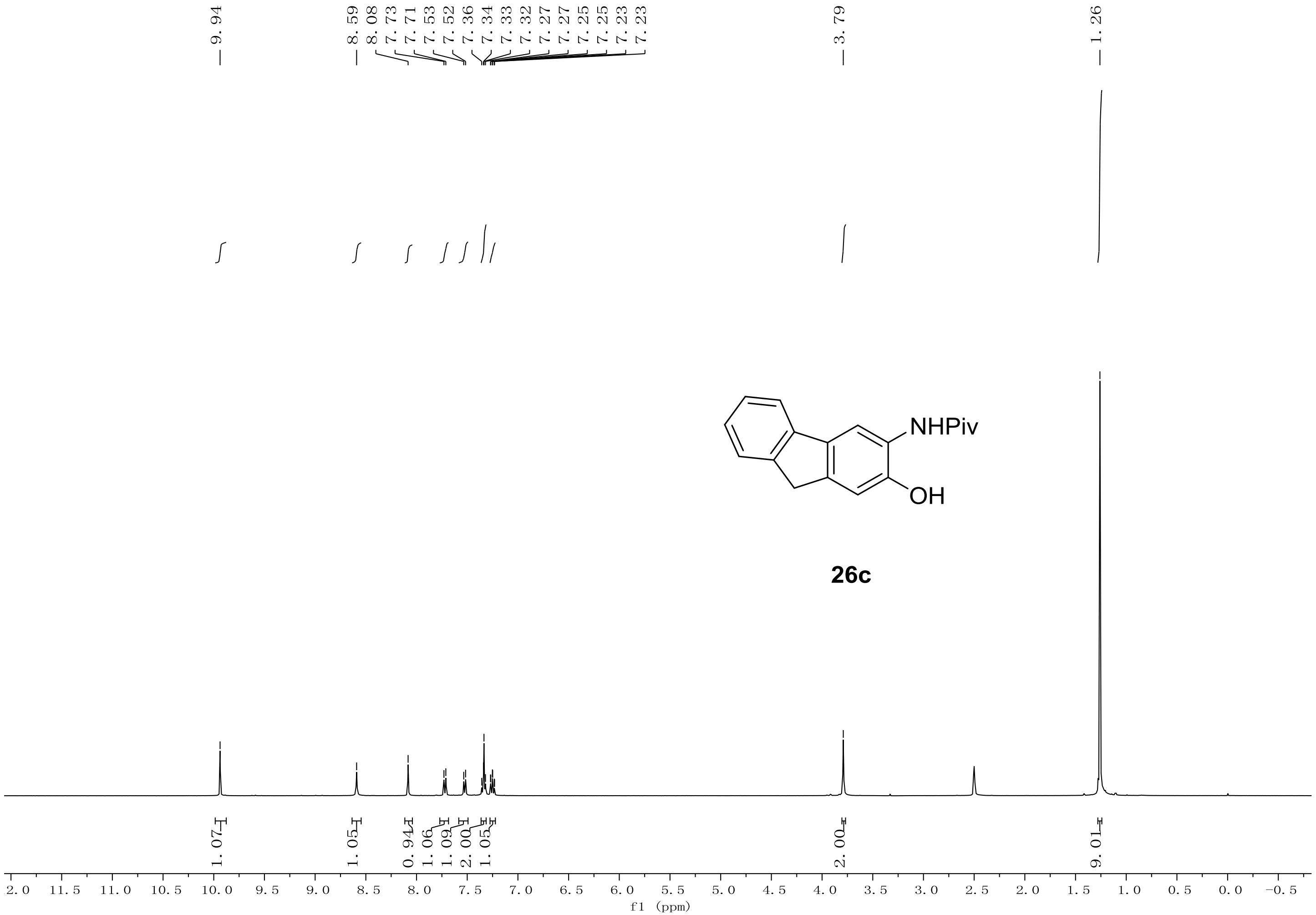

Supplementary Figure 54. ^{1}H NMR spectrum for **26c**

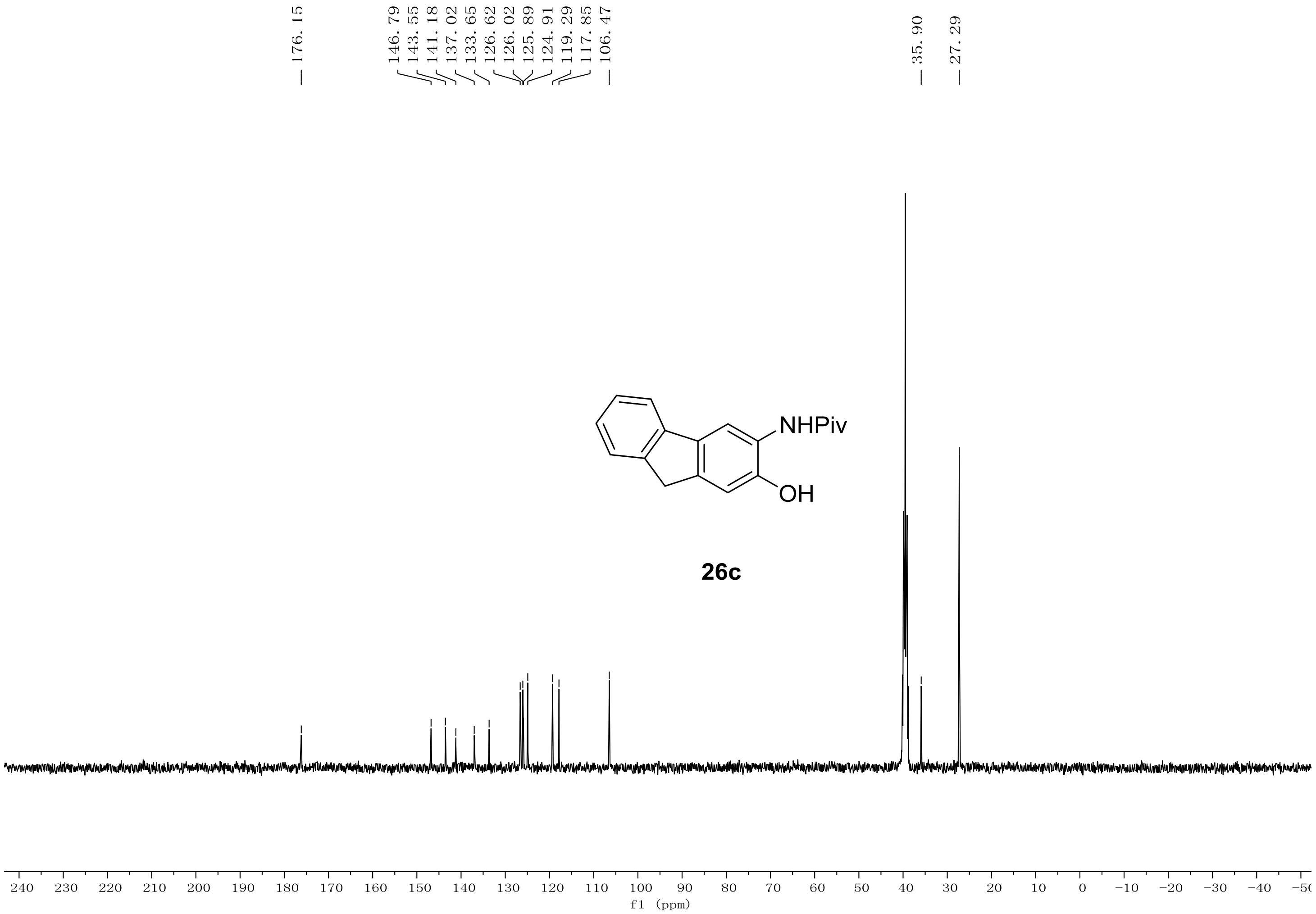

Supplementary Figure 55. ^{13}C NMR spectrum for **26c**

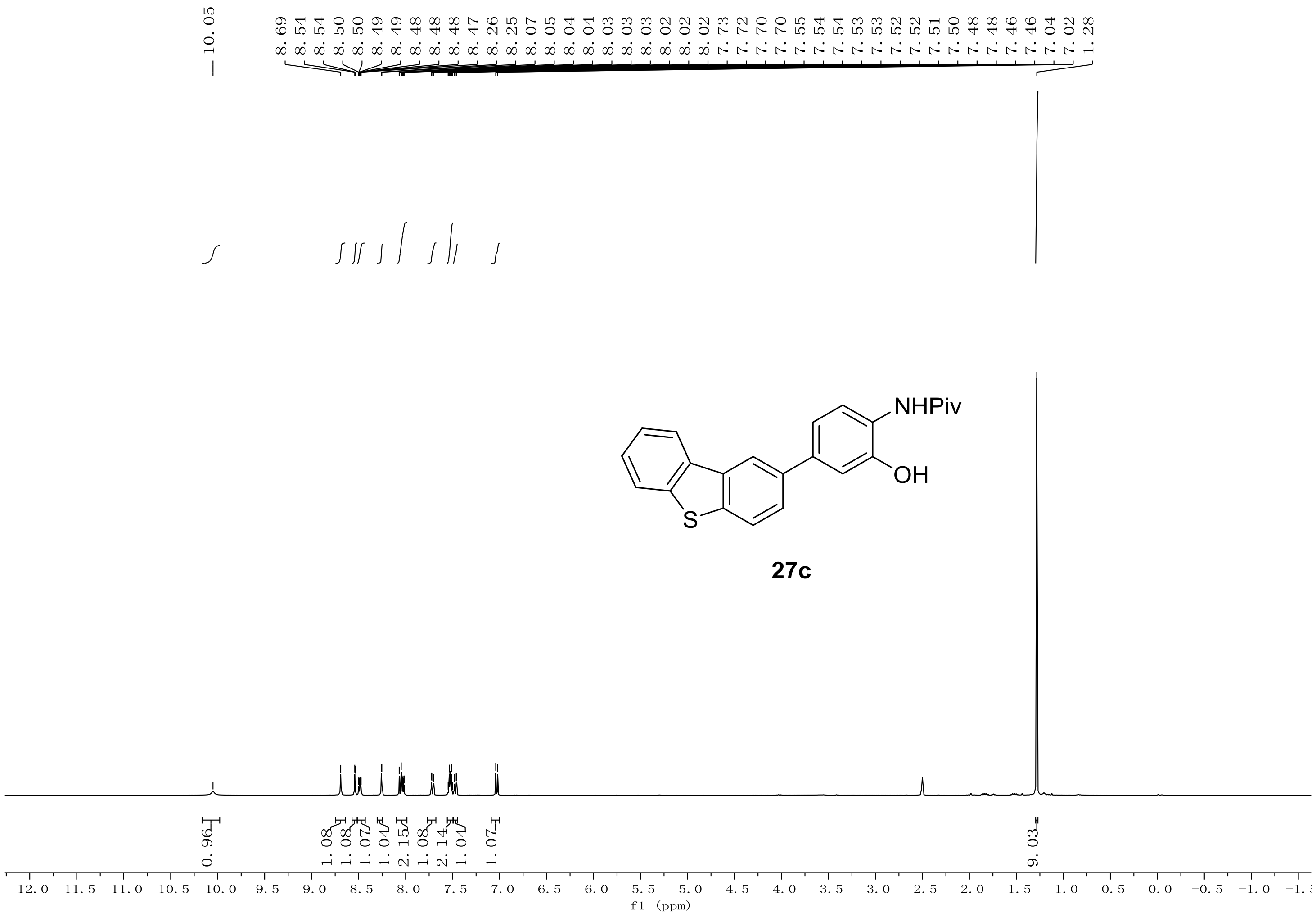

27c

Supplementary Figure 56. ^{1}H NMR spectrum for **27c**

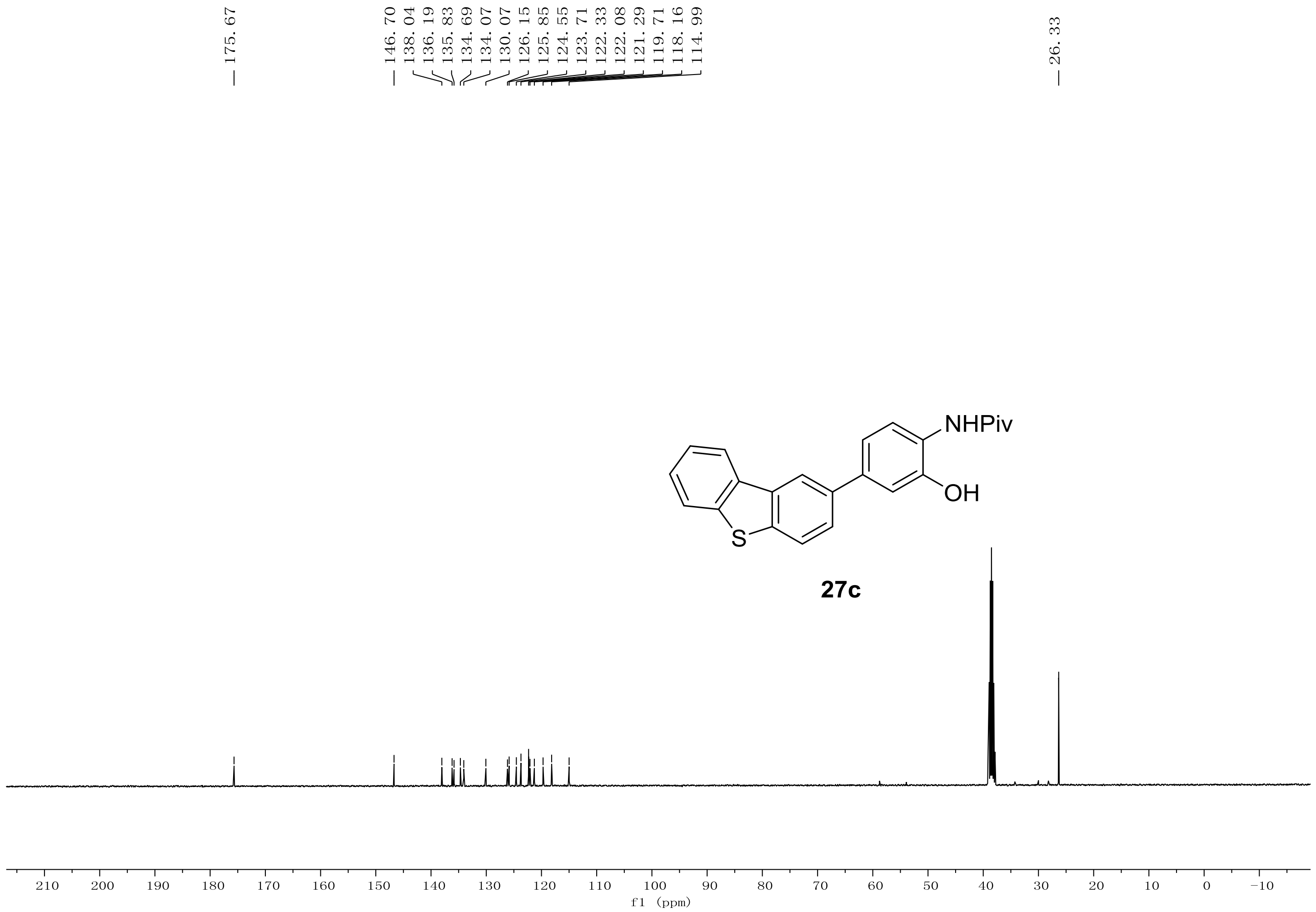

Supplementary Figure 57. ^{13}C NMR spectrum for **27c**

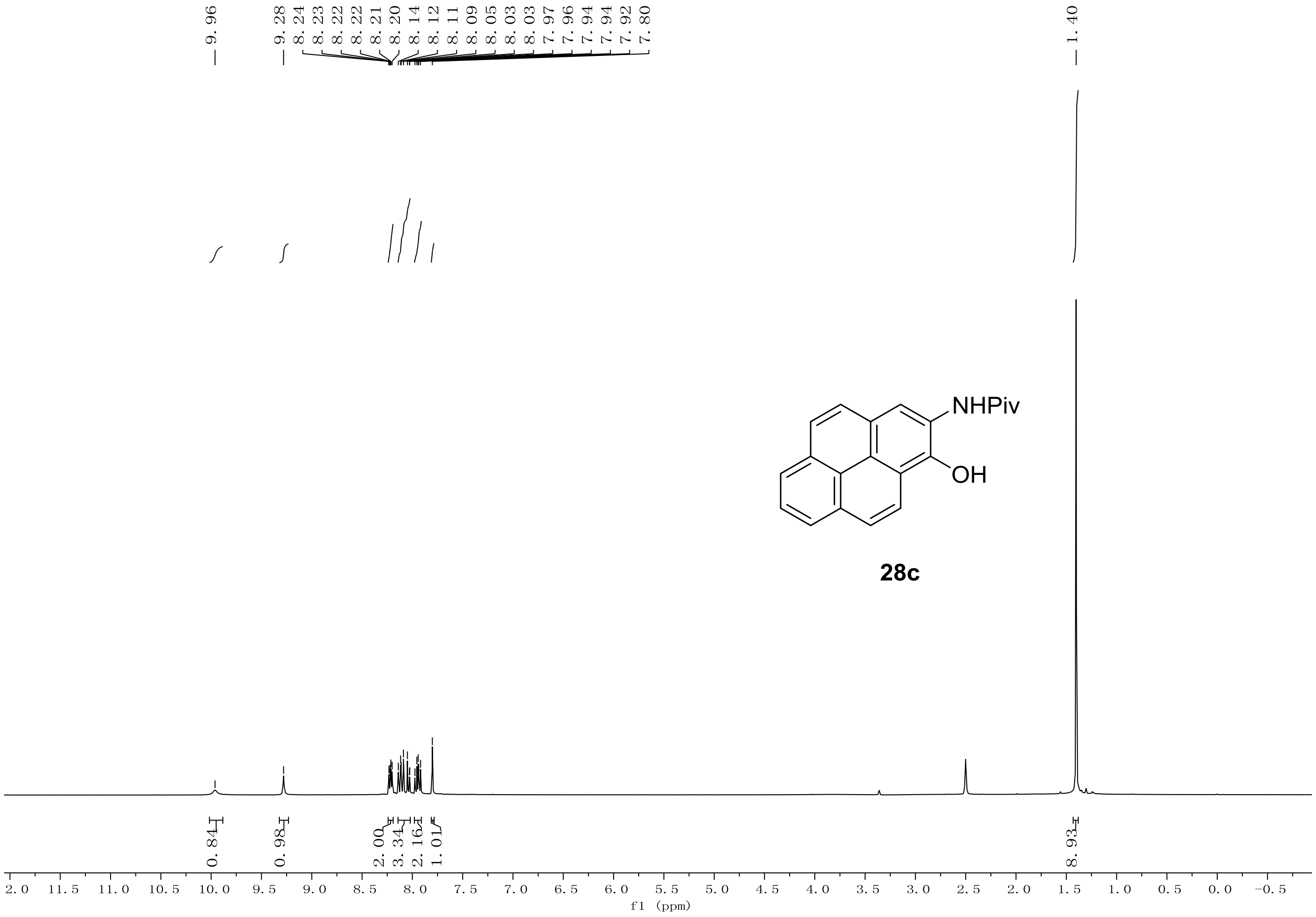

28c

Supplementary Figure 58. ^{1}H NMR spectrum for **28c**

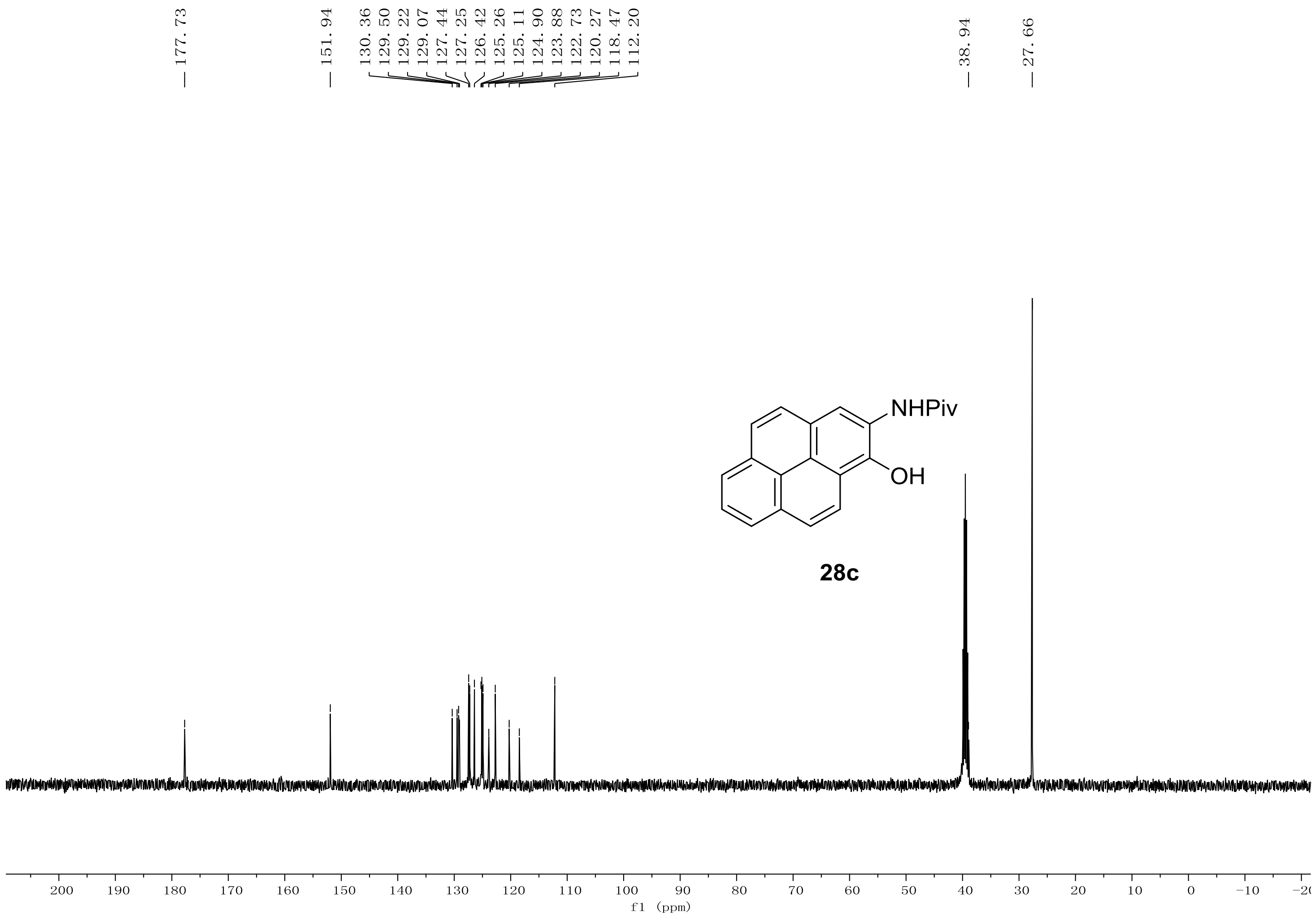

Supplementary Figure 59. ^{13}C NMR spectrum for **28c**

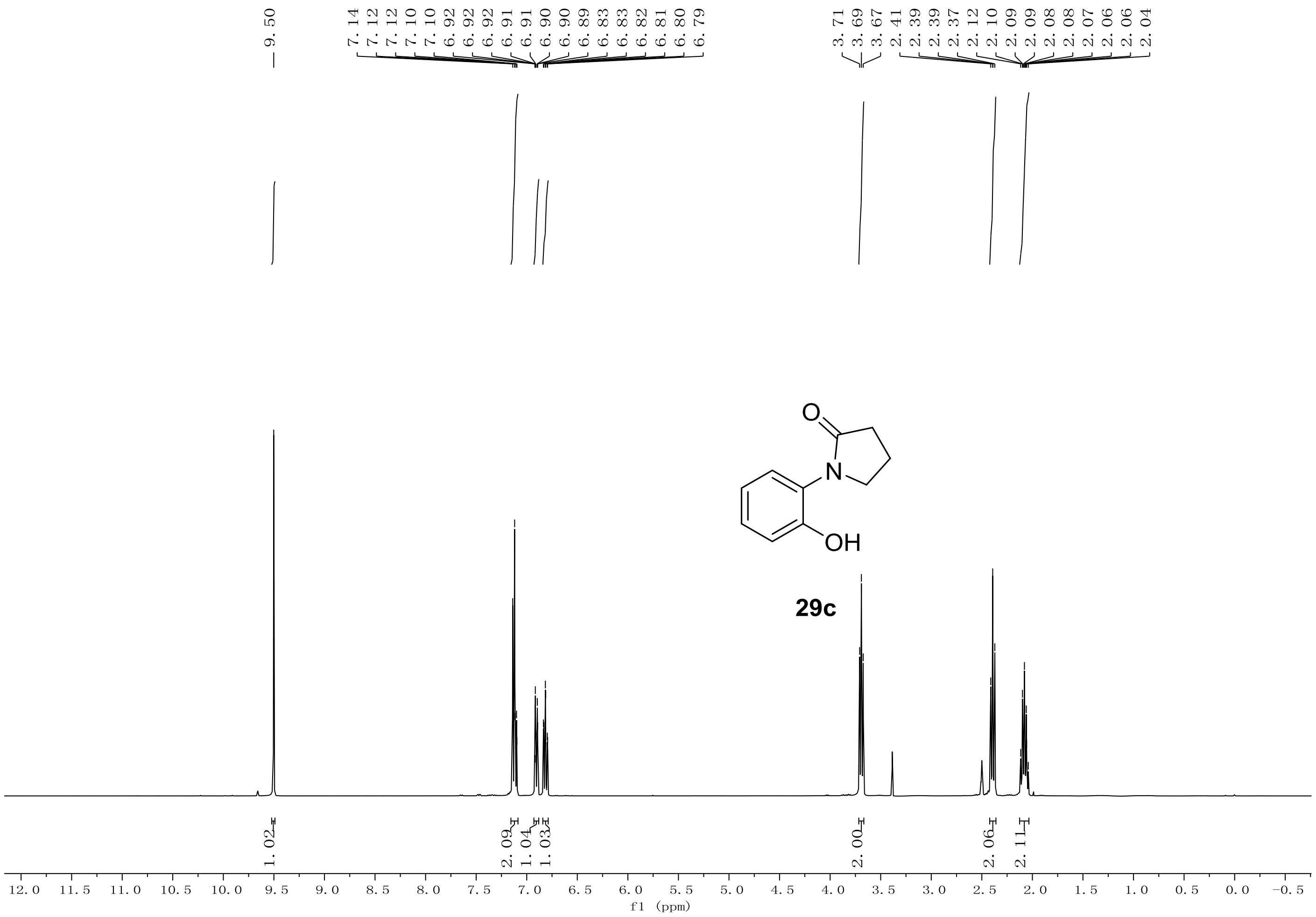

Supplementary Figure 60. ^{1}H NMR spectrum for **29c**

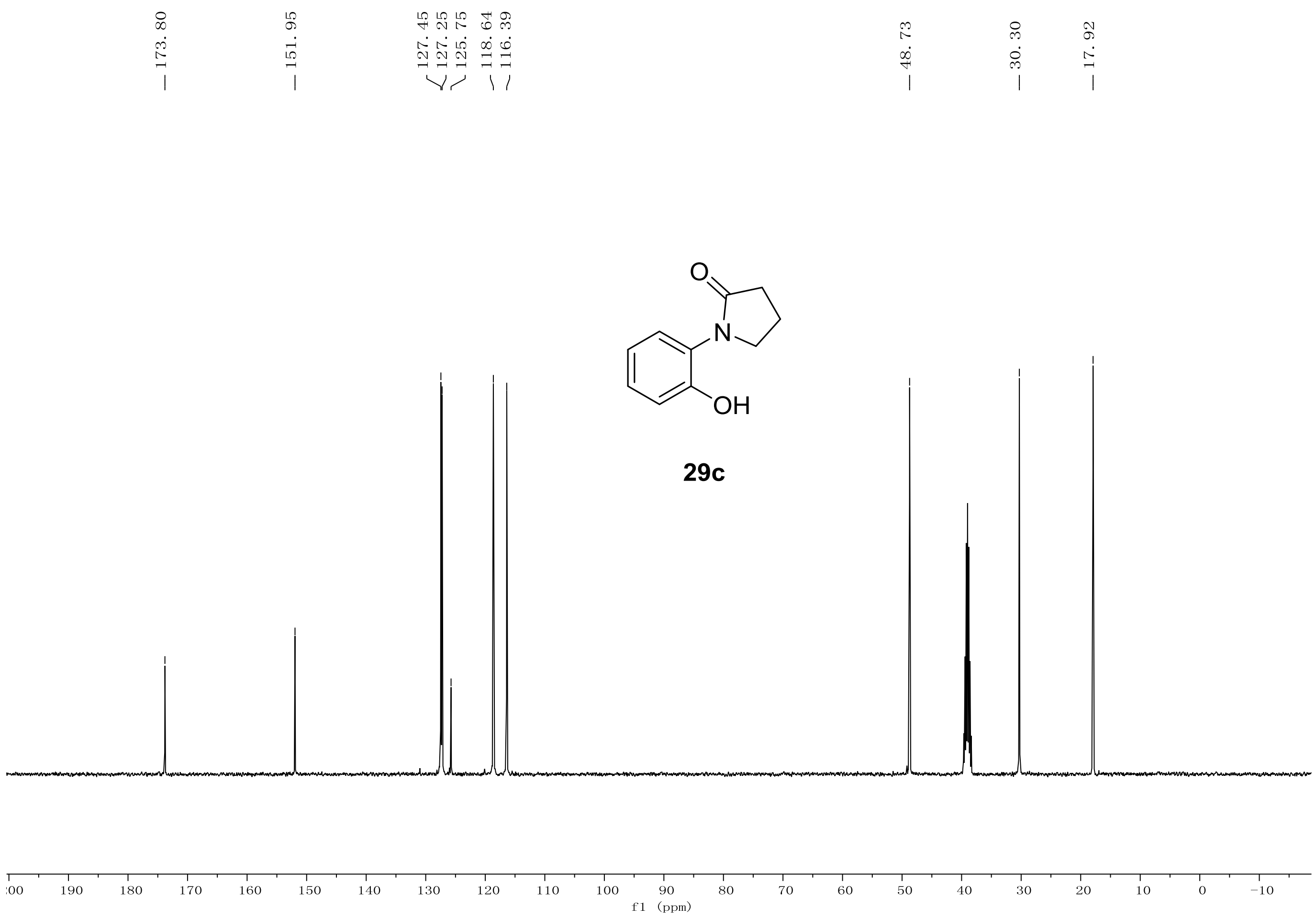

Supplementary Figure 61. ^{13}C NMR spectrum for **29c**

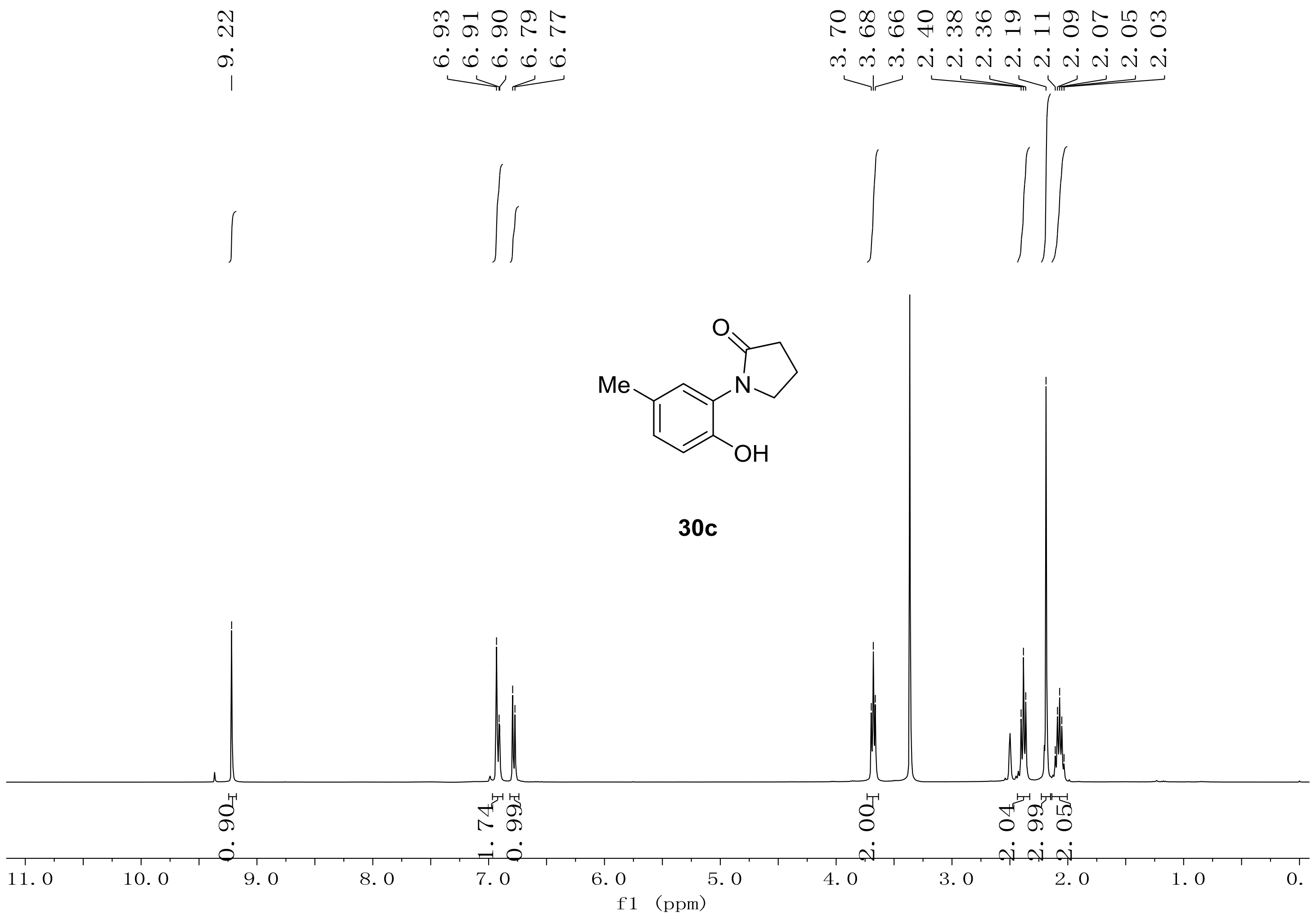

Supplementary Figure 62. ^{1}H NMR spectrum for **30c**

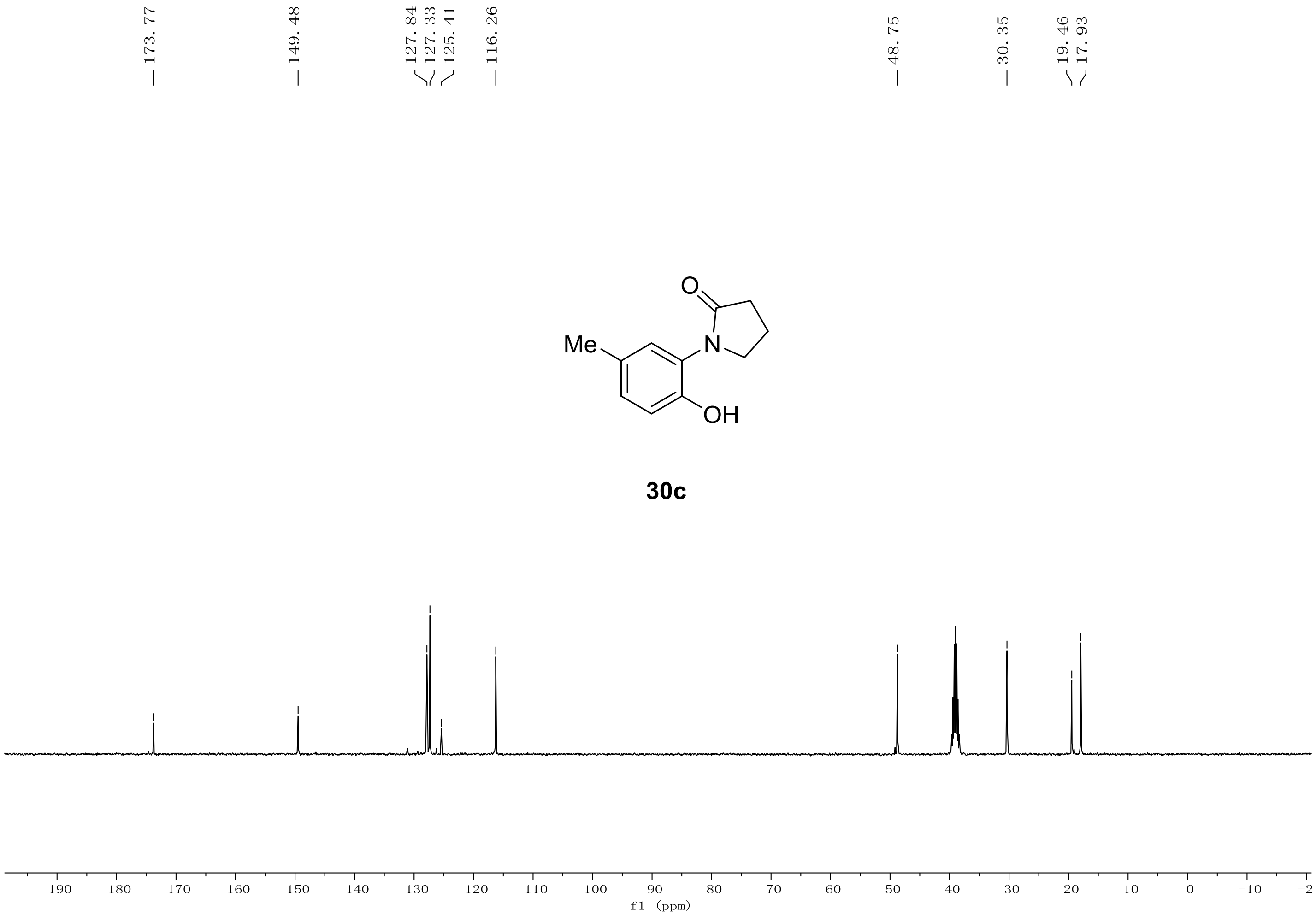

Supplementary Figure 63. ^{13}C NMR spectrum for **30c**

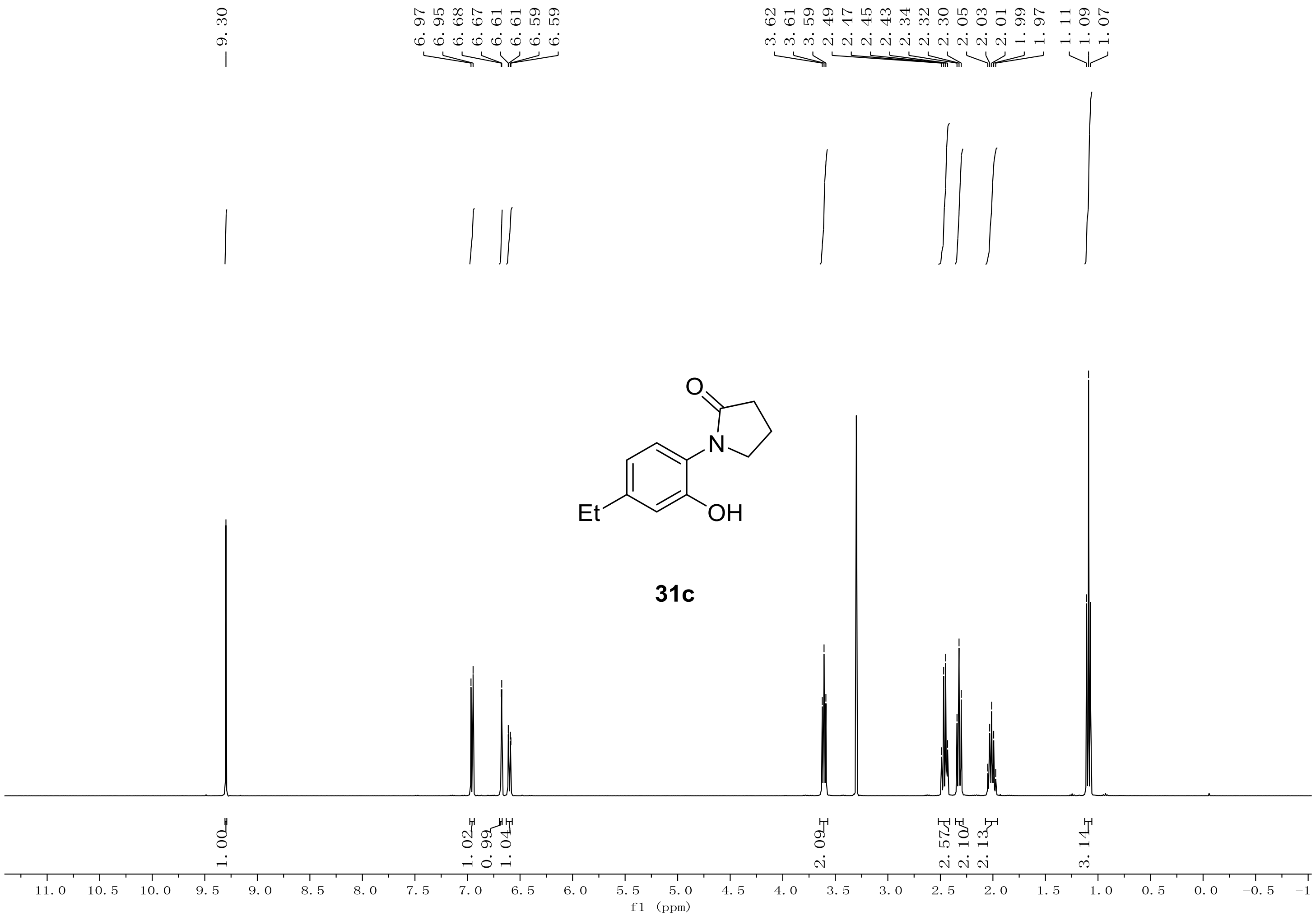

Supplementary Figure 64. ^{1}H NMR spectrum for **31c**

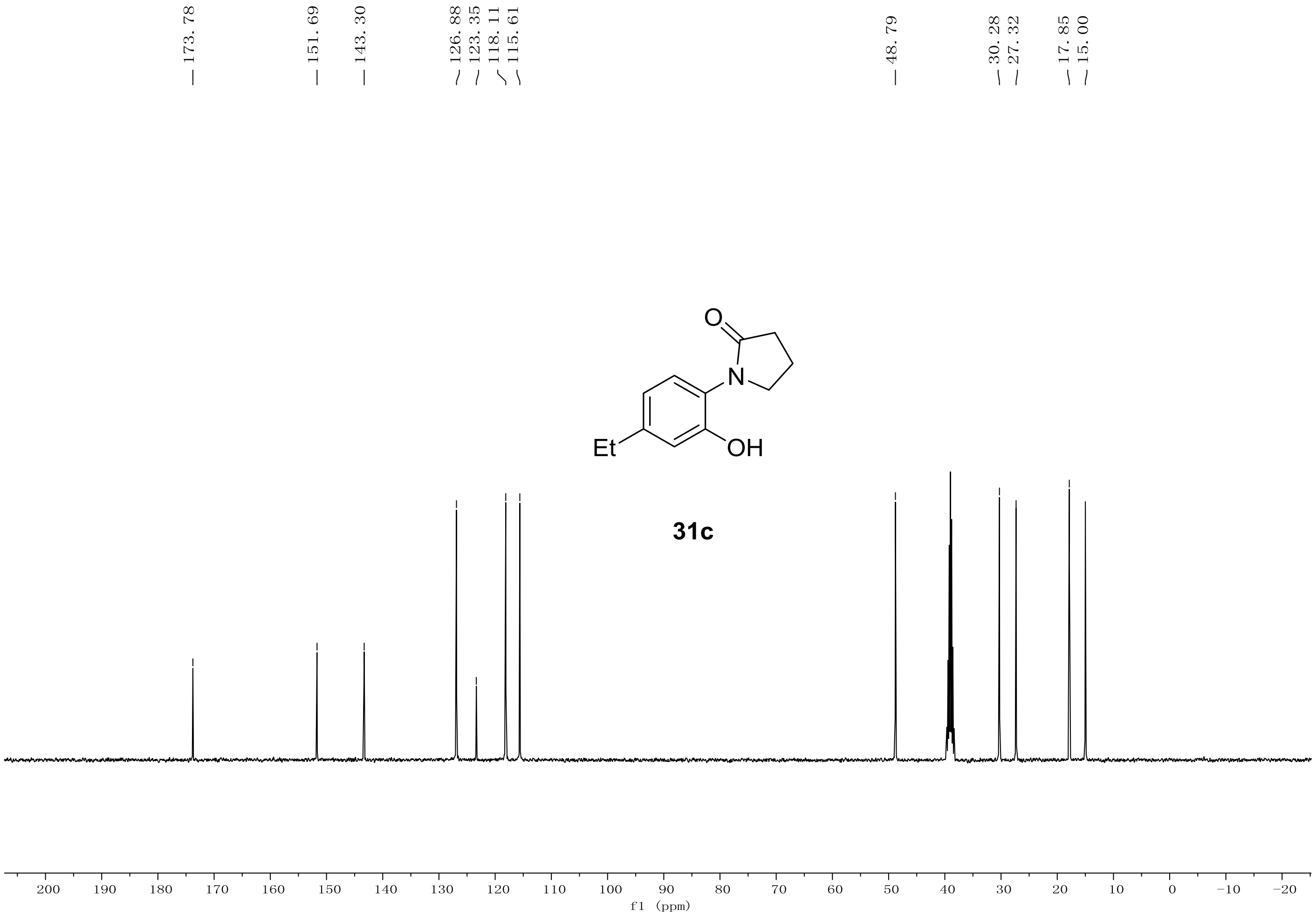

Supplementary Figure 65. ^{13}C NMR spectrum for **31c**

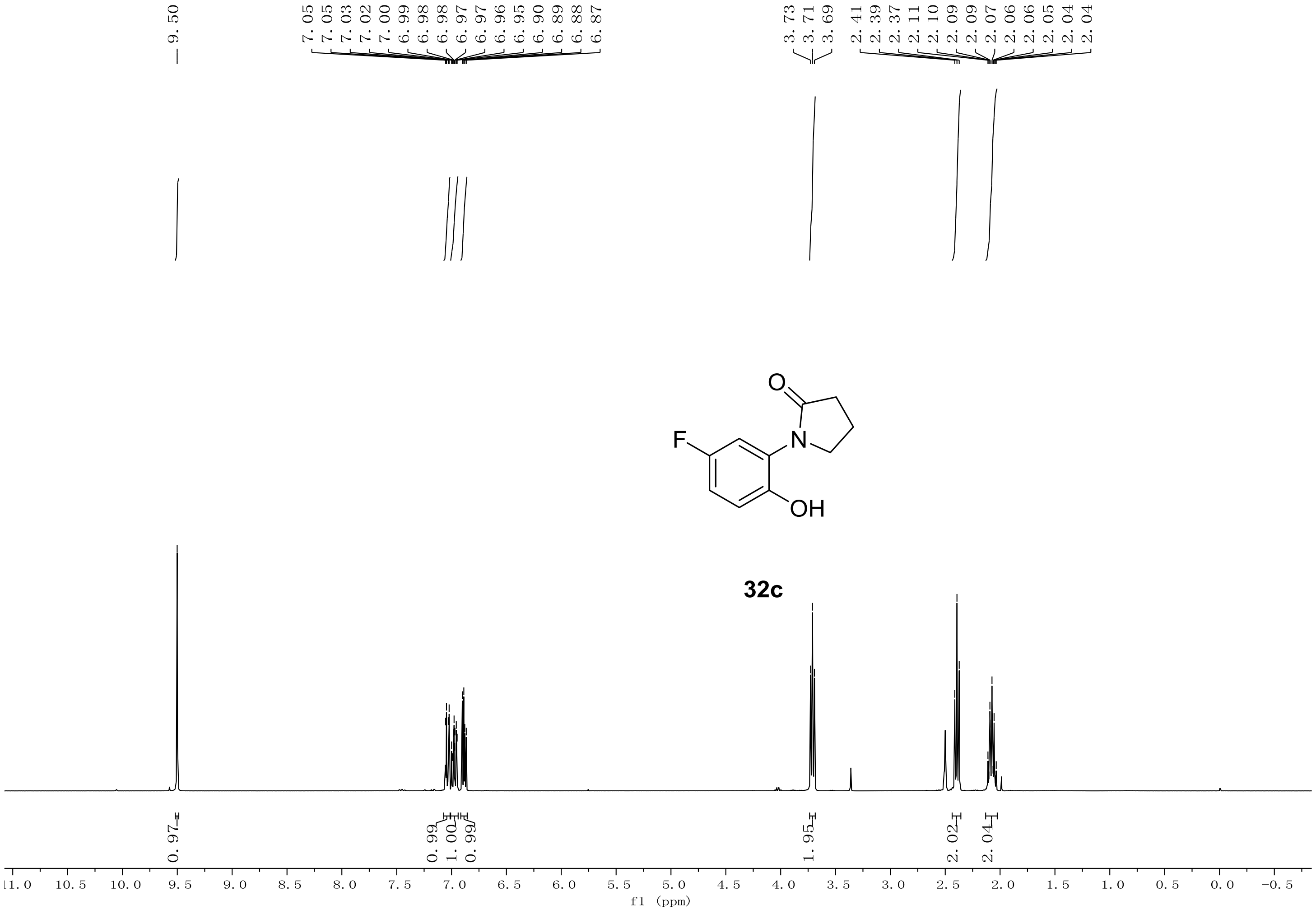

Supplementary Figure 66. ^{1}H NMR spectrum for **32c**

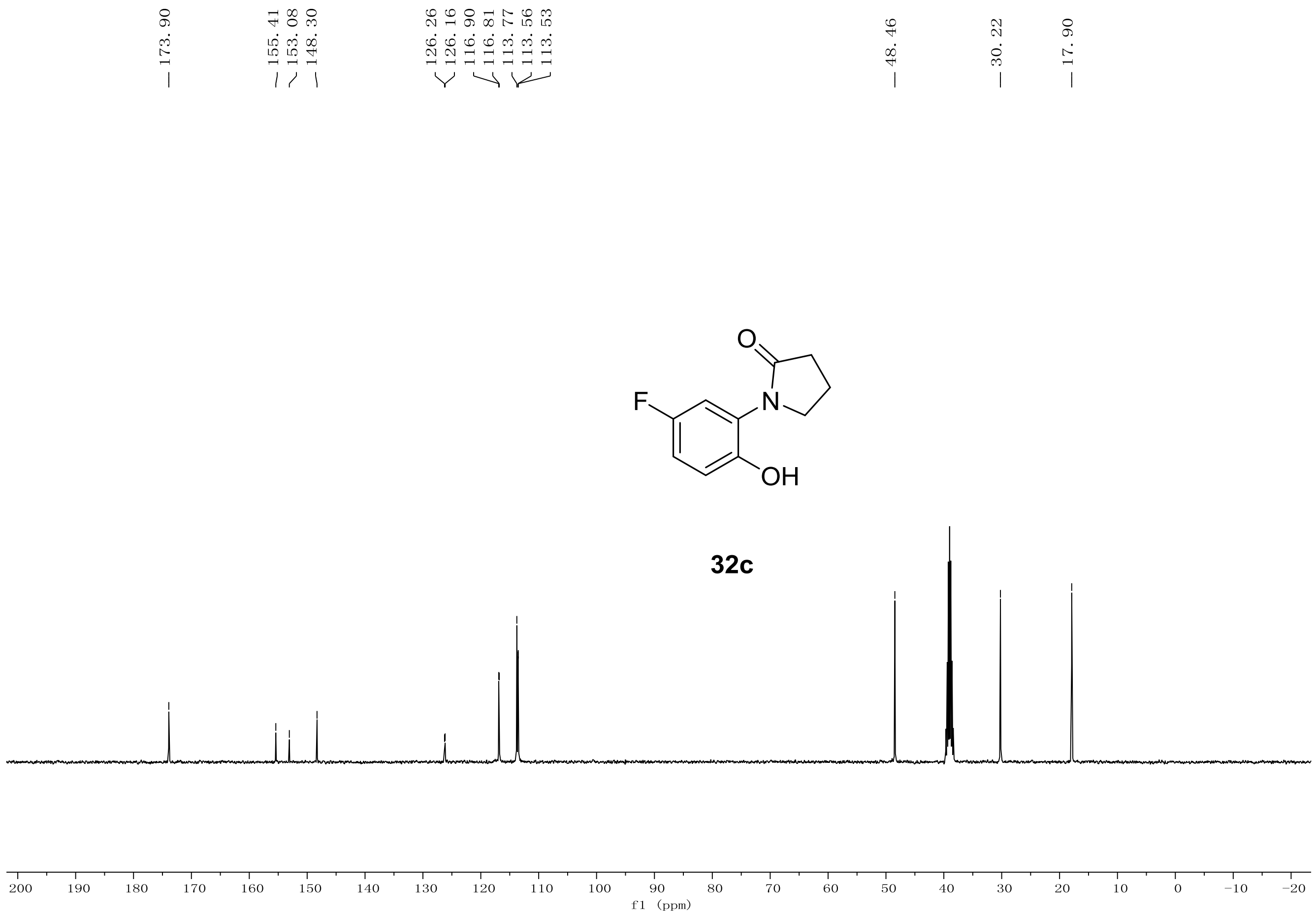

Supplementary Figure 67. ^{13}C NMR spectrum for **32c**

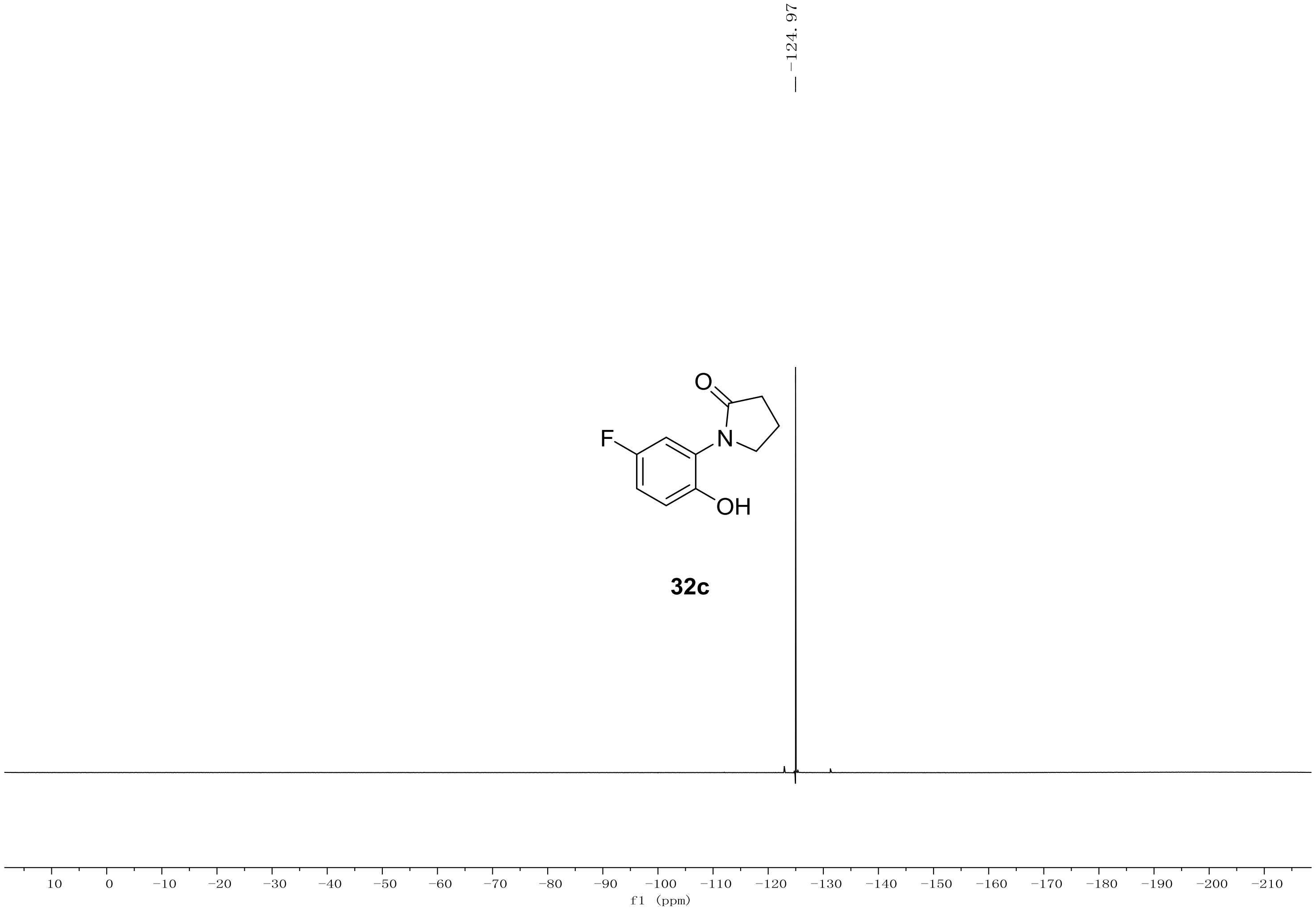

Supplementary Figure 68. ^{19}F NMR spectrum for **32c**

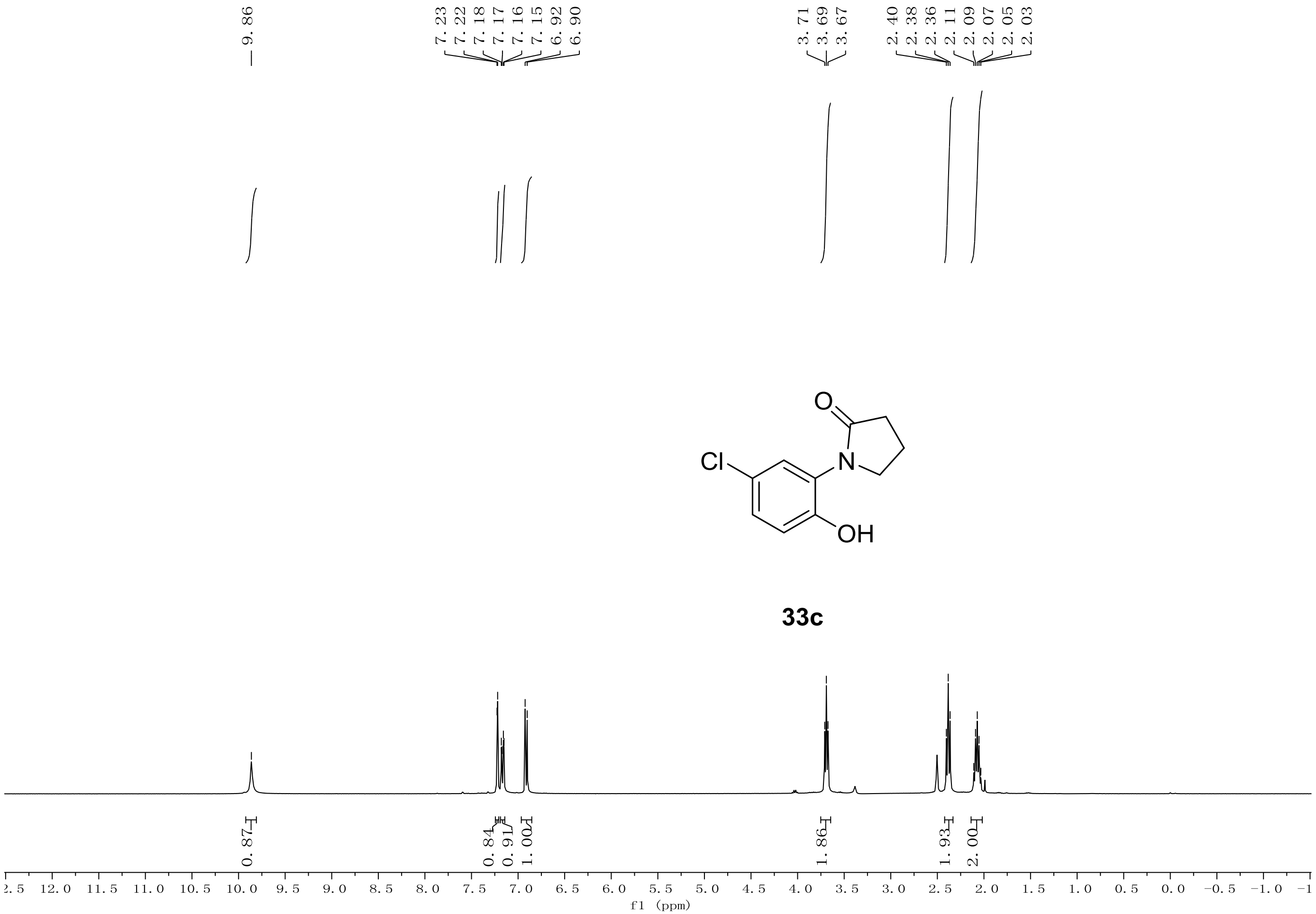

Supplementary Figure 69. ^{1}H NMR spectrum for **33c**

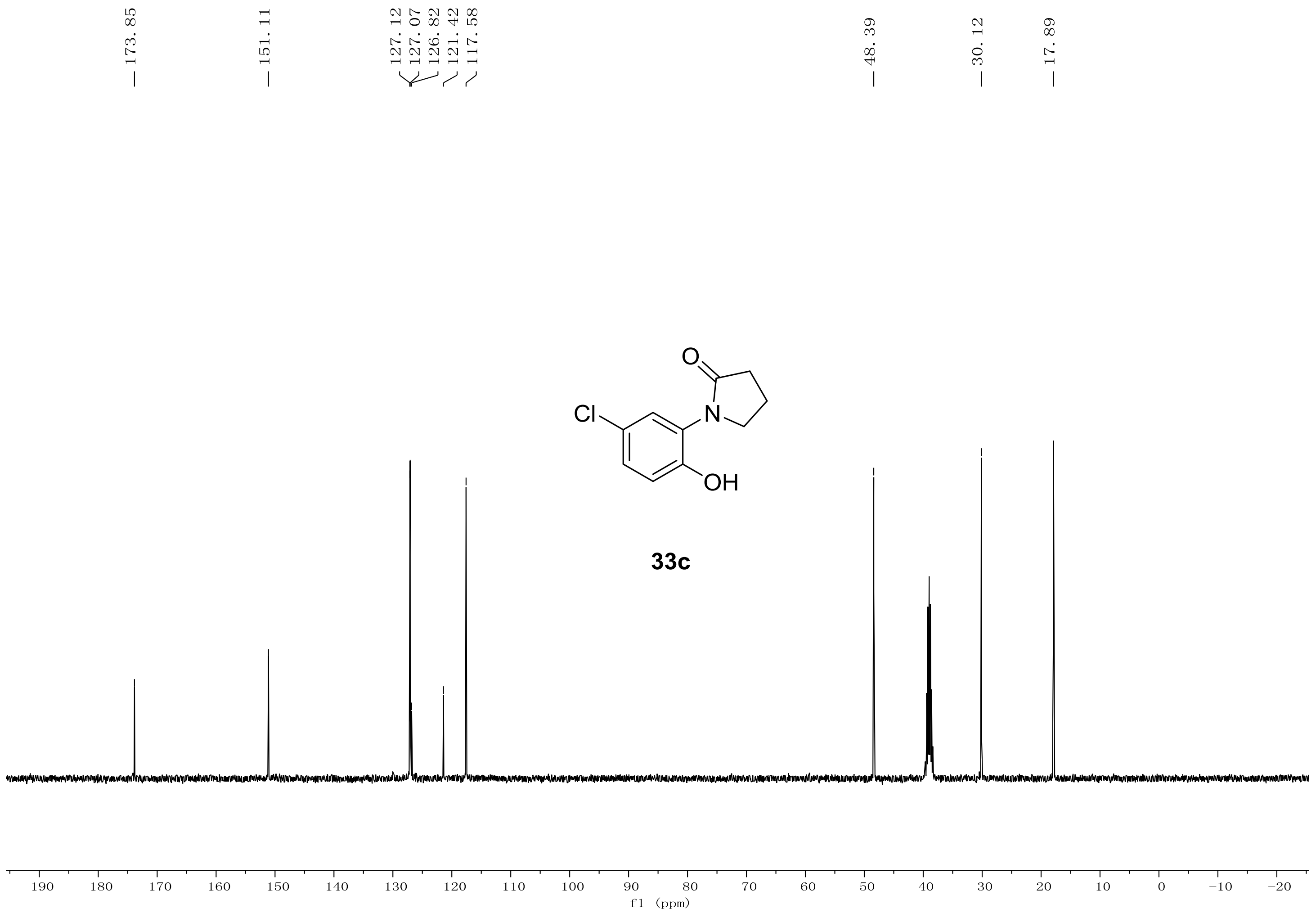

Supplementary Figure 70. ^{13}C NMR spectrum for **33c**

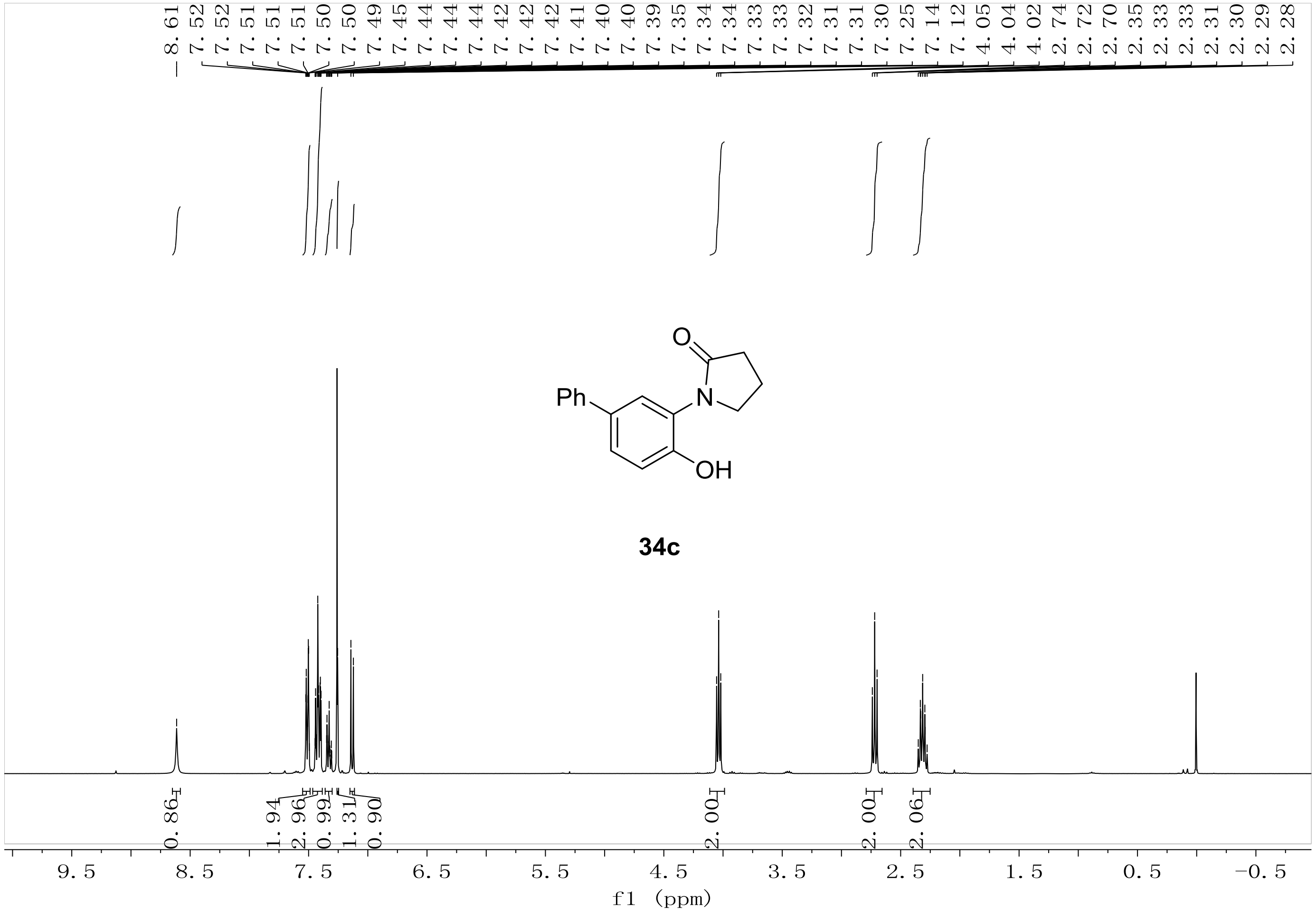

Supplementary Figure 71. ^{1}H NMR spectrum for **34c**

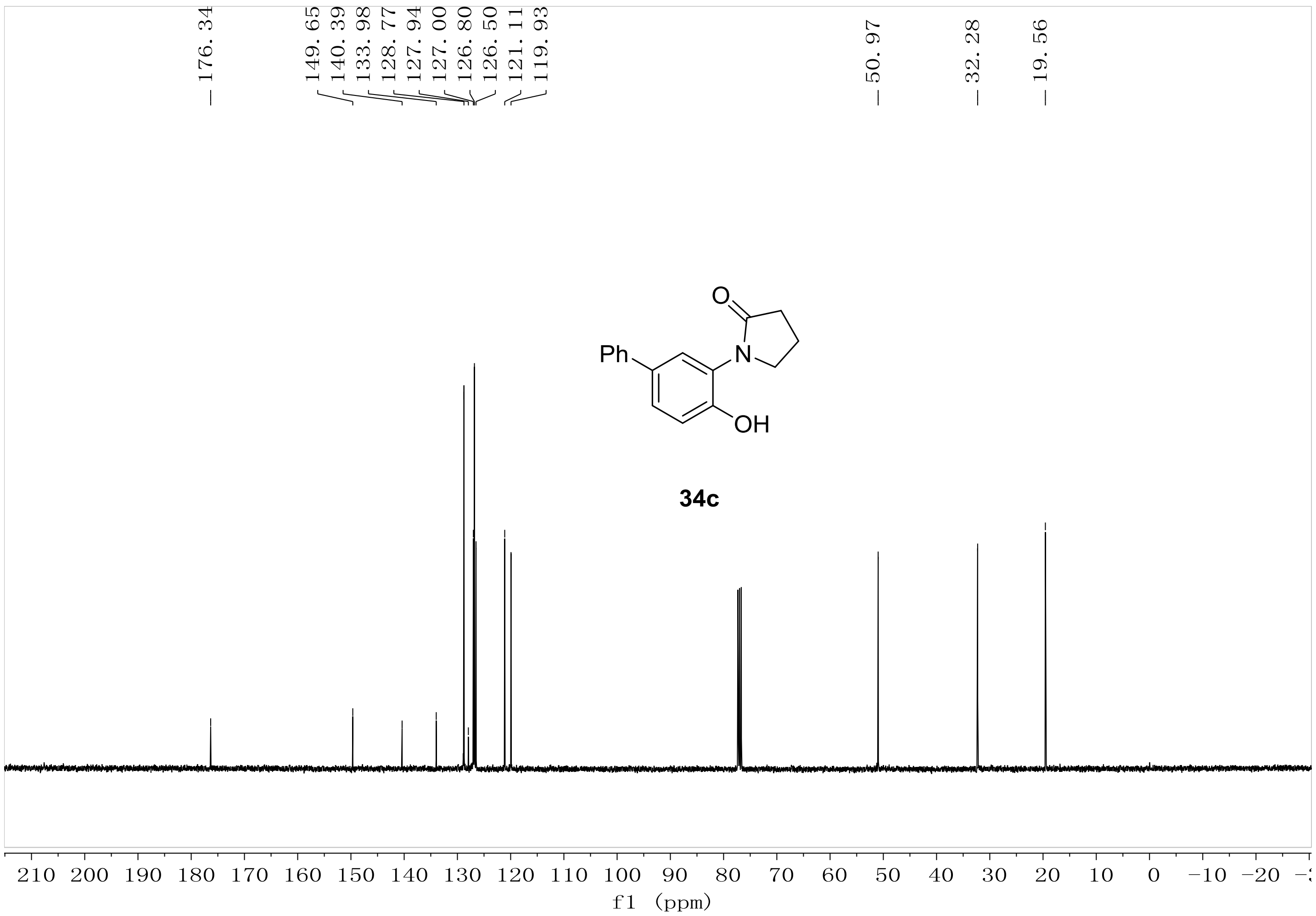

Supplementary Figure 72. ^{1}H NMR spectrum for **34c**

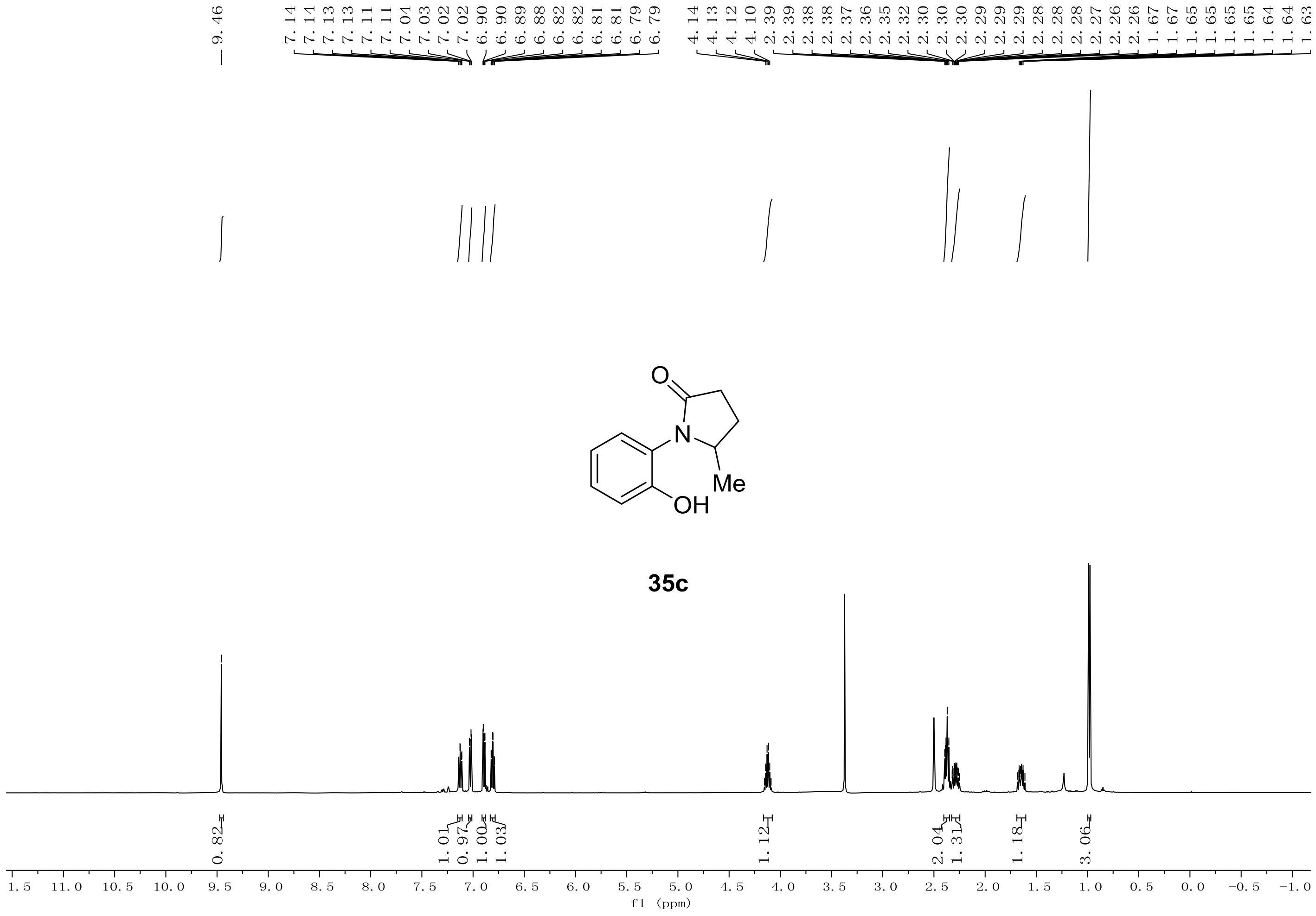

Supplementary Figure 73. ^{1}H NMR spectrum for **35c**

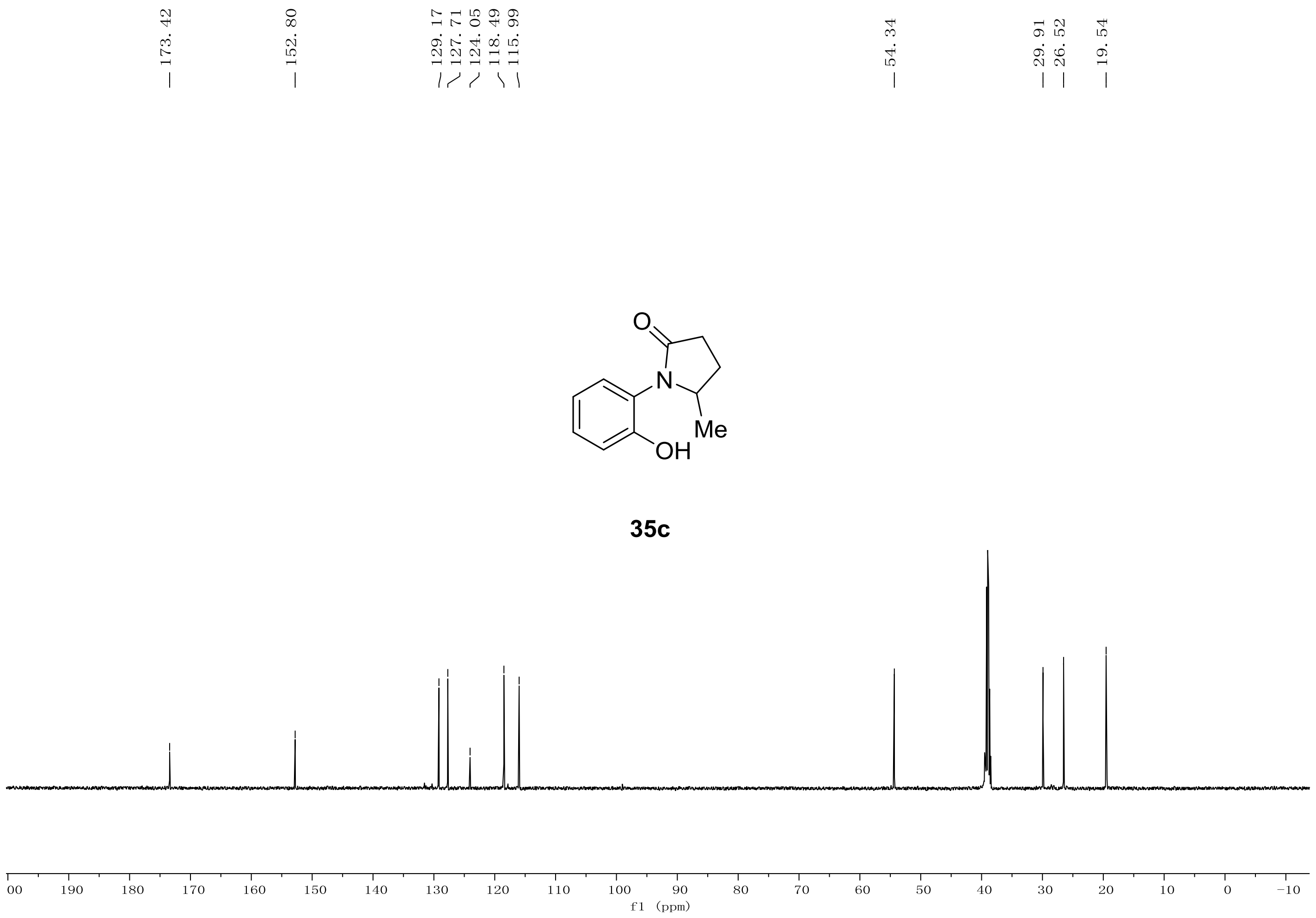

Supplementary Figure 74. ^{13}C NMR spectrum for **35c**

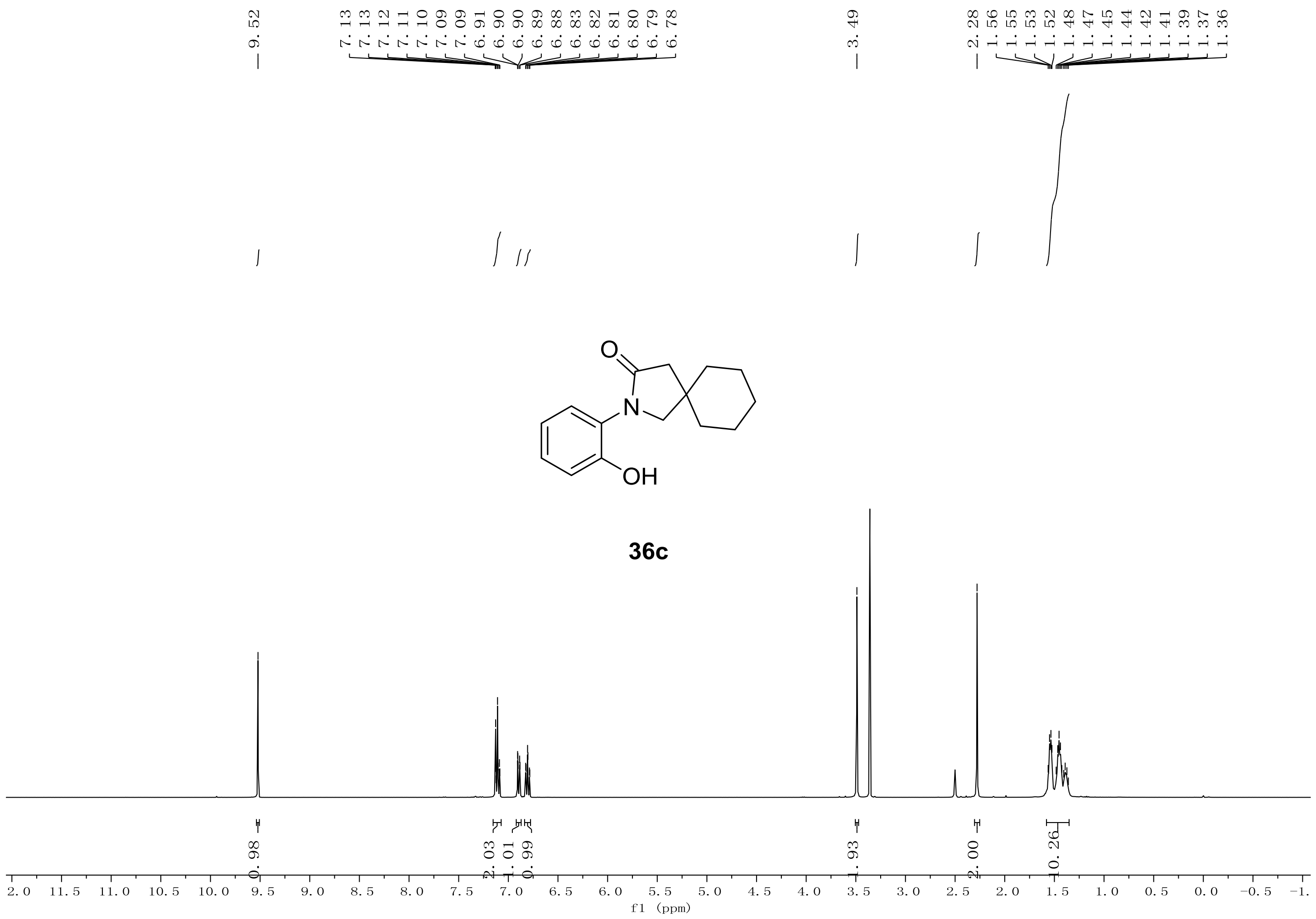

Supplementary Figure 75. ^{1}H NMR spectrum for **36c**

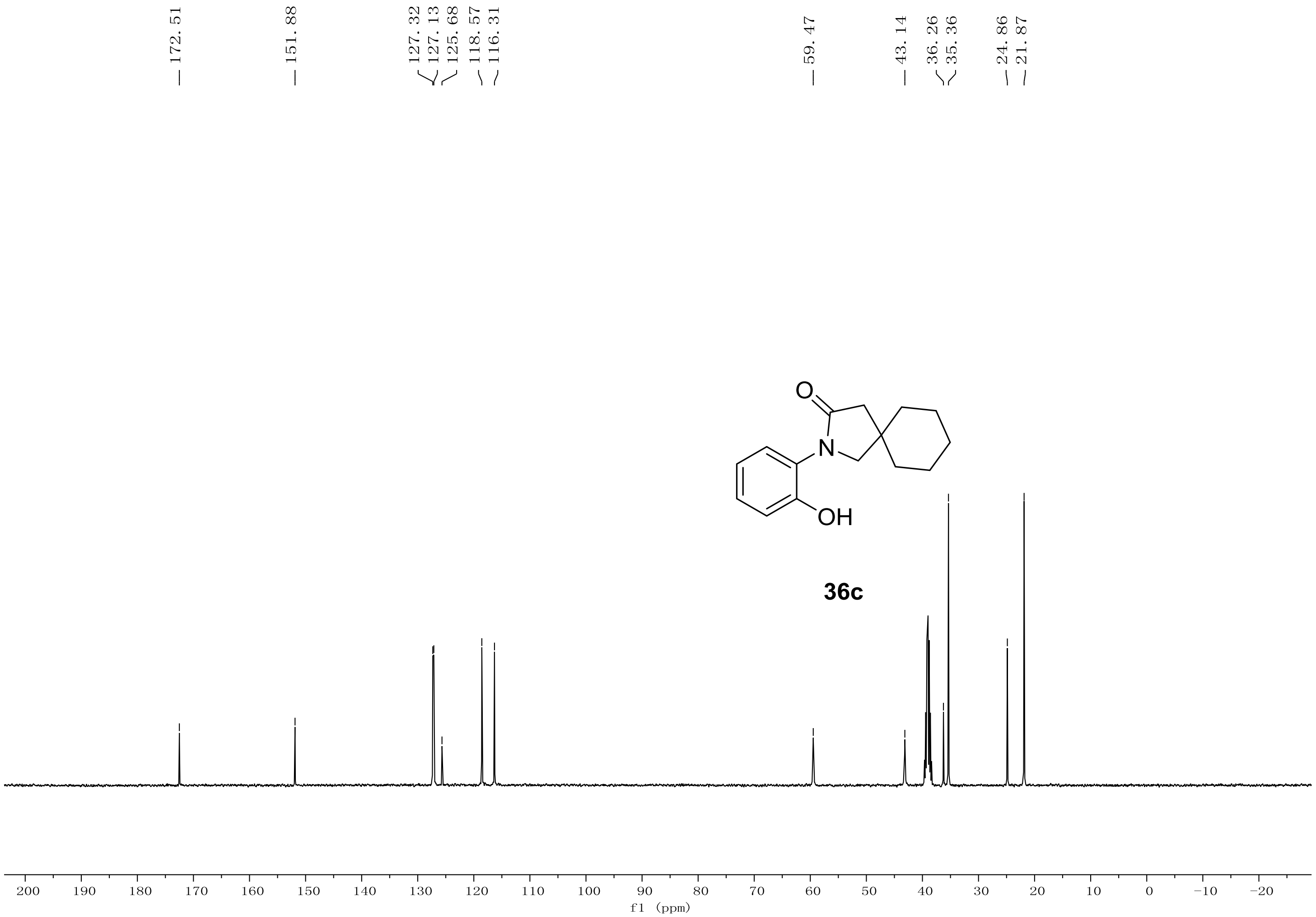

Supplementary Figure 76. ^{13}C NMR spectrum for **36c**

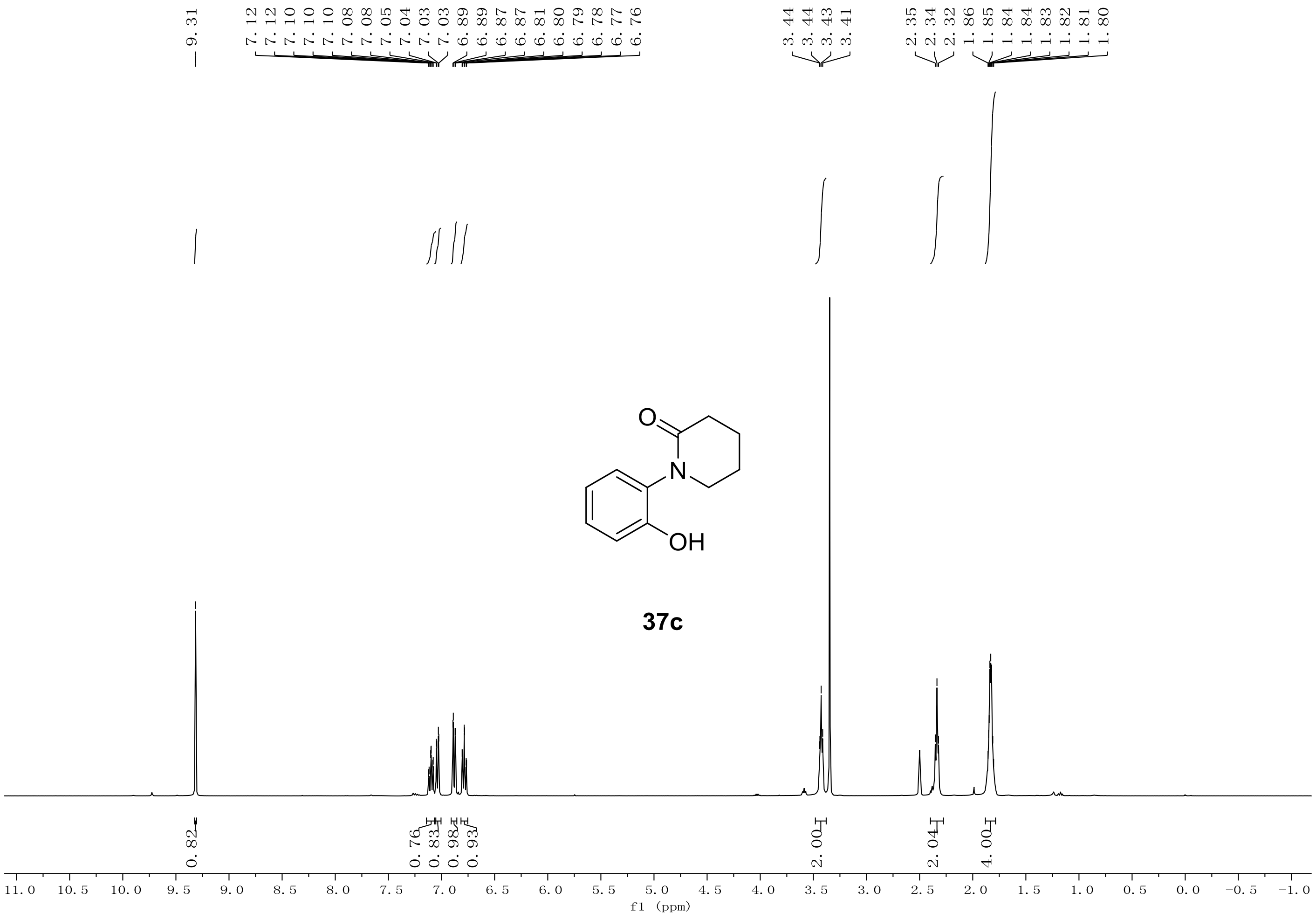

Supplementary Figure 77. ^{1}H NMR spectrum for **37c**

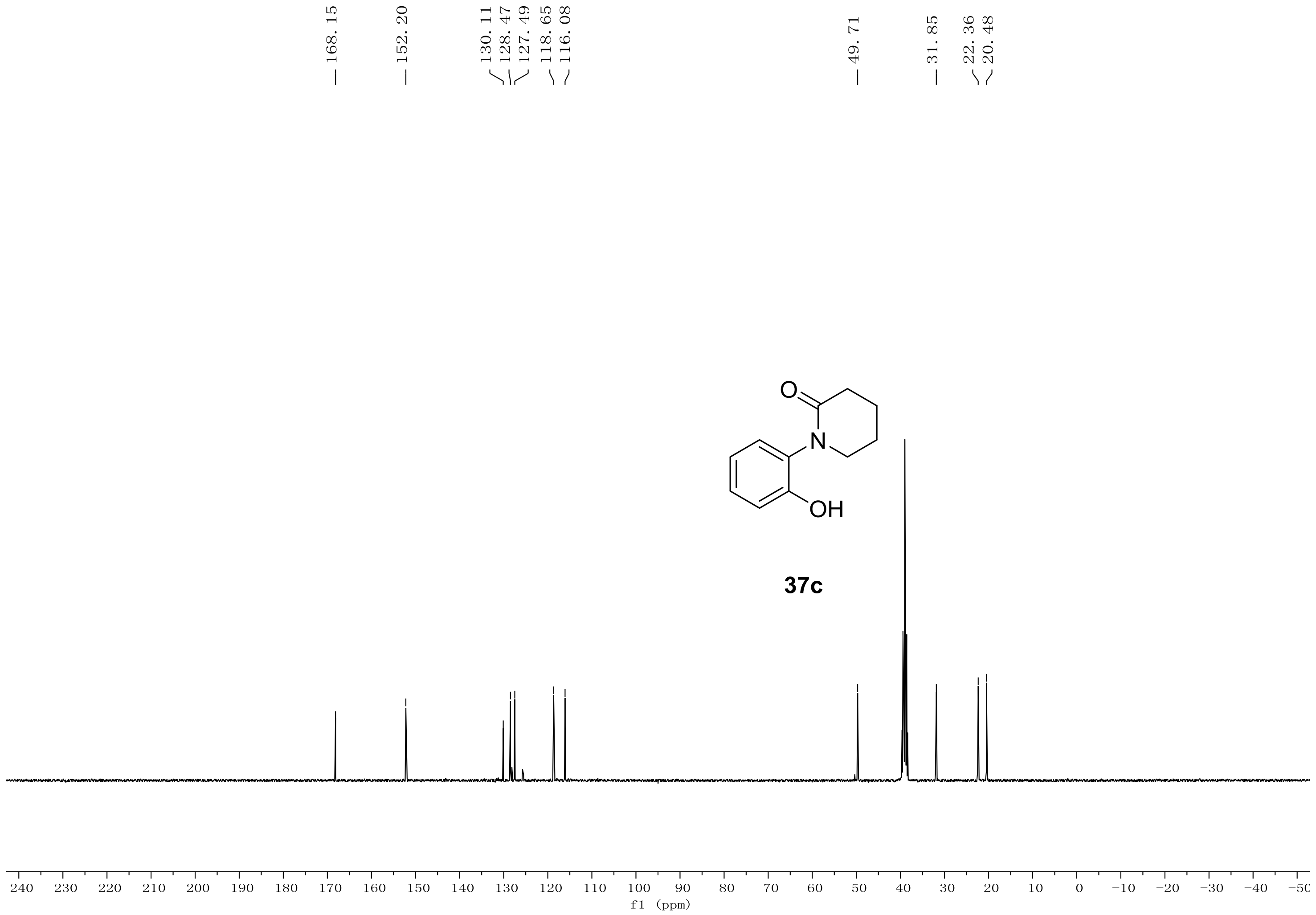

Supplementary Figure 78. ^{13}C NMR spectrum for **37c**

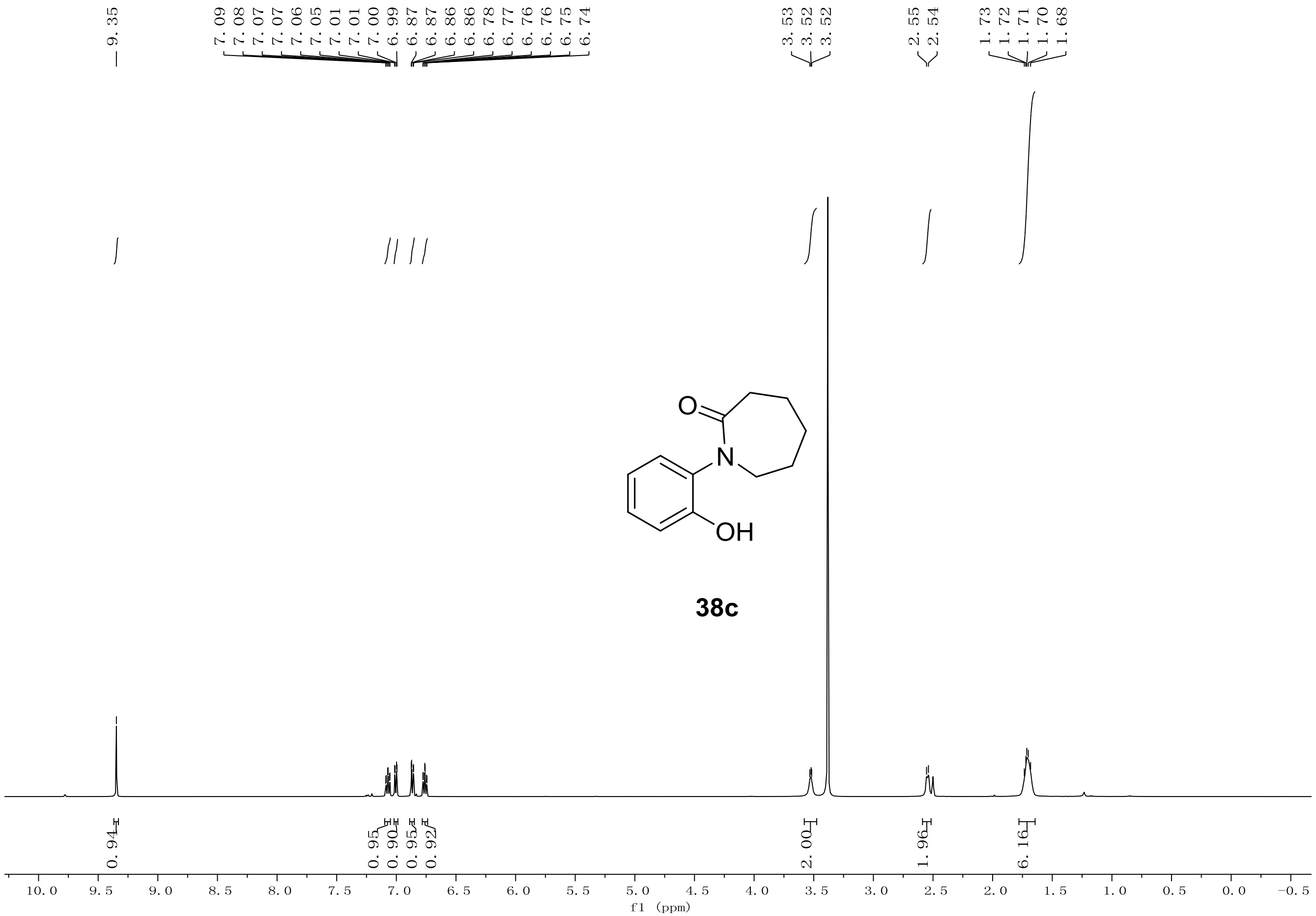

Supplementary Figure 79. ^{1}H NMR spectrum for **38c**

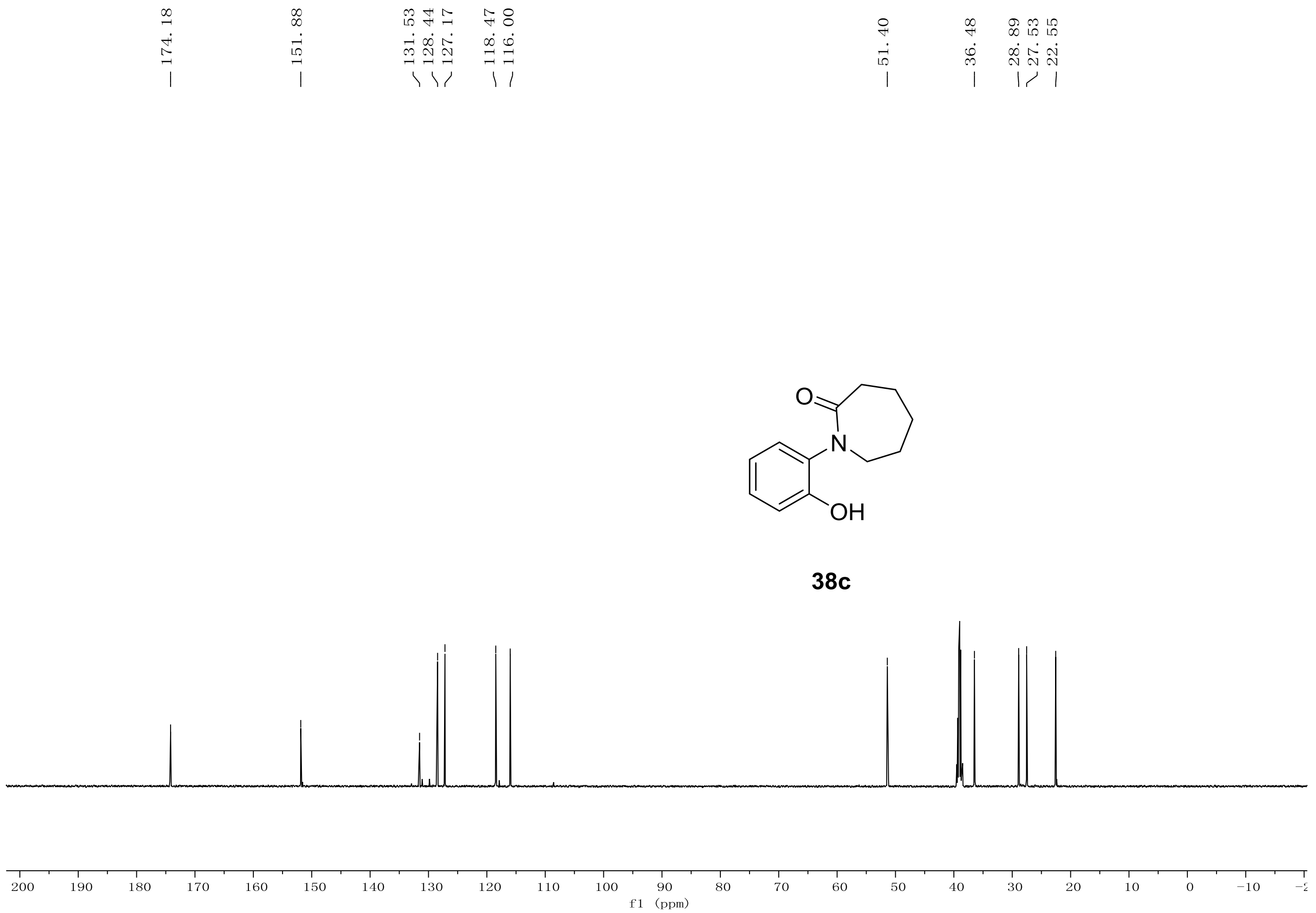

Supplementary Figure 80. ^{13}C NMR spectrum for **38c**

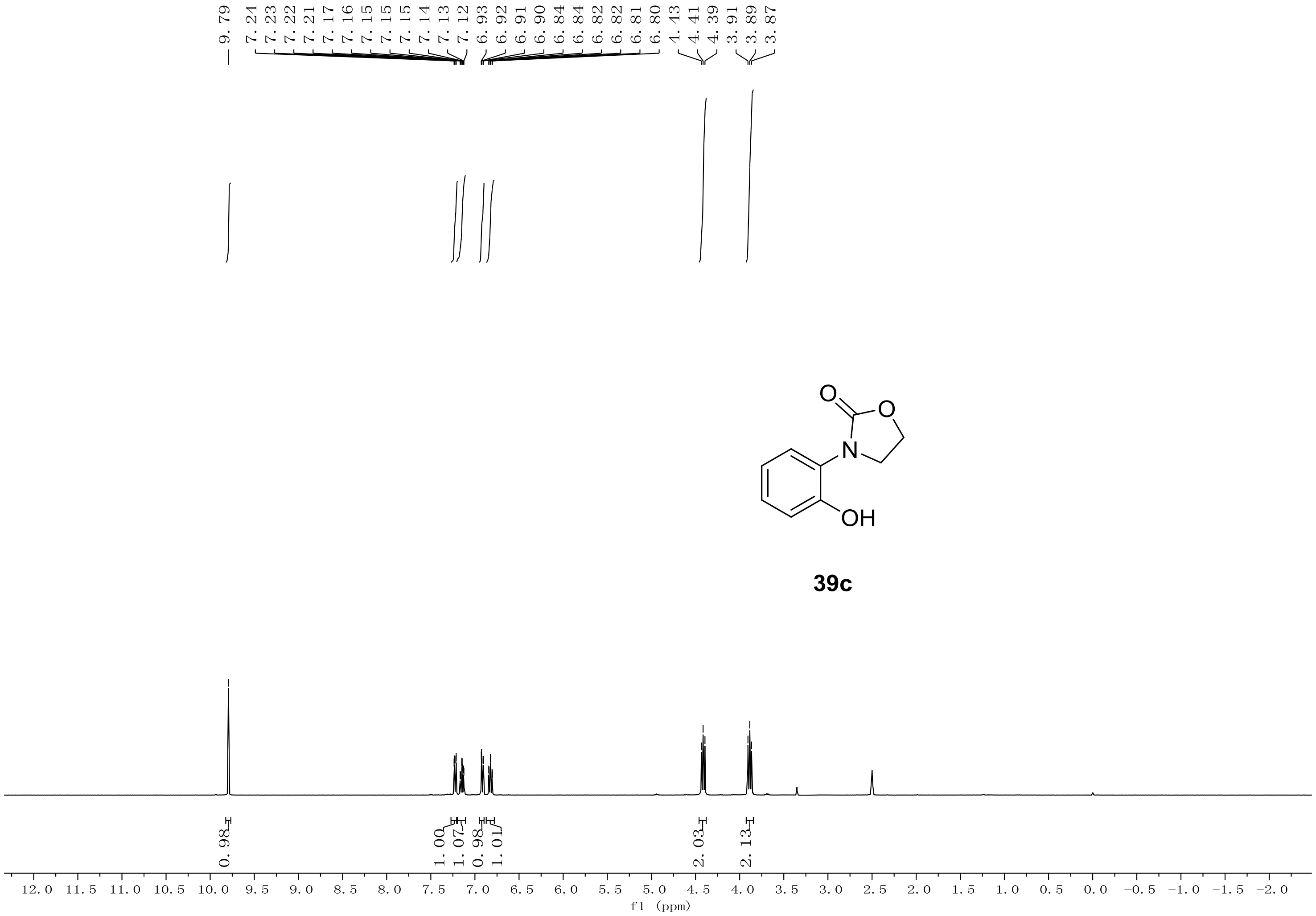

Supplementary Figure 81. ^{1}H NMR spectrum for **39c**

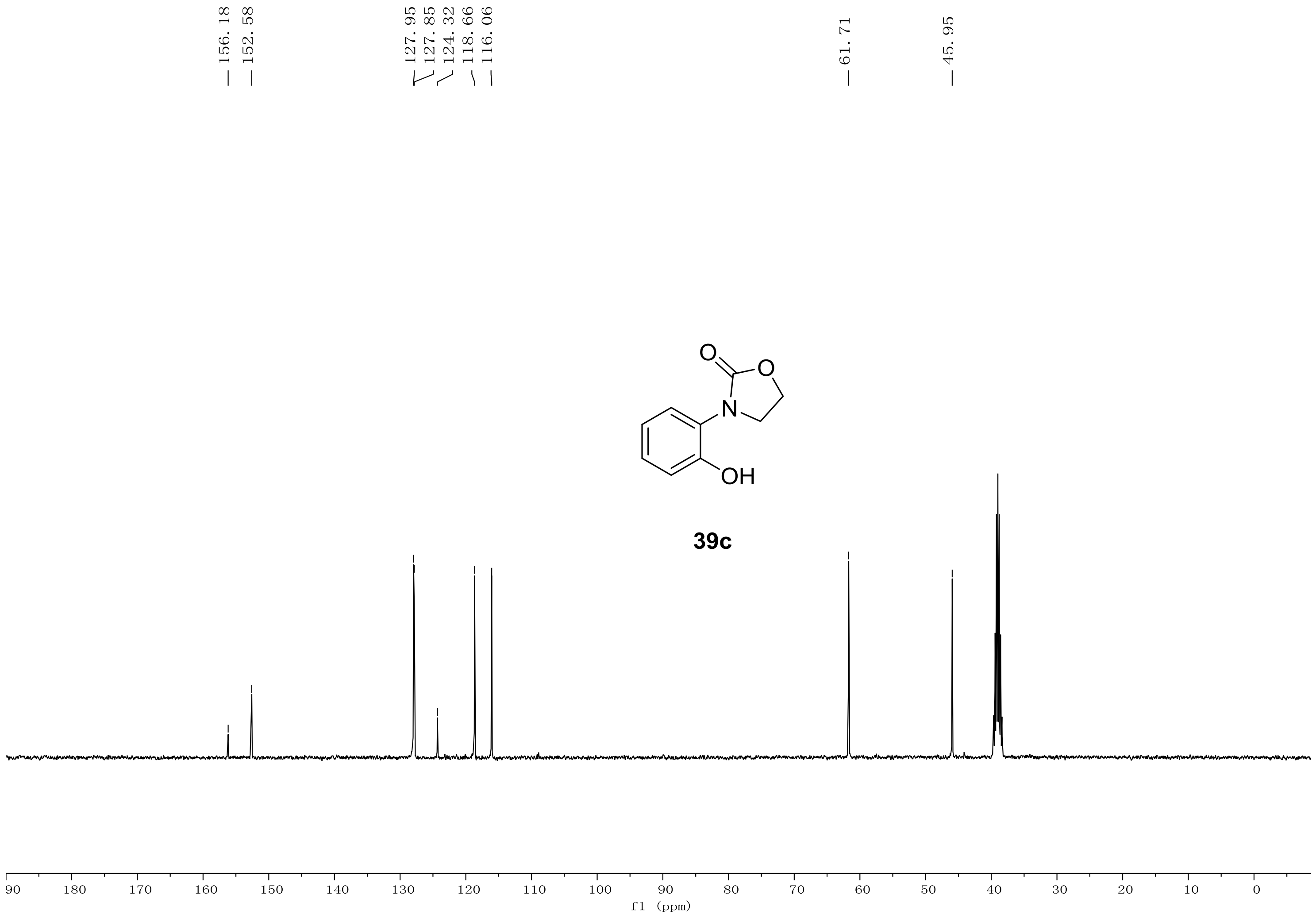

Supplementary Figure 82. ^{13}C NMR spectrum for **39c**

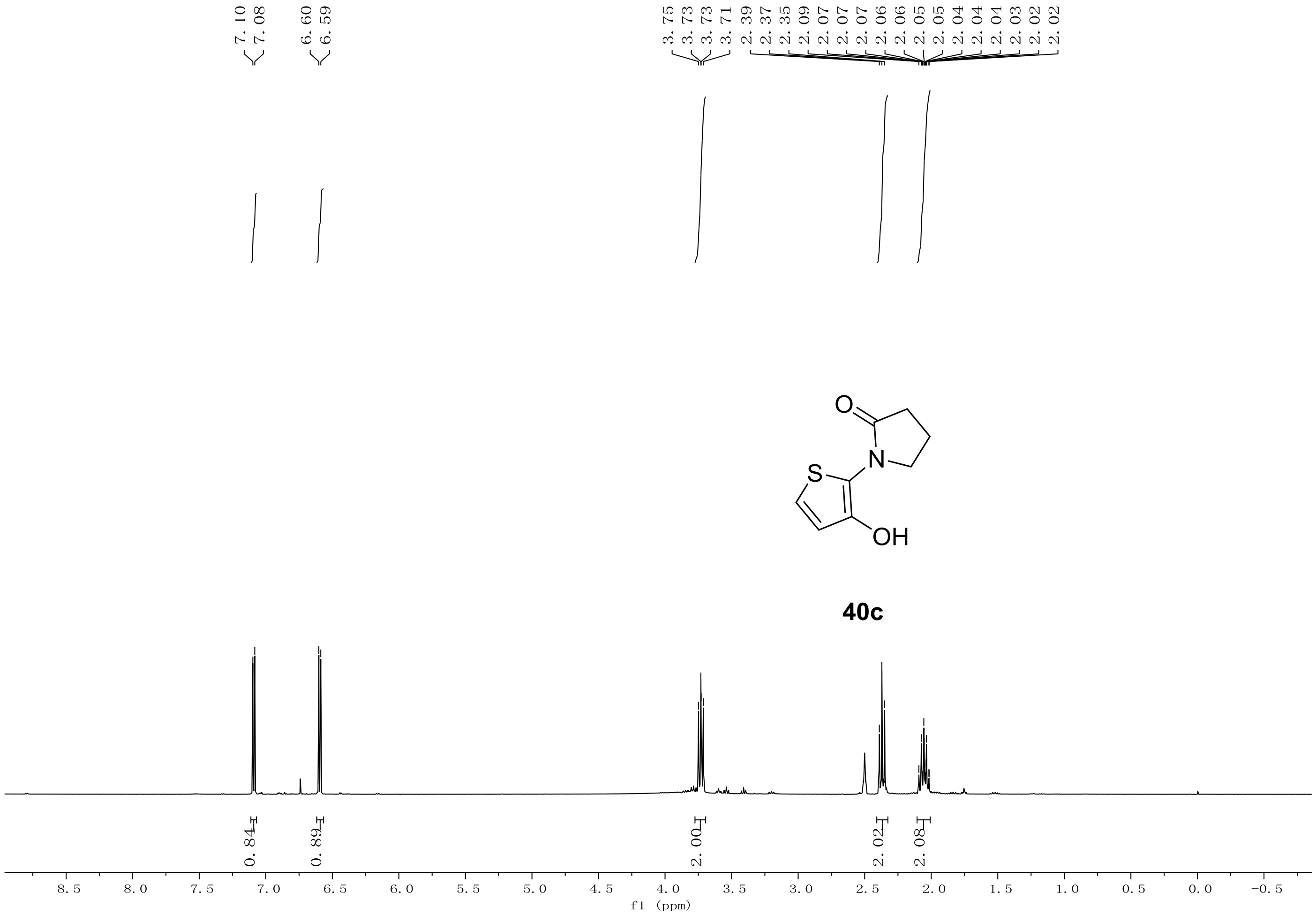

Supplementary Figure 83. ^{1}H NMR spectrum for **4c**

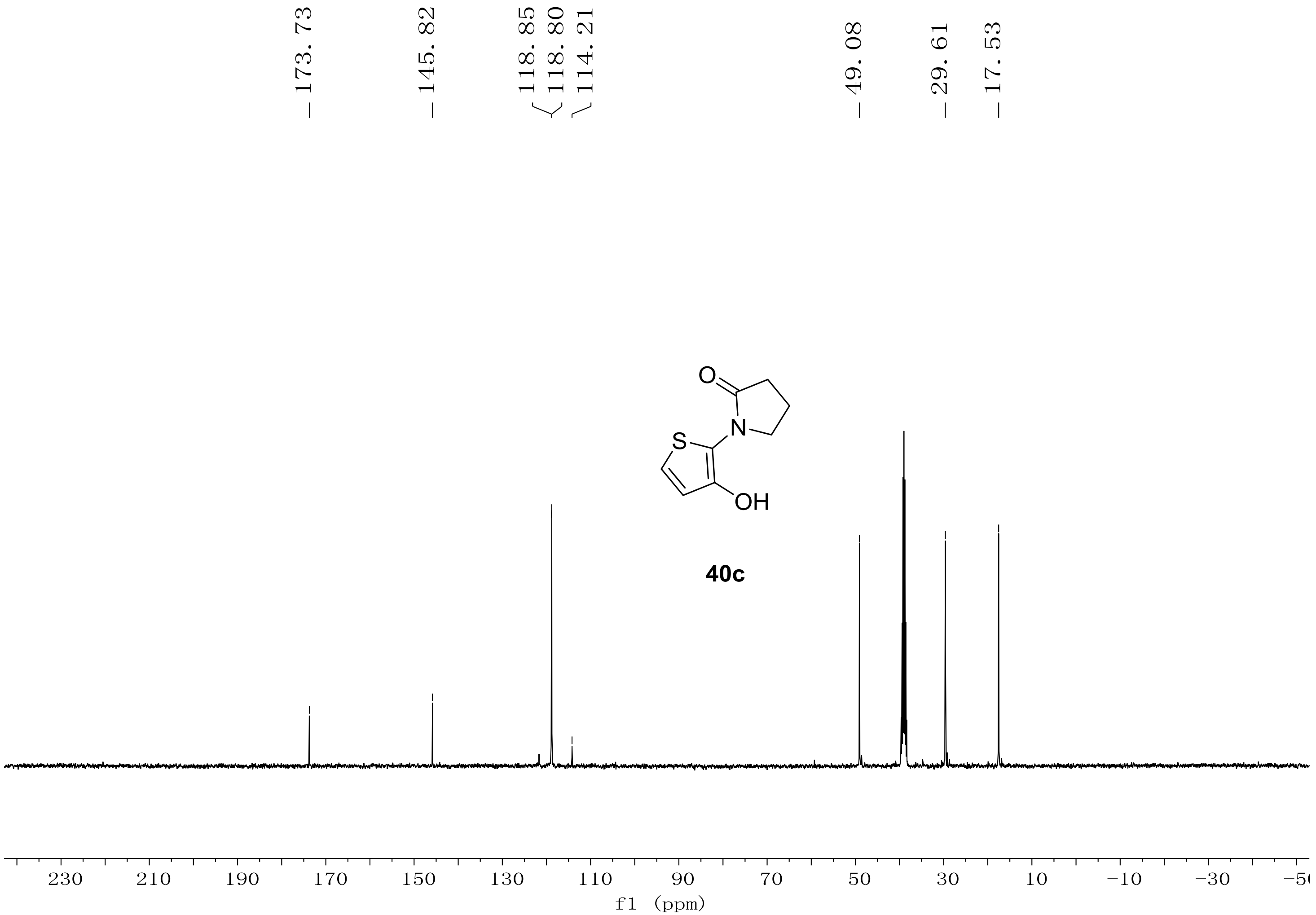

Supplementary Figure 84. ^{13}C NMR spectrum for **40c**

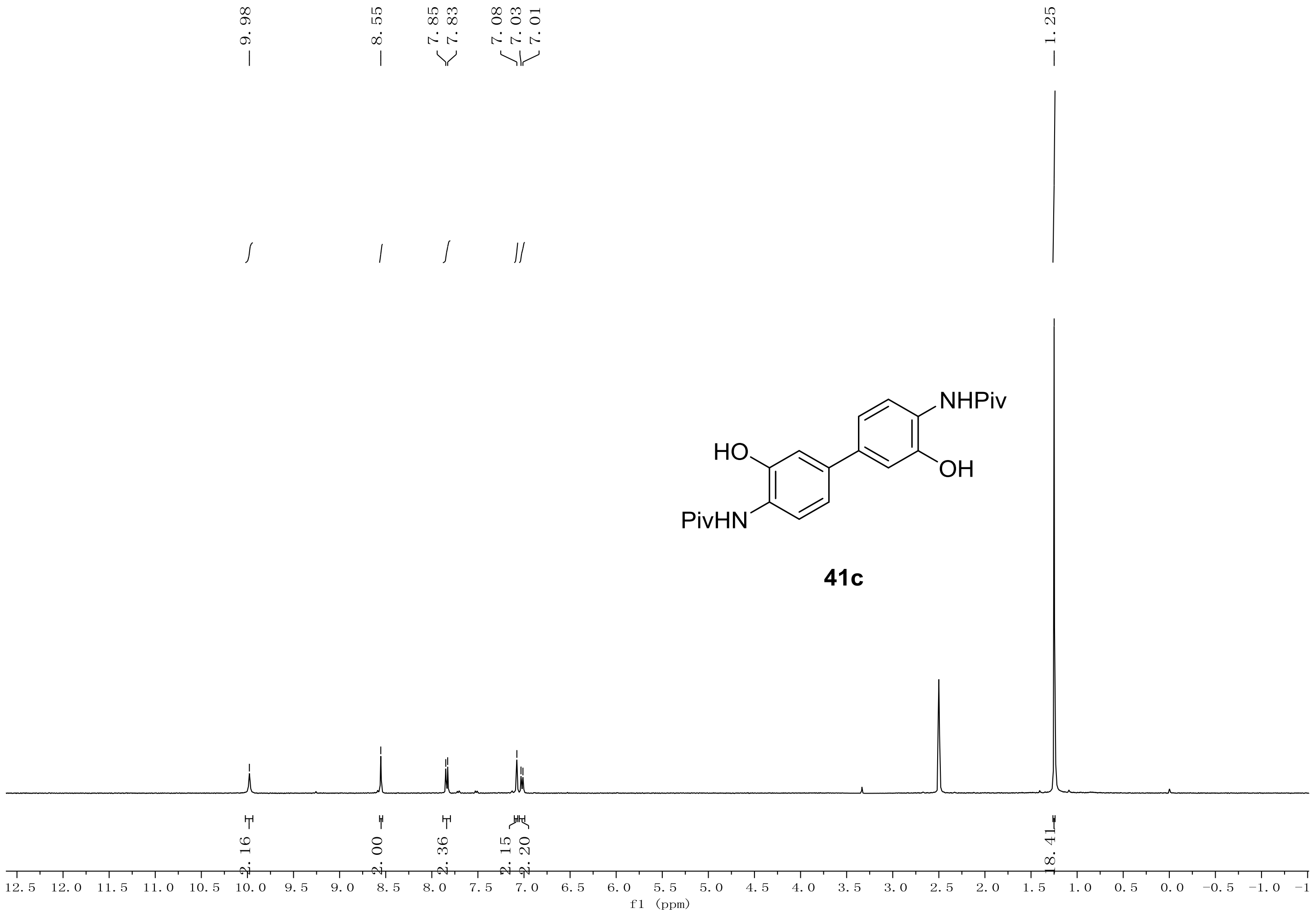

Supplementary Figure 85. ^{1}H NMR spectrum for **41c**

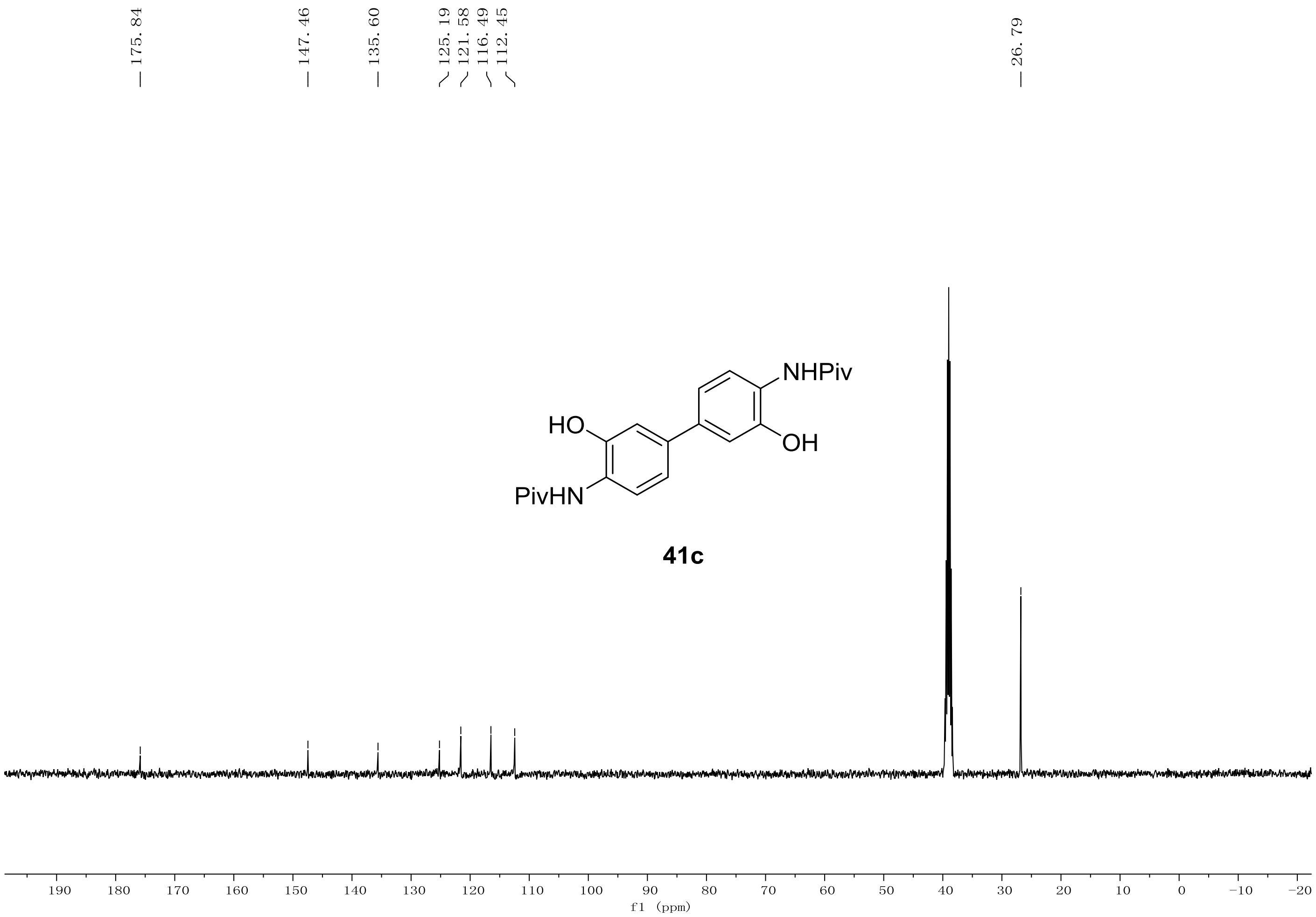

Supplementary Figure 86. ^{13}C NMR spectrum for **41c**

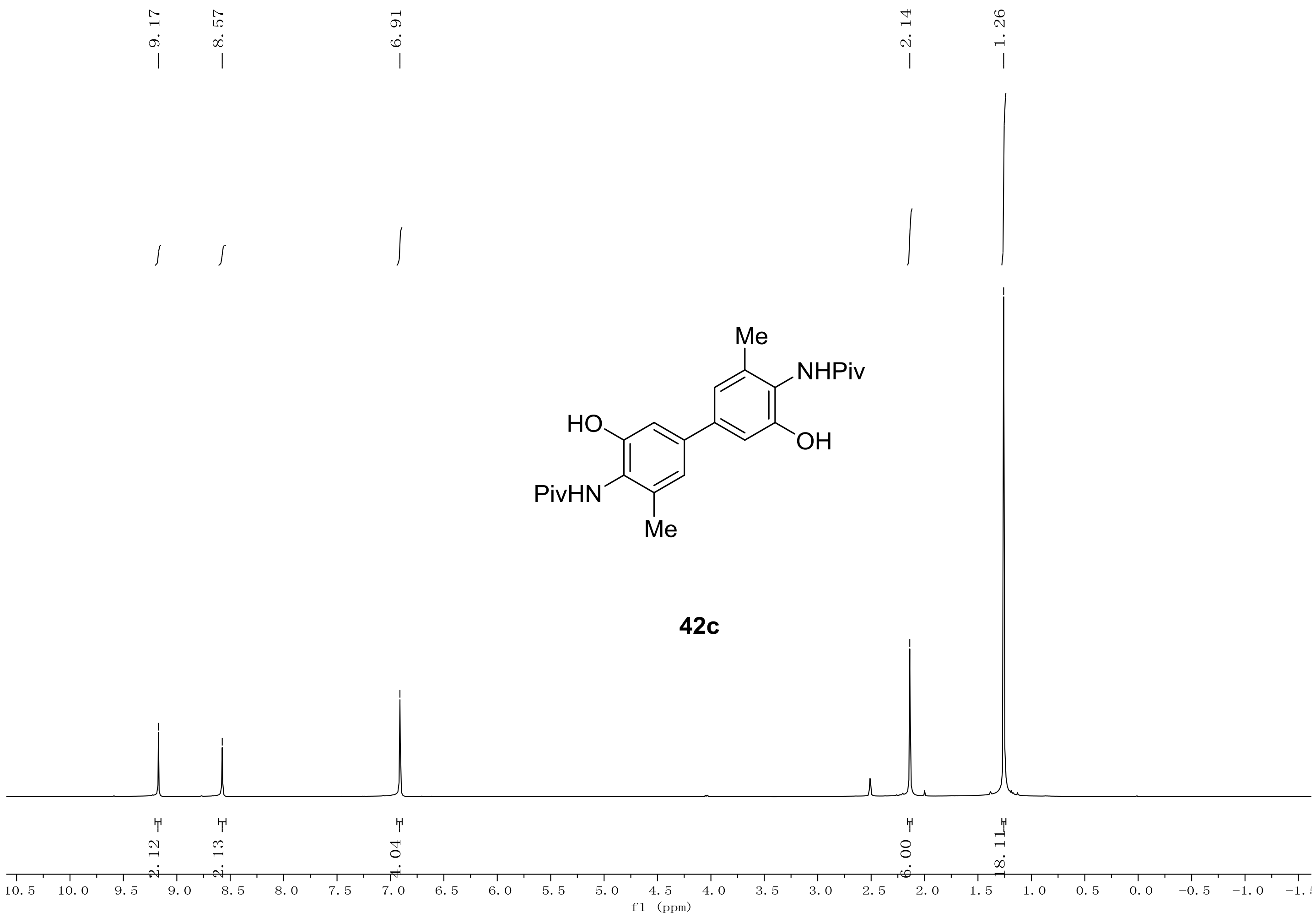

Supplementary Figure 87. ^{1}H NMR spectrum for **42c**

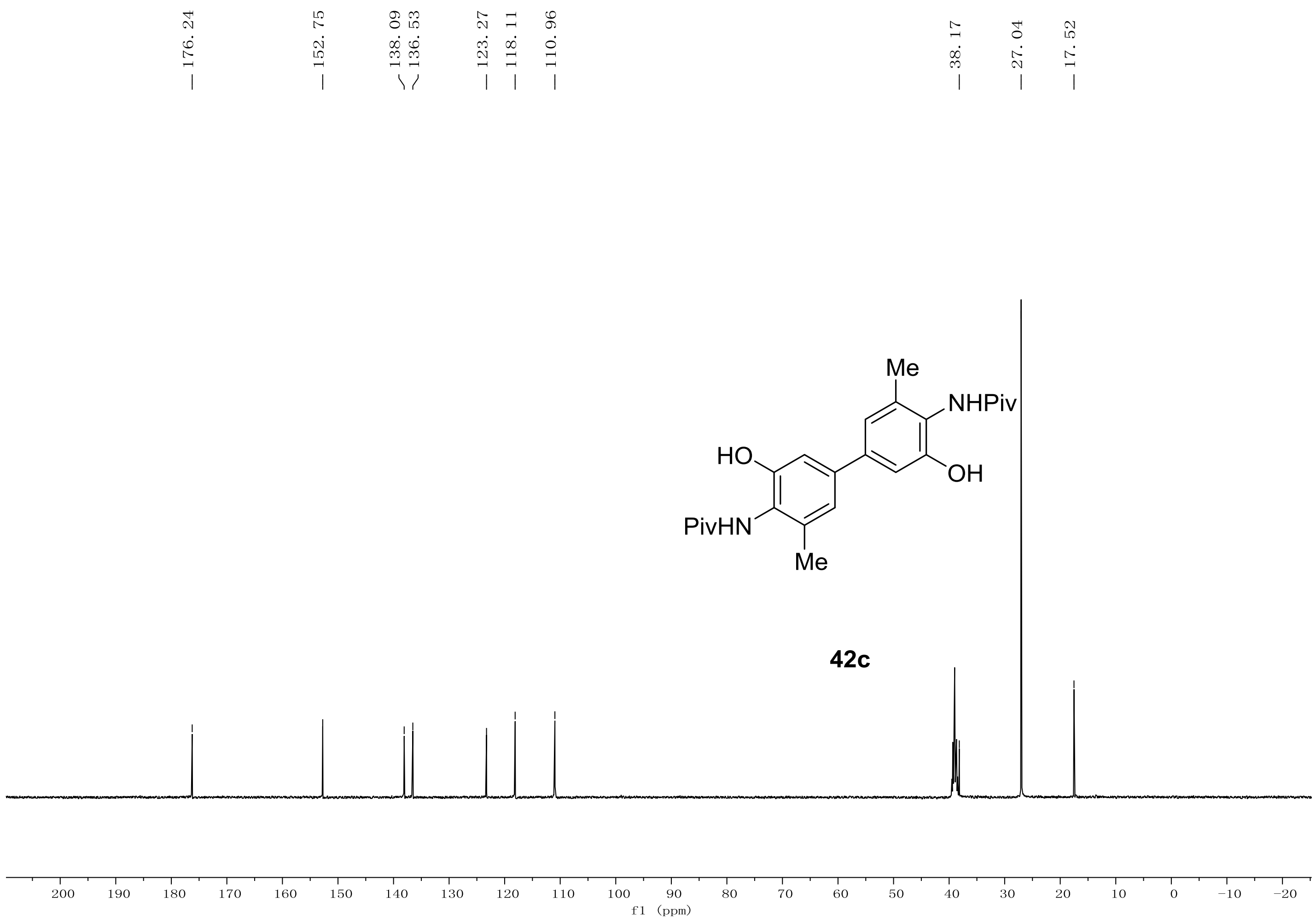

Supplementary Figure 88. ^{13}C NMR spectrum for **42c**

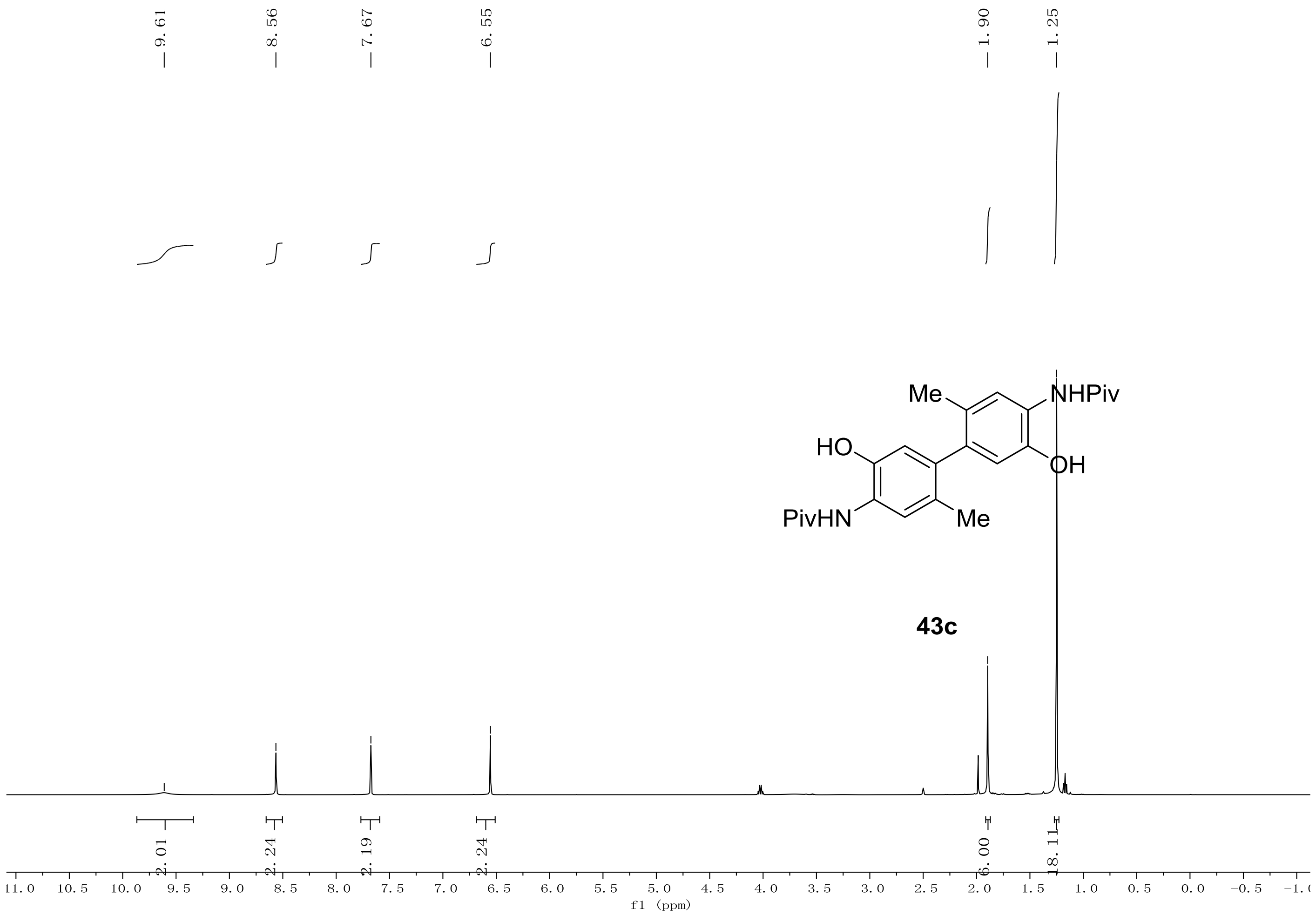

Supplementary Figure 89. ^{1}H NMR spectrum for **43c**

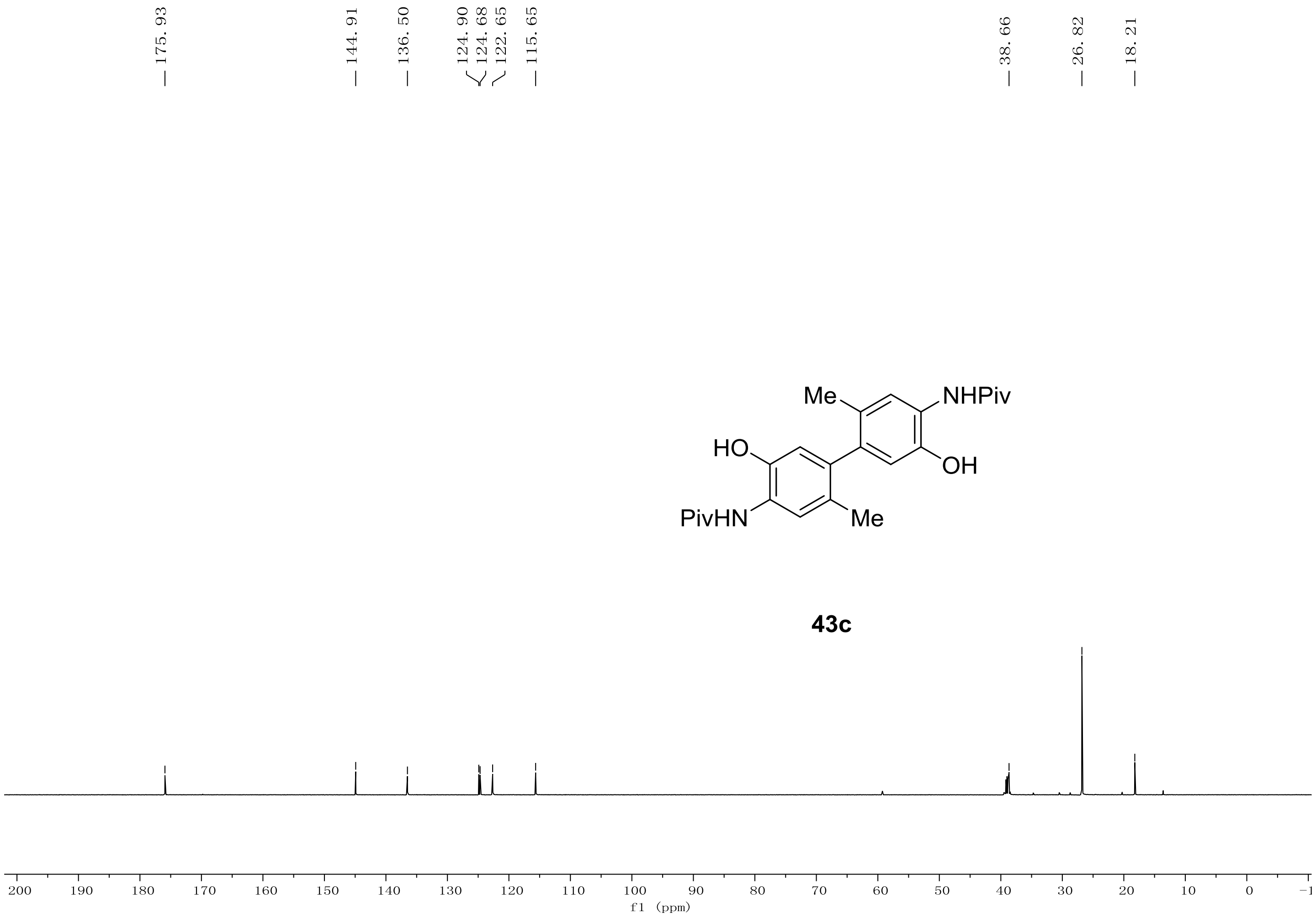

Supplementary Figure 90. ^{13}C NMR spectrum for **43c**

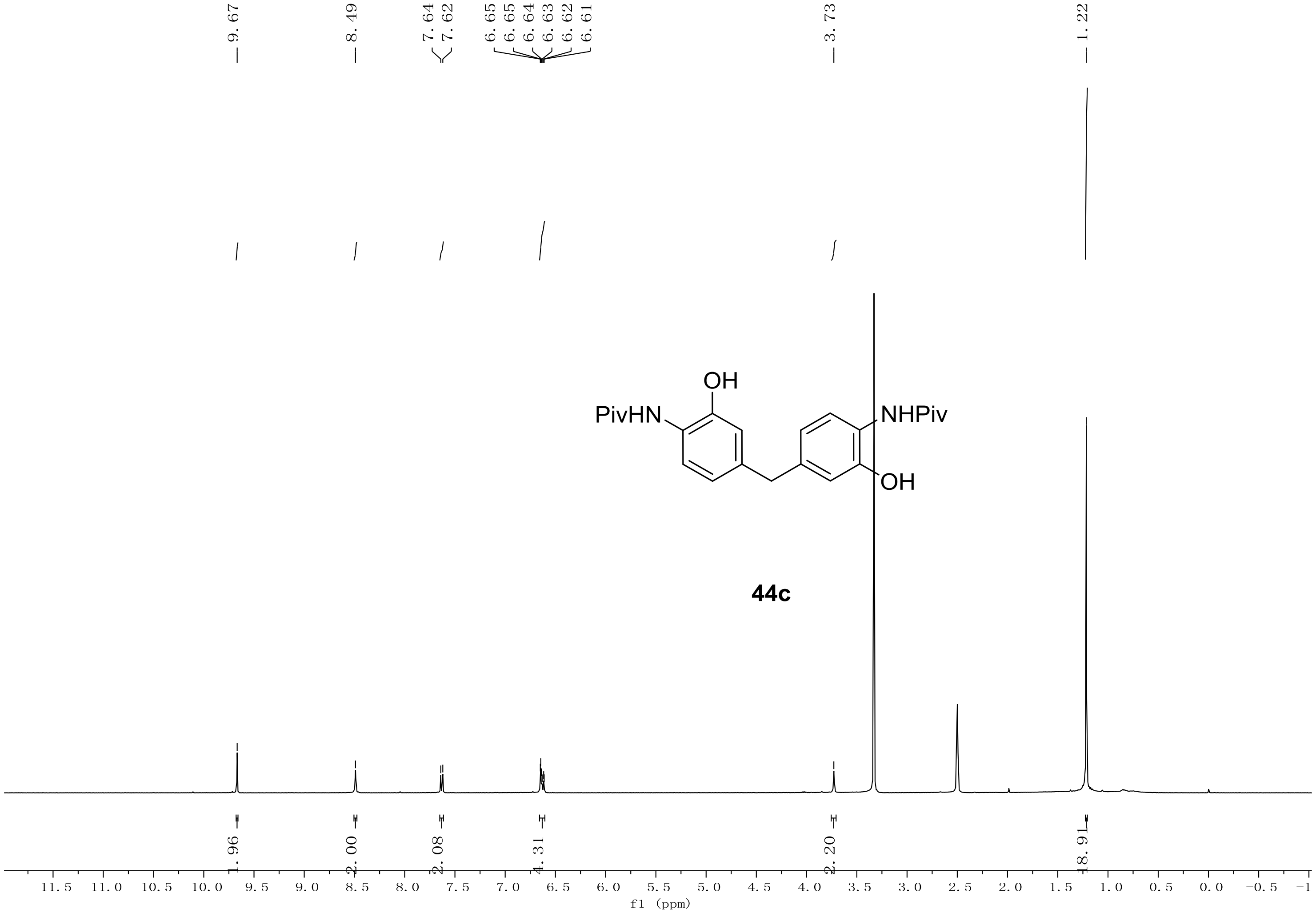

Supplementary Figure 91. ^{1}H NMR spectrum for **44c**

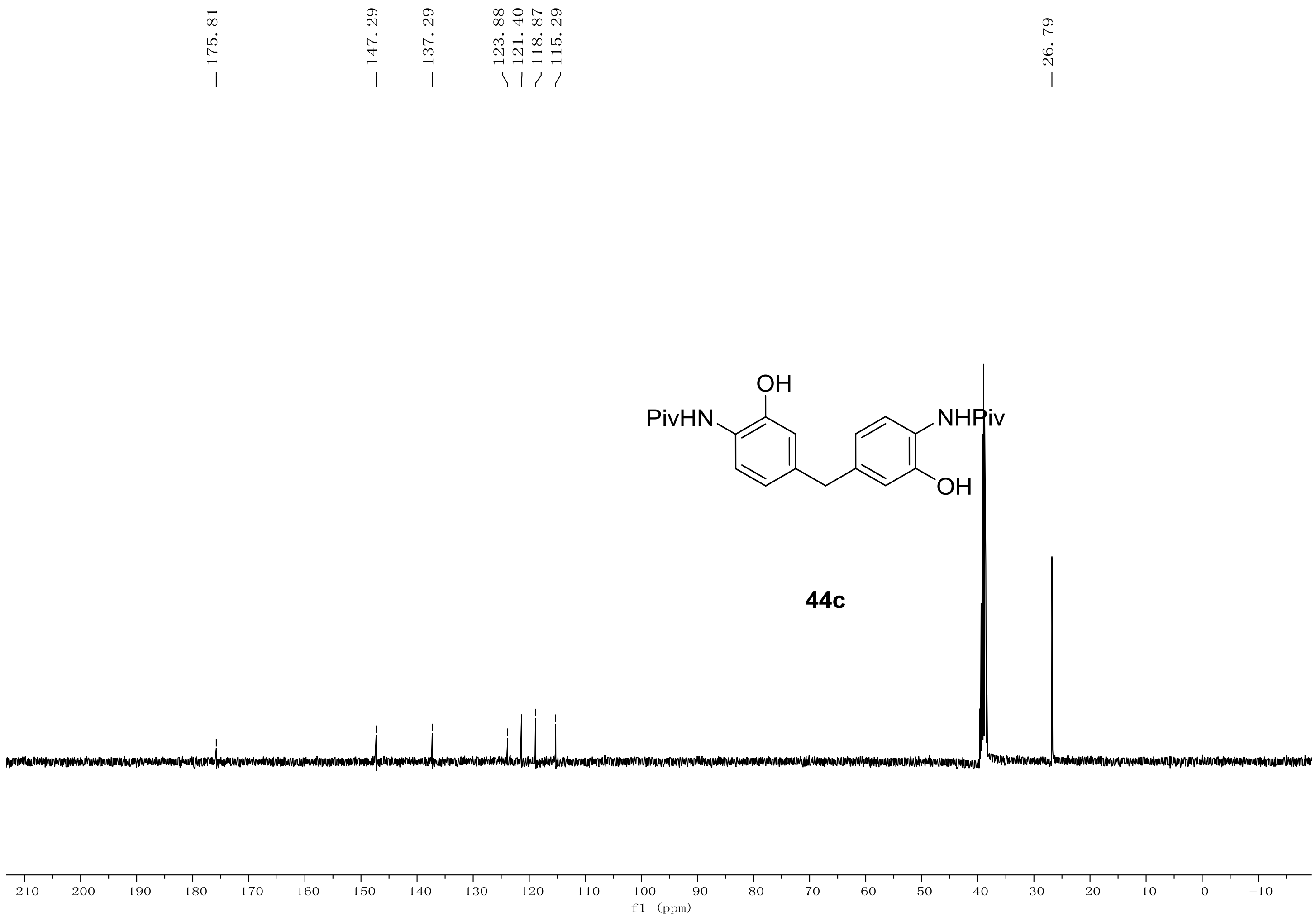

Supplementary Figure 82. ^{13}C NMR spectrum for **44c**

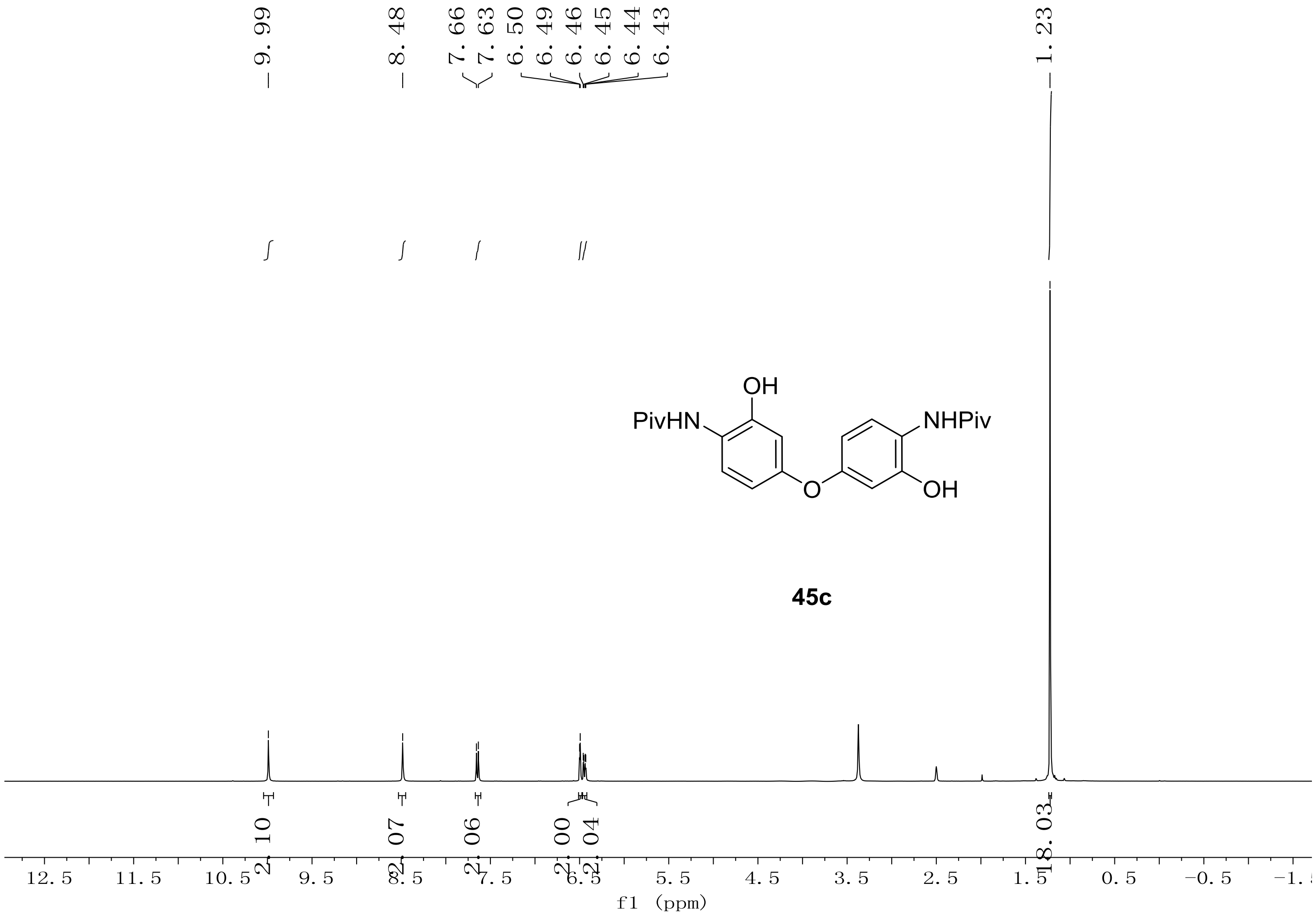

Supplementary Figure 83. ^{1}H NMR spectrum for **45c**

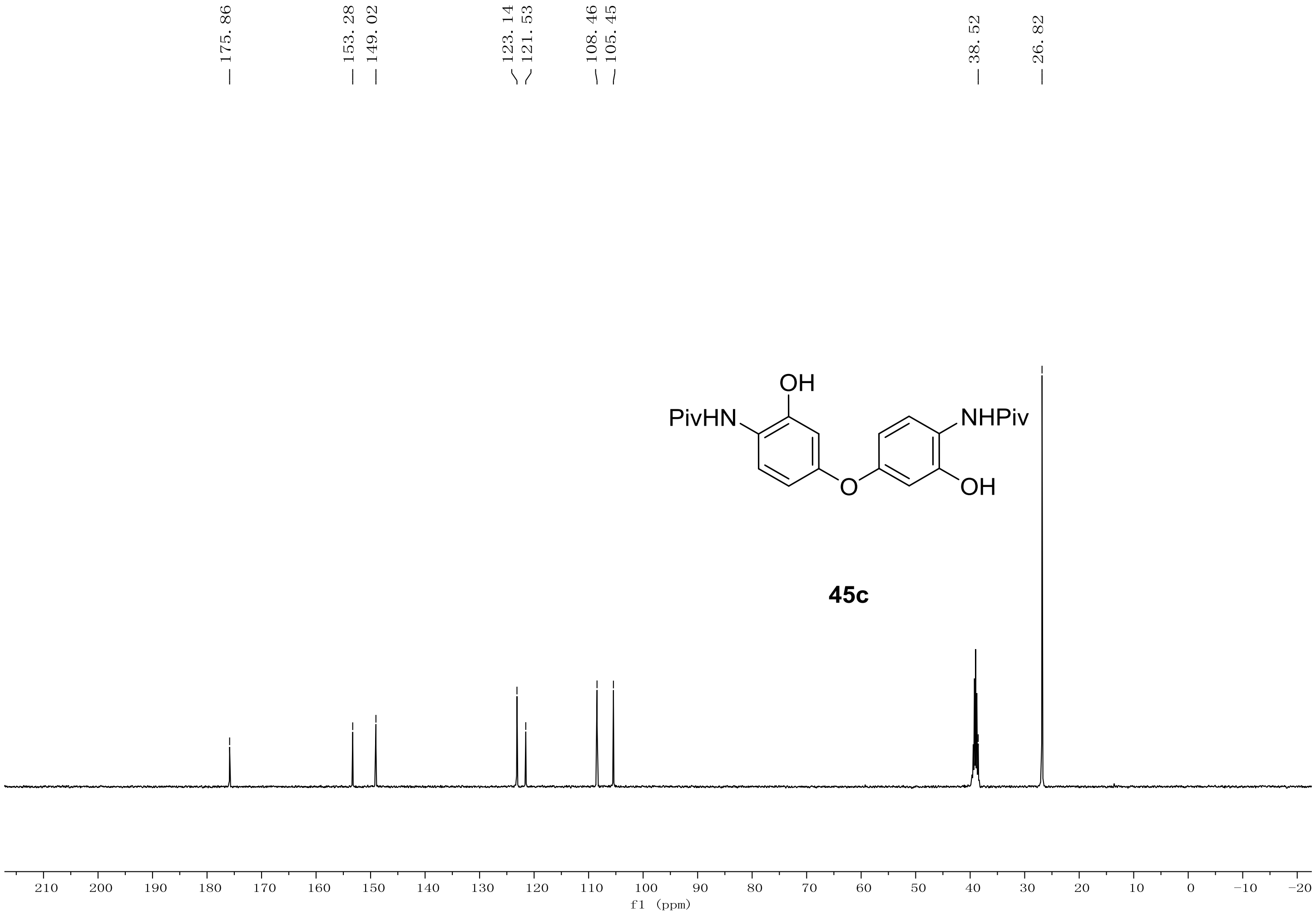

Supplementary Figure 84. ^{13}C NMR spectrum for **45c**

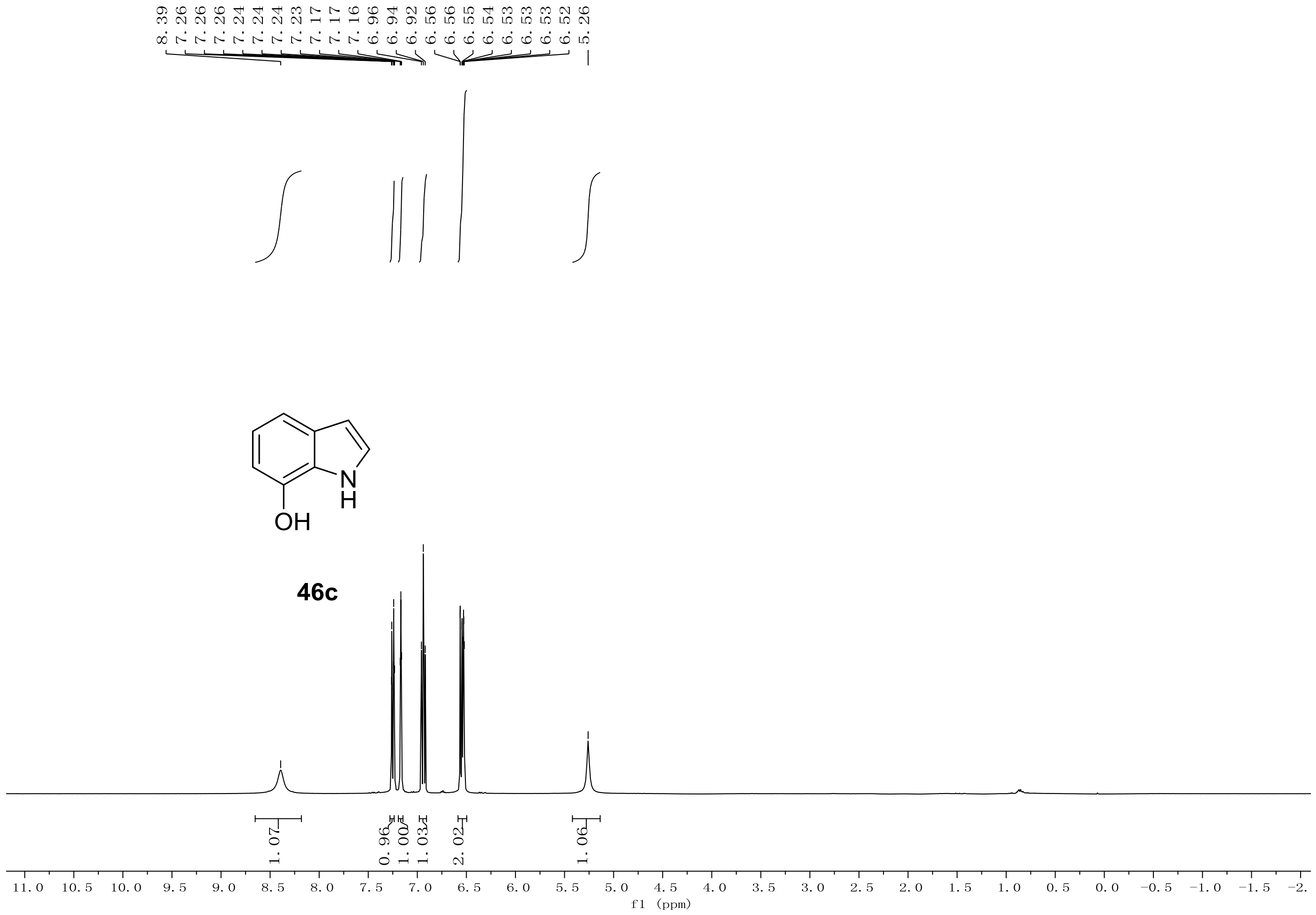

Supplementary Figure 85. ^{1}H NMR spectrum for **46c**

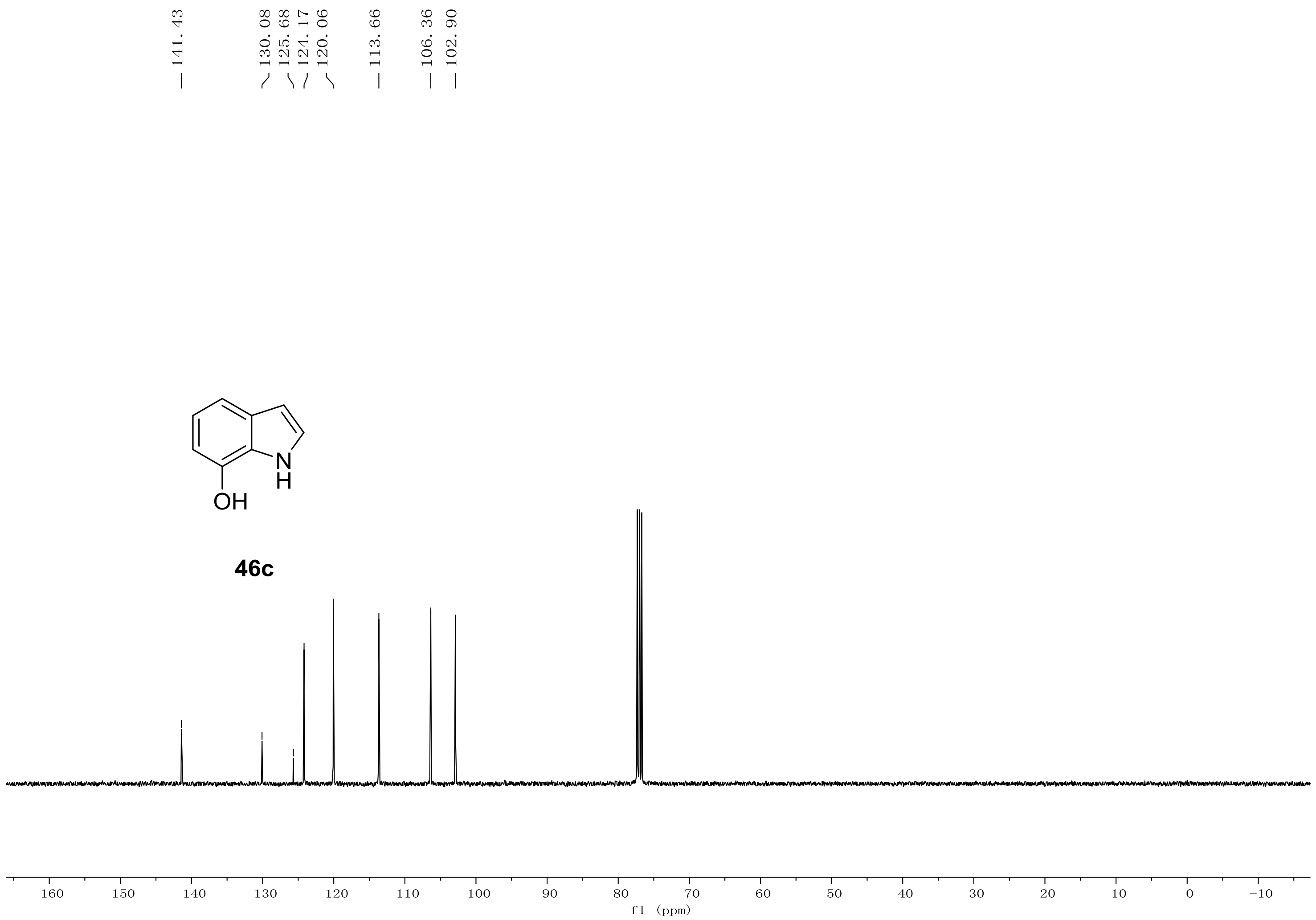

Supplementary Figure 86. ^{13}C NMR spectrum for **46c**

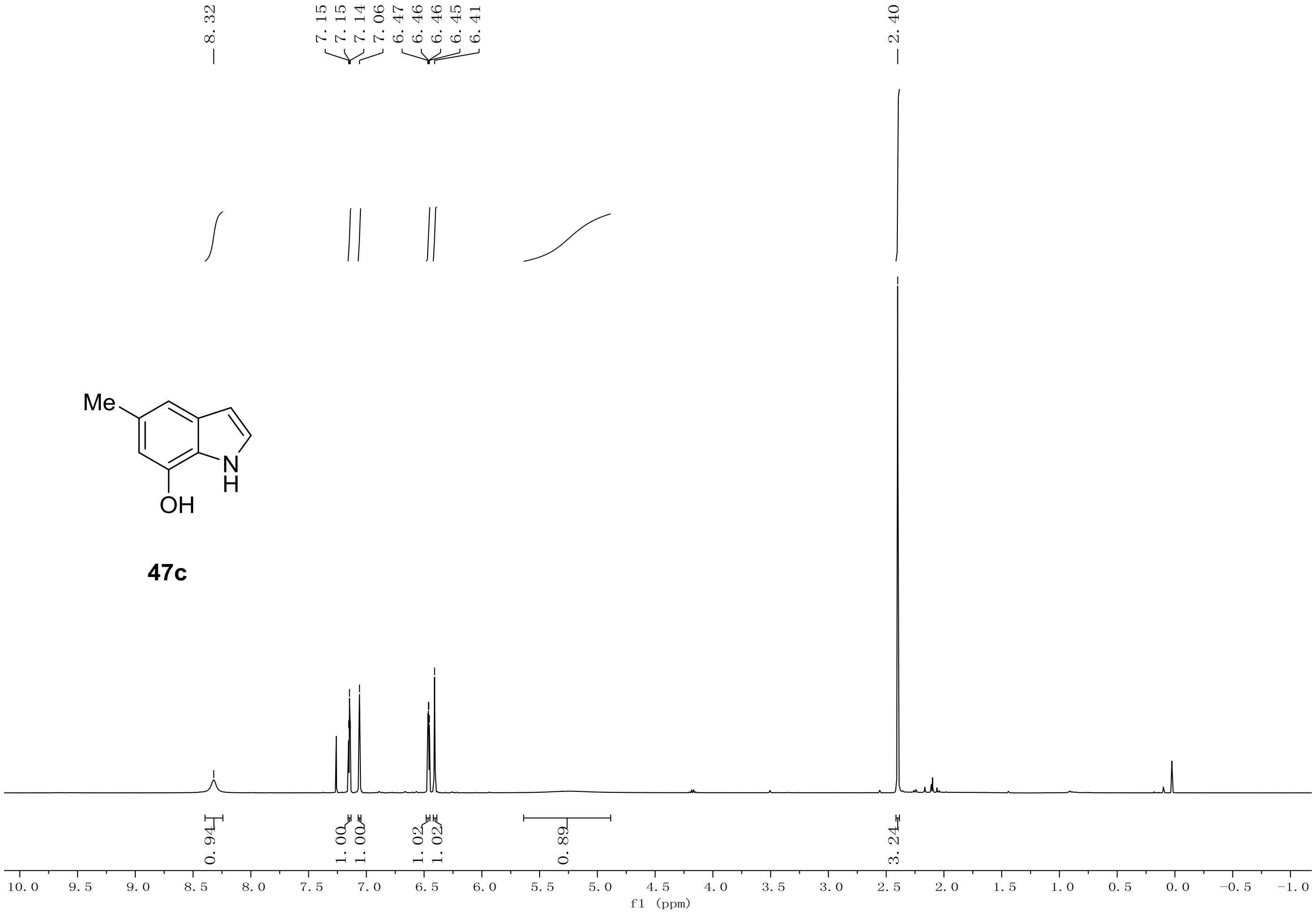

Supplementary Figure 87. ^{1}H NMR spectrum for **47c**

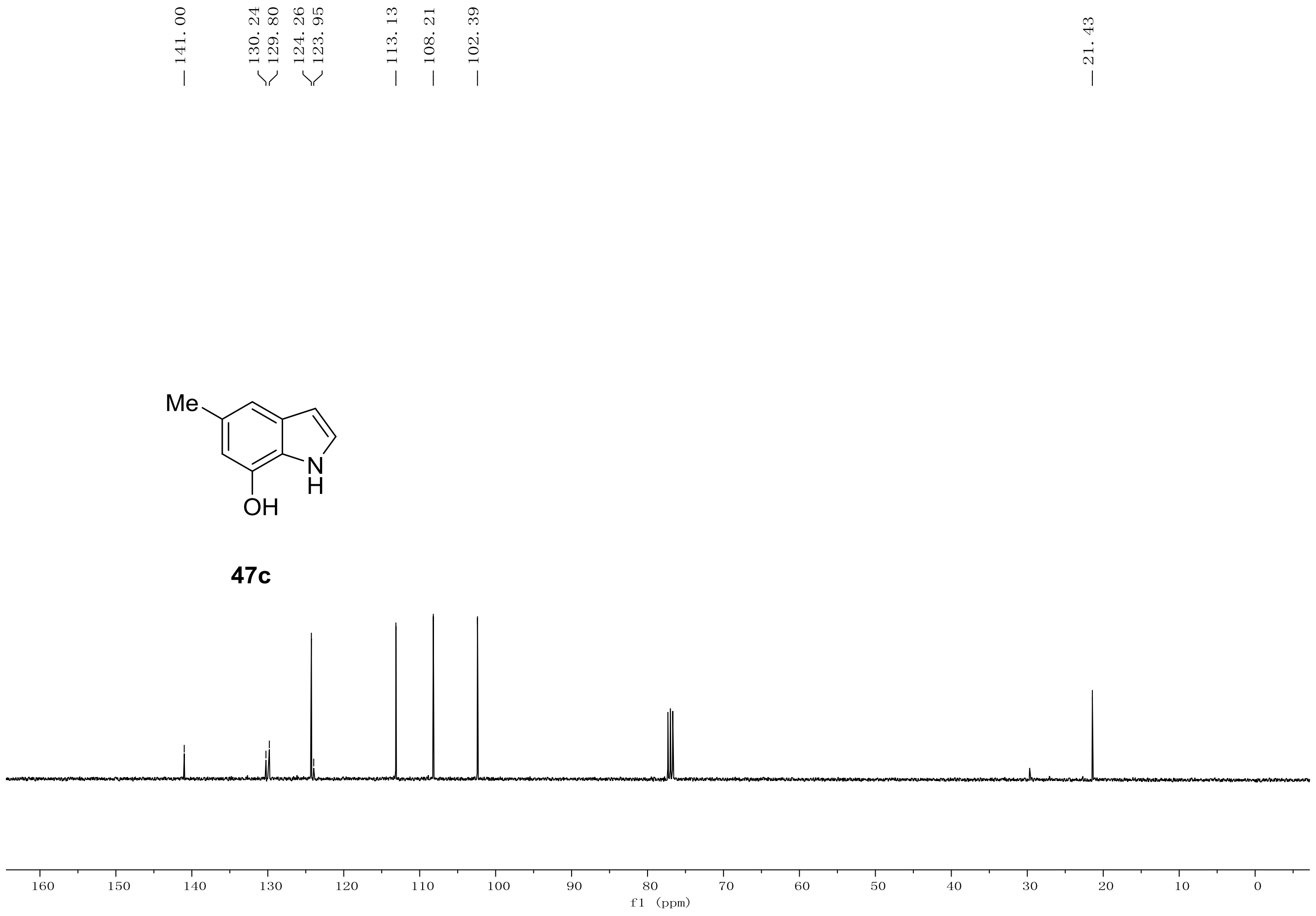

47c

Supplementary Figure 88. ^{13}C NMR spectrum for **47c**

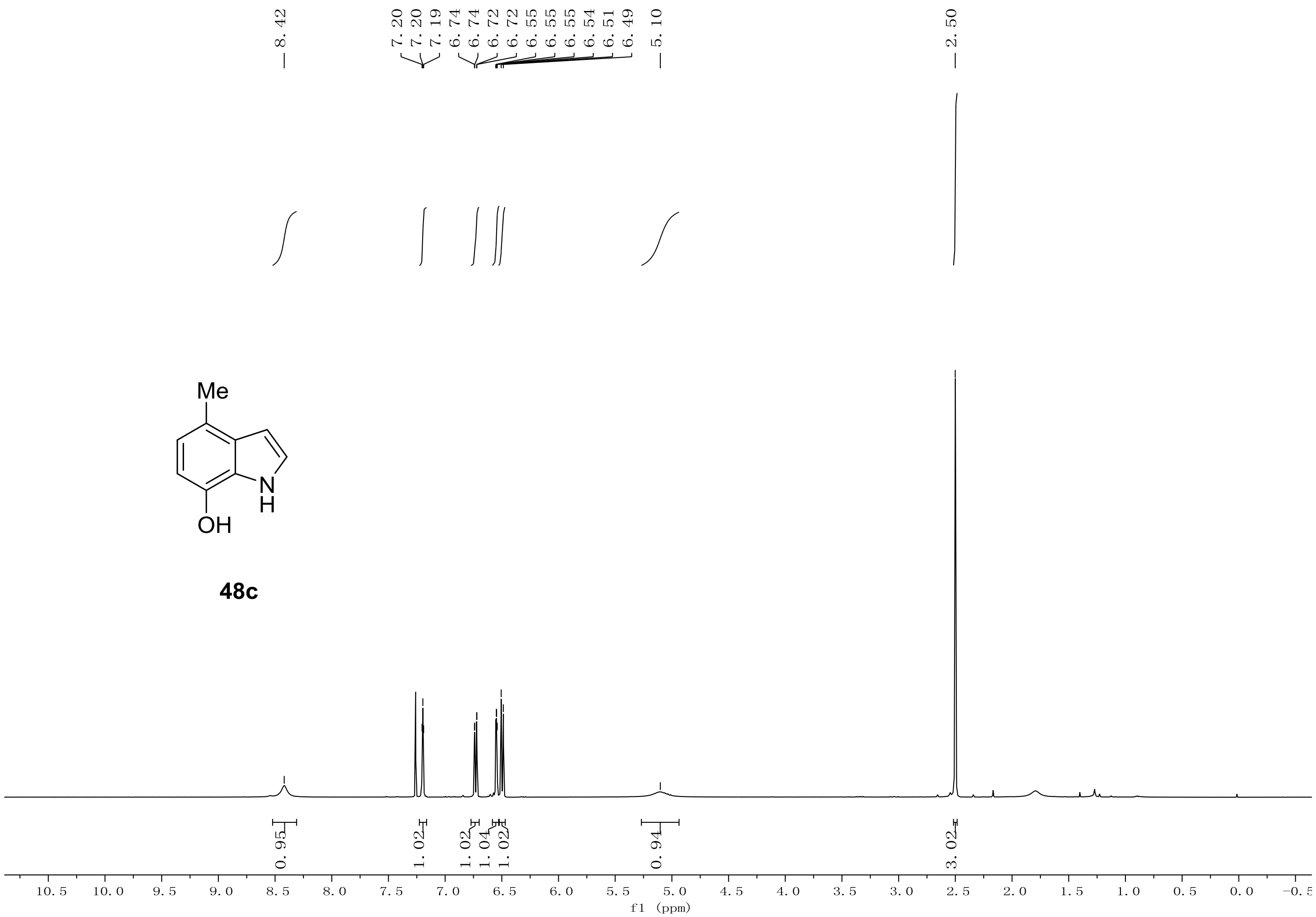

Supplementary Figure 89. ^{1}H NMR spectrum for **48c**

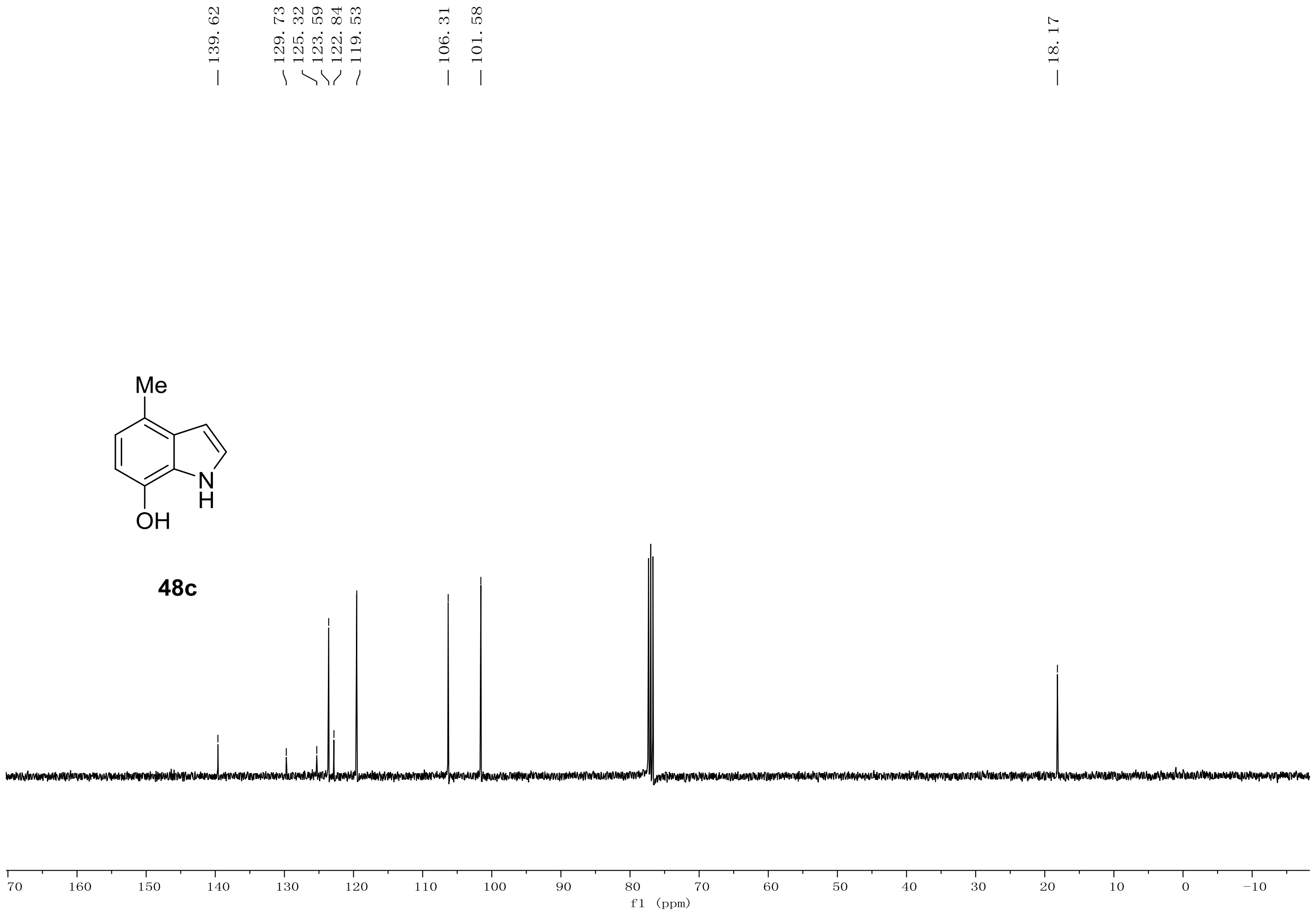

Supplementary Figure 90. ^{13}C NMR spectrum for **48c**

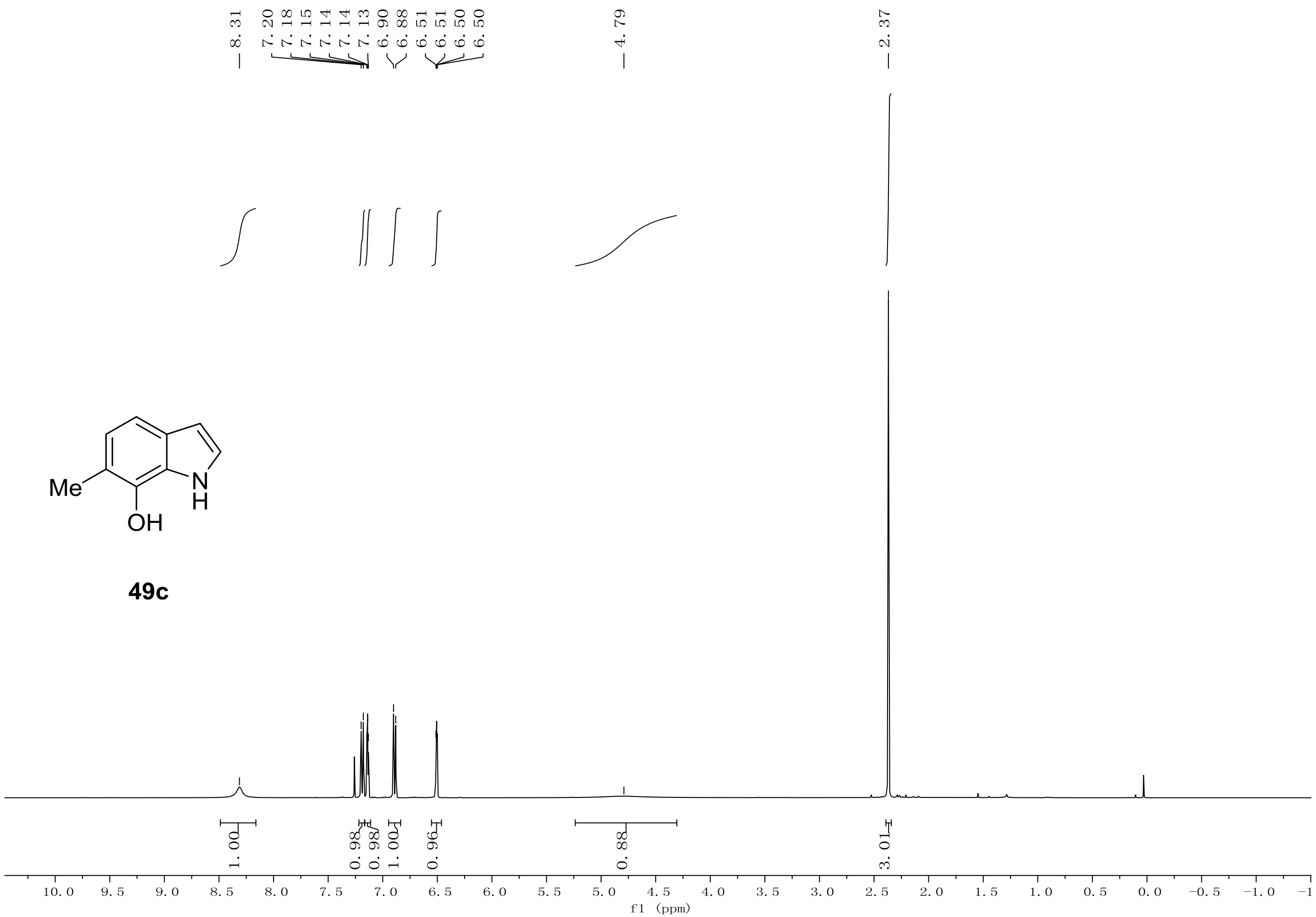

49c

Supplementary Figure 91. ^{1}H NMR spectrum for **49c**

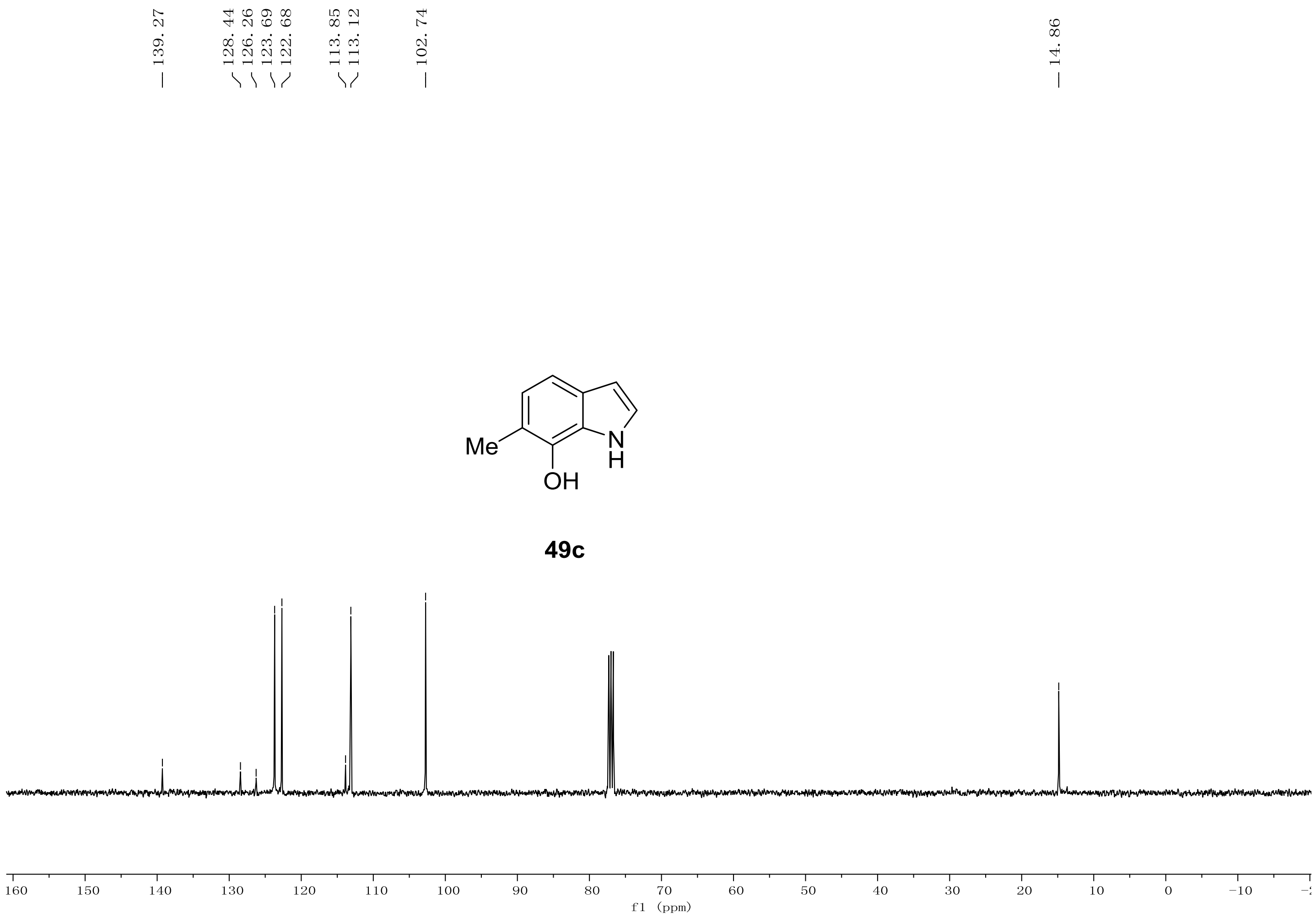

Supplementary Figure 92. ^{13}C NMR spectrum for **49c**

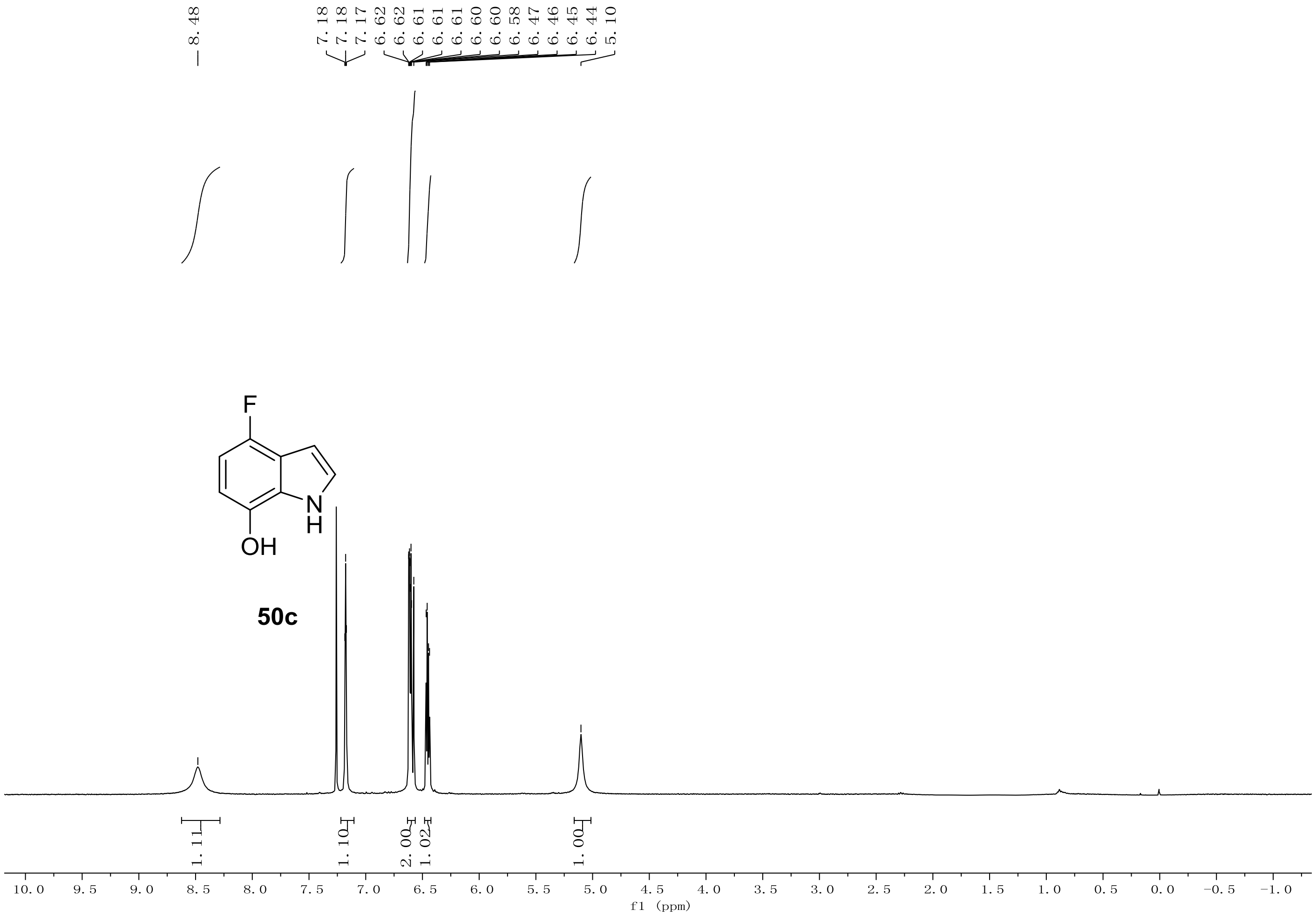

Supplementary Figure 93. ^{1}H NMR spectrum for **50c**

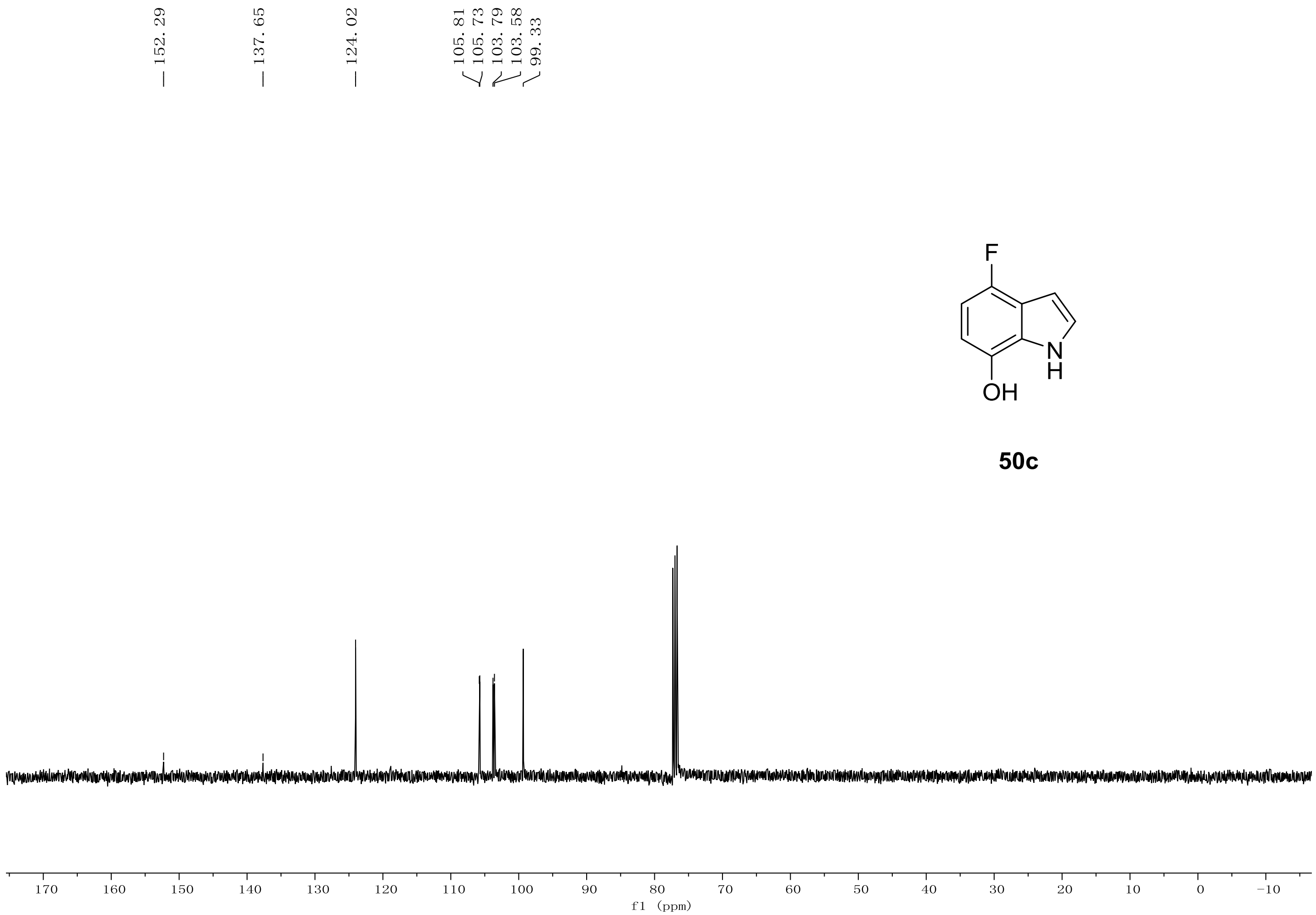

50c

Supplementary Figure 94. ^{13}C NMR spectrum for **50c**

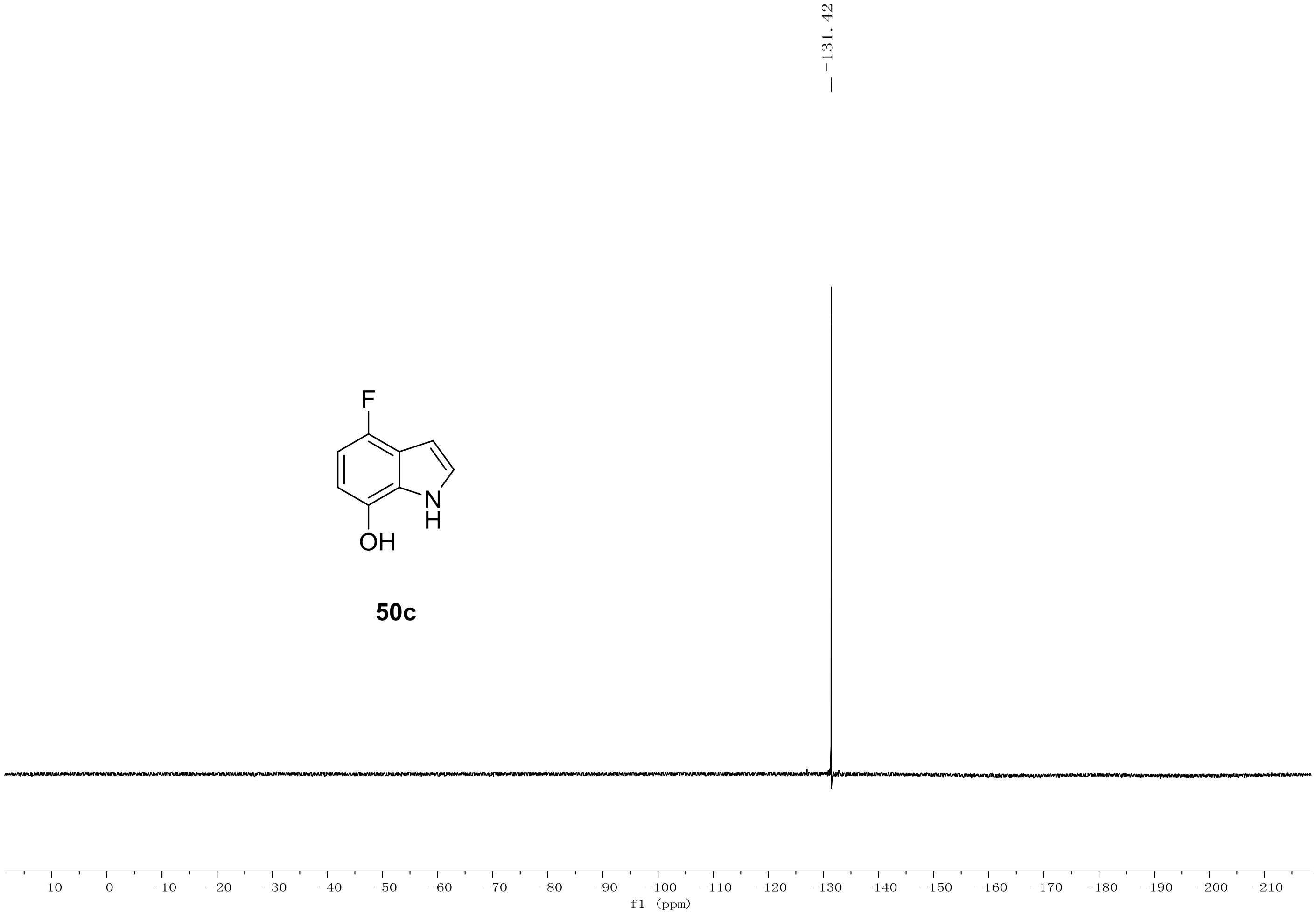

Supplementary Figure 95. ^{19}F NMR spectrum for **50c**

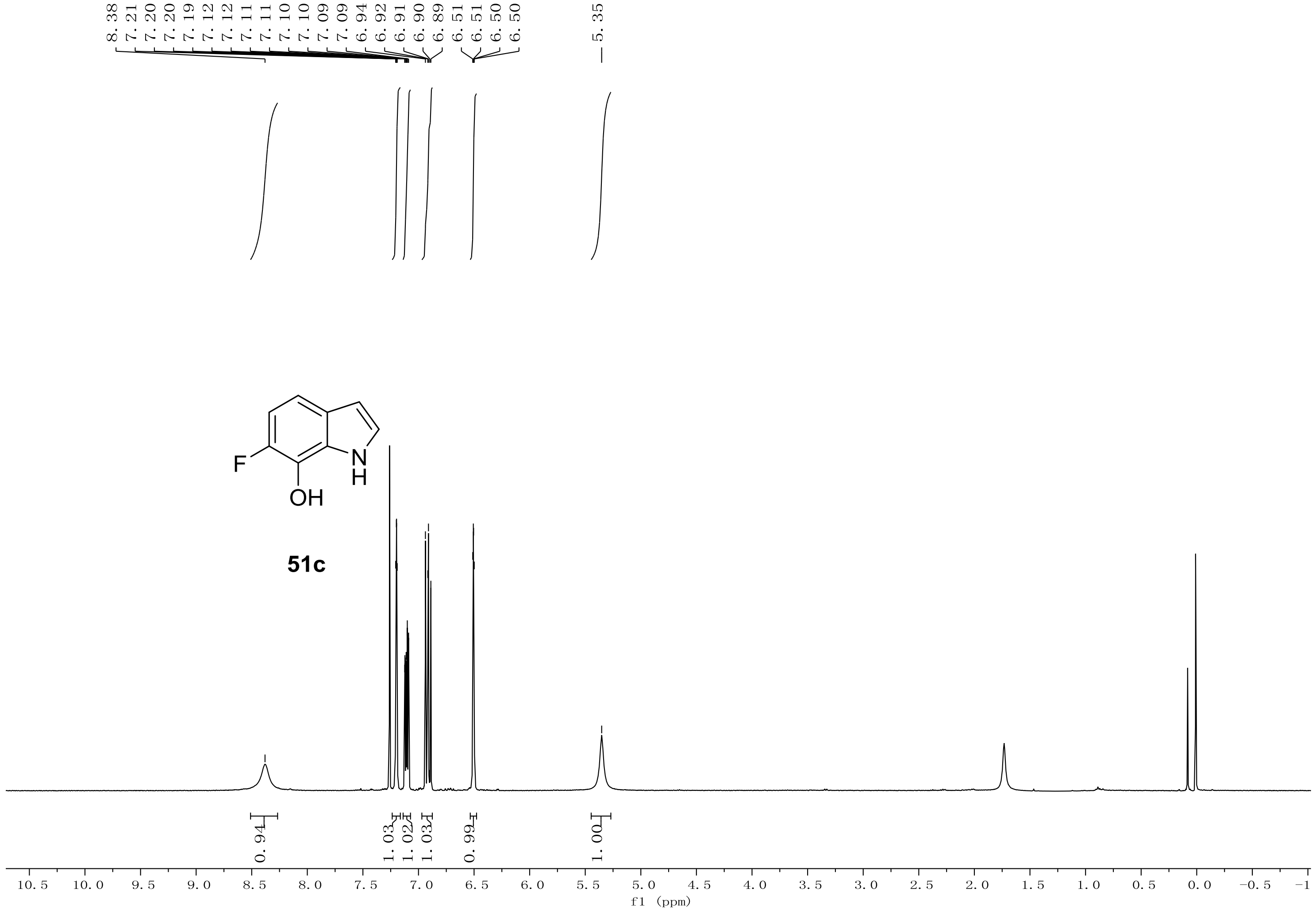

Supplementary Figure 96. ^{1}H NMR spectrum for **51c**

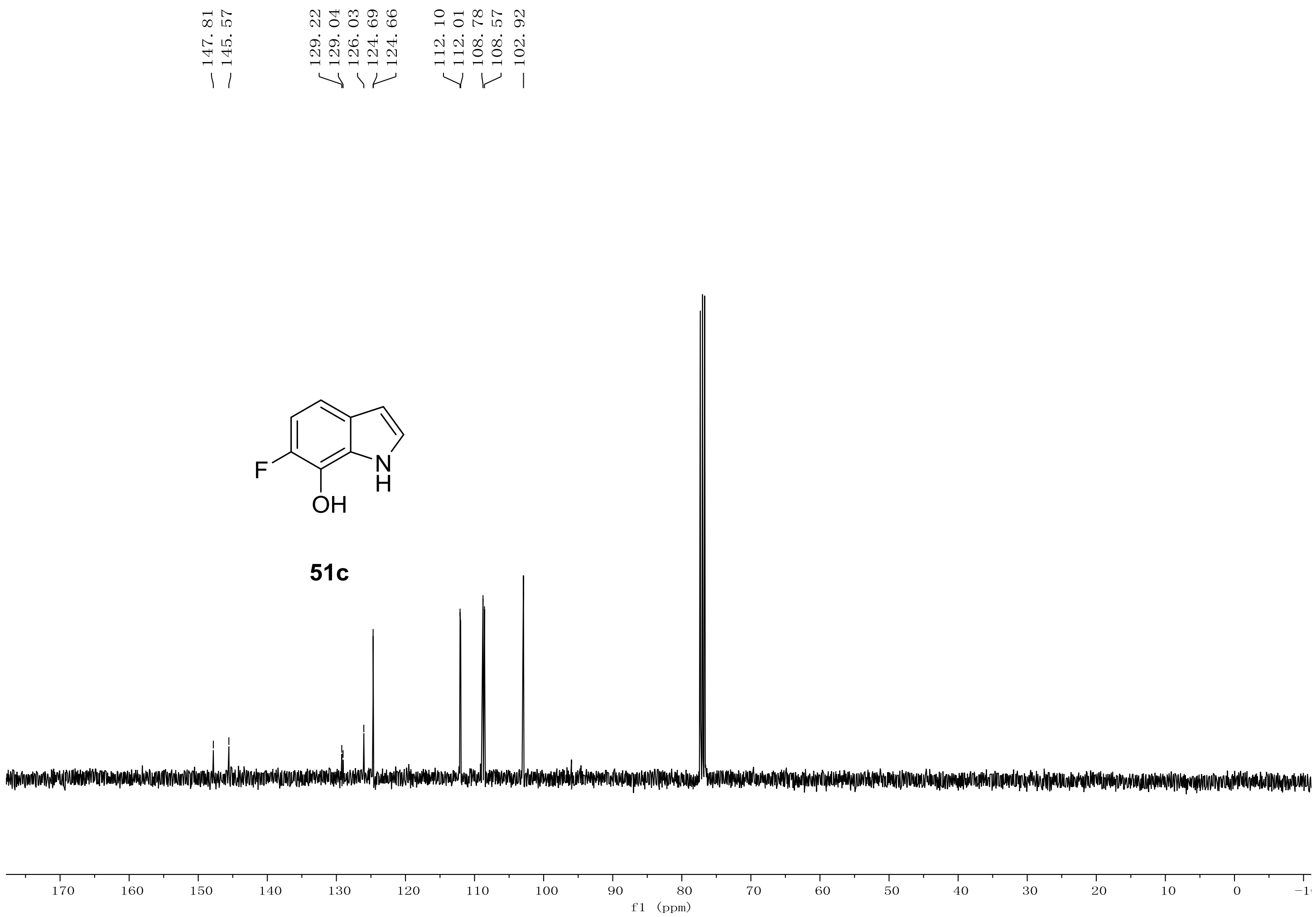

Supplementary Figure 97. ^{13}C NMR spectrum for **51c**

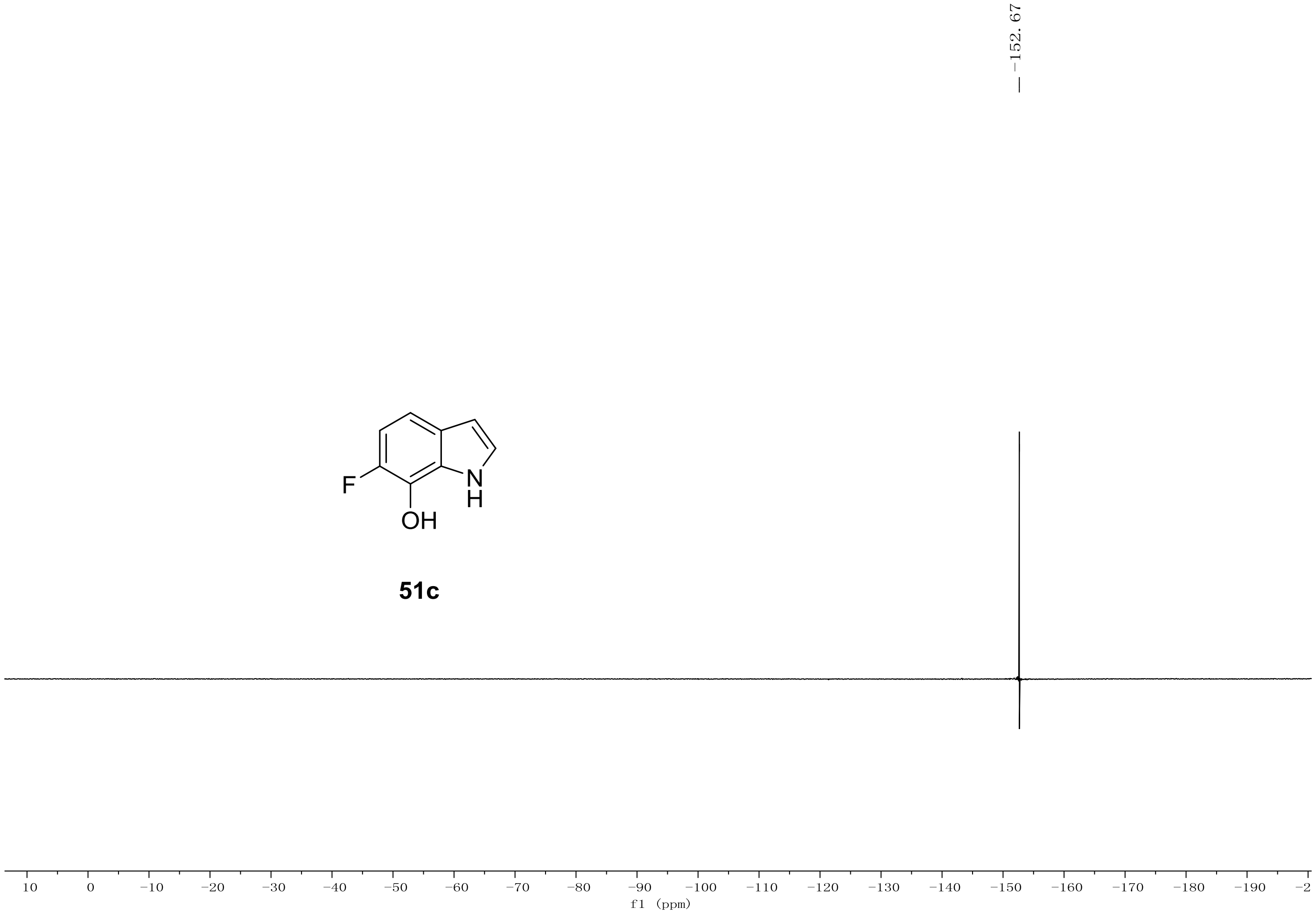

Supplementary Figure 98. ^{19}F NMR spectrum for **51c**

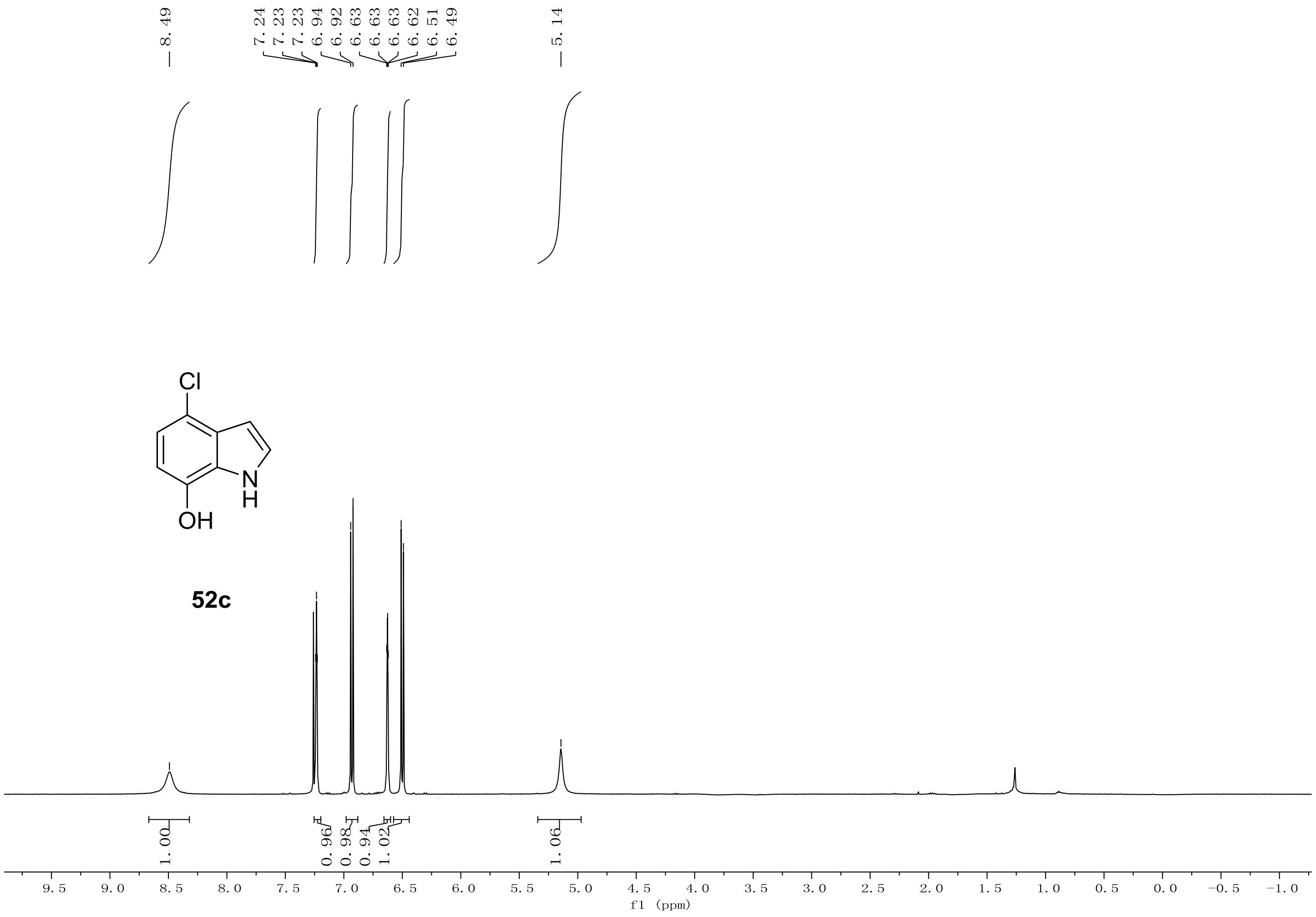

Supplementary Figure 99. ^{1}H NMR spectrum for **52c**

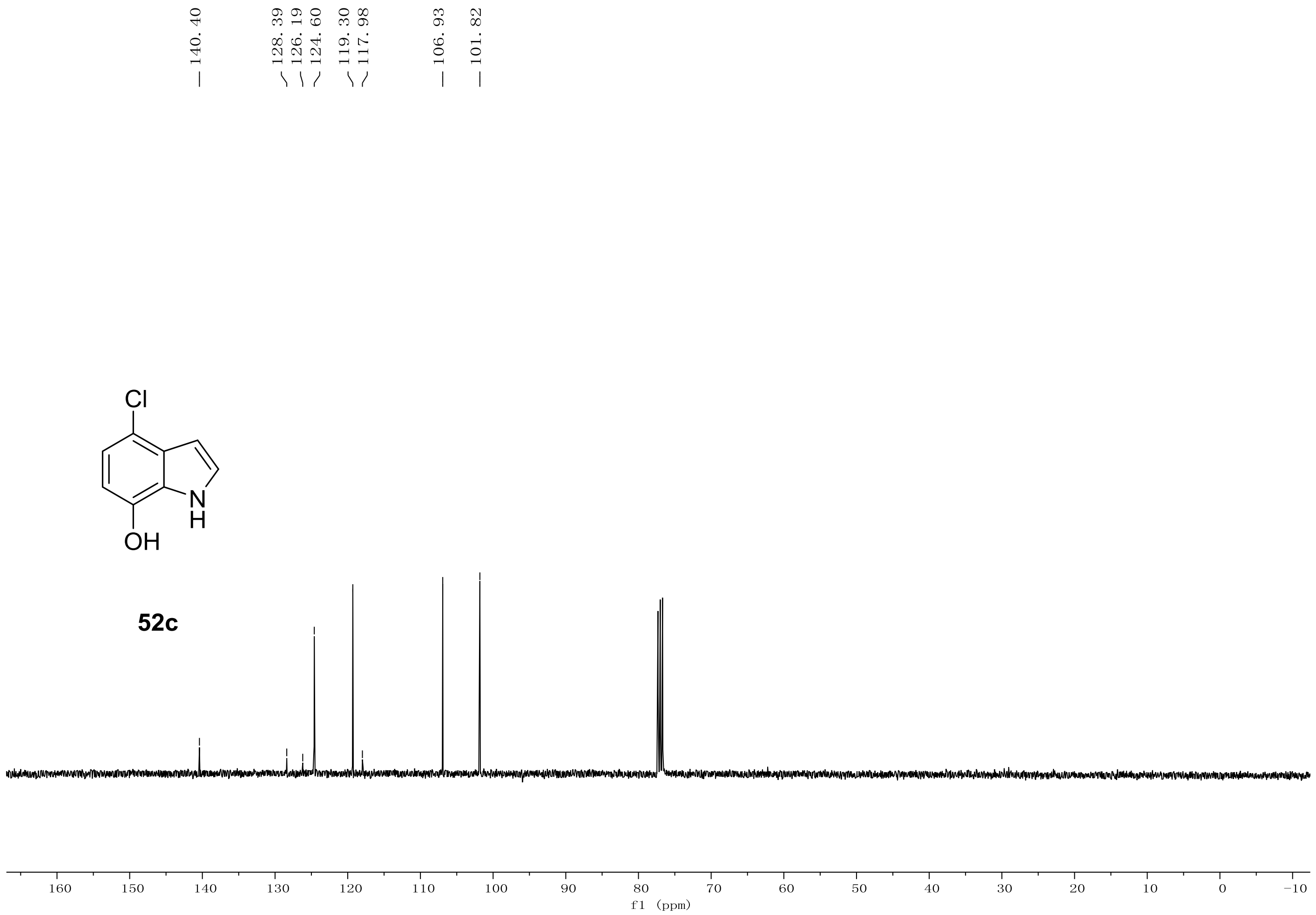

Supplementary Figure 100. ^{13}C NMR spectrum for **52c**

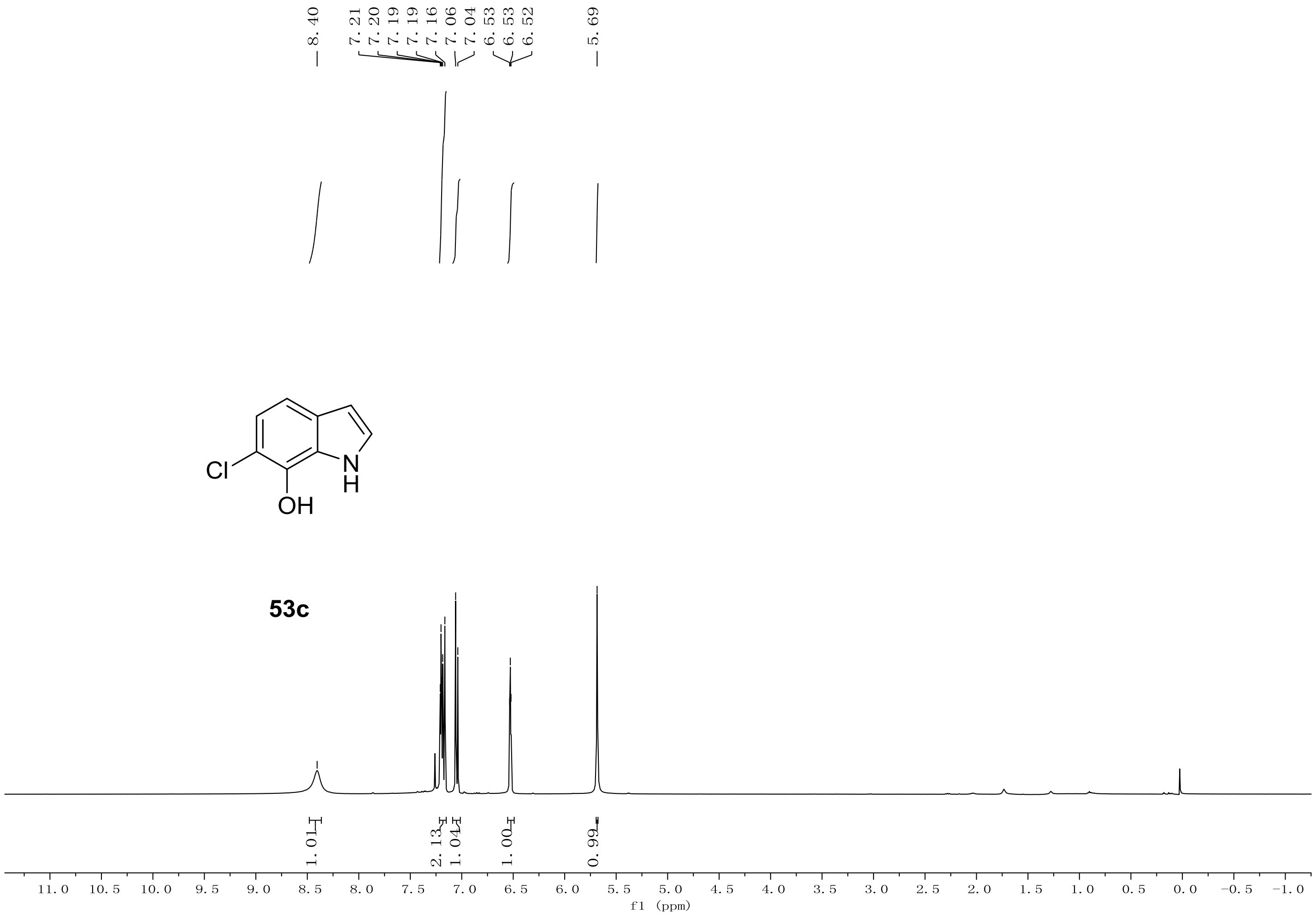

Supplementary Figure 101. ^{1}H NMR spectrum for **53c**

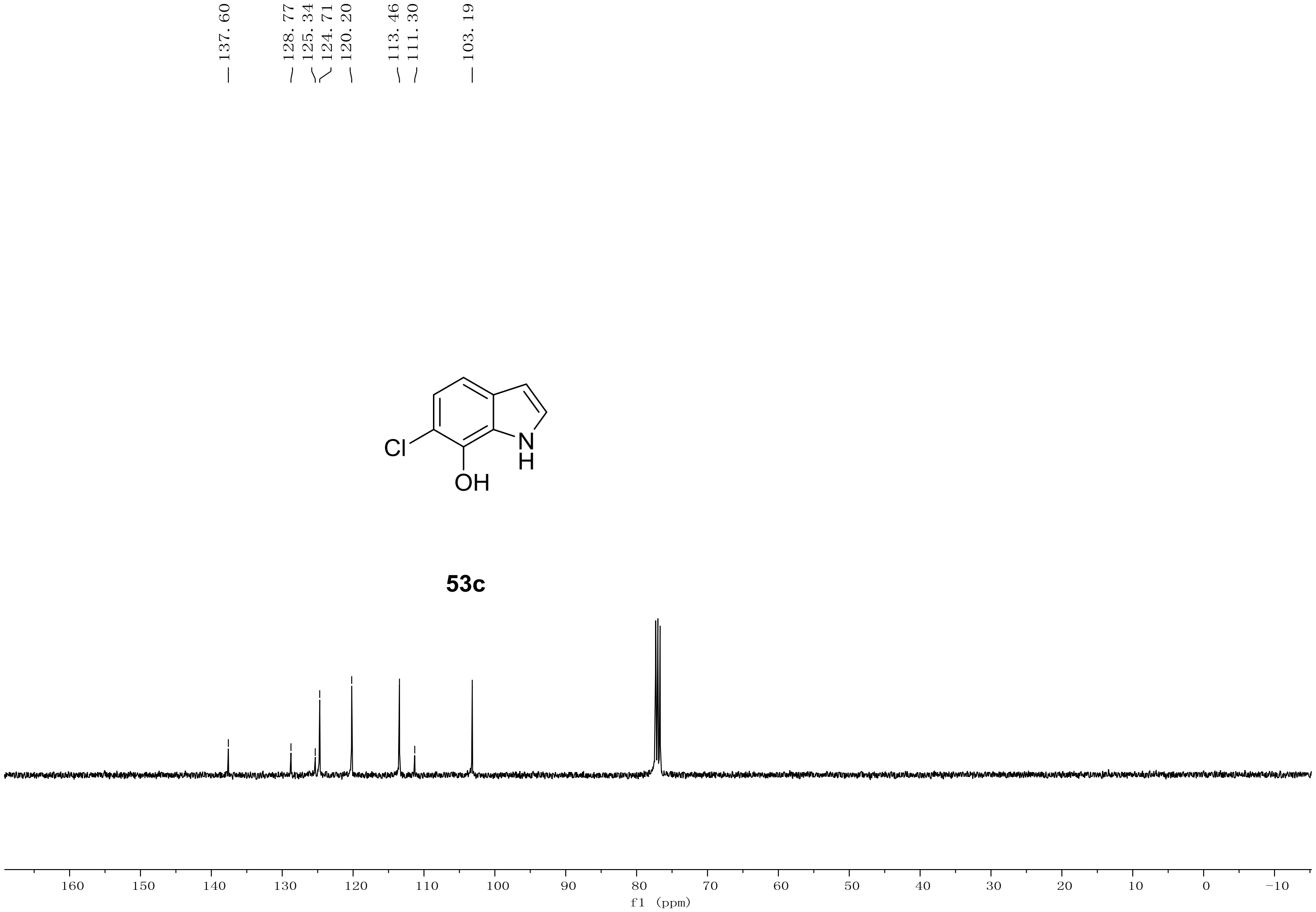

Supplementary Figure 102. ^{13}C NMR spectrum for **53c**

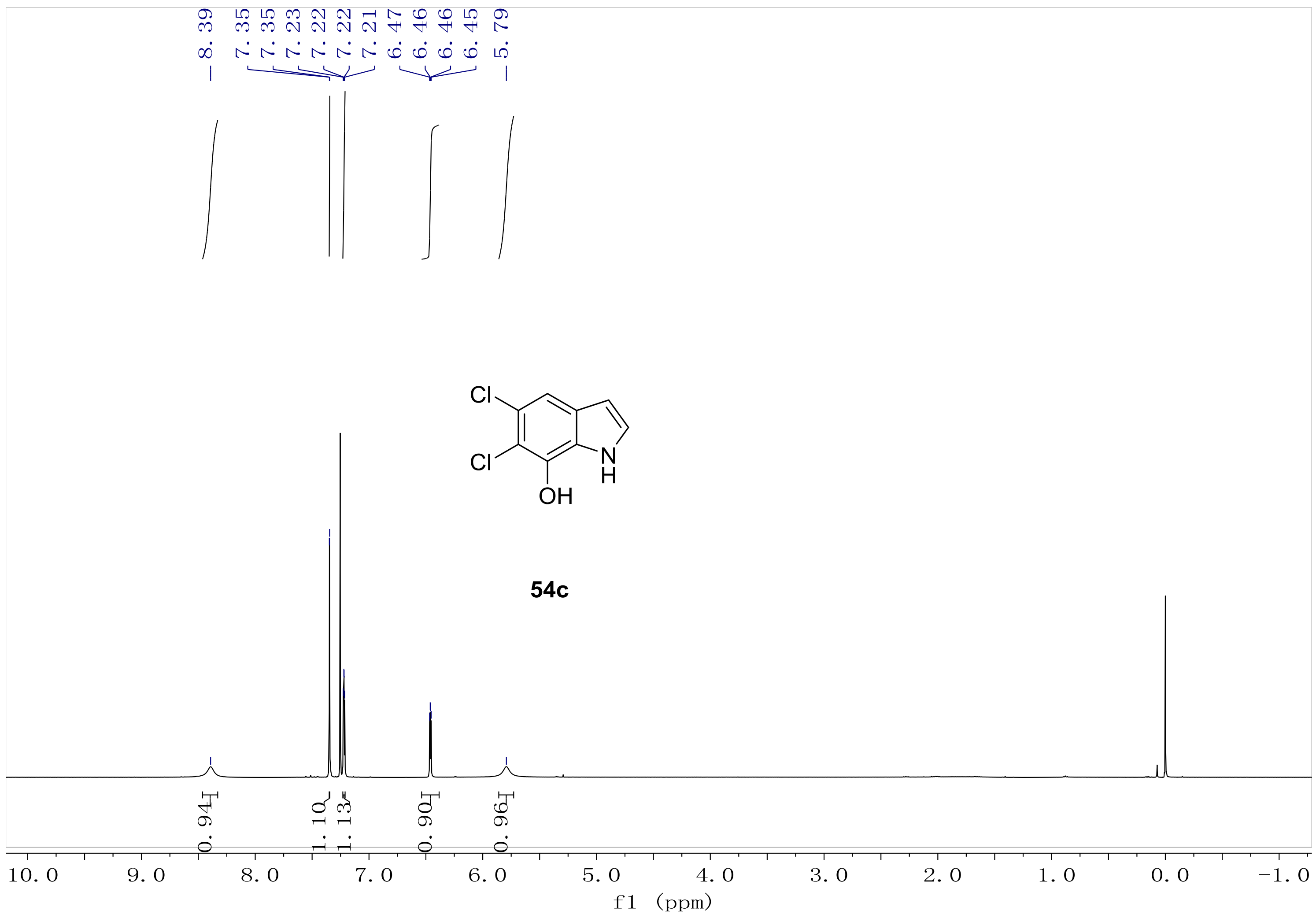

Supplementary Figure 103. ^{1}H NMR spectrum for **54c**

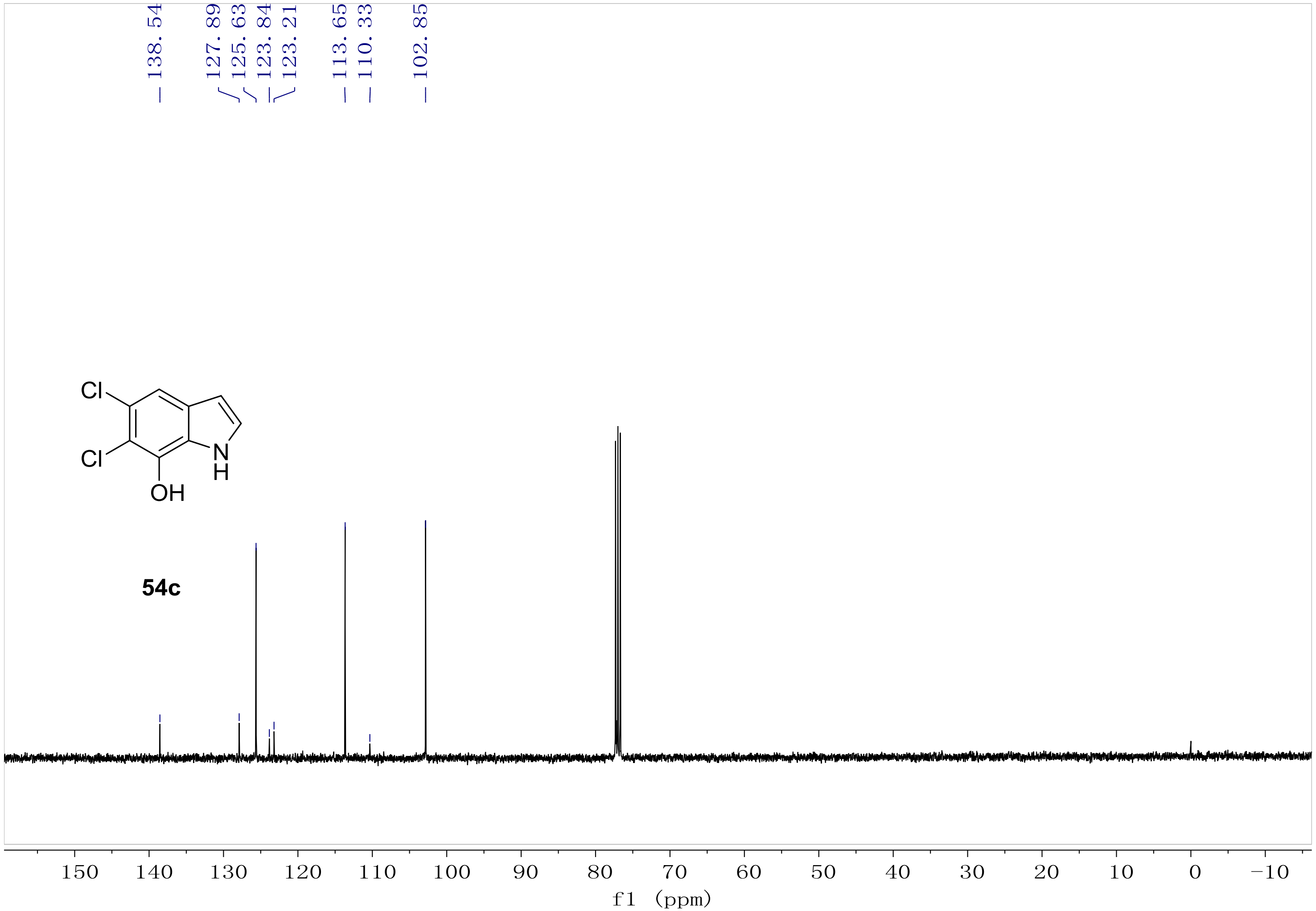

Supplementary Figure 104. ^{13}C NMR spectrum for **54c**

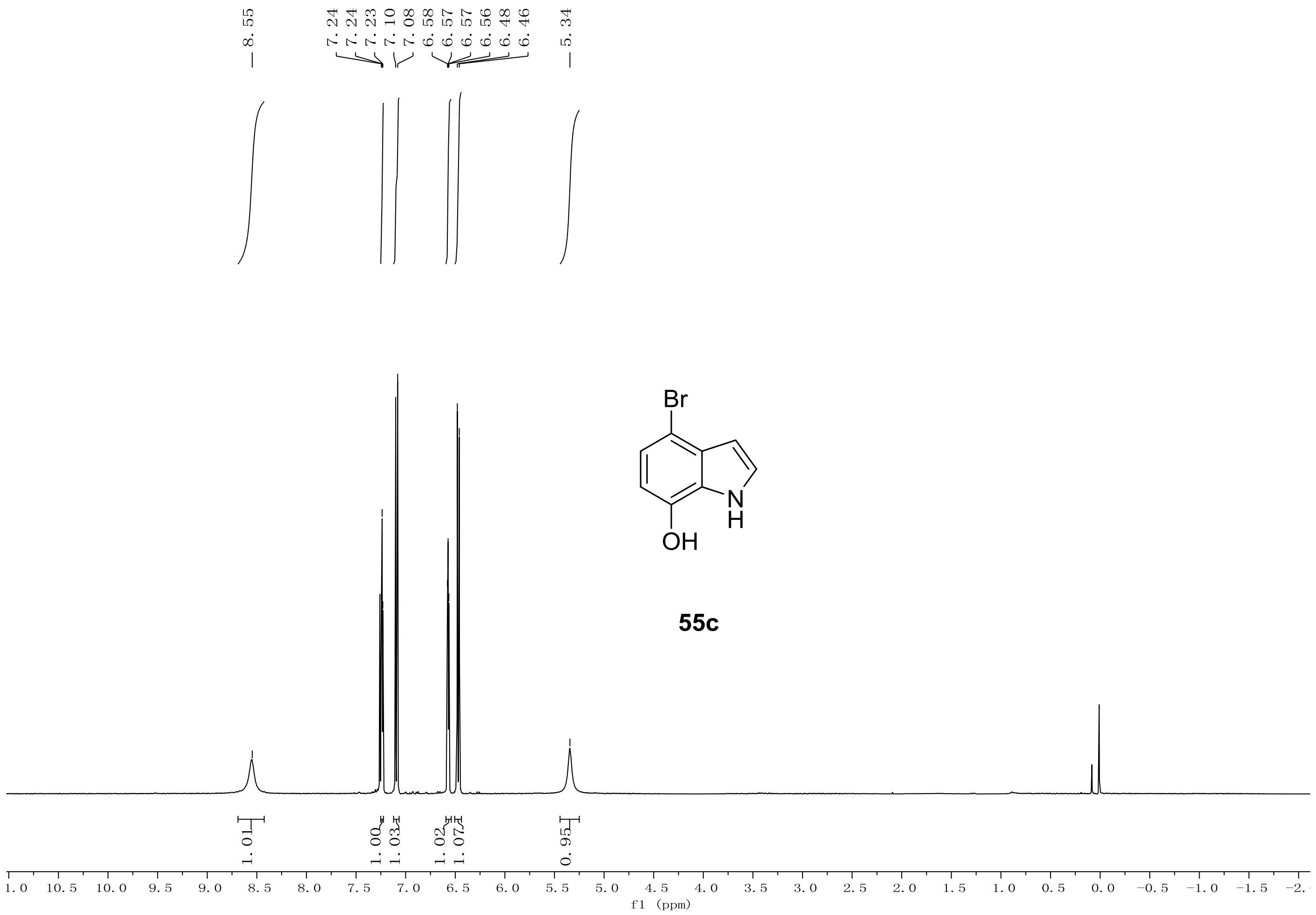

Supplementary Figure 105. ^{1}H NMR spectrum for **55c**

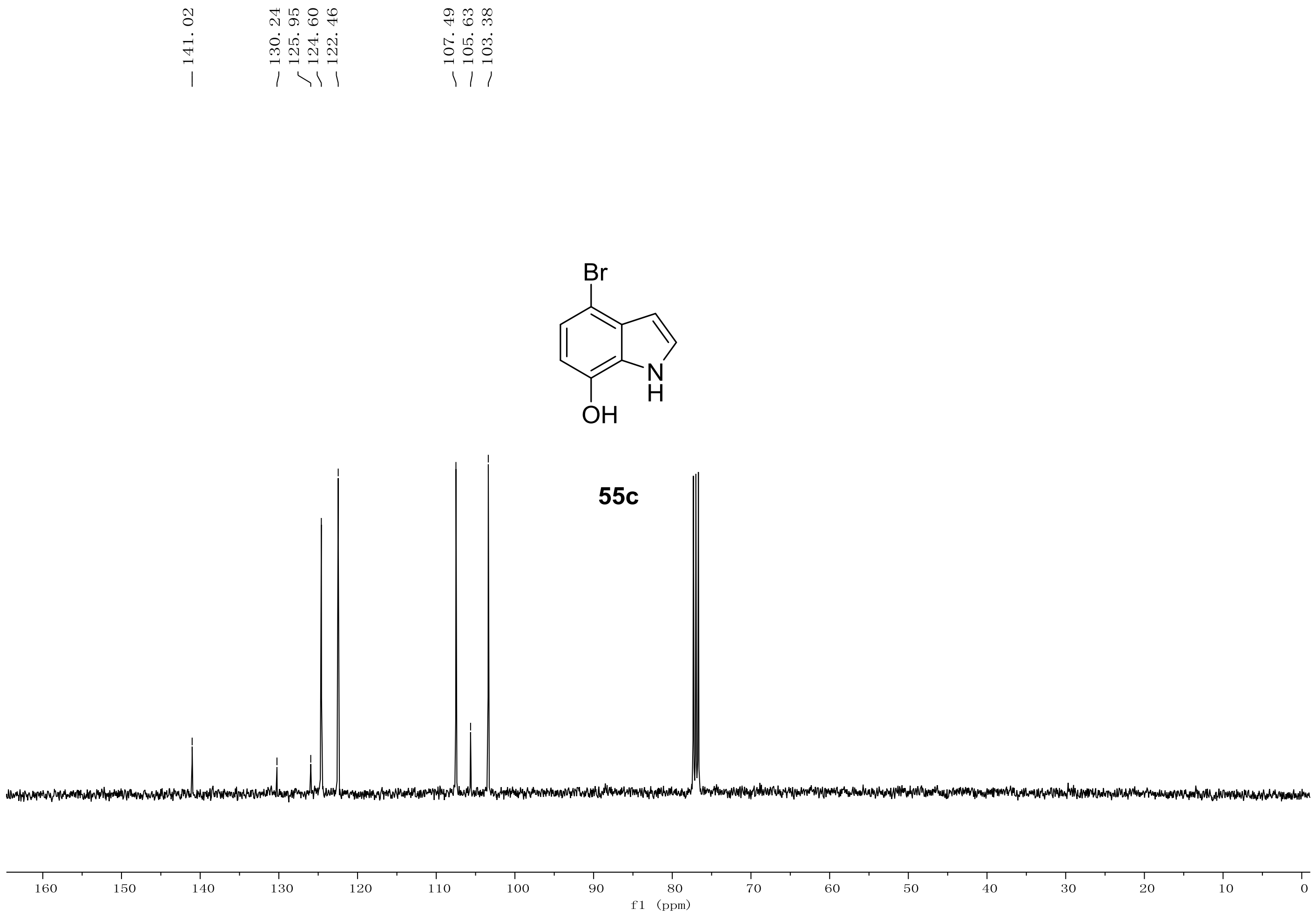

Supplementary Figure 106. ^{13}C NMR spectrum for **55c**

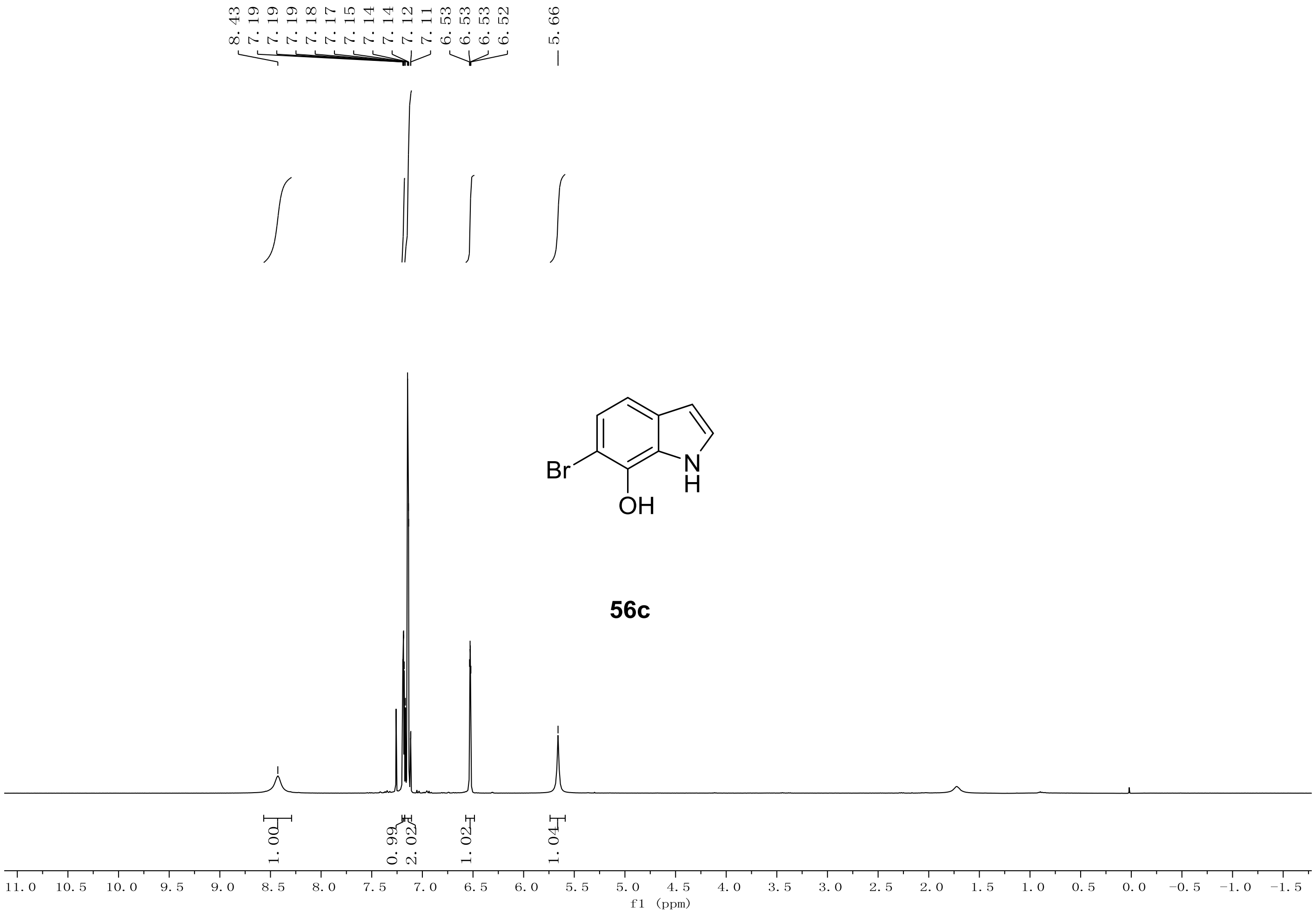

Supplementary Figure 107. ^{1}H NMR spectrum for **56c**

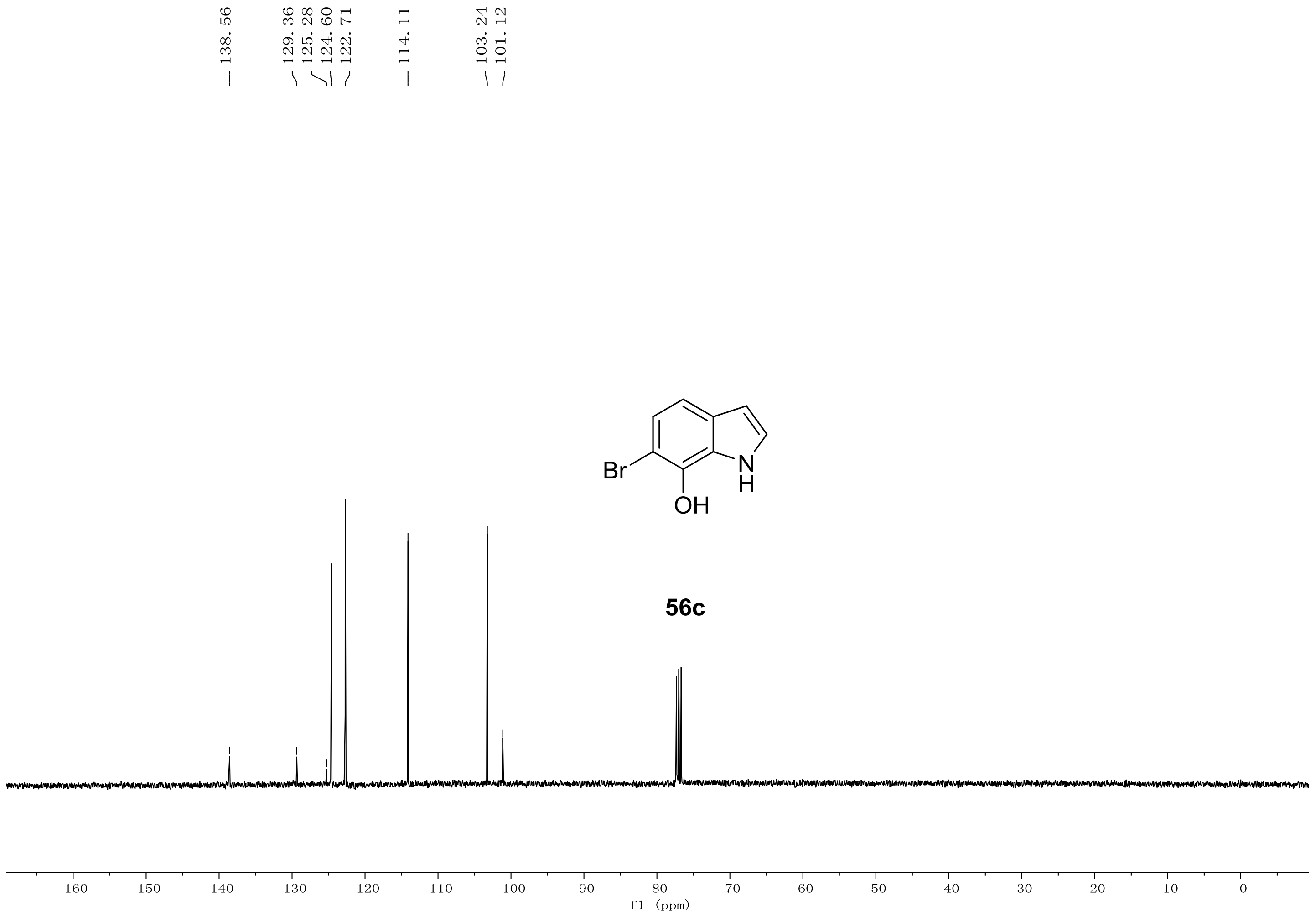

Supplementary Figure 108. ^{13}C NMR spectrum for **56c**

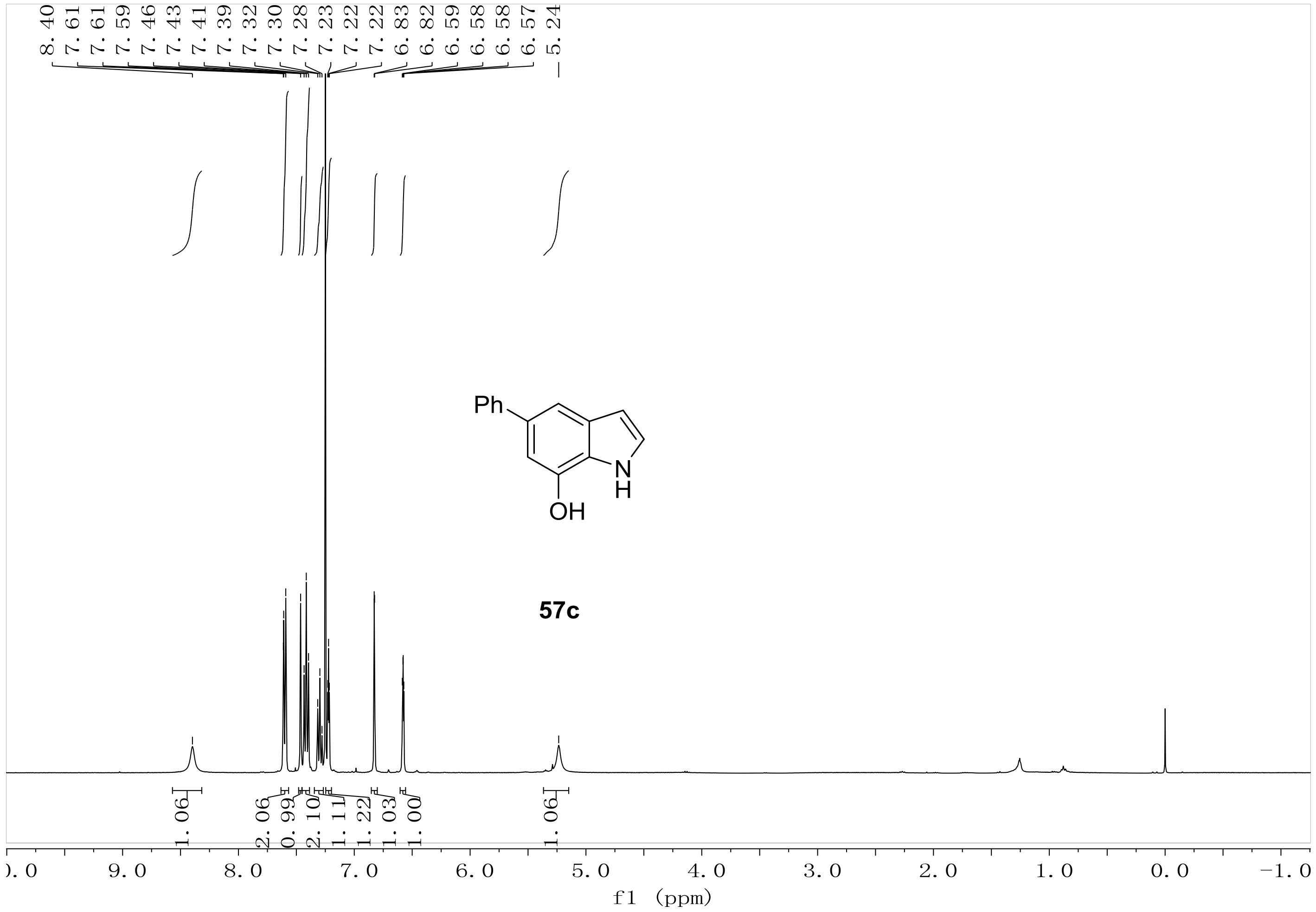

Supplementary Figure 109. ^{1}H NMR spectrum for **57c**

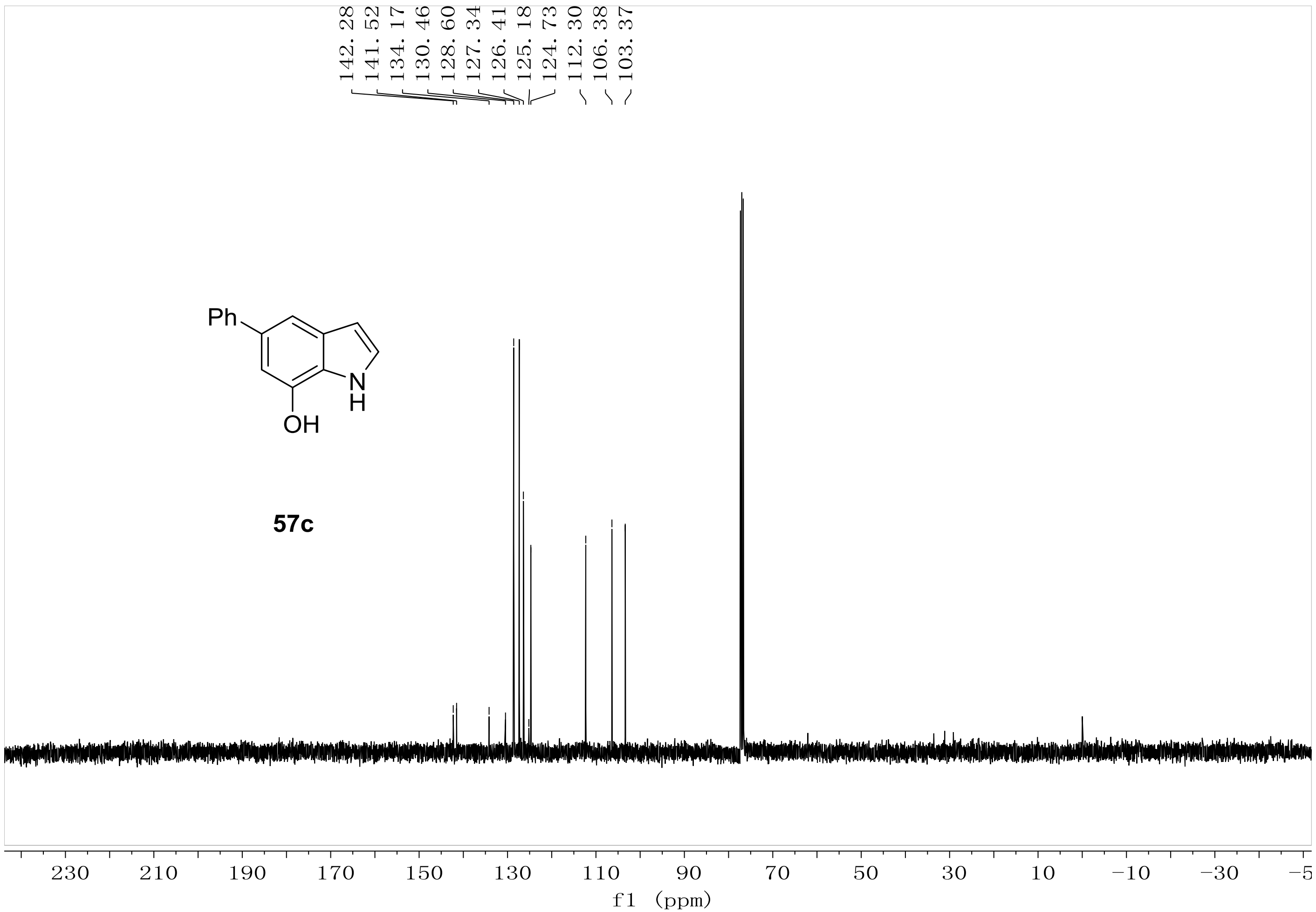

Supplementary Figure 110. ^{13}C NMR spectrum for **57c**

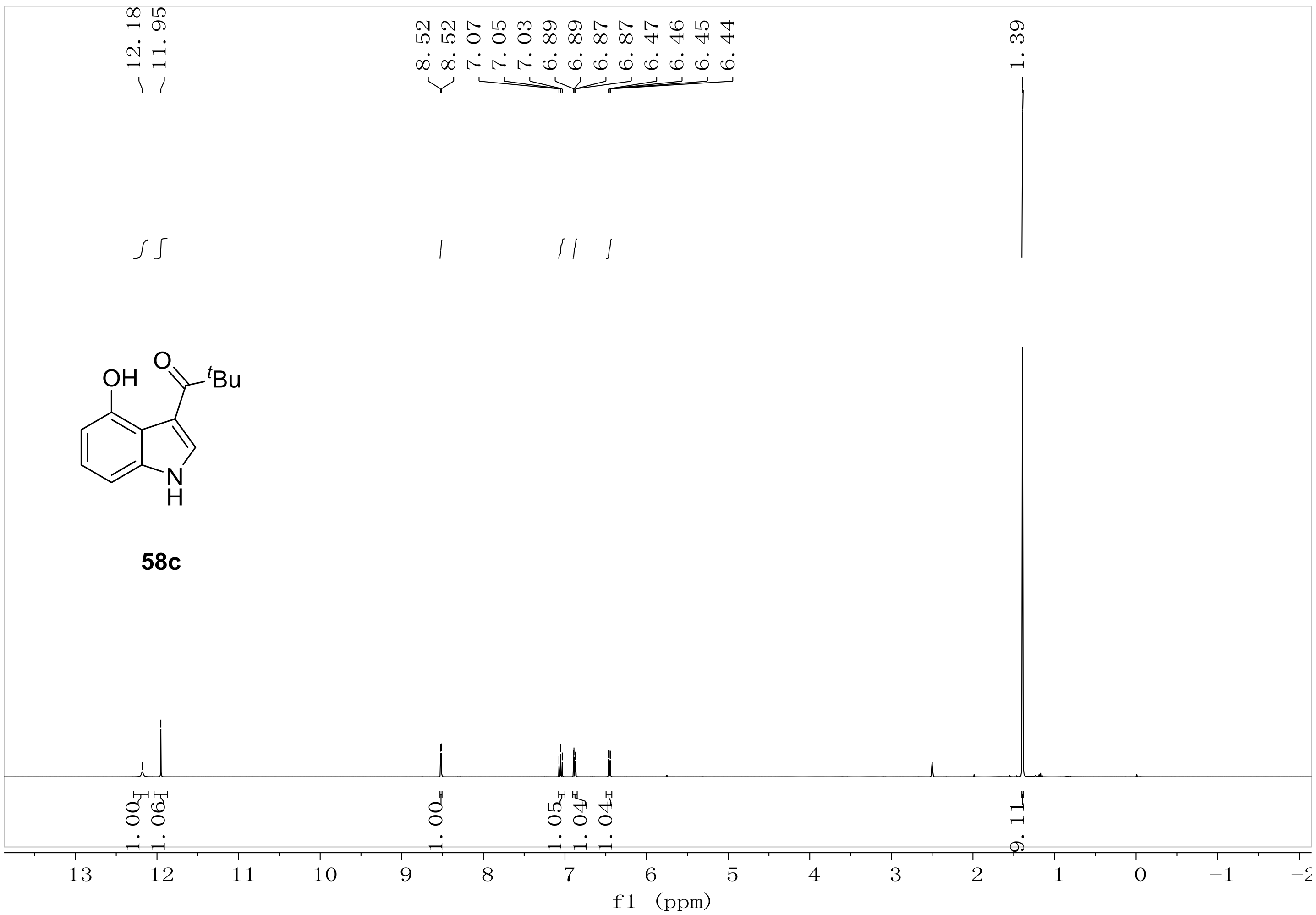

Supplementary Figure 111. ¹H NMR spectrum for **58c**

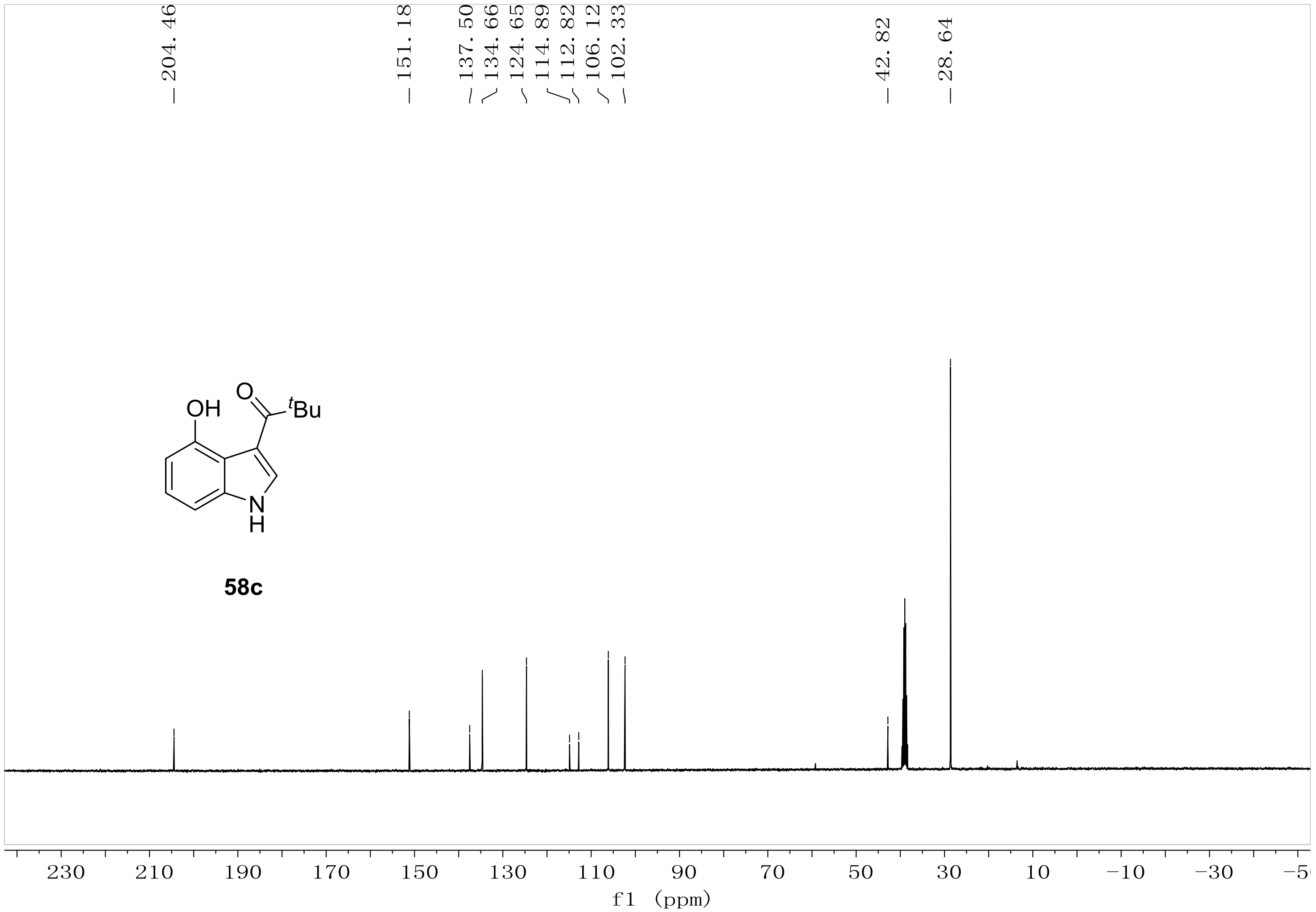

Supplementary Figure 112. ^{13}C NMR spectrum for **58c**

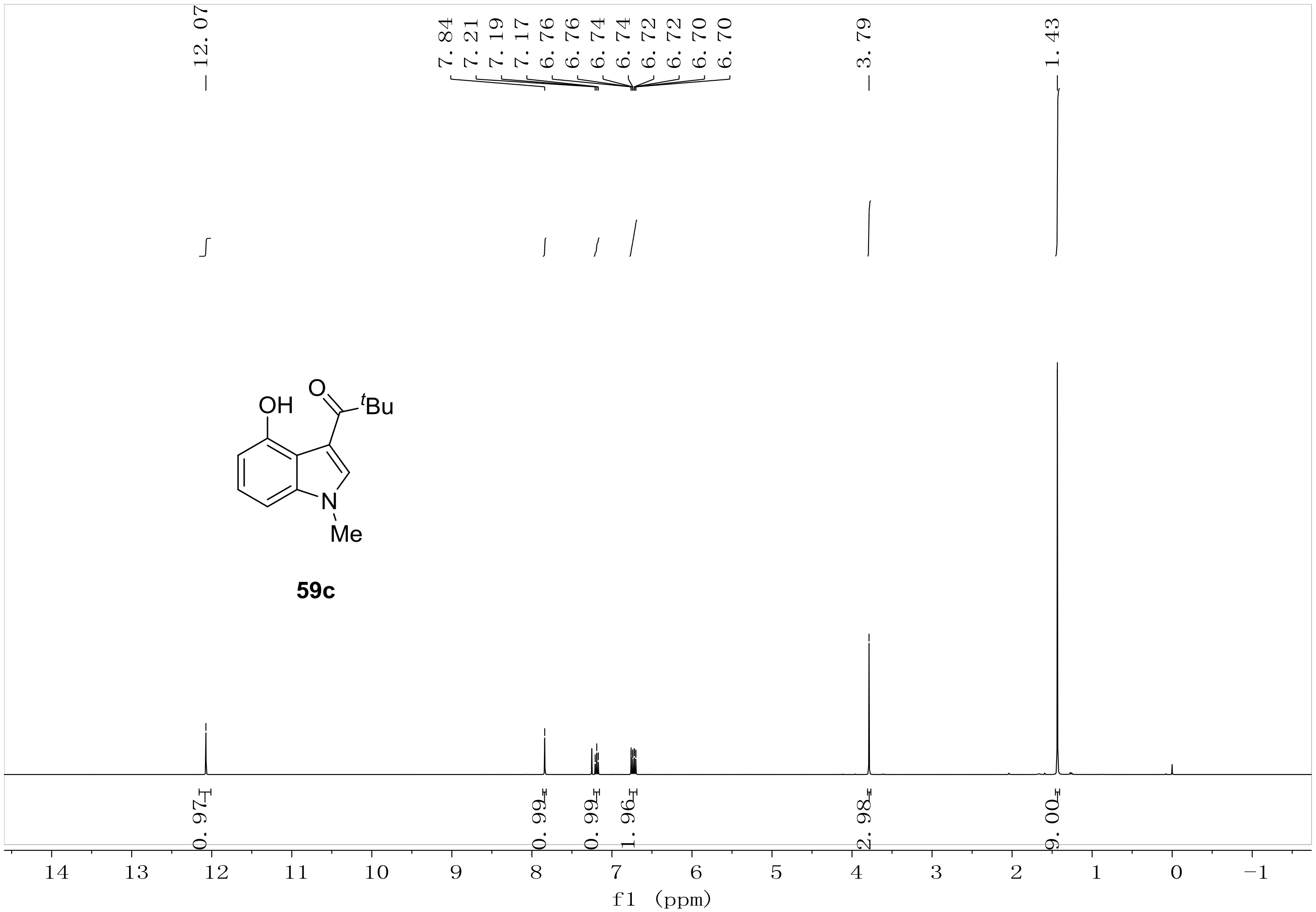

Supplementary Figure 113. ^{1}H NMR spectrum for **59c**

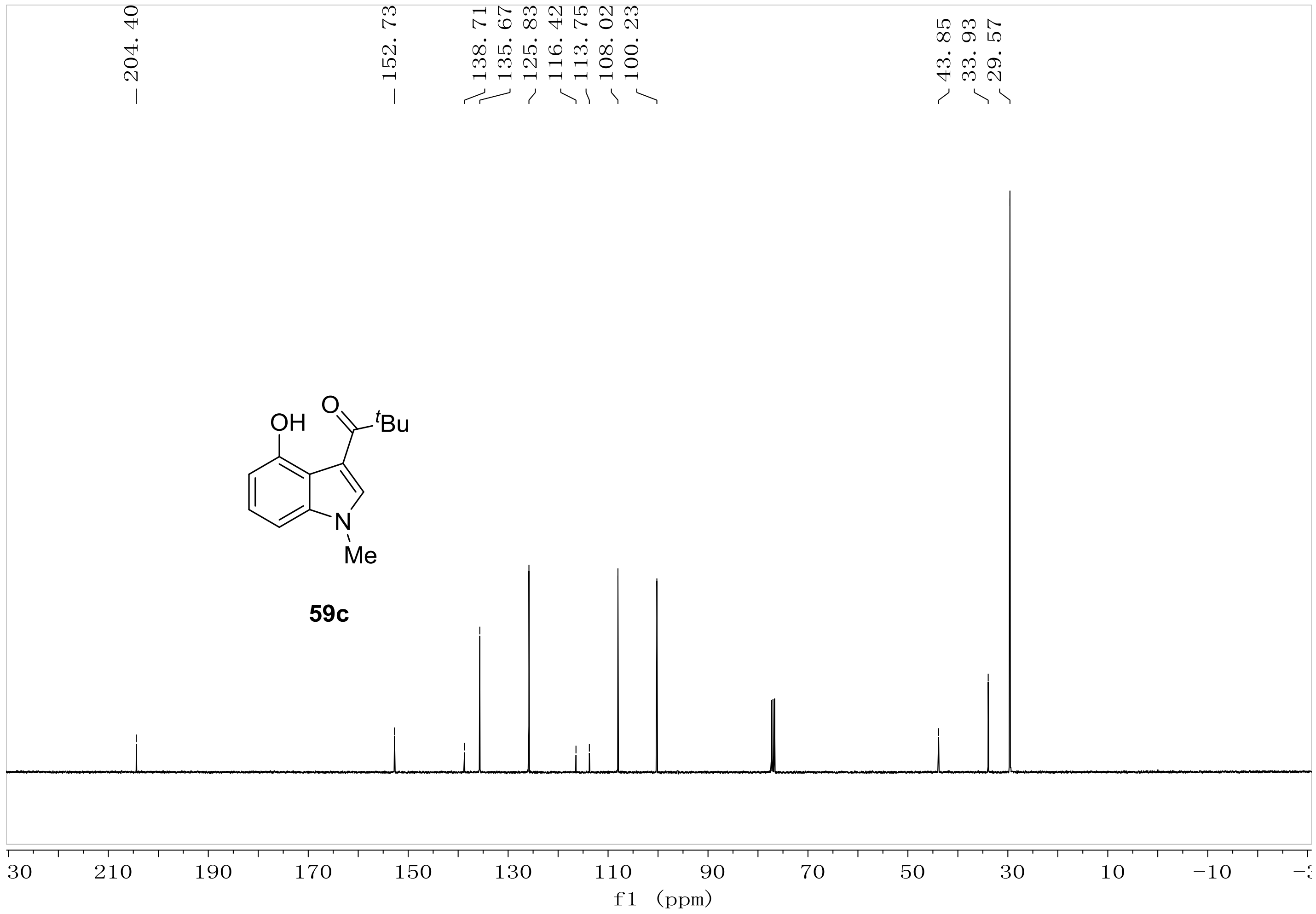

Supplementary Figure 114. ^{13}C NMR spectrum for **59c**

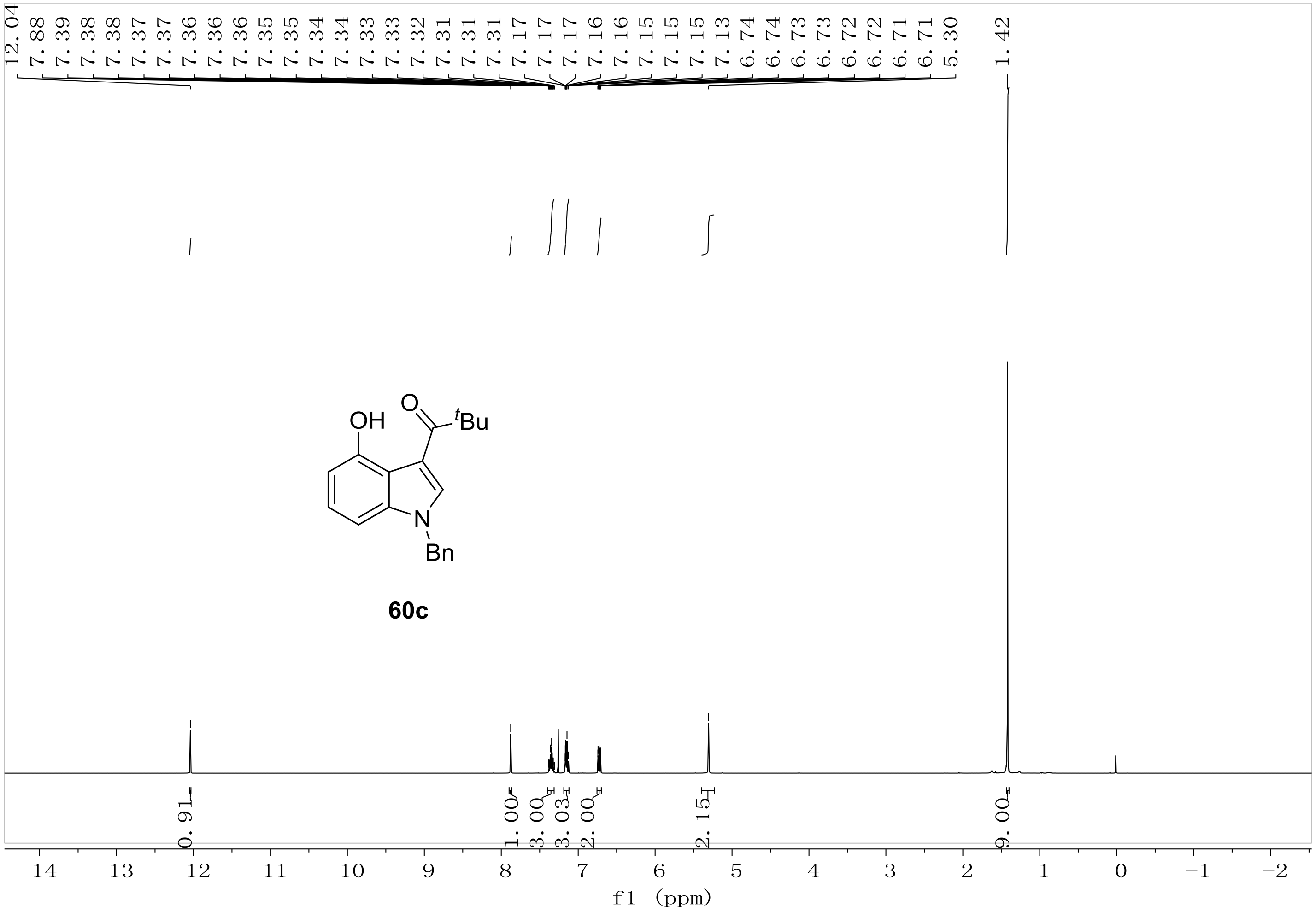

Supplementary Figure 115. ^{1}H NMR spectrum for **60c**

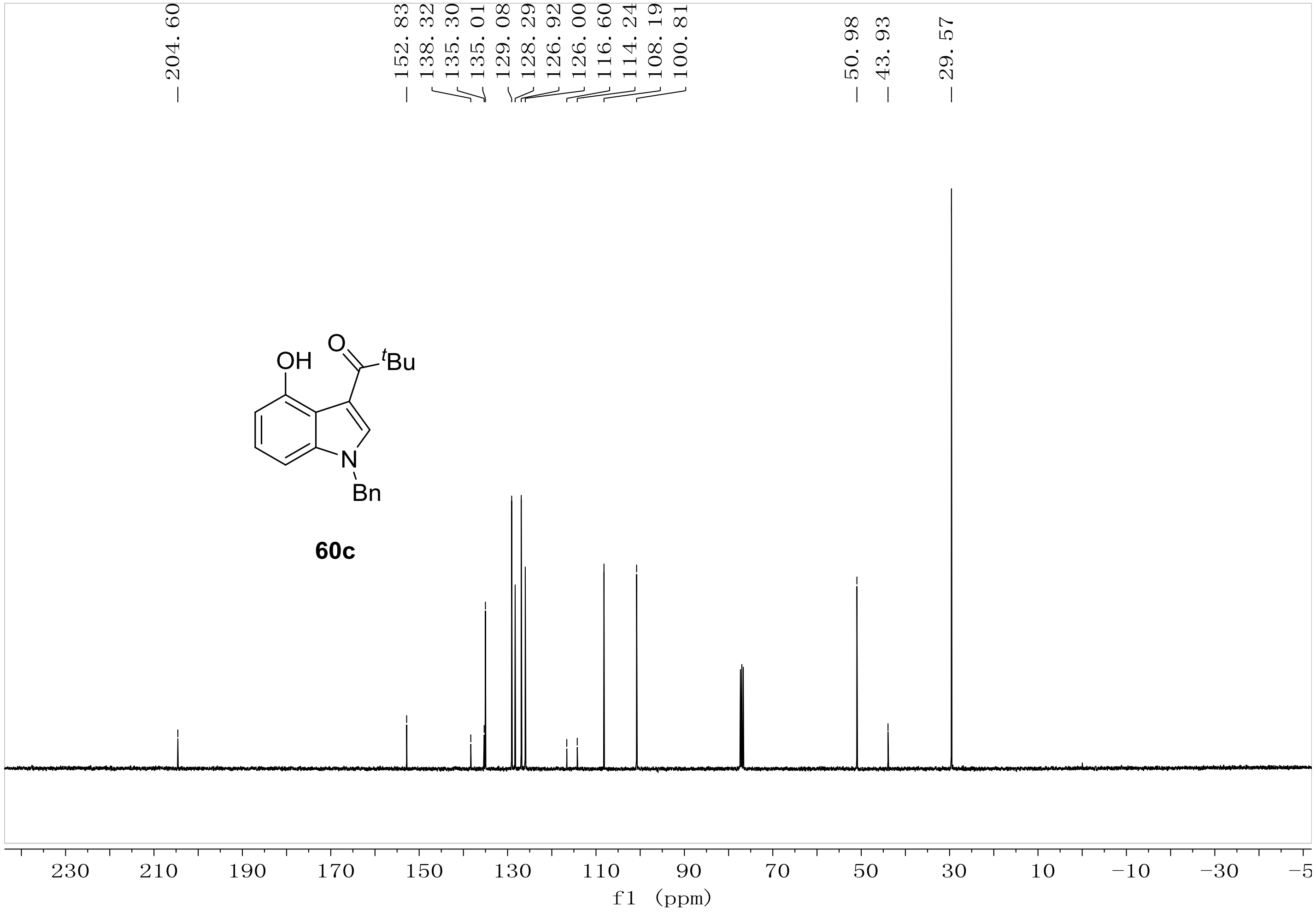

Supplementary Figure 116. ^{13}C NMR spectrum for **60c**

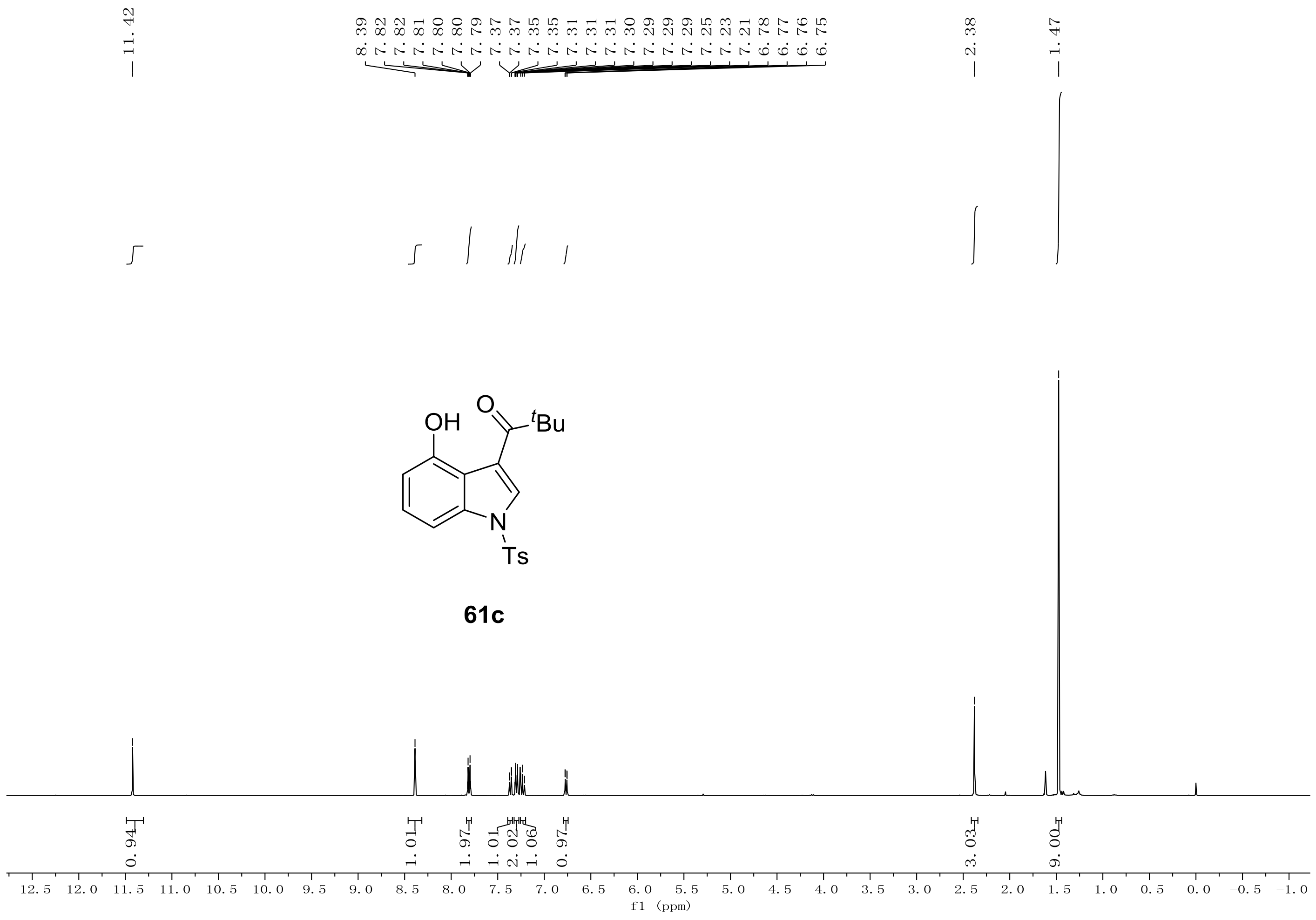

Supplementary Figure 117. ^{1}H NMR spectrum for **61c**

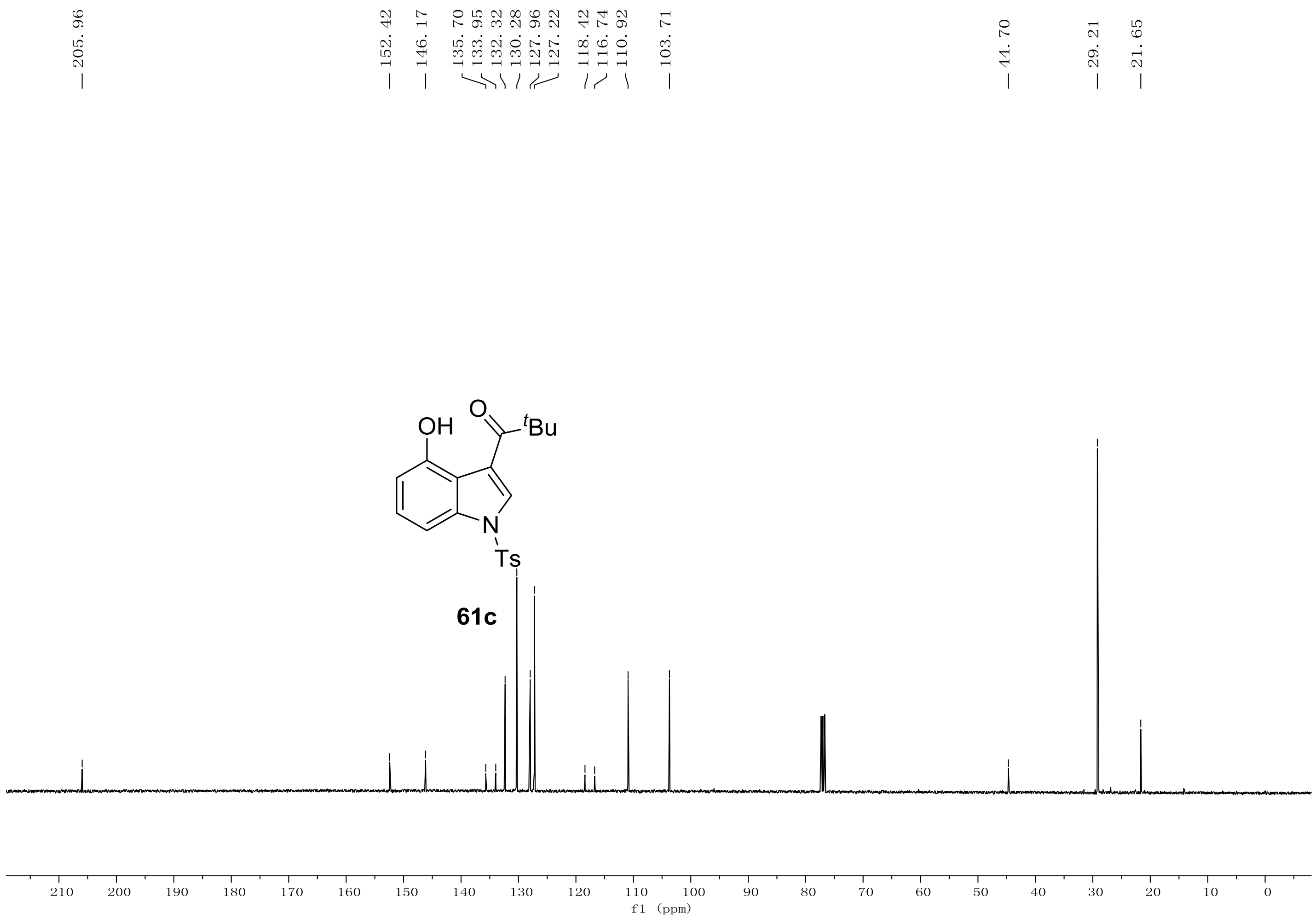

Supplementary Figure 118. ^{13}C NMR spectrum for **61c**

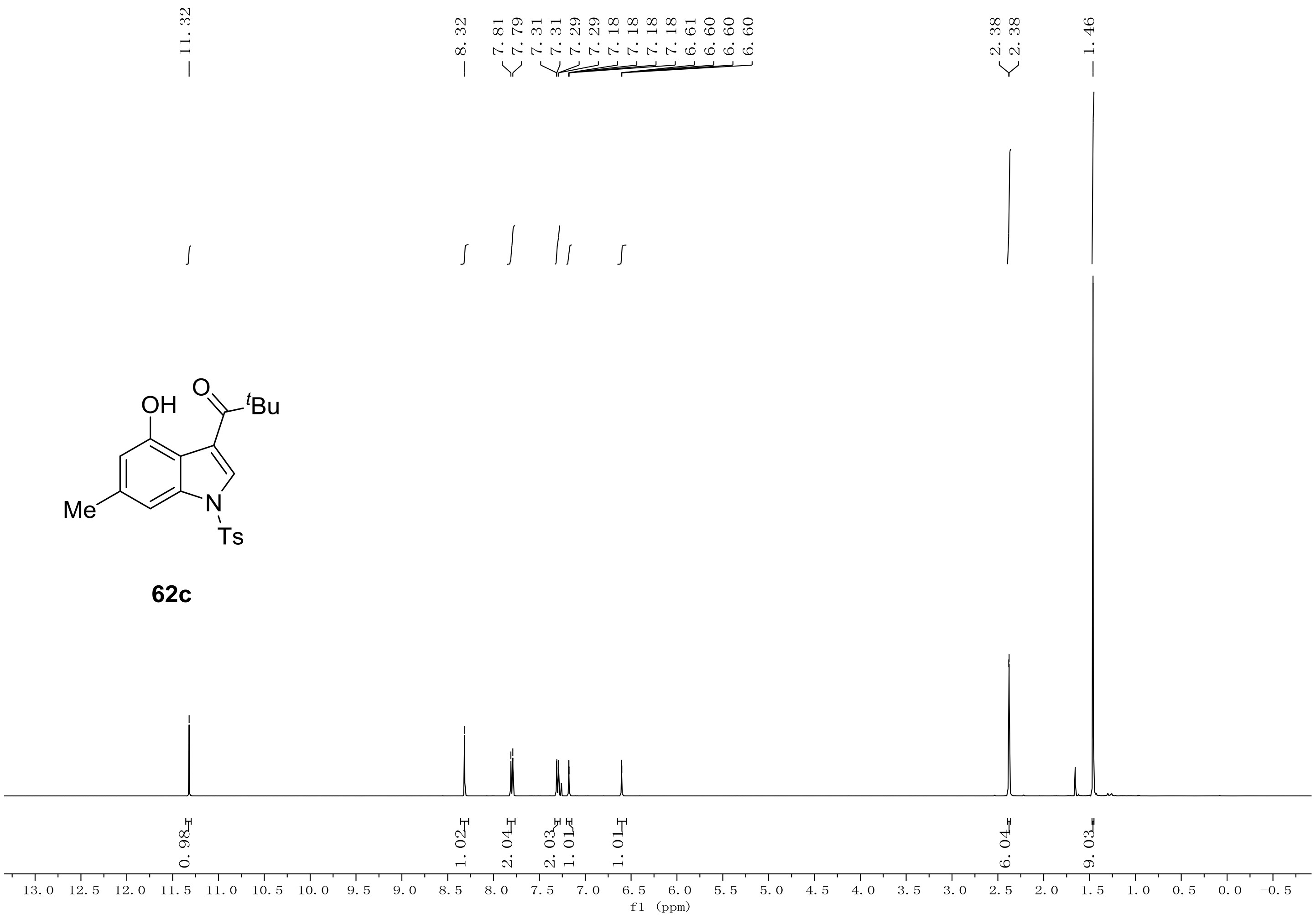

Supplementary Figure 119. ^{1}H NMR spectrum for **62c**

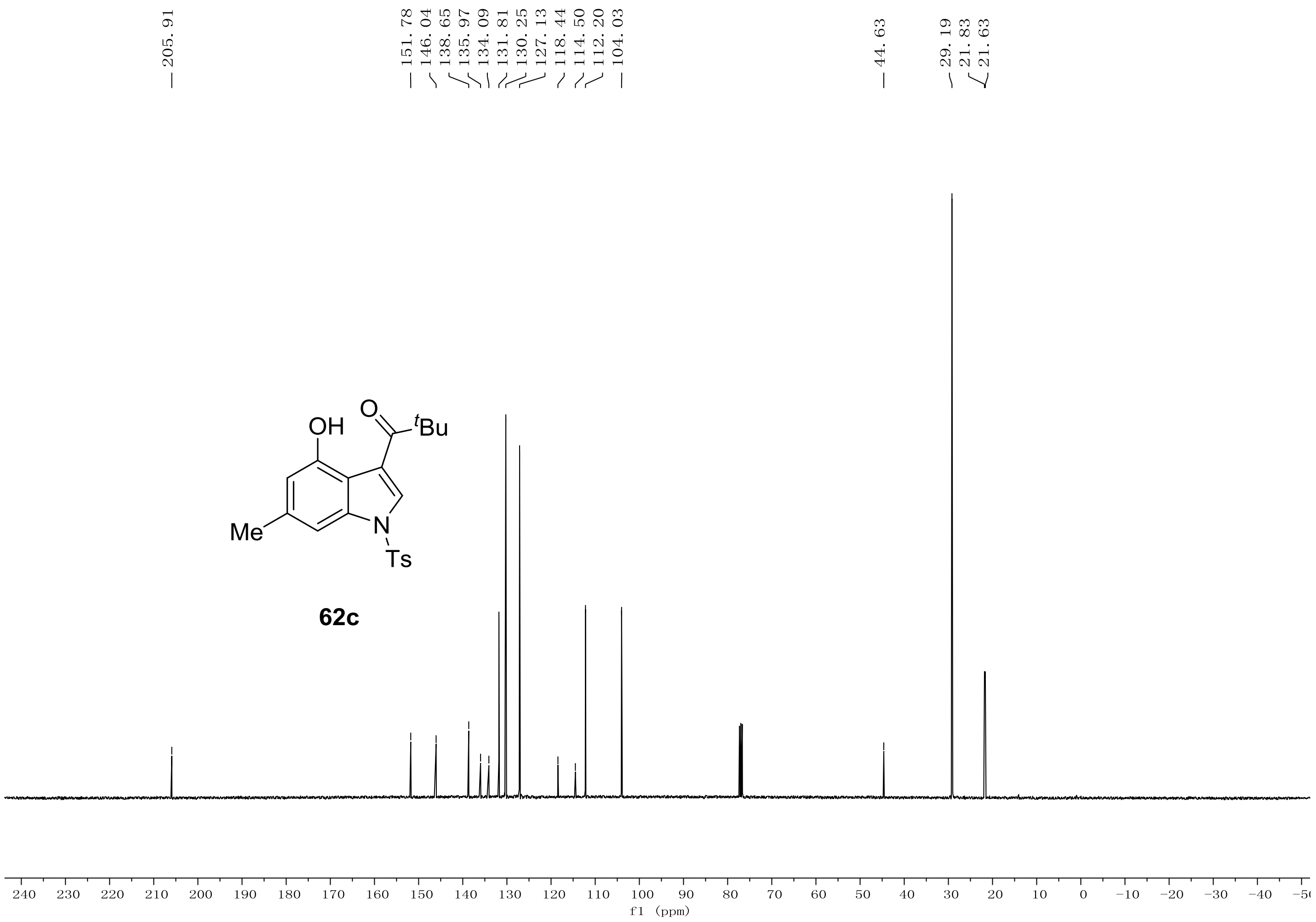

Supplementary Figure 120. ^{13}C NMR spectrum for **62c**

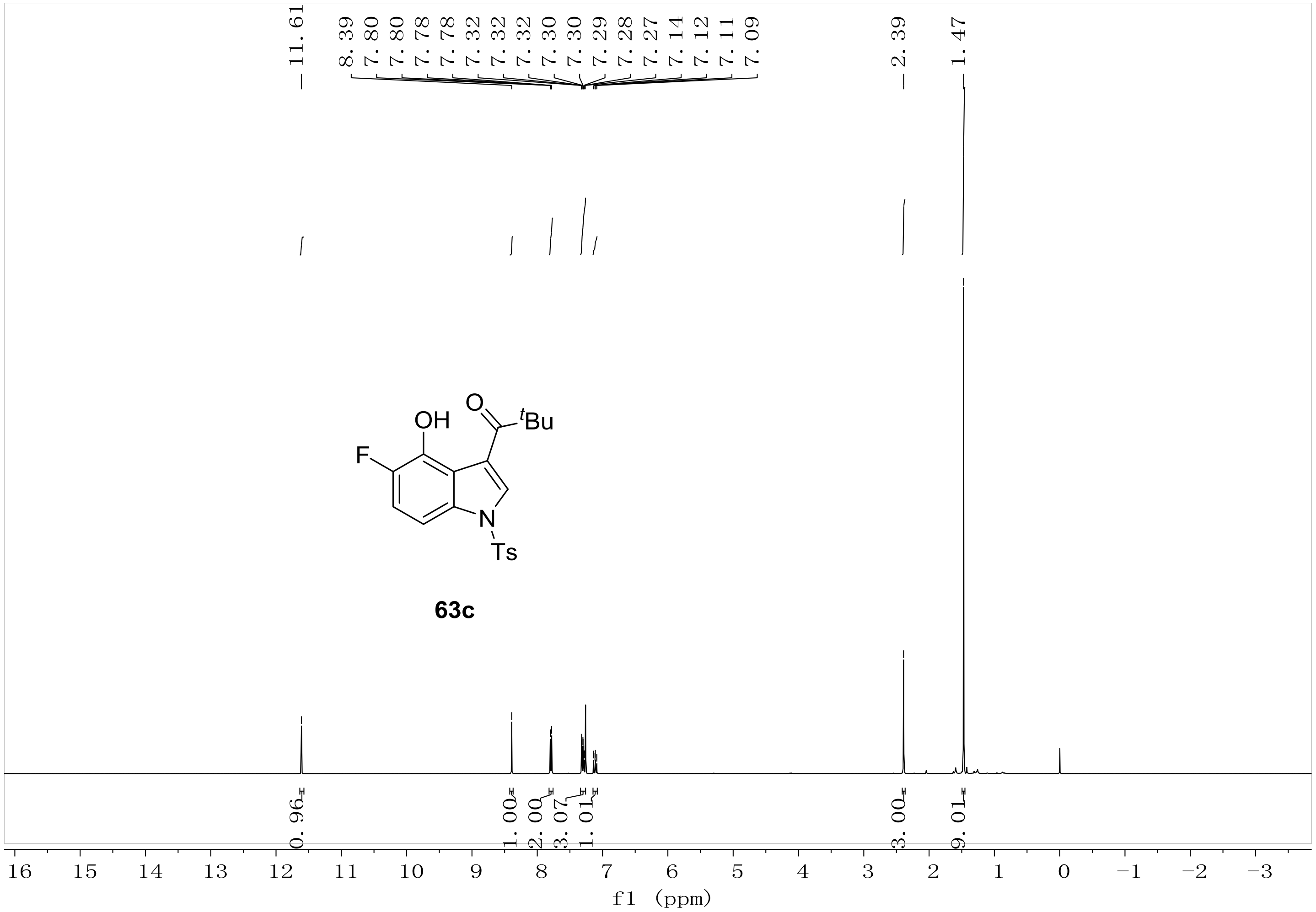

Supplementary Figure 121. ^{1}H NMR spectrum for **63c**

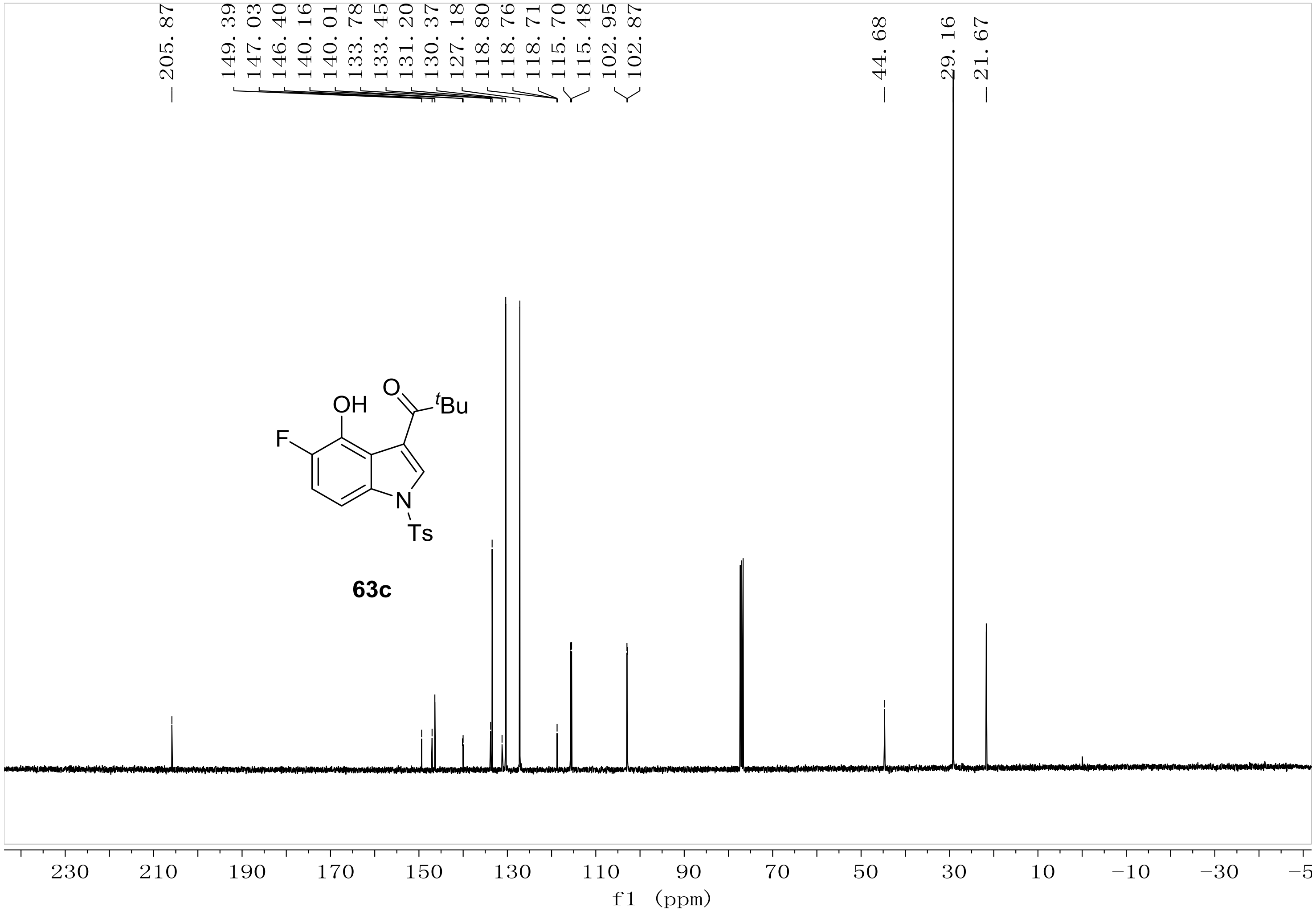

Supplementary Figure 122. ^{13}C NMR spectrum for **63c**

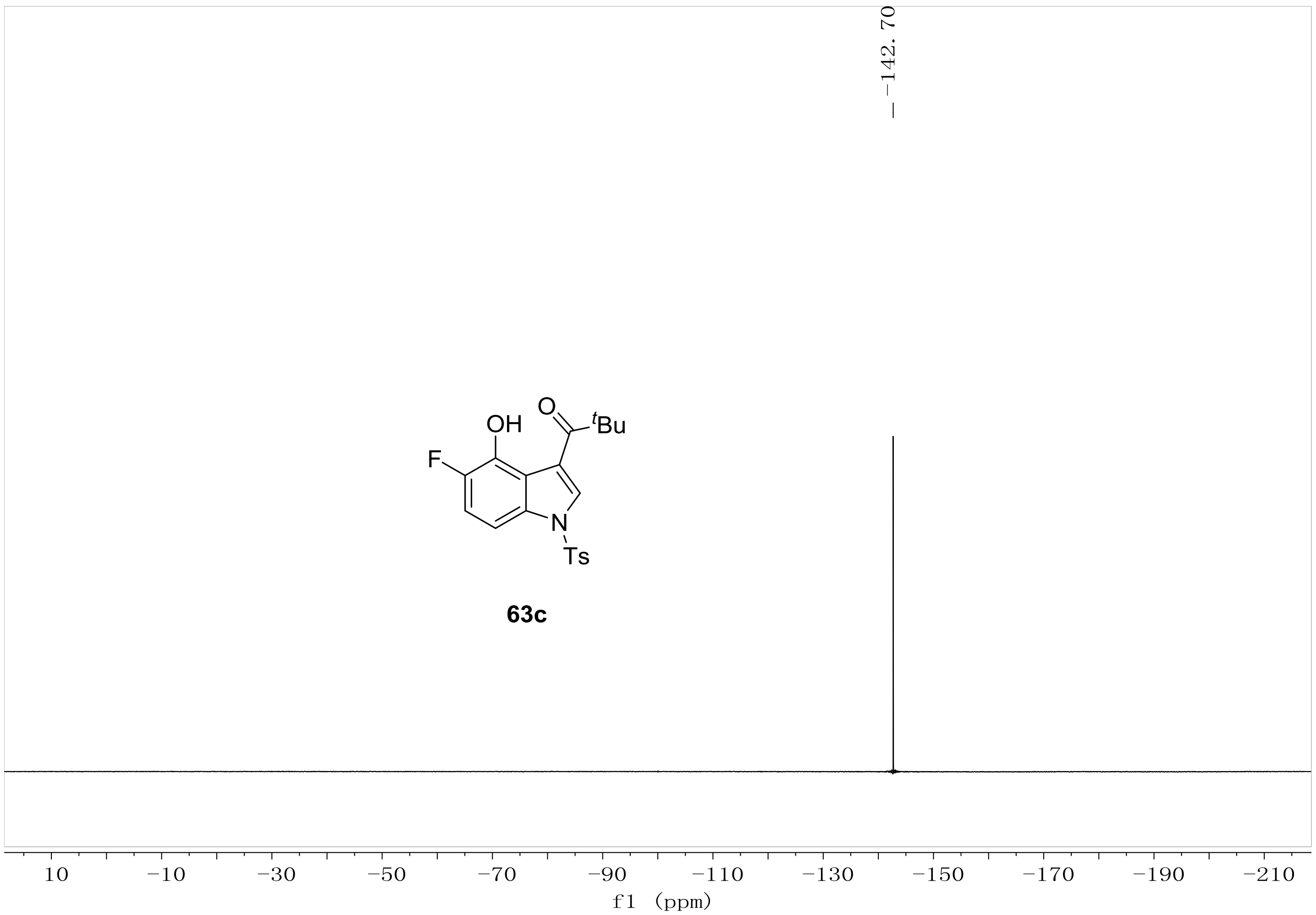

Supplementary Figure 123. ^{19}F NMR spectrum for **63c**

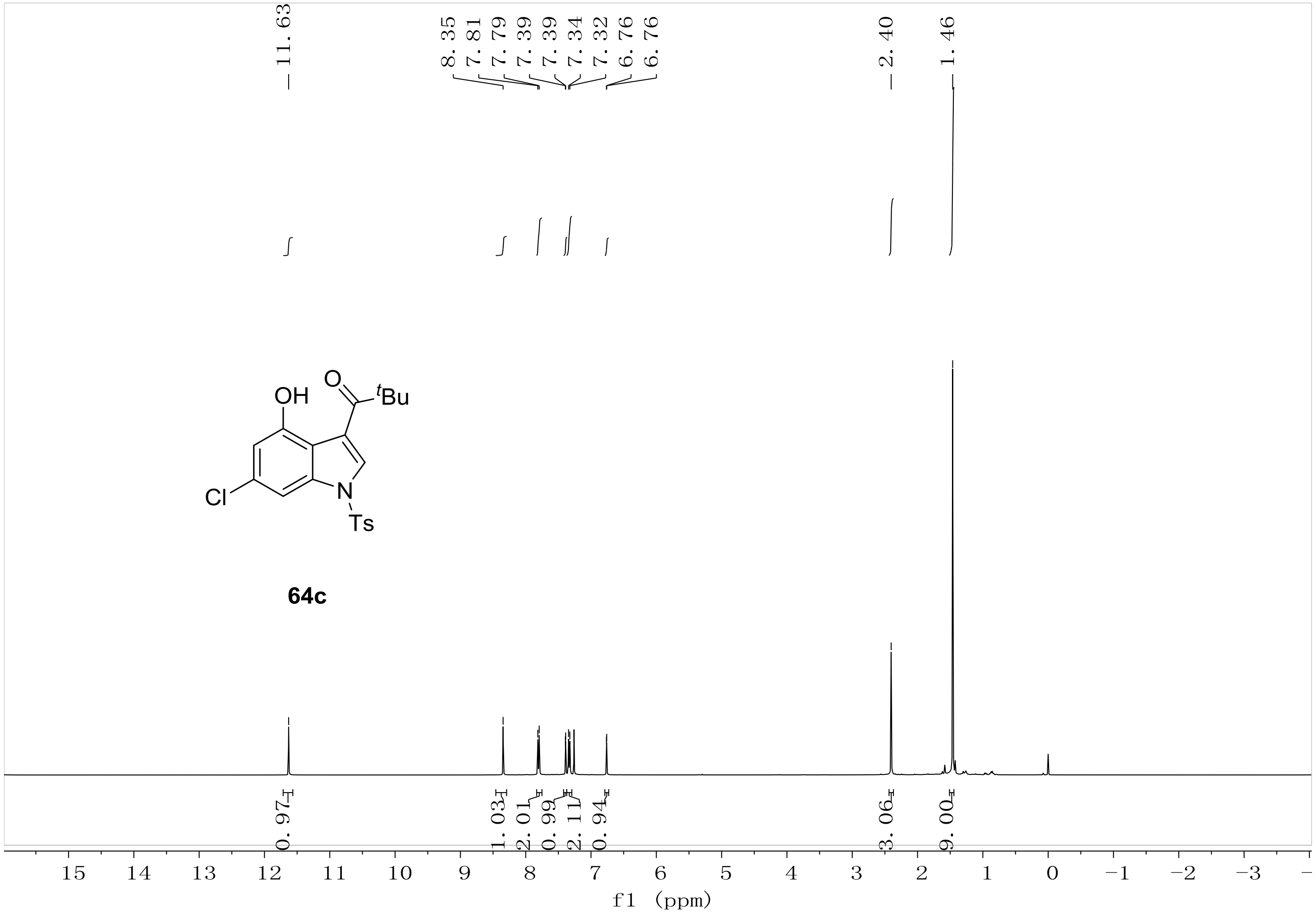

Supplementary Figure 124. ^{1}H NMR spectrum for **64c**

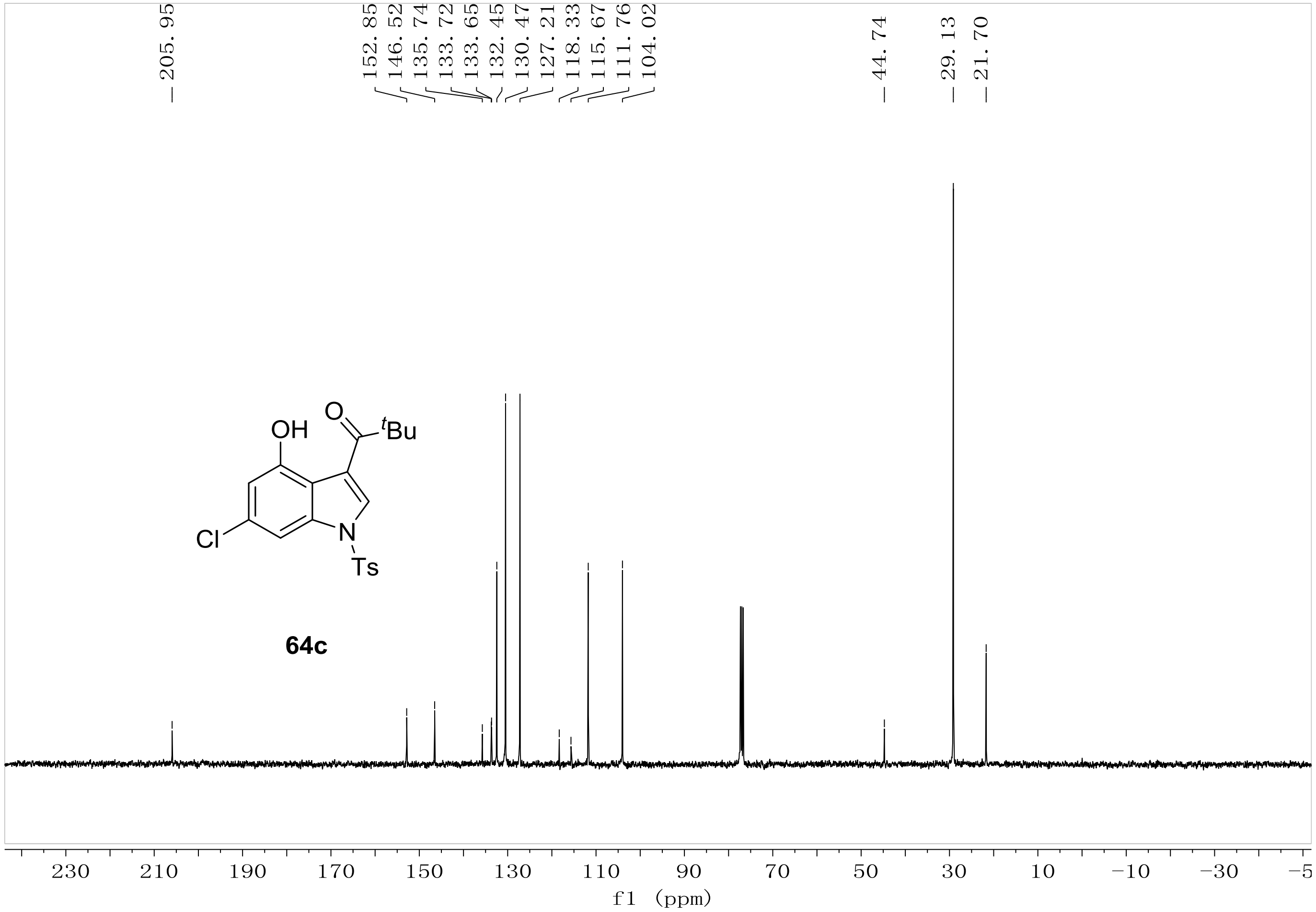

Supplementary Figure 125. ^{13}C NMR spectrum for **64c**

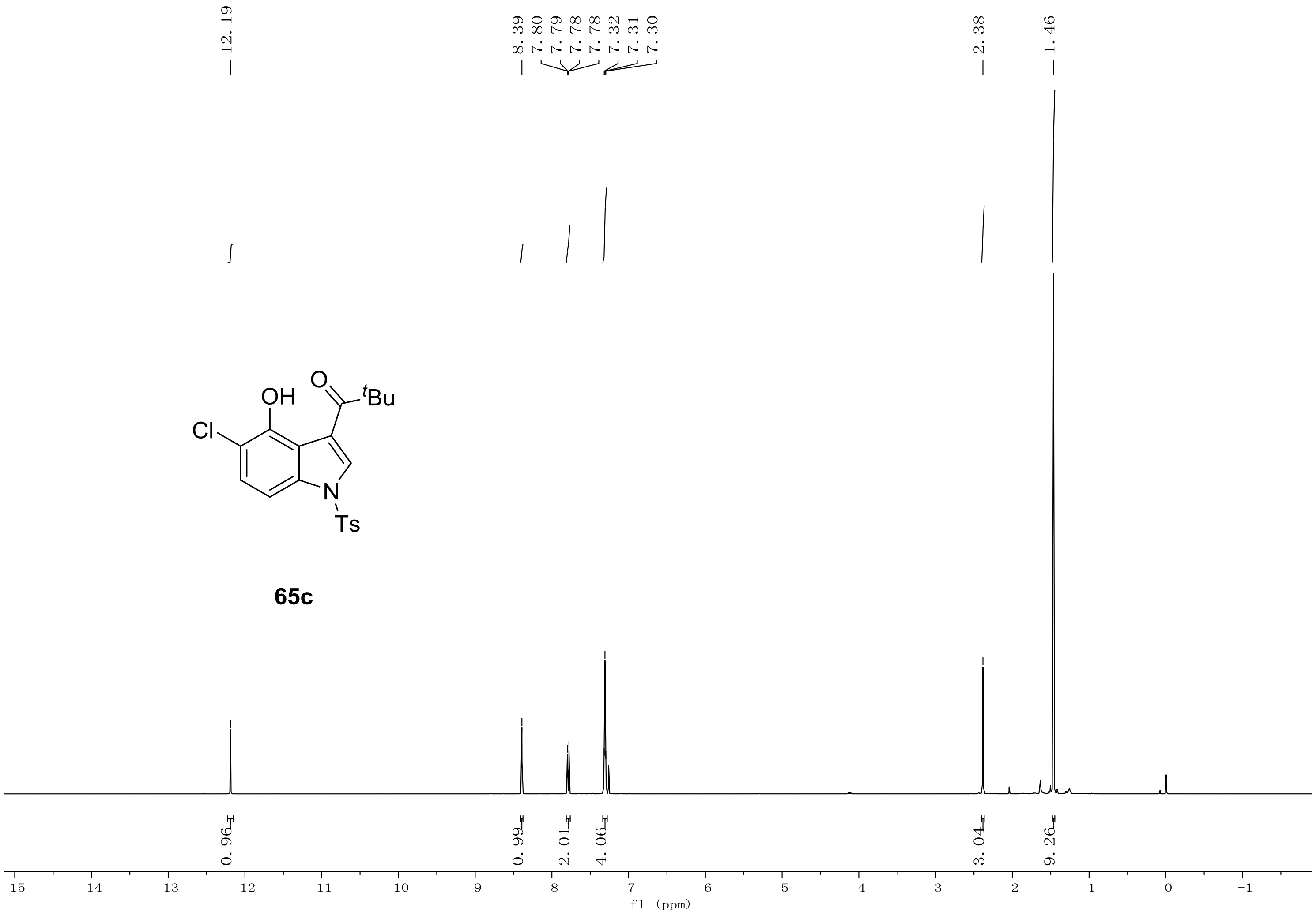

Supplementary Figure 126. ^{1}H NMR spectrum for **65c**

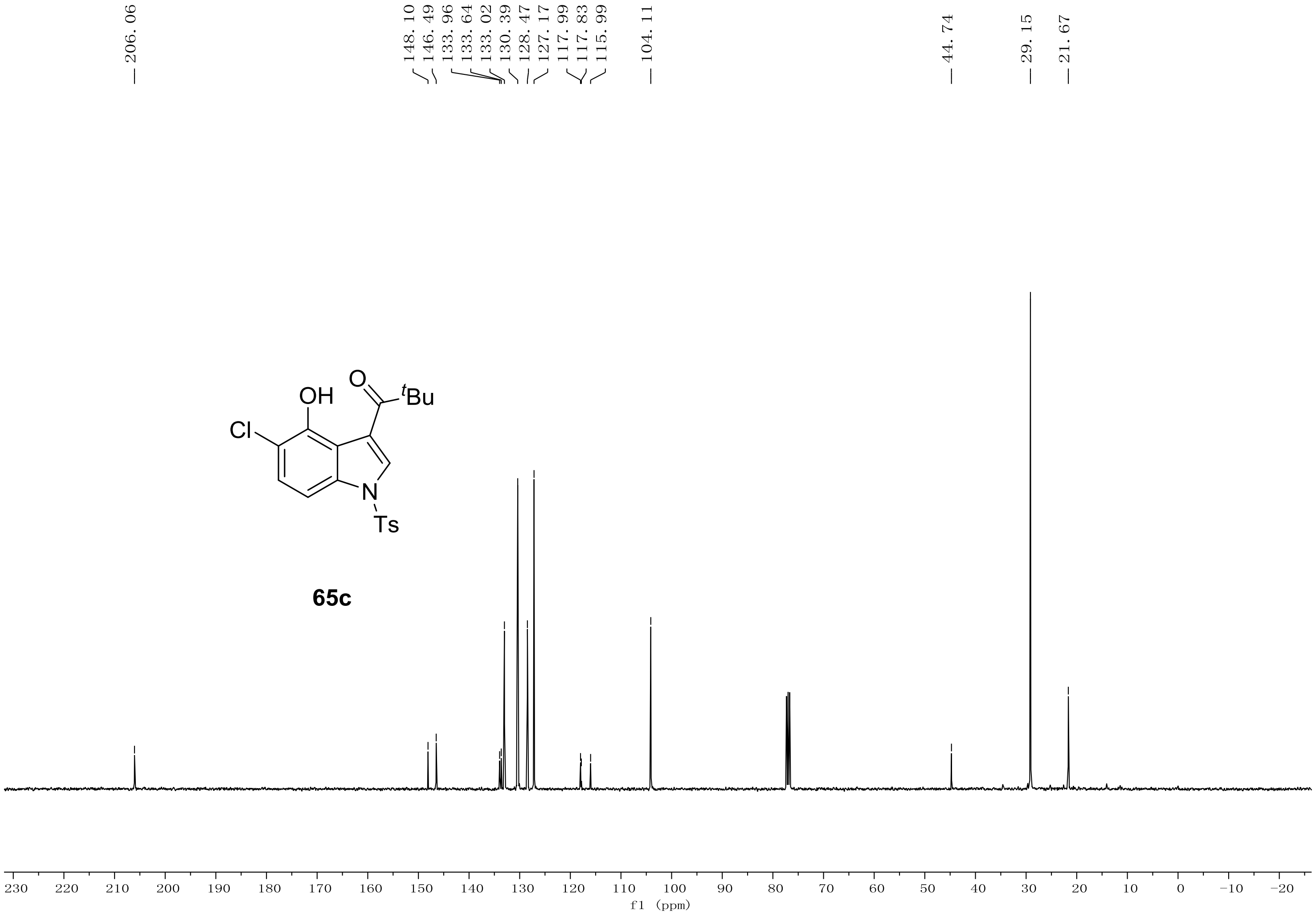

Supplementary Figure 127. ^{13}C NMR spectrum for **65c**

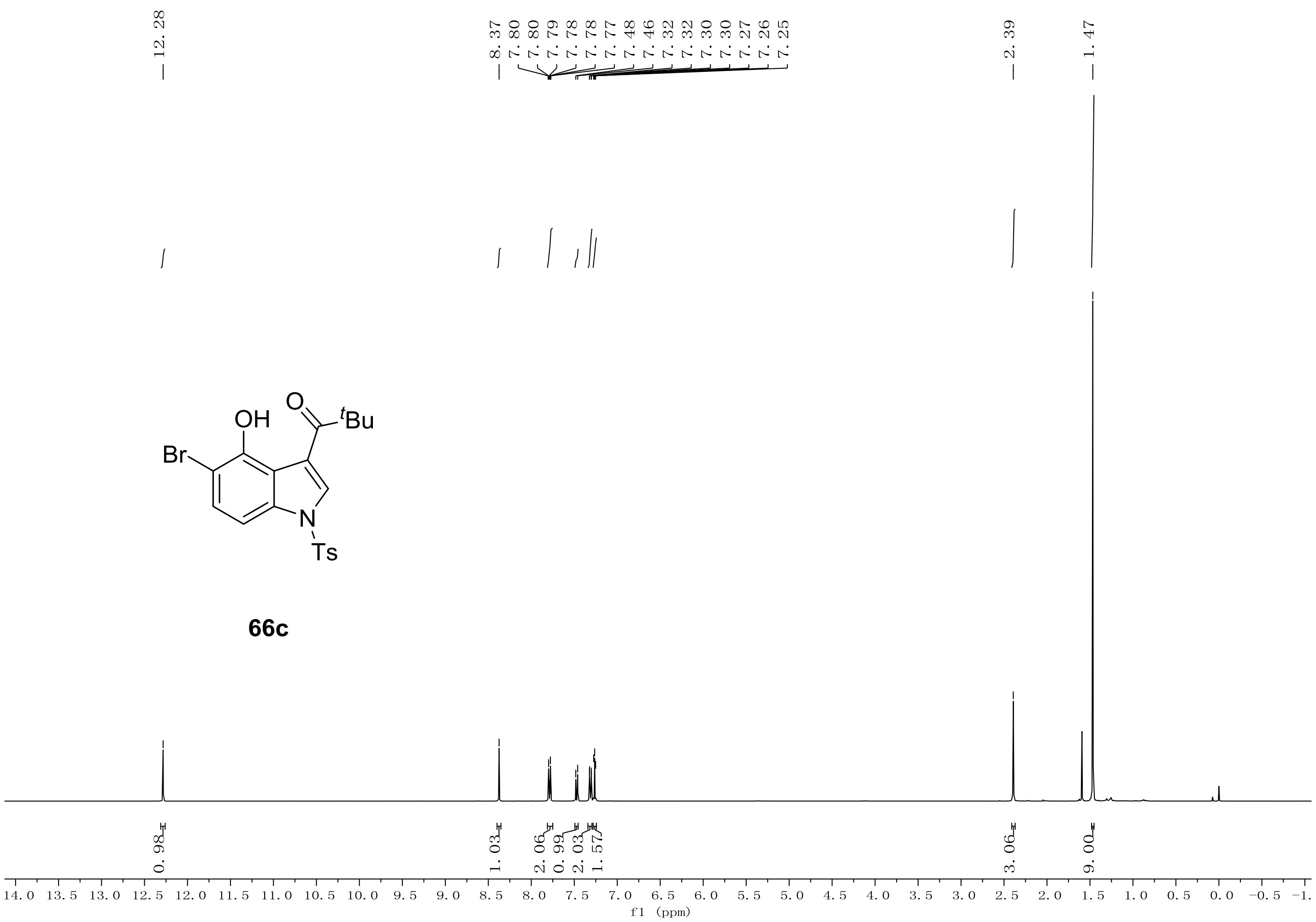

Supplementary Figure 128. ^{1}H NMR spectrum for **66c**

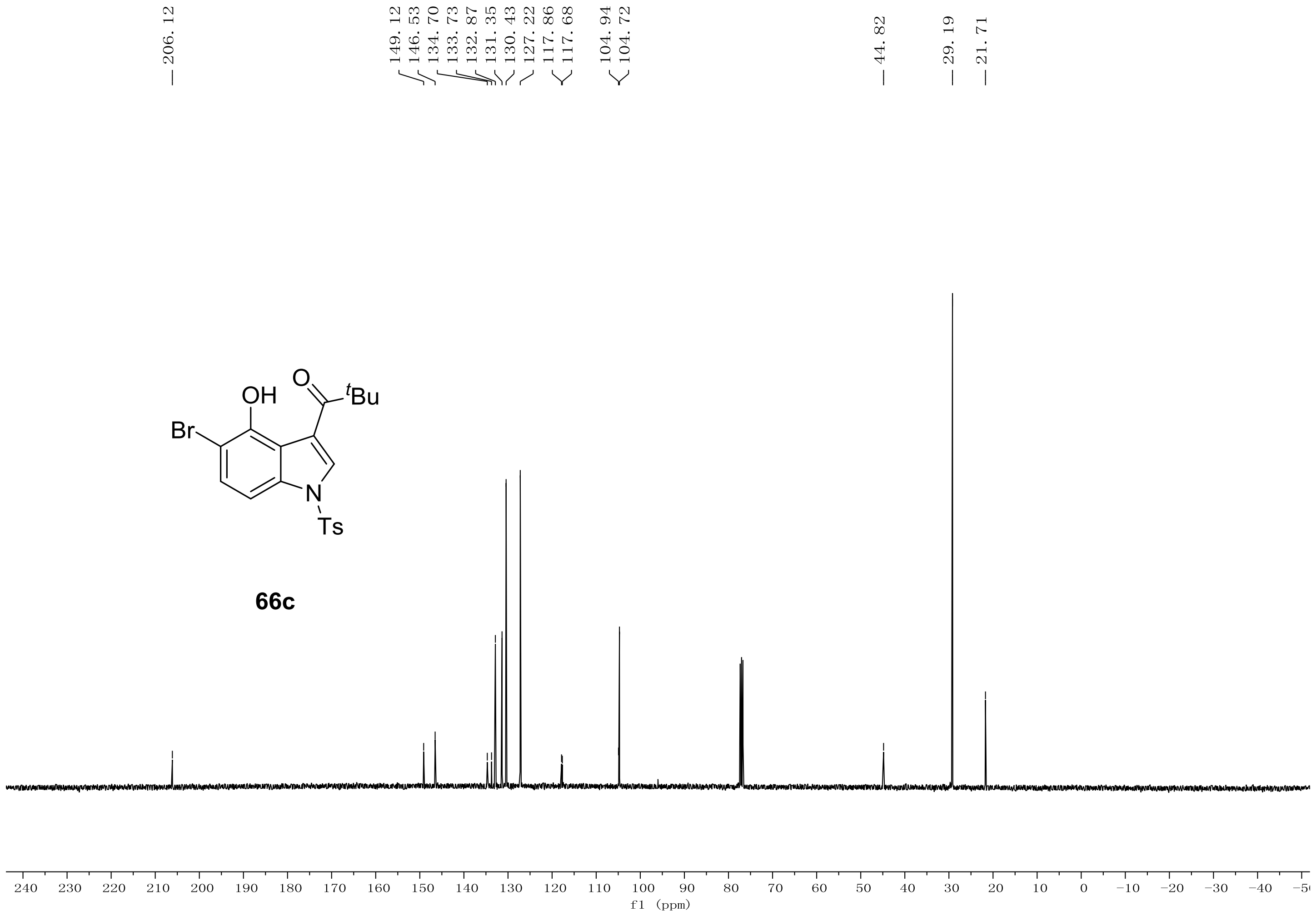

Supplementary Figure 129. ^{13}C NMR spectrum for **66c**

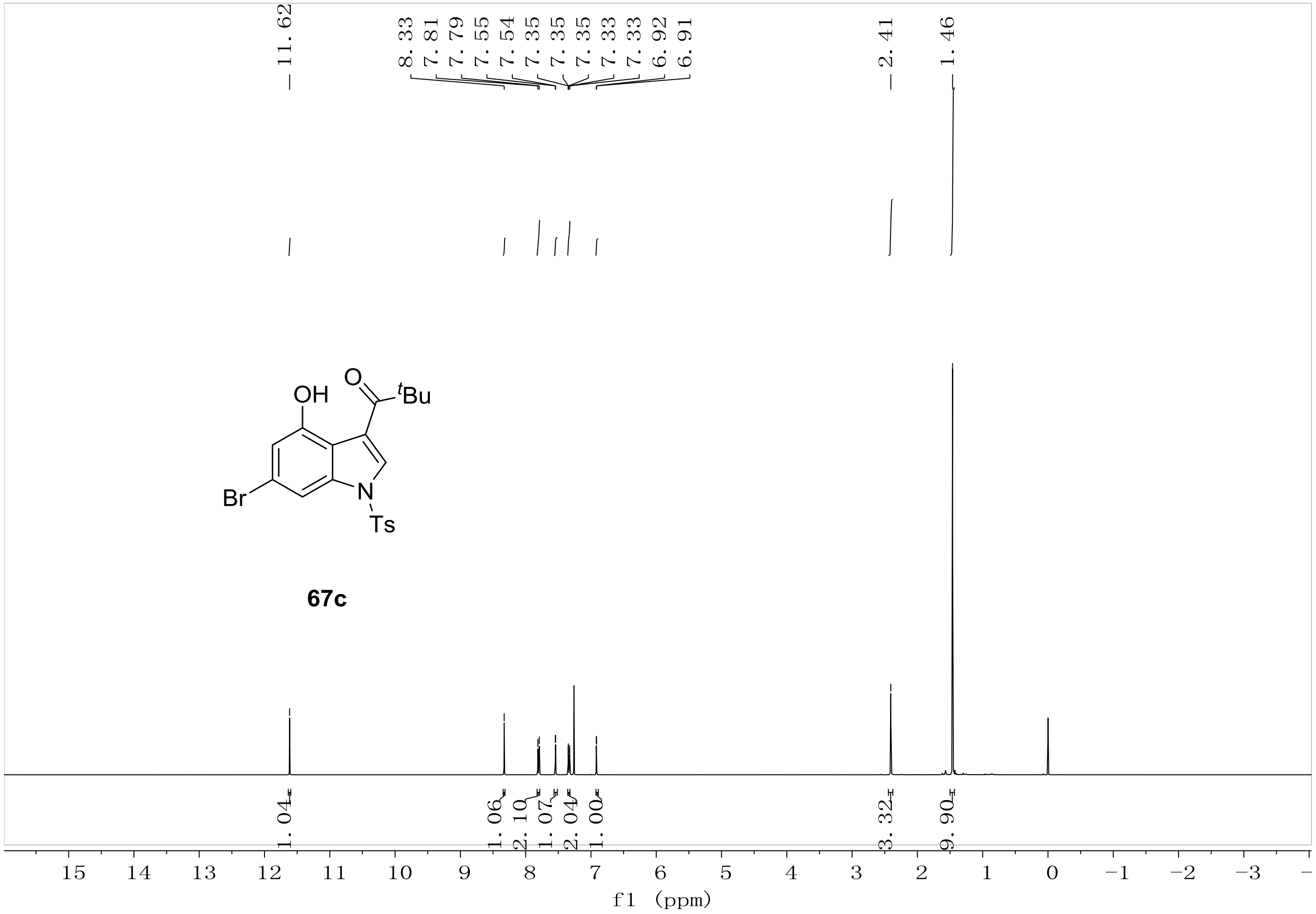

Supplementary Figure 130. ^{1}H NMR spectrum for **67c**

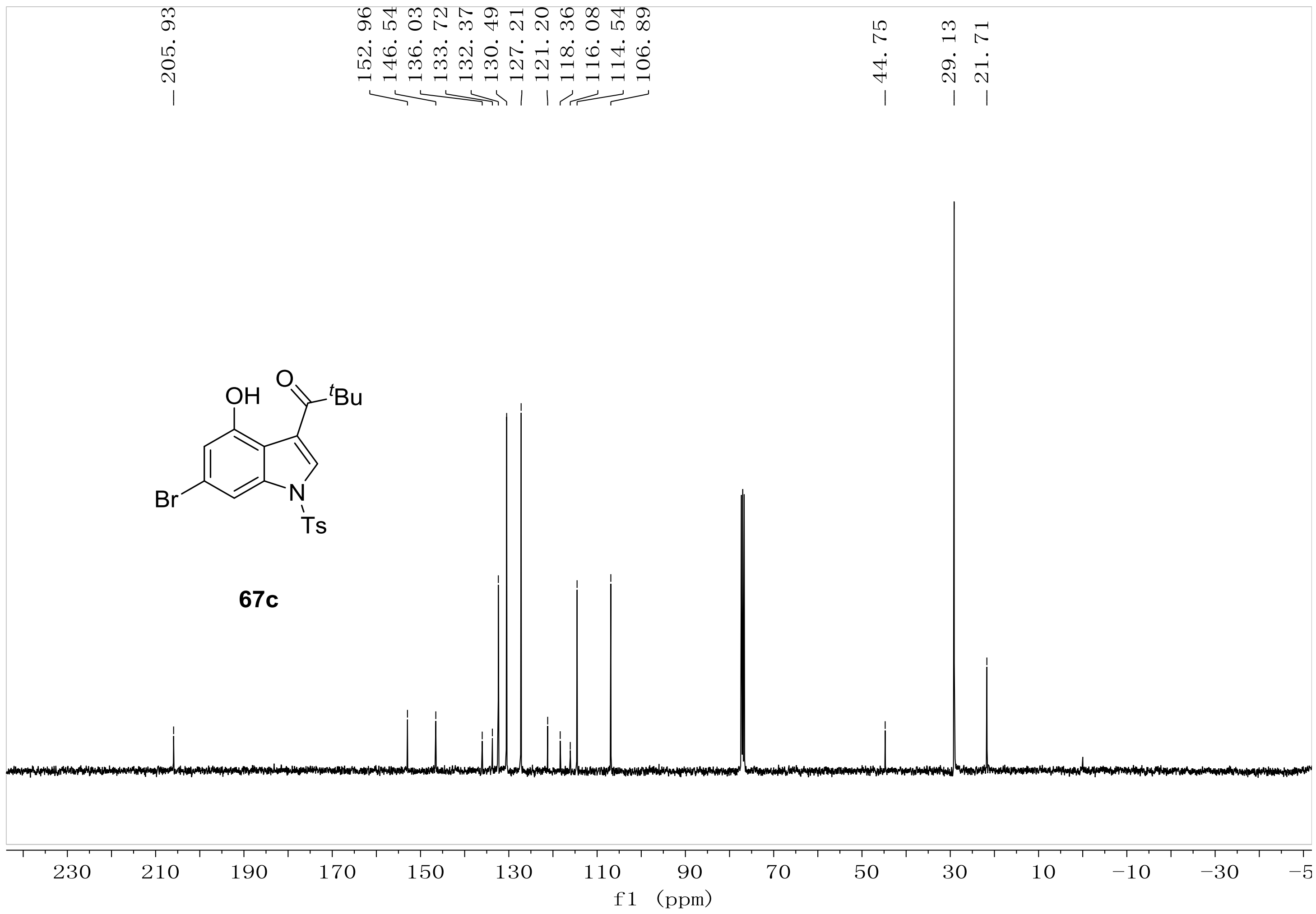

Supplementary Figure 131. ^{13}C NMR spectrum for **67c**

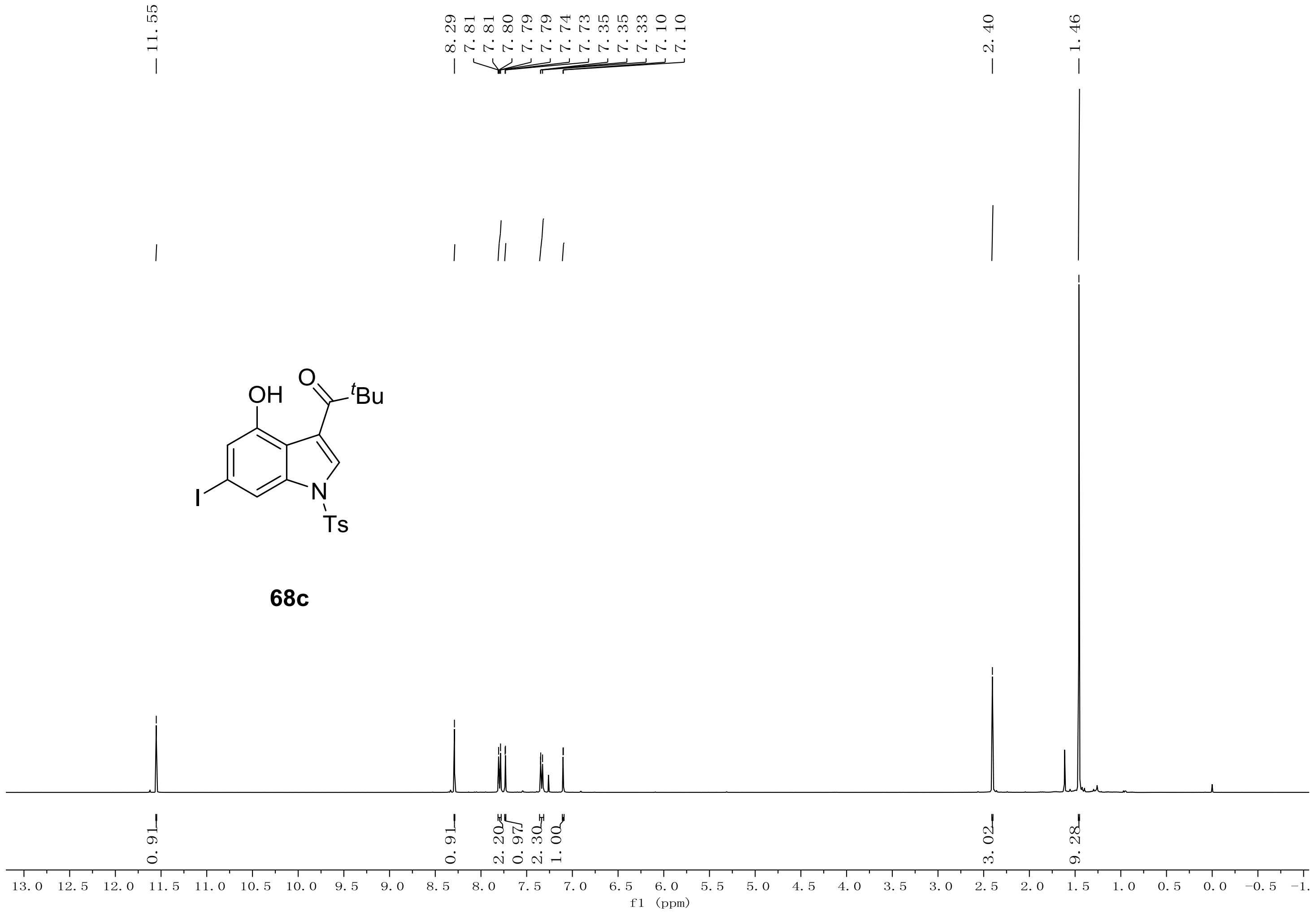

Supplementary Figure 132. ^{1}H NMR spectrum for **68c**

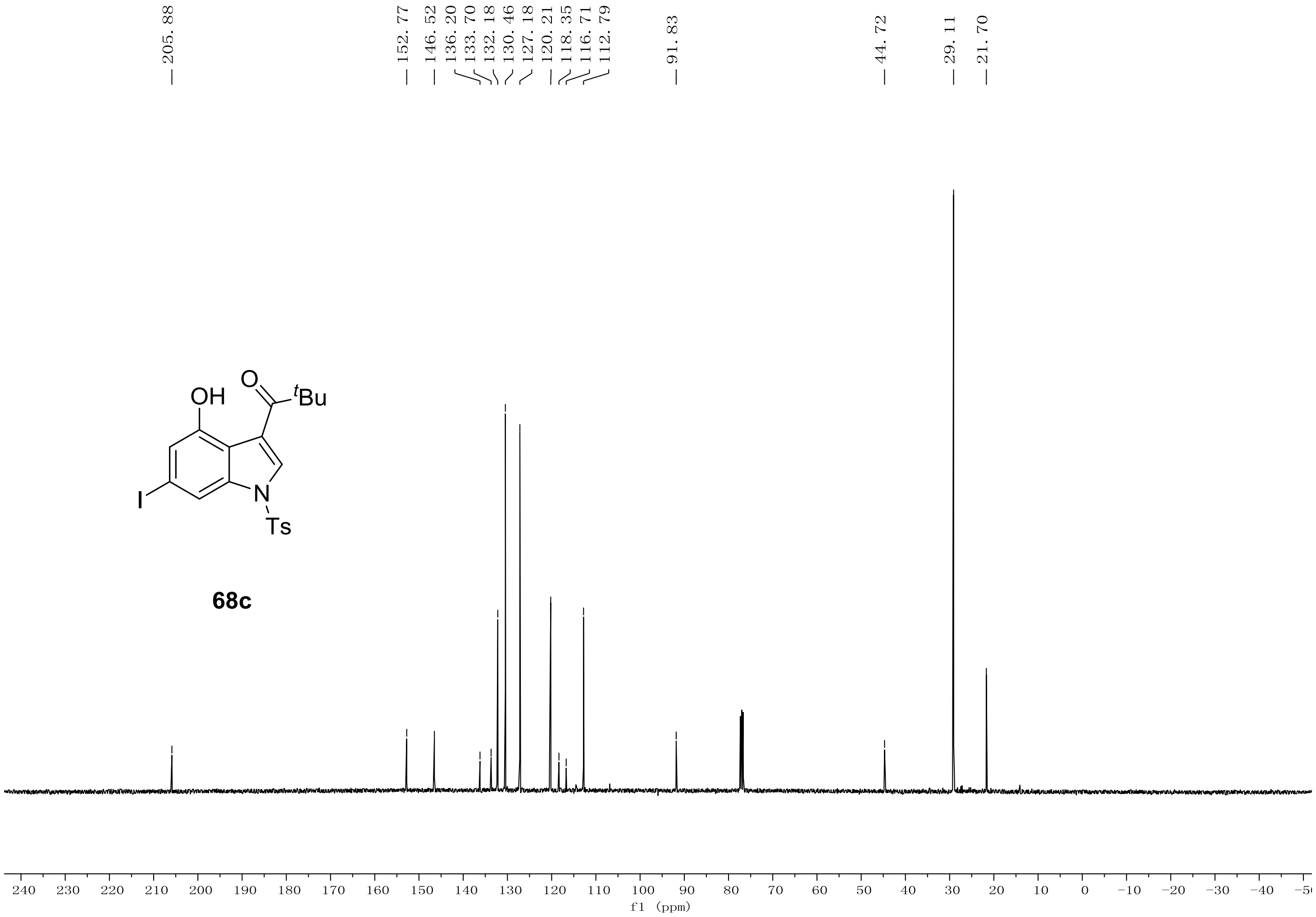

Supplementary Figure 133. ^{13}C NMR spectrum for **68c**

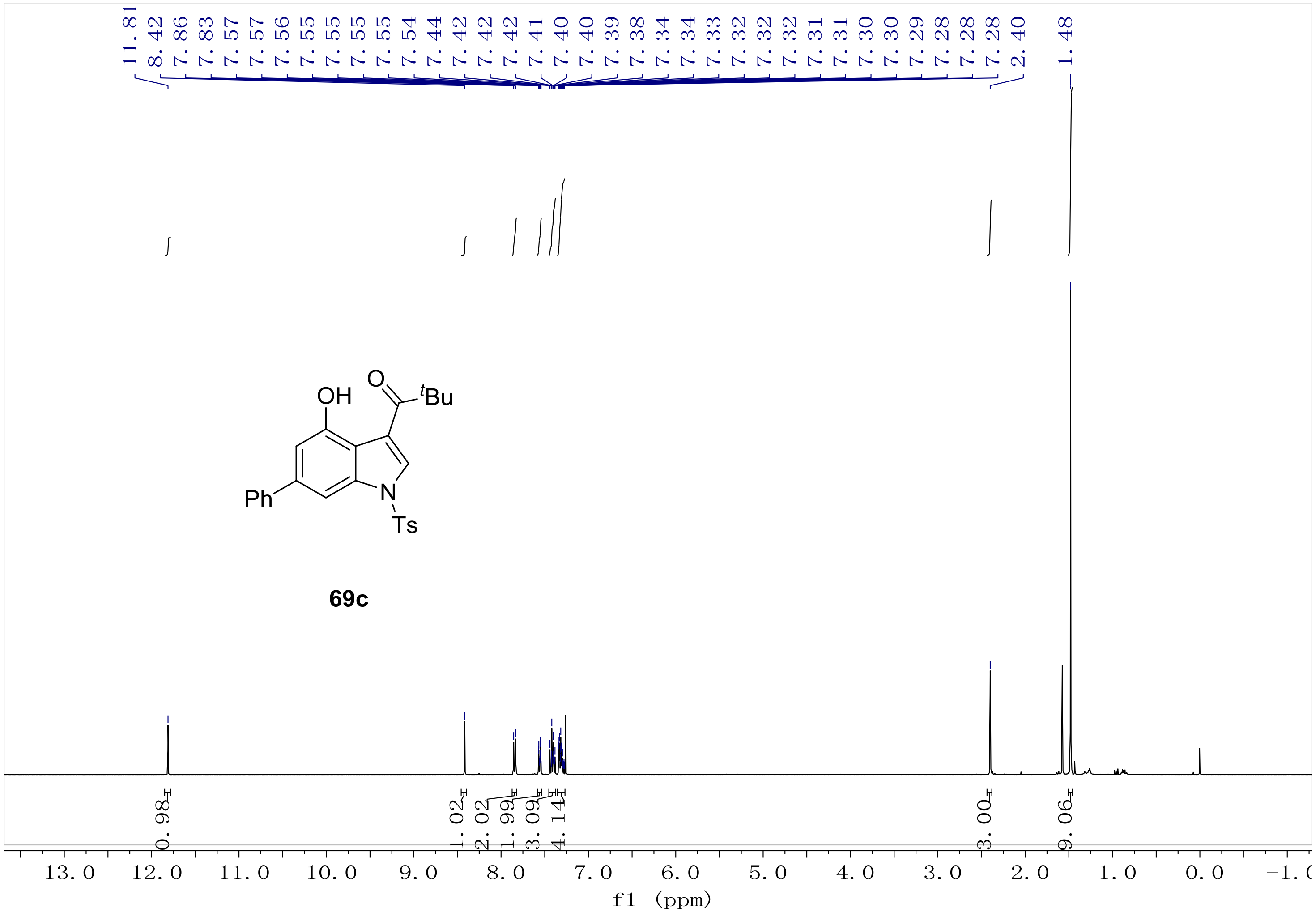

Supplementary Figure 134. ^{1}H NMR spectrum for **69c**

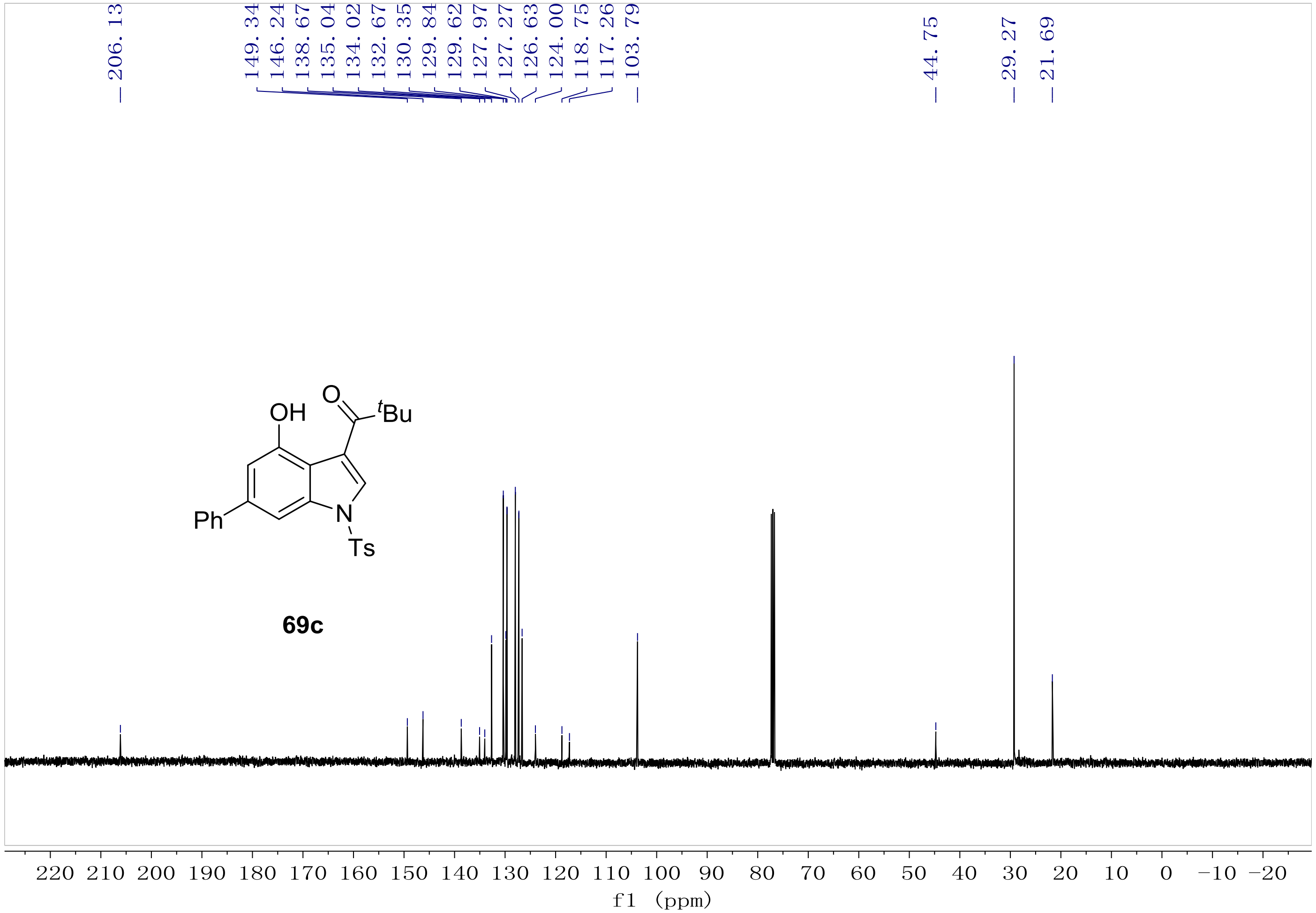

Supplementary Figure 135. ^{13}C NMR spectrum for **69c**

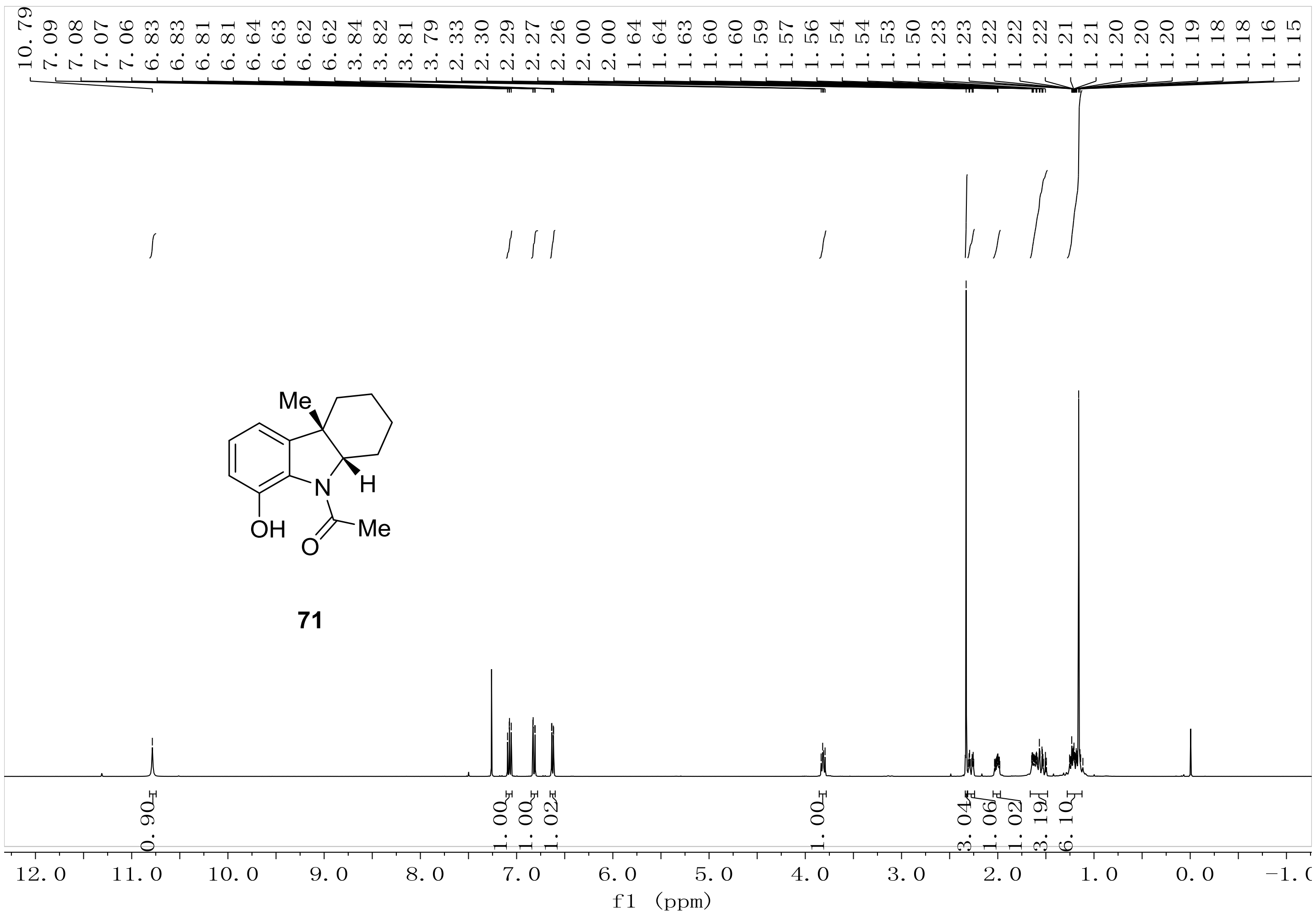

Supplementary Figure 136. ^{1}H NMR spectrum for **71**

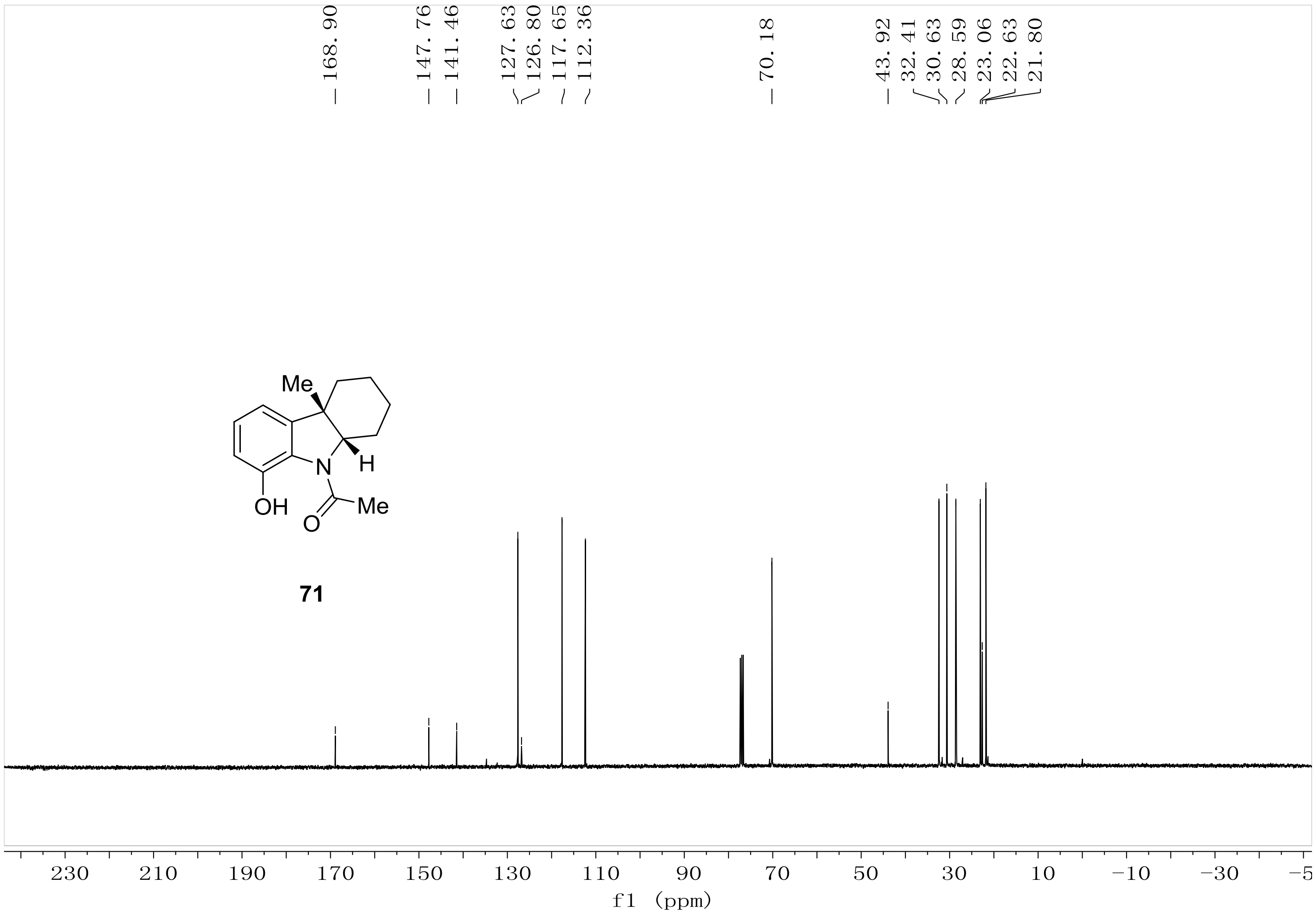

Supplementary Figure 137. ^{13}C NMR spectrum for **71**

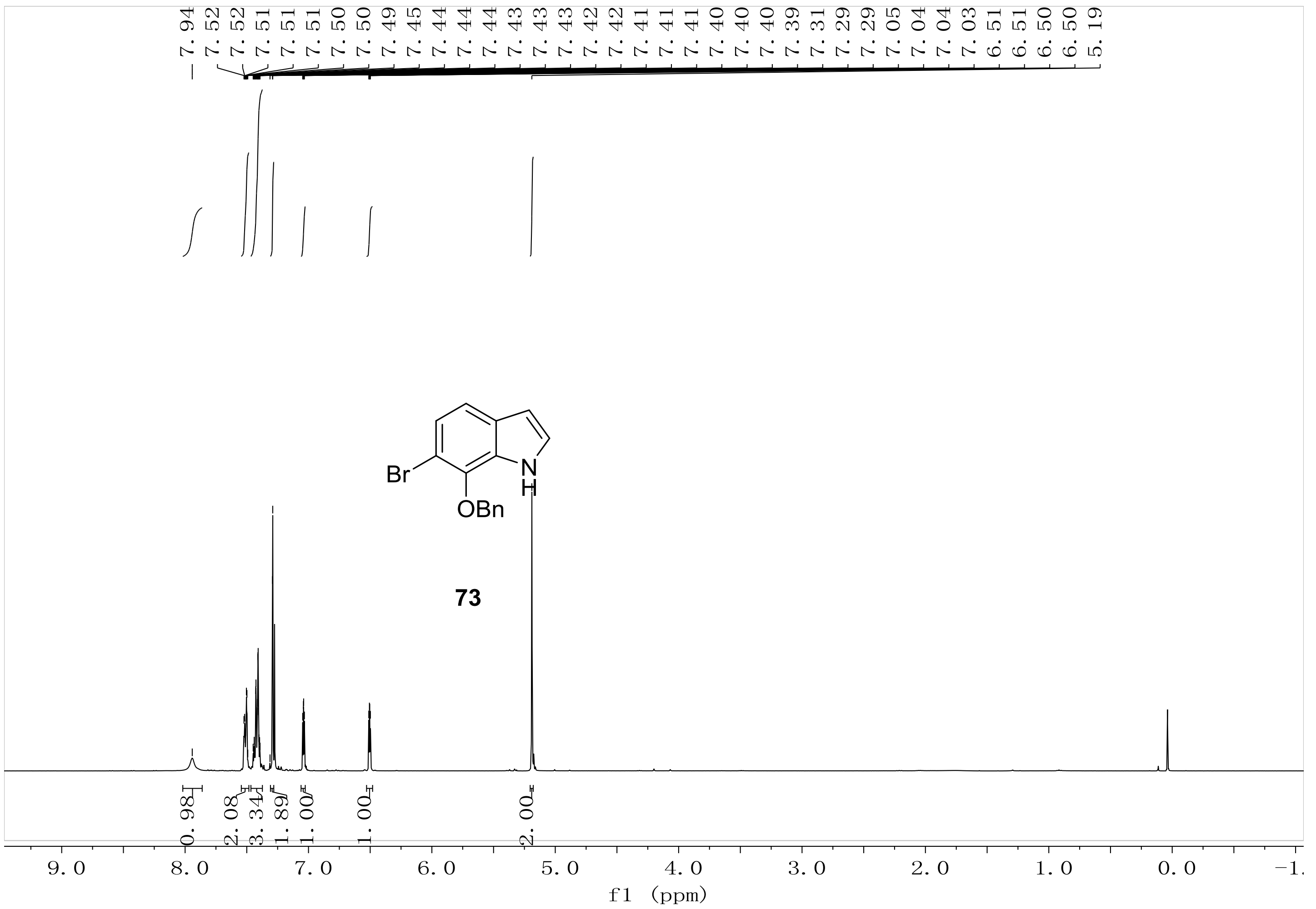

Supplementary Figure 138. ^{1}H NMR spectrum for **73**

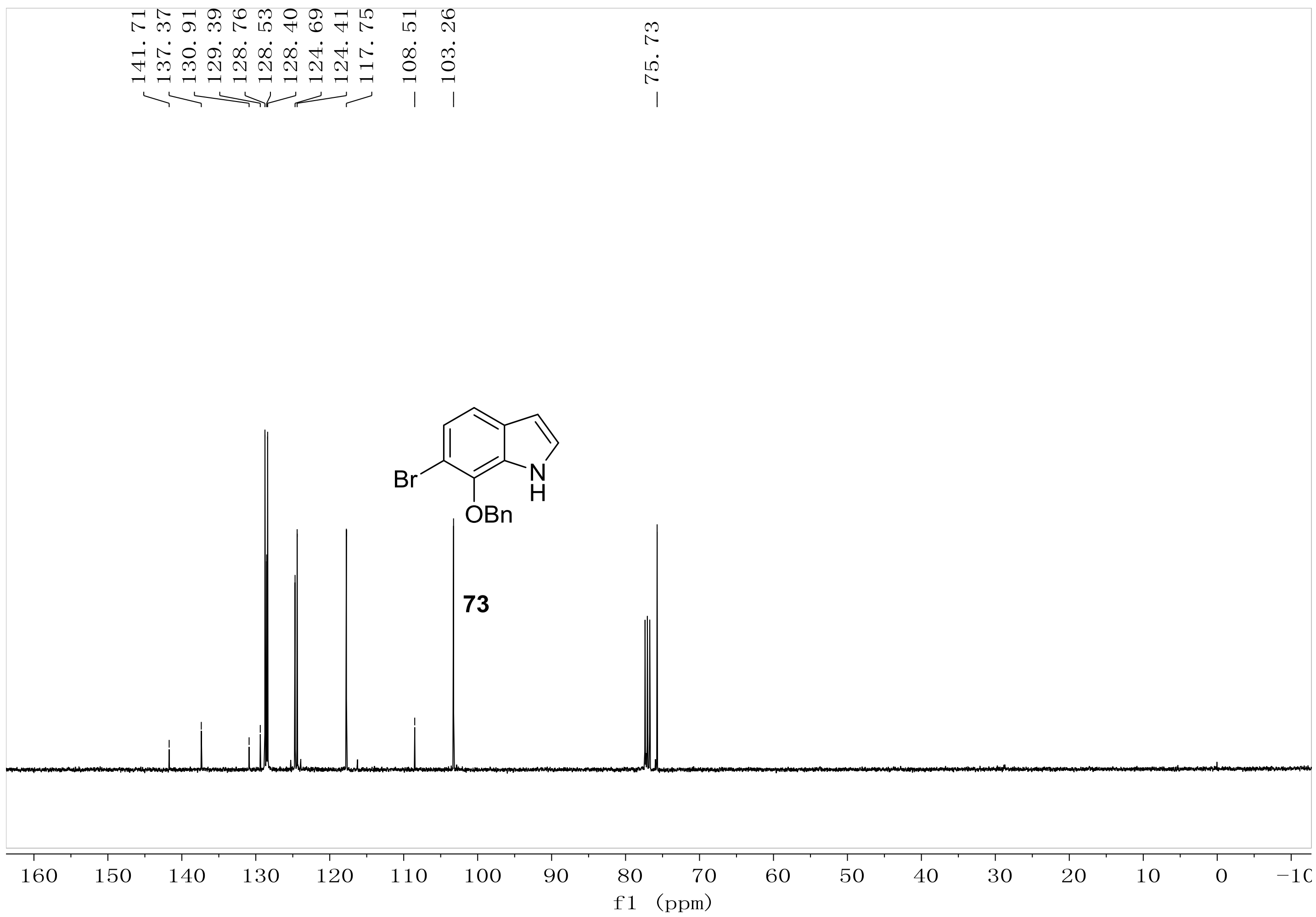

Supplementary Figure 139. ^{13}C NMR spectrum for **73**

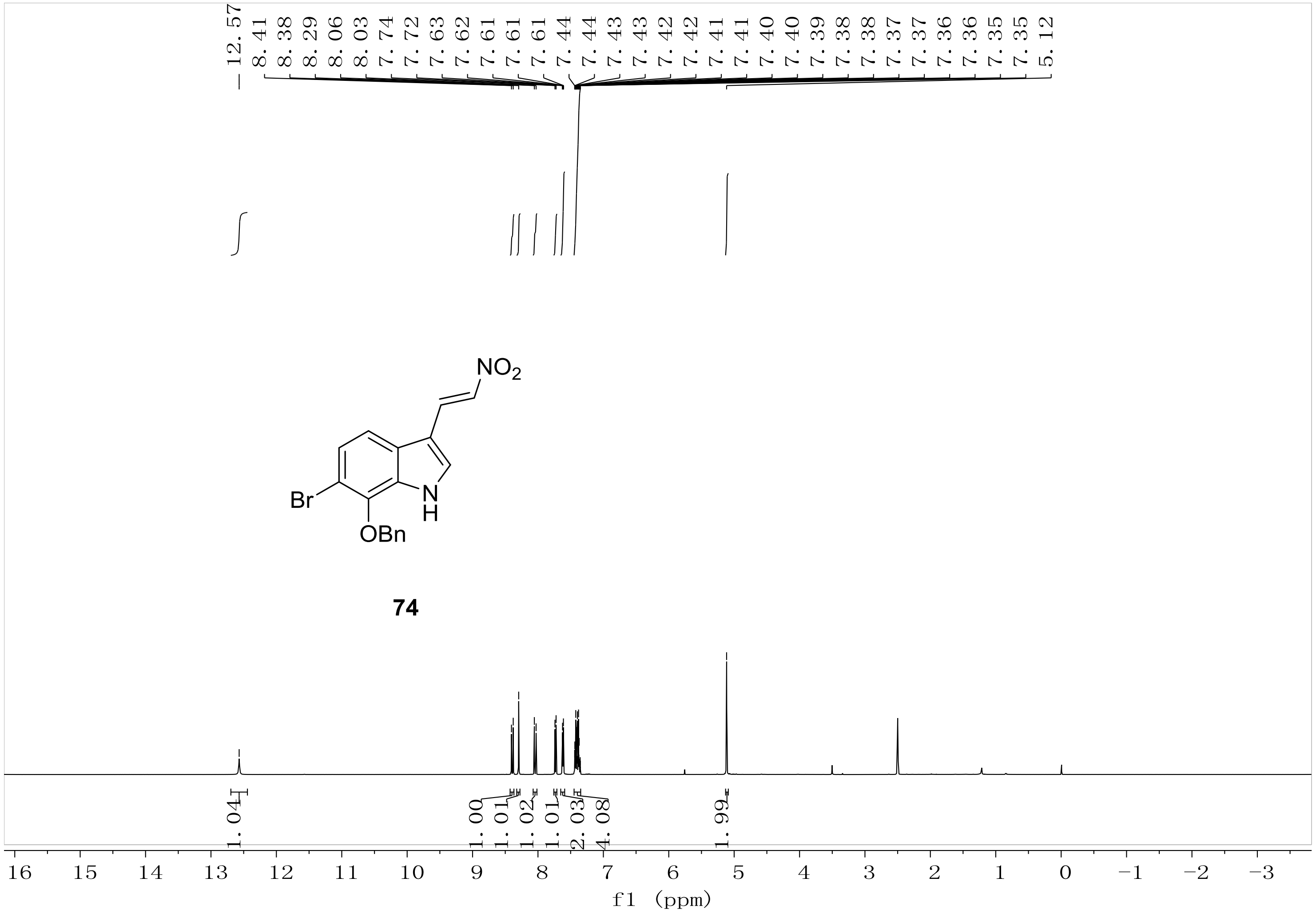

Supplementary Figure 140. ^{1}H NMR spectrum for **74**

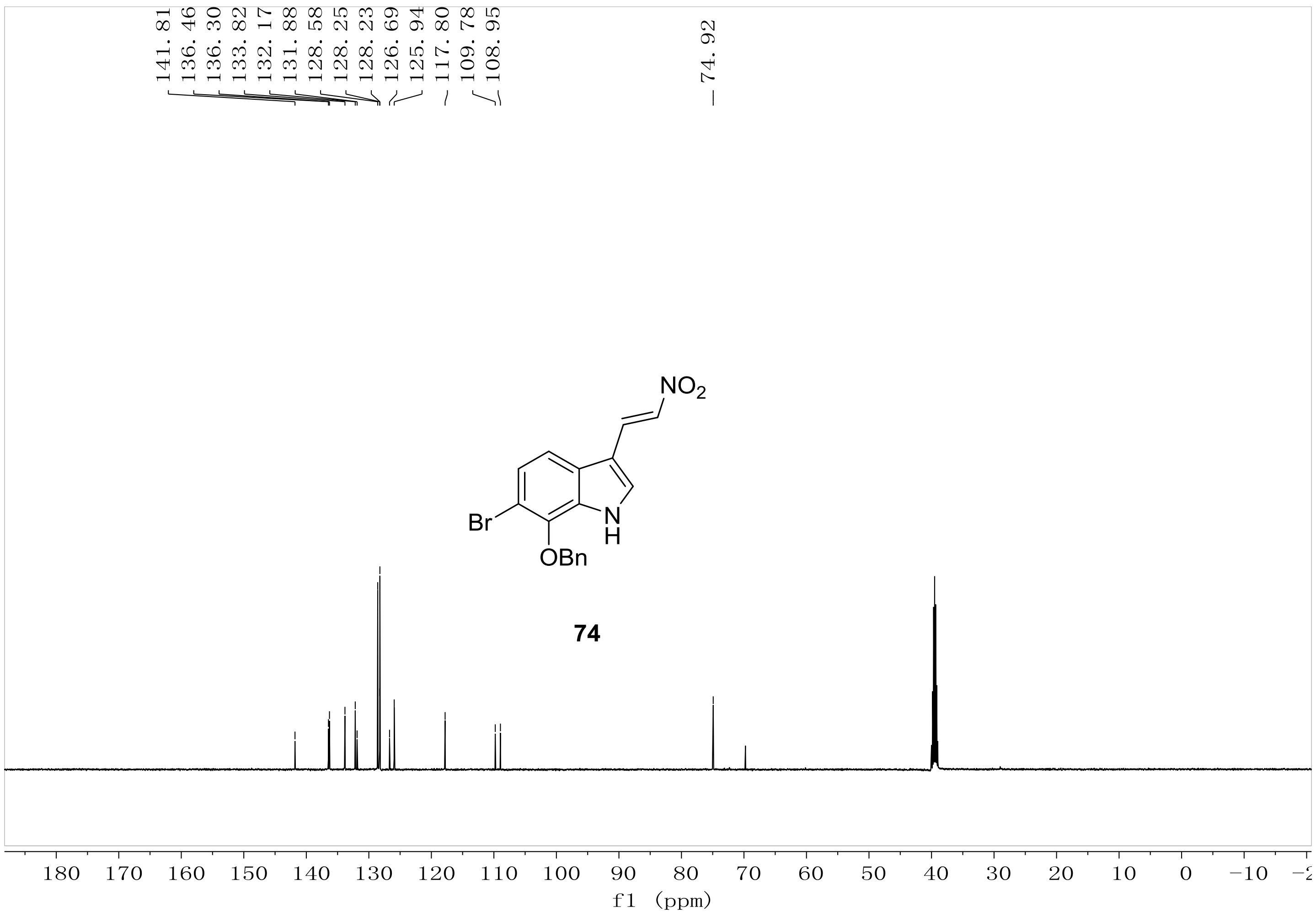

Supplementary Figure 141. ^{13}C NMR spectrum for **74**

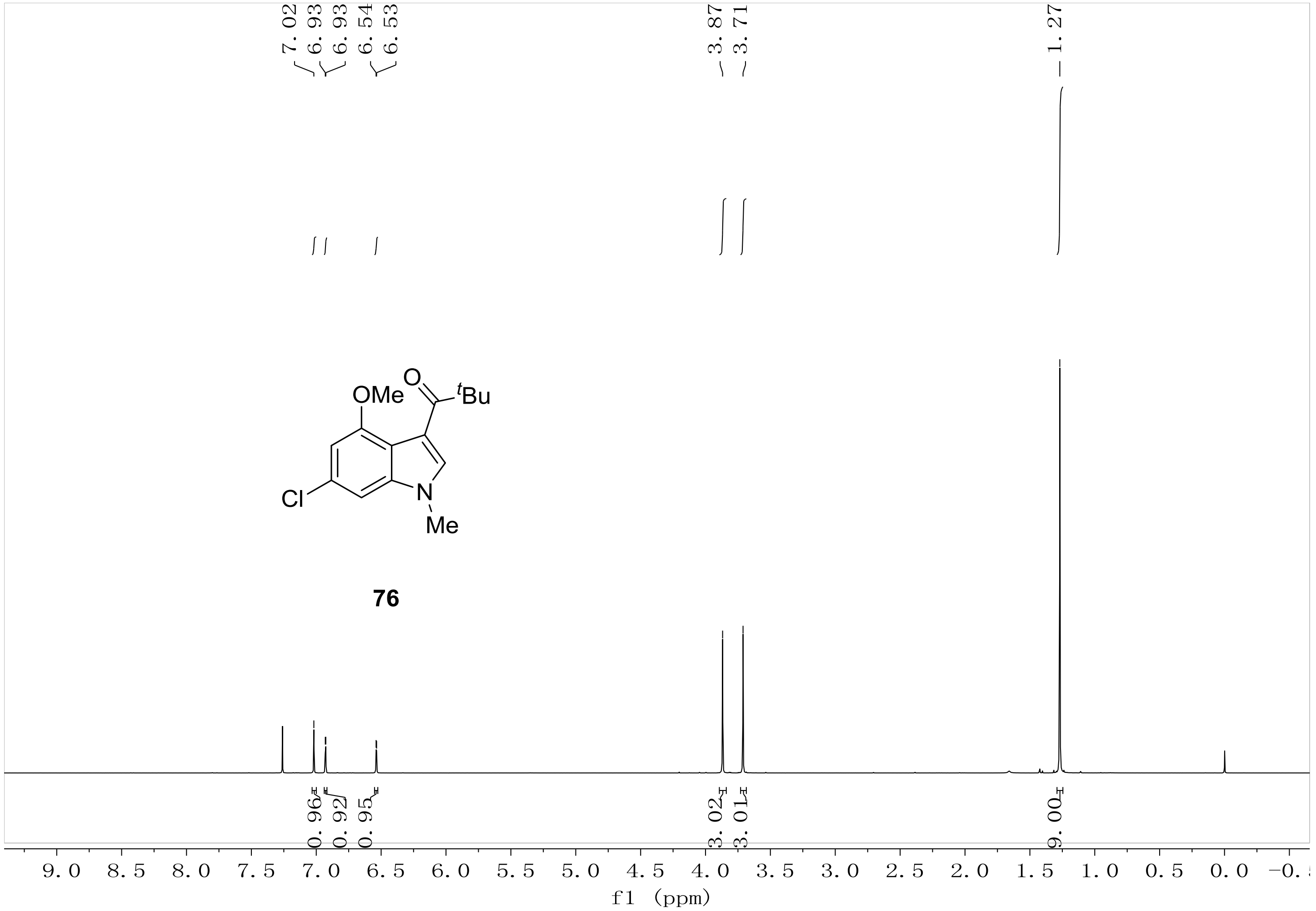

Supplementary Figure 142. ^{1}H NMR spectrum for **76**

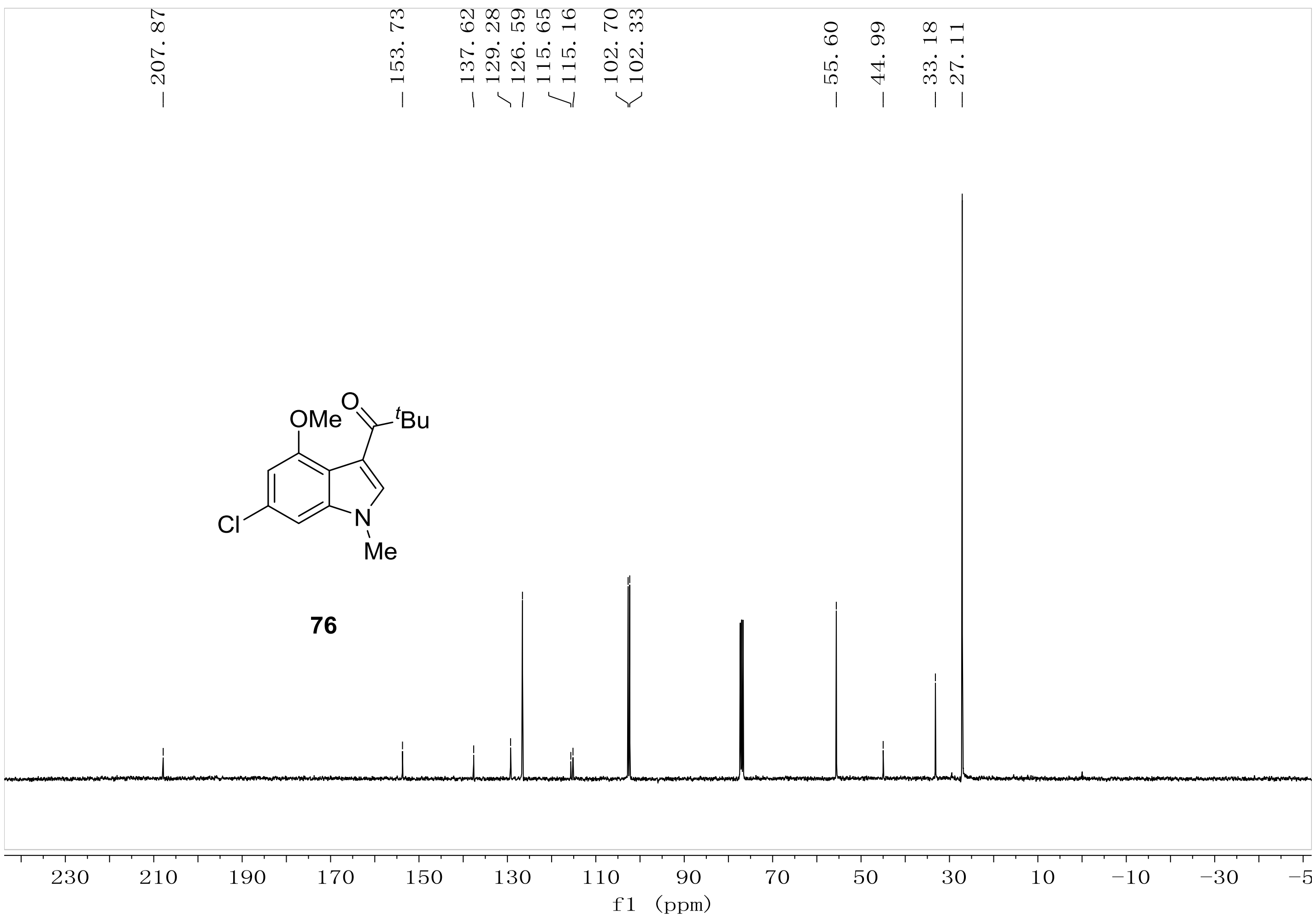

Supplementary Figure 143. ^{13}C NMR spectrum for **76**

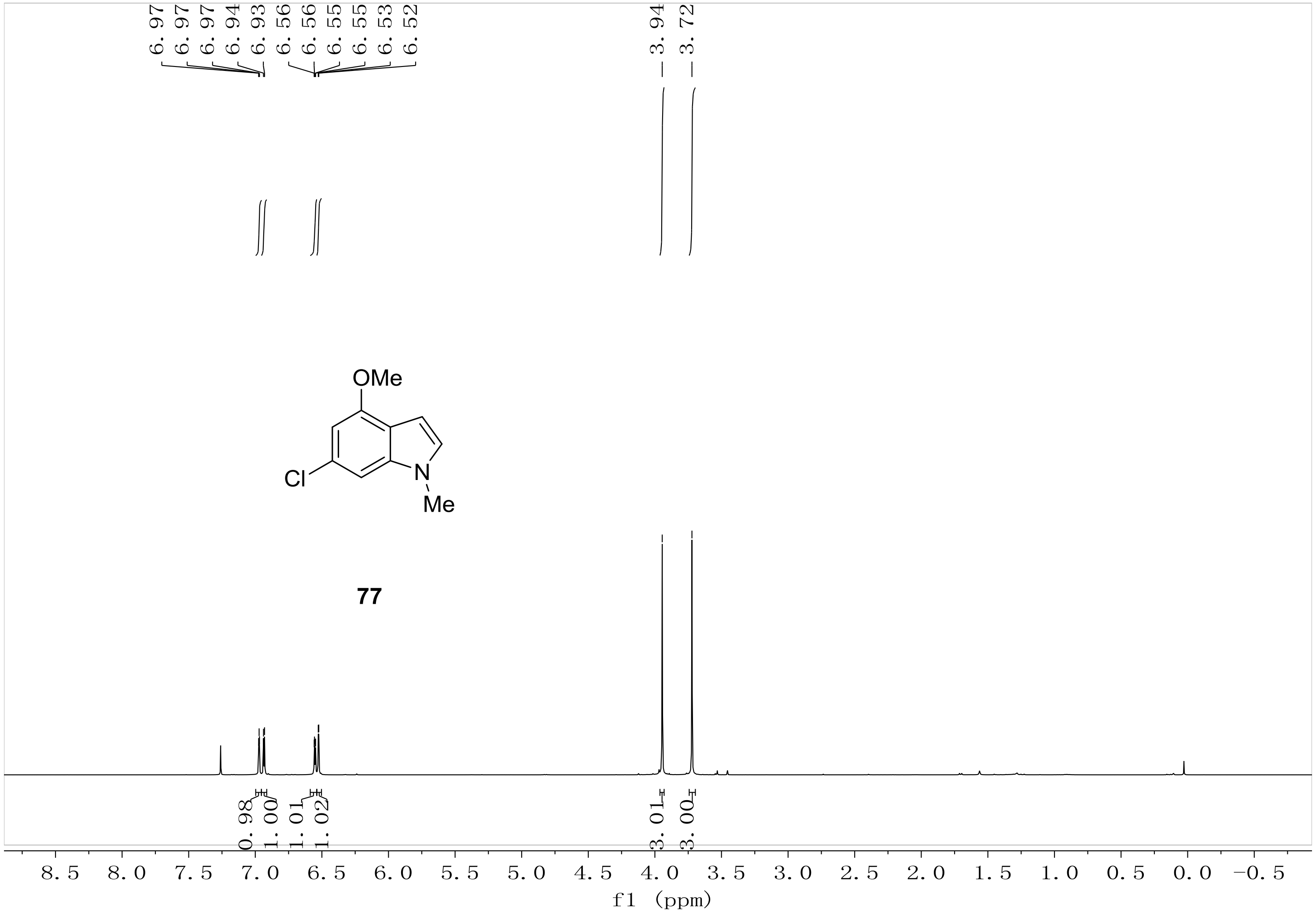

Supplementary Figure 144. ^{1}H NMR spectrum for **77**

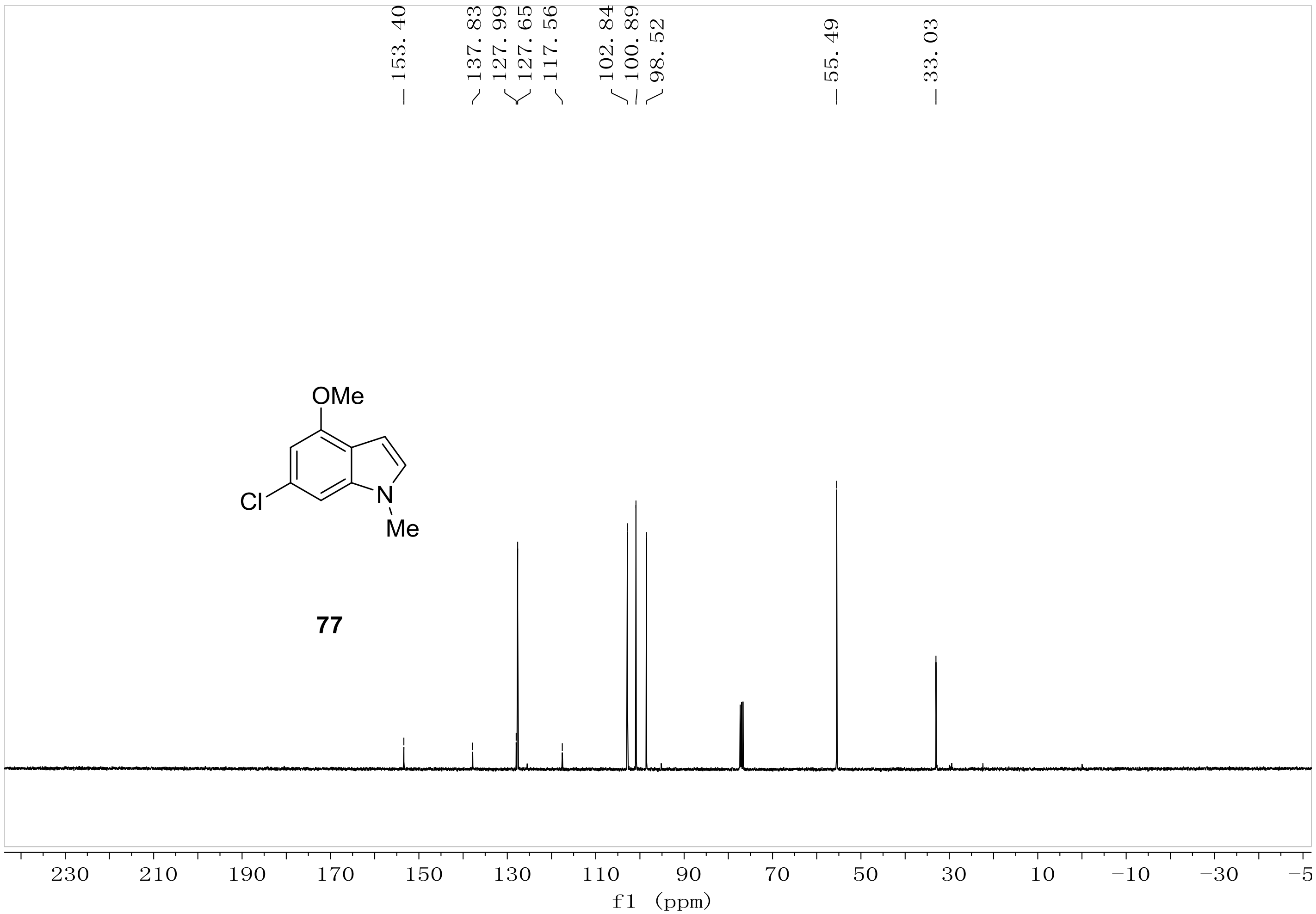

Supplementary Figure 145. ^{13}C NMR spectrum for **77**

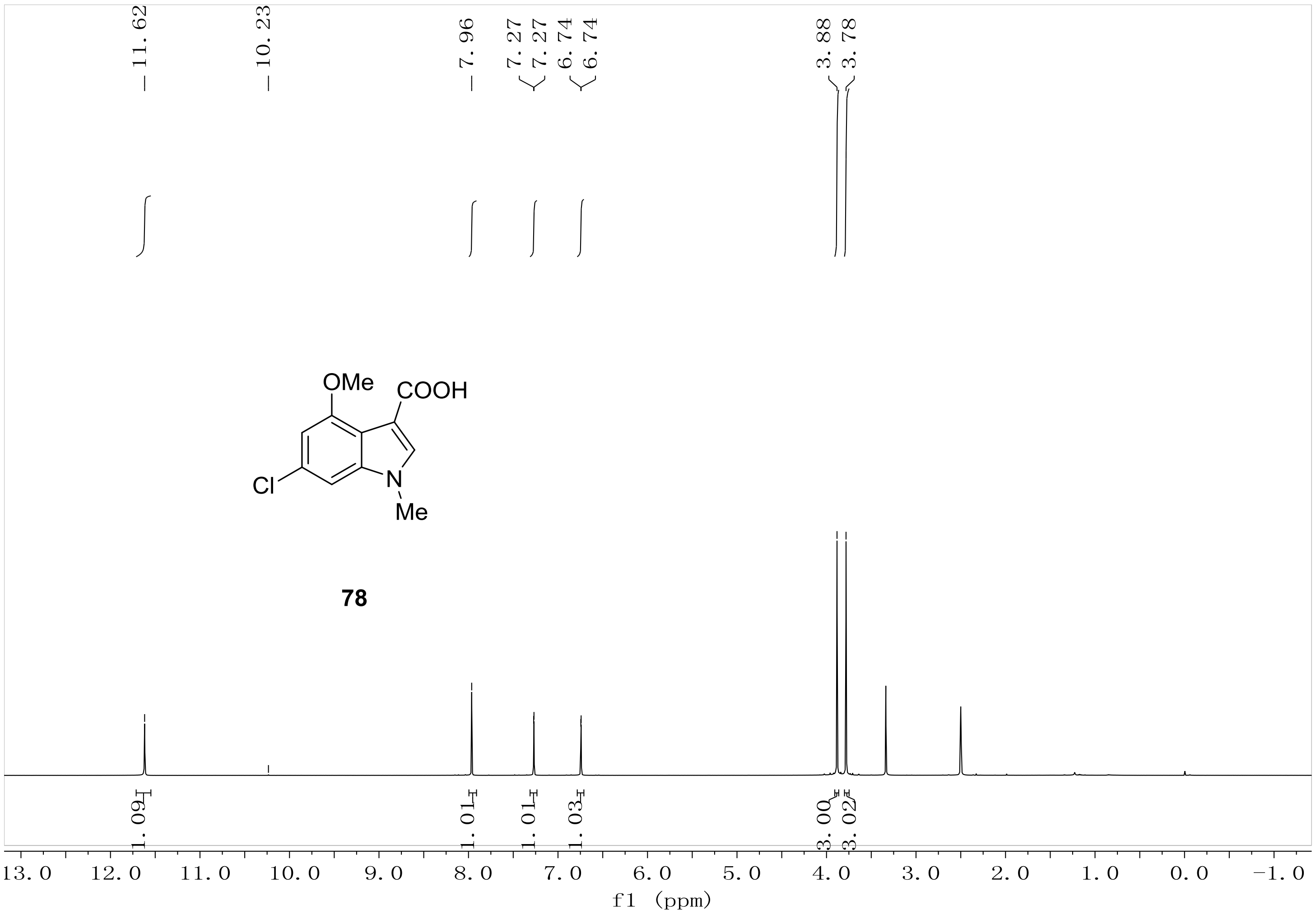

Supplementary Figure 146. ^{1}H NMR spectrum for **78**

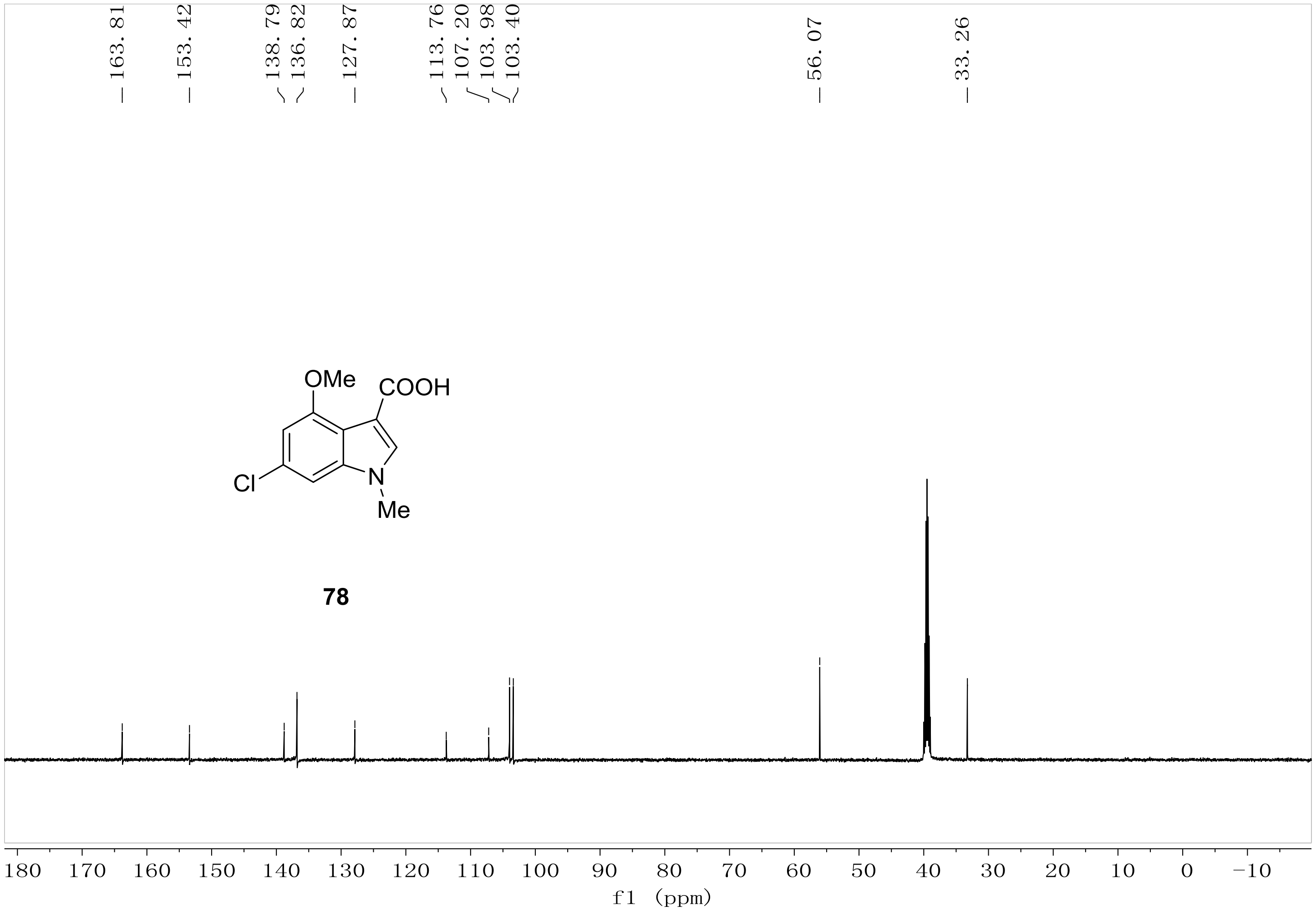

Supplementary Figure 147. ^{13}C NMR spectrum for **78**
